# Preclinical and first-in-human of purinostat mesylate, a novel selective HDAC I/IIb inhibitor, in relapsed/refractory multiple myeloma and lymphoma

**DOI:** 10.1038/s41392-025-02285-w

**Published:** 2025-06-23

**Authors:** Linyu Yang, Qiang Qiu, Jie Wang, Yi Wen, He Li, Rui Liang, Yunyu Feng, Fang Wang, Xiaojing Lin, Minghai Tang, Jianhong Yang, Heying Pei, Peng Zhao, Jishi Wang, Jin Xiang, Jia Miao, Li Zheng, Ke Tan, Yongsheng Wang, Yiguo Hu, Lijuan Chen, Weili Zhao, Ting Niu

**Affiliations:** 1 Department of Hematology, State Key Laboratory of Biotherapy and Cancer Center, West China Hospital, Sichuan University, Chengdu, China; 2 Collaborative Innovation Center of Biotherapy, West China Hospital, Sichuan University, Chengdu, China; 3 National Facility for Translational Medicine (Sichuan), West China Hospital, Sichuan University, Chengdu, China; 4 Chengdu Zenitar Biomedical Technology Co. Ltd, Chengdu, China; 5 Department of Hematology, The Affiliated Hospital of Guizhou Medical University, Guiyang, China; 6 Clinical Trial Center, West China Hospital, Sichuan University, Chengdu, China; 7 Shanghai Institute of Hematology, State Key Laboratory of Medical Genomics, National Research Center for Translational Medicine at Shanghai, Ruijin Hospital Affiliated to Shanghai Jiao Tong University School of Medicine, Shanghai, China; 8 Pôle de Recherches Sino-Français en Science du Vivant et Génomique, Laboratory of Molecular Pathology, Shanghai, China

**Keywords:** Haematological cancer, Drug development

## Abstract

Simultaneously targeting key pathogenic drivers and remodeling of the tumor microenvironment represents a critical therapeutic strategy for relapsed or refractory (r/r) multiple myeloma (MM) and lymphoma. Purinostat mesylate (PM), a highly selective HDAC I/II binhibitor, exhibits excellent antitumor activity in MM and lymphoma cell lines and mouse models, outperforming the pan-HDAC inhibitor panobinostat or first-line/second-line multi-drug combinations. Different from panobinostat, bulk RNA-seq analysis revealed that PM suppressed essential tumor survival factors and triggered inflammation and interferon responses. The scRNA-seq of 5TMM models further indicated that PM enhanced antitumor immunity by boosting monocyte- and T cell-mediated immune responses. In a phase I trial (NCT05526313; N = 29) of PM at doses up to 15 mg/m², treatment-related Grade ≥3 adverse events predominantly comprised hematologic toxicities: thrombocytopenia (75.9%), neutropenia (55.2%), leukopenia (41.4%), and lymphopenia (31.0%), with no dose-limiting toxicities observed. PM monotherapy achieved a disease control rate of 72.7% (8/11) and an objective response rate (ORR) of 9.1% (1/11) in r/r MM. Notably, r/r lymphoma patients showed an ORR of 61.6% (11/18), particularly reaching 63.6% (7/11) with 6 complete responses in diffuse large B-cell lymphoma (DLBCL). Treatment responders exhibited enhanced immune activation, with elevated CD3^+^CD8^+^ T cells and increased cytokine levels, such as IFN-γ and CXCL10. Overall, PM is safe and moderately effective in MM, but highly effective in lymphoma. Additionally, PM combined with pomalidomide and dexamethasone showed strong synergistic activity in r/r MM treatment. These findings support further open-label, multicenter phase Ib/IIa trials of PM combination therapy with immunomodulators for r/r MM, as well as phase II monotherapy trials for r/r DLBCL and r/r T-cell lymphoma.

## Introduction

In the past 30 years, the incidence of multiple myeloma (MM) and lymphoma has been on the rise worldwide.^1^ Substantial progress has been achieved in MM and lymphoma treatment, benefiting from the development of novel chemotherapies and biological therapeutics.^2–4^ Despite these advancements, MM remains incurable, and a significant number of lymphomas are prone to relapse and refractory challenges, and limited treatment options pose a challenge to control disease progression.^4,5^ The development of novel agents or exploration of therapeutic strategies for relapsed or refractory (r/r) MM and lymphoma treatment are still unmet needs.

Histone deacetylases (HDACs) are subdivided into class I (HDAC1, 2, 3, and 8), class IIa (HDAC4, 5, 7, and 9), class IIb (HDAC6 and 10), class III (Sirtuins), and class IV (HDAC11). HDAC inhibitors (HDACis) promote an open chromatin structure by sustaining acetylated histones, thereby facilitating the transcription of relevant tumor suppressor genes and immune-regulatory genes.^6,7^ Currently, five HDACis including pan-HDACis vorinostat, belinostat, panobinostat, and selective inhibitors romidepsin and chidamide, are approved under various jurisdictions.^8,9^ Romidepsin, a macrocyclic depsipeptide derived from a natural product, is a selective inhibitor of HDAC1 and HDAC2, exhibiting weaker inhibitory activity against HDAC4 and HDAC6.^8^ Chidamide, which has a benzamide structure, inhibits HDAC1, 2, 3, and 10, and has weaker effects on HDAC8 and HDAC11.^9^ Panobinostat was the most active among the approved HDACis and the only one approved for the treatment of r/r MM in combination with bortezomib and dexamethasone. Unfortunately, its approval was withdrawn in 2021 as the manufacturer declined to perform the confirmatory studies required to support its initial accelerated approval. The abnormal expression or dysregulation of class I and IIb HDACs in MM and lymphoma is closely associated with the occurrence of tumors and poor prognosis.^6,10,11^ Osteolysis and hypercalcemia are common clinical symptoms associated with MM patients.^12^ It has been reported that HDAC 1, 2, and 3 play crucial roles in osteoclastogenesis, and HDACis inhibit osteoclast differentiation by targeting the RANKL/RANK pathway.^13,14^ Class IIa HDAC isoforms are primarily expressed in the heart, brain, and skeletal muscle, and genetic evidence confirms their crucial role in tissue-specific growth and development.^7,15,16^ Moreover, the involvement of class IIa and IV HDACs in tumorigenesis remains contentious, with accumulating evidence suggesting that inhibiting class IIa and IV may lead to more adverse effects associated with cardiovascular toxicity and immunosuppression.^17,18^

It is well known that improving the immune microenvironment can benefit r/r MM and lymphoma treatment.^19,20^ Previous studies have shown that class I HDACis enhance the functionality of NK and CD8^+^ T cells while decreasing the number and activity of Tregs to exert antitumor immune responses in various tumor models.^20,21^ Inhibition of HDAC6 has been reported to reduce the percentage of myeloid-derived suppressor cells (MDSCs) and Tregs and suppress the expression of PD-1 on CD8^+^ T cells in BM cells of myeloma patients.^22^ Inhibition of HDAC10 was essential for NK cell-mediated antitumor immunity,^23^ and disruption of HDAC11 resulted in an increase of IL-10 and limited antigen-specific T cell responses.^24^ Loss of HDAC5 impaired the suppressive function of Tregs and reduced the ability of CD8^+^ T cells to produce IFN-γ.^25^ Moreover, pan-HDACis have been reported to exhibit immunosuppressive effects, impairing the innate immune response, inhibiting the proliferation of CD4^+^ and CD8^+^ T cells, promoting the generation and function of Tregs, *etc*.^26–28^ Thence, the precision in developing highly efficient and selective class I and IIb HDACi could yield greater clinical benefits for patients with MM and lymphoma.

Different from the selective HDACis romidepsin and chidamide, purinostat mesylate (PM), a hydroxamic acid HDACi we previously developed, possesses a non-linear, large triangular cap and a rigid linker that significantly enhances its binding affinity to I and IIb HDACs, and its inhibition activity and selectivity on I and IIb are stronger than all other approved HDACis.^29,30^ Herein, we reported the efficacy, safety, and mechanism of PM on MM and lymphoma in preclinical and phase I clinical trial. Preclinical studies have shown that PM has stronger antitumor activity than pan-HDACi panobinostat and several first-/second-line multi-drug combinations. The results of this phase I clinical trial (NCT05526313) demonstrated the overall safety and favorable efficacy of PM monotherapy in the r/r MM and r/r lymphoma patients. Mechanistically, we sought to determine the effects of PM on improving the tumor immune microenvironment. Our findings demonstrate that PM significantly inhibits multiple essential proteins and signaling pathways critical for tumor survival and potentially enhances antitumor immune responses.

## Results

### PM exhibits more potent activity against MM and lymphoma than pan-HDACi or several multi-drug combinations

**Fig. 1:**
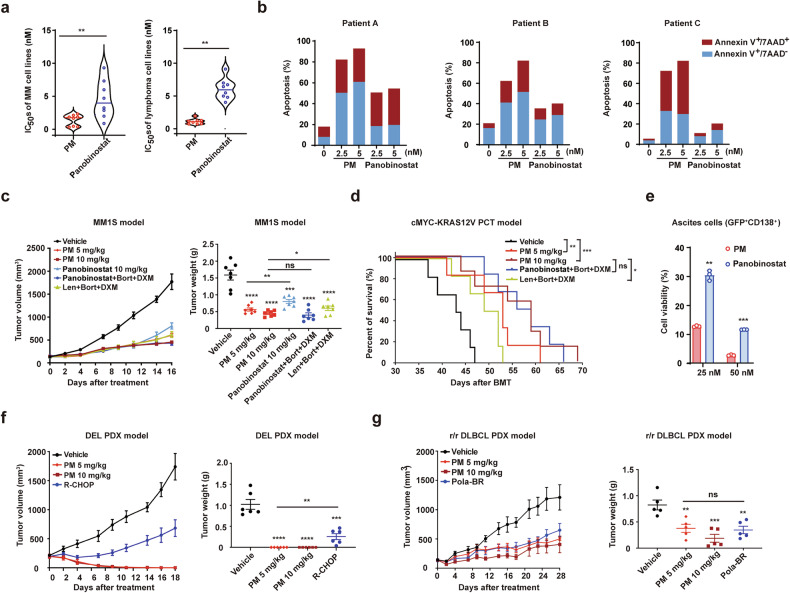
PM exhibits potent activity against MM and lymphoma cells and mouse models. **a** Human MM cell lines (MM1S, MM1R, RPMI-8226, RPMI-8226R, AMO1, OPM2, ARD, and KMS-11) and lymphoma cell lines (RL, Ramos, Daudi, Raji, TMD-8, SU-DHL-4, Karpas299, and NKYS) were treated with PM or panobinostat at indicated concentrations for 72 h, and cell viability were analyzed. **b** Primary MM and lymphoma cells from PB of MM patients (patient A and B) and lymphoma patient (patient C) were treated with PM or panobinostat at indicated concentrations, and cell apoptosis was analyzed after 48 h. **c** Tumor volumes and tumor weights of MM1S xenograft models treated with vehicle, PM, panobinostat, panobinostat+Bort+DXM or Len+Bort+DXM (n = 7 per group). **d** Kaplan-Meier survival curves for cMYC-KRAS12V PCT mice treated with vehicle, PM, panobinostat+Bort+DXM, or Len+Bort+DXM (n = 6 per group) from day 35 post-transplantation. **e** Cell viability of ascites cells from cMYC-KRAS12V mice after treated with PM or panobinostat in vitro (n = 3 per group). **f**, **g** Tumor volumes and tumor weights of DEL and r/r DLBCL PDX mouse models treated with vehicle, PM, R-CHOP, or Pola-BR (n = 6 per group in DEL PDX, n = 5 per group in r/r DLBCL PDX). All data are represented as mean ± SEM (standard error of the mean). ns P > 0.05, * p < 0.05, ** p < 0.01, *** p < 0.001, **** p < 0.0001, compared with the specified group

Our prior studies reported that PM selectively inhibited HDAC I/IIb (IC_50_ 0.81–11.5 nM) over IIa/IV (436–3349 nM).^29,30^ In comparison, the pan-HDACi panobinostat had IC_50_s of 2.1–277 nM against I and IIb, and 2.7–531 nM against IIa and IV. In addition, PM demonstrated significantly low inhibition for the human ether-à-go-go-related gene (hERG) channel (IC_50_ > 100 μM) compared to panobinostat (IC_50_ = 3.5 μM) (Supplementary Fig. 1a), indicating that PM may possess a more favorable cardiac safety profile. The antiproliferation assays conducted on 8 MM and 8 lymphoma cell lines demonstrated that PM exhibited IC_50_ values all below 5 nM, outperforming panobinostat (Fig. 1a, Supplementary Table 1). In primary MM cells from relapsed patients A and B, and diffuse large B-cell lymphoma (DLBCL) cells from patient C, PM at 5 nM induced 92.9%, 82.0%, and 82.1% apoptosis, respectively, compared to 54.5%, 40.1%, and 20.49% with panobinostat (Fig. 1b, Supplementary Table 2).

Furthermore, the MM1S xenograft mouse model and the cMYC-KRAS12V primary plasmacytoma (PCT) mouse model were used to evaluate the preclinical in vivo efficacy of PM in the treatment of MM. In the MM1S xenograft model, 5 mg/kg PM achieved a tumor inhibition rate of 64.39%, surpassing the 49.37% with 10 mg/kg panobinostat. Notably, PM at 10 mg/kg monotherapy showed superior activity compared to the first-line combined treatment with Len, Bort, and DXM (71.76% vs. 62.05%) and demonstrated comparable efficacy to the combination of panobinostat, Bort, and DXM (Fig. 1c). cMYC abnormal activation and KRAS mutation play a critical role in MM pathogenesis.^31–33^ We observed that PM demonstrated approximately a 4-fold superior antiproliferation activity compared to panobinostat on c-MYC/KRAS12V transformed BaF3 cells (Supplementary Fig. 1b). In c-MYC/KRAS12V induced PCT mouse model, PM at 5 mg/kg significantly prolonged the median survival time (MST) of recipient mice compared to the vehicle (68 days vs. 47 days, P = 0.0278) (Supplementary Fig. 1c). In a more severe PCT model, PM at 10 mg/kg achieved an MST of 59 days, significantly outperforming Len+Bort+DXM (50.5 days, P = 0.0288) and slightly exceeding panobinostat+Bort+DXM (57.5 days) (Fig. 1d). We further tested the inhibitory activity of PM on ascites cells from PCT mice, and the results showed that it was more active than panobinostat (Fig. 1e).

Moreover, we generated two patient-derived xenograft (PDX) mouse models to evaluate the preclinical in vivo efficacy of PM in lymphoma. The first double-expressing lymphoma (DEL) PDX model was derived from a DEL patient. When tumor volume reached ~200 mm³, mice were randomized and treatment was initiated. After 18 days of PM monotherapy (at doses of 5 mg/kg or 10 mg/kg), all mice achieved complete remission, while no mice in the R-CHOP group achieved complete remission, with a tumor inhibition rate of 76.9% (Fig. 1f, Supplementary Table 2). The second PDX model was derived from an r/r DLBCL patient who relapsed after six cycles of R-CHOPE treatment. PM monotherapy at 5 mg/kg significantly inhibited tumor growth compared to the vehicle group (P = 0.009), with efficacy comparable to polatuzumab vedotin plus BR (Pola-BR), a standard treatment for r/r DLBCL. At 10 mg/kg, PM achieved a tumor inhibition rate of 77.14%, surpassing the 57.91% observed with Pola-BR (Fig. 1g, Supplementary Table 2). Additionally, no weight loss or other evident side effects were observed in the PM treatment groups in the above mouse models, whereas significant weight loss occurred in the panobinostat+Bort+DXM and Pola-BR groups (Supplementary Fig. 1d). Overall, PM monotherapy shows outstanding activity in MM and lymphoma cells and mouse models.

### PM alters multiple essential factors for MM and lymphoma survival and triggers immune-related inflammation and interferon responses

**Fig. 2:**
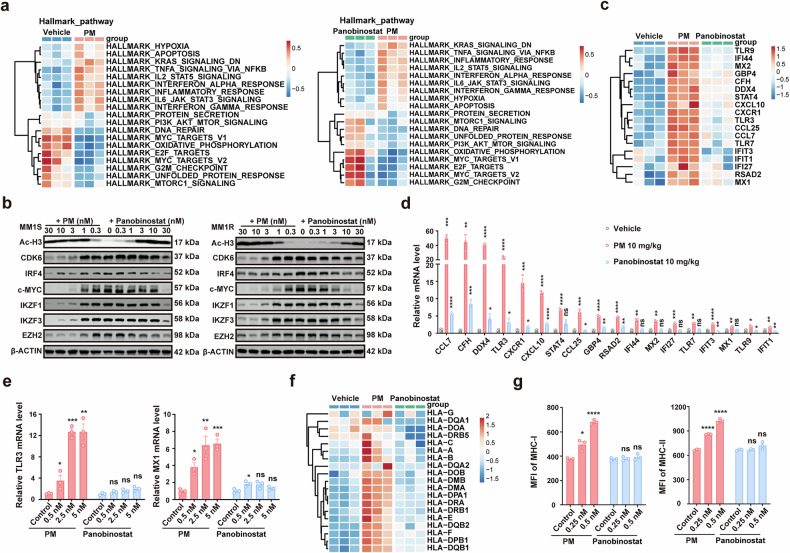
PM alters multiple gene expressions in MM. **a** Hallmark gene sets from bulk RNA-seq of MM1S xenograft mice treated with vehicle, PM or panobinostat (10 mg/kg, n = 3) for 24 h. **b** The protein levels change in MM1S and MM1R cells treated with PM or panobinostat for 24 h. β-ACTIN served as the loading control. **c** Expression levels of 18 selected genes related to inflammatory cytokines and chemokines from RNA-seq of MM1S tumor tissues. **d** RT-PCR validated selected gene mRNA levels from MM1S tumor tissues. **e** RT-PCR determination of TLR3 and MX1 mRNA expression in MM1S cells treated with control, PM, or panobinostat at the indicated concentrations for 24 h. **f** Heat-map of gene expression change of MHC from RNA-seq of MM1S tumor tissues. **g** Flow cytometry analysis of MHCI (HLA-A, B, C) and MHCII (HLA-E) expression in MM1S cells treated with control, PM, or panobinostat for 24 h. All data are represented as mean ± SEM. * p < 0.05, ** p < 0.01, *** p < 0.001, **** p < 0.0001, compared with vehicle or control

To investigate the mechanisms of PM treatment and identify key factors for its superior activity over panobinostat, we conducted bulk RNA-seq analysis on MM1S cells treated with vehicle (control), PM and panobinostat, both in vitro and in vivo, as well as the DEL PDX model treated with vehicle and PM. Consistent with its therapeutic effect, gene set enrichment analysis (GSEA) results showed that the expression of genes was significantly decreased and enriched in the gene sets including “MYC_TARGETs”, “DNA_REPAIR”, “OXIDATIVE_PHOSPHORYLATION”, “E2F_TARGETs”, “G2M_CHECKPOINT”, “UNFOLDED_PROTEIN_RESPONSE”, and “mTORC1_SIGNALING in PM treated samples compared to vehicle (control), or panobinostat treated samples (Fig. 2a, Supplementary Fig. 2a-d). Multiple factors, such as EZH2, c-MYC, IKZF1, IKZF3, IRF4, and CDK6, which are essential for the proliferation and survival of malignant plasma cells and lymphoma,^34,35^ were decreased upon treatment with PM in all tested MM and lymphoma cell lines, including MM1S, MM1R, and TMD-8 (Fig. 2b, Supplementary Fig. 2e).

In addition, bulk RNA-seq analysis of MM1S tumor tissues identified differential modulation of immune-related pathways between PM and panobinostat treatments. PM induced significant enrichment and upregulation of “INTERFERON_ALPHA_RESPONSE”, “INTERFERON_GAMMA_RESPONSE”, “TNFA_SIGNALING_VIA_NFKB”, “IL2_STAT5_SIGNALING”, and “IL6_JAK_STAT5_SIGNALING”, which were not observed in the panobinostat group (Fig. 2a, Supplementary Fig. 2a-c). Bulk RNA-seq of DEL PDX mouse models also revealed that PM treatment significantly upregulated these signaling pathways (Supplementary Fig. 2d). Likewise, interferon response genes (including *TLR3*, *MX1*, *RSAD2*, *IFI44*, *IFI27*, *IFIT1*, and *IFIT3*), and chemokine genes (including *CCL7*, *CXCL10*, *CXCR1*, and *CCL25*) were significantly increased in PM-treated MM tumor tissues, whereas panobinostat showed minimal effects at equivalent doses (Fig. 2c). These results were further confirmed by RT-PCR (Fig. 2d). Moreover, we observed dose-dependent increases in TLR3 and MX1 expression in MM1S cells treated with PM, but no significant increase in the panobinostat group (Fig. 2e). PM treatment also notably increased MHC-related gene expression in both MM1S and DEL PDX model tumor tissues (Fig. 2f, Supplementary Fig. 2f). Flow cytometry analysis confirmed concentration-dependent upregulation of MHC-I and MHC-II in MM1S cells following PM treatment, but not after panobinostat treatment (Fig. 2g). Collectively, these results indicate that PM significantly suppresses the expression of multiple genes that are critical for survival in MM and lymphoma, and activates immune response-related signaling, while pan-HDACi panobinostat has no obvious effect on them.

### PM enhances its antitumor effects by improving the immune microenvironment in 5TMM mouse model

**Fig. 3:**
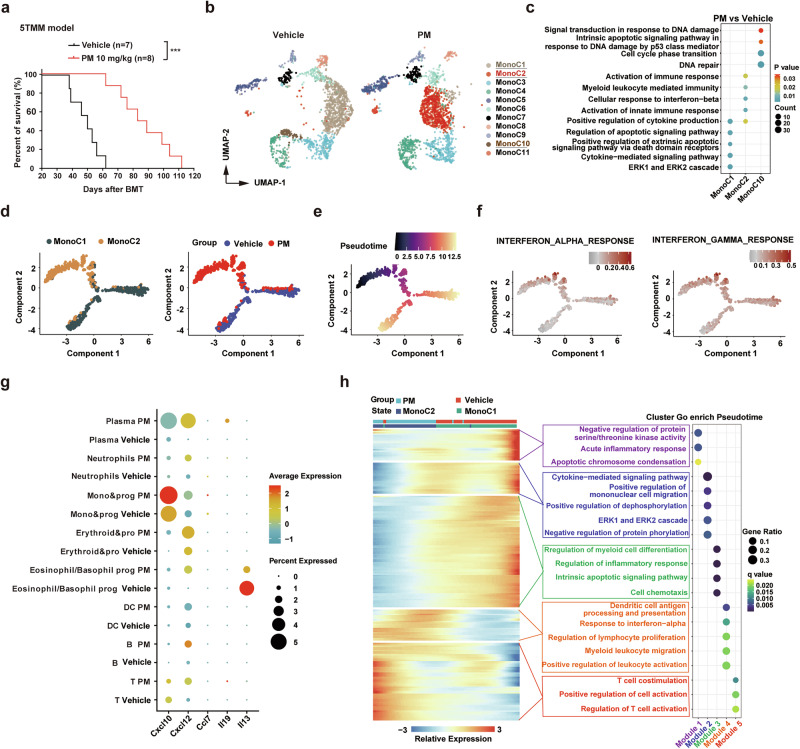
The scRNA-seq analysis of monocyte populations and gene expression changes in 5TMM tumor-burdened mice post PM treatment. **a** Kaplan-Meier survival curves for 5TMM model mice treated with vehicle or PM. **b** UMAP visualizes the distribution of monocytes in BM of 5TMM model mice treated with vehicle or PM. **c** Dot plots illustrate GO enrichment analysis results for MonoC1, MonoC2, and MonoC10 cell clusters. “Count” signifies gene numbers, with the color scale indicating P values. **d**, **e** Pseudo-time order analysis on MonoC1 and MonoC2 cells in the 5TMM model mice treated with vehicle or PM. **f**, **g** “INTERFERON_ALPHA_RESPONSE” and “INTERFERON_GAMMA_RESPONSE” gene sets in vehicle and PM treated samples from two independent experiments were scored along pseudo-time order. **g** Dots were plotted to visualize the expression levels and frequencies of inflammatory factors Il-13 and Il-19, and chemokines Cxcl10, Cxcl12, and Ccl7 in indicated cell type in vehicle or PM groups. Dot sizes represent expression frequencies, and the color scale indicates expression levels. **h** Heat-map displays the dynamic gene expression changes along the pseudo-time order (cataloged hierarchically into five gene modules) of genes relative to GO enrichments. q value < 0.05 is considered statistically significant for GO enrichment analysis

5TMM mouse model can mimic the pathological features of osteolytic lesions in clinical MM patients.^36^ We observed that PM had stronger antiproliferative activity than panobinostat and induced more cellular apoptosis in 5TGM cells (Supplementary Fig. 3a, b). In vivo, PM at 10 mg/kg significantly extended disease mice survival compared to the vehicle (86 days vs. 50 days, P = 0.0001) (Fig. 3a). Additionally, PM treatment for two weeks also reduced serum calcium levels compared with the vehicle group (8.972 vs. 20.93 mmol/L, P = 0.0027) (Supplementary Fig. 3c).

To comprehensively investigate the impact of PM on the immune system and microenvironment, BM cells from the vehicle or PM-treated 5TMM model mice were analyzed using scRNA-seq. After stringent filtration, 16,660 cells from the vehicle and 22,219 cells from the PM-treated group were retained for analysis. UMAP divided the cell clusters into 11 types, including B, T, DC, plasma, CAR, eosinophil/basophil prog, erythroid&prog, HSPC, macrophage, neutrophils, and mono&prog cells (Supplementary Fig. 4a-c), and the proportions are shown in Supplementary Fig. 4d. Plasma cells were further divided into 5 clusters (Supplementary Fig. 5a-c). To distinguish malignant plasma cells from normal cells, we calculated the large-scale chromosomal copy number variation (CNV). The CNV scores for clusters 0-4 were all greater than 1,000, and all plasma cells revealed extensive CNVs, indicating they are all neoplastic plasma cells (Supplementary Fig. 4e, f and Supplementary Fig. 5d). Notably, PM significantly altered the proportion of all plasma cell clusters, especially cluster 4 (Supplementary Fig. 5e). Gene set scores analysis revealed that the “CELL_CYCLE”, “STEMNESS” and “OXIDATIVE_PHOSPHORYLATION” gene sets in clusters 0 and 4 had higher scores, indicating that these cells were in a more active proliferative state (Supplementary Fig. 5f-h). Genes in the “APOPTOSIS” gene set were highly expressed and enriched in cluster 4, specifically in the PM treated samples (Supplementary Fig. 5i). Moreover, consistent with the bulk RNA-seq results, GSEA also revealed that PM significantly decreased the gene expression in gene sets including “MYC_TARGETs”, “OXIDATIVE_PHOSPHORYLATION”, “E2F_TARGETs”, “G2M_CHECKPOINT” and “UNFOLDED_PROTEIN_RESPONSE” (Supplementary Fig. 5j). These results explain why PM effectively suppressed malignant plasma cells in the 5TMM mouse model.

We then analyzed the monocytes in scRNA-seq and observed distinct differences in the distribution of the three monocyte clusters between the vehicle and PM groups of the 5TMM mouse model. Monocytes in the vehicle group were majorly represented in MonoC1 and MonoC10, while those in the PM group were mainly represented in the MonoC2 cluster (Fig. 3b). Gene Ontology (GO) analysis results of DEGs in the MonoC2 cluster revealed that PM treatment affected the innate immune response and cytokine production signaling pathways, while in MonoC1 and MonoC10 it mainly affected the apoptosis and DNA damage signaling pathways (Fig. 3c). UMAP clustering and pseudo-time order analysis indicated that MonoC1 and MonoC2 likely represent adjacent monocyte states (Fig. 3b, d, e). Compared to MonoC1, DEGs in MonoC2 cluster exhibited a significant up-enrichment in the “INTERFERON_ALPHA_RESPONSE” and “INTERFERON_GAMMA_RESPONSE” gene sets (Fig. 3f), indicating PM treatment may stimulate interferon response. Additionally, PM increased the mRNA levels of *Cxcl10*, *Cxcl12* and *Ccl7* in multiple immune cells and plasma cells (Fig. 3g). Based on the transcriptional changes associated with transitional states of MonoC1 and MonoC2, five distinct GO modules (Modules 1–5) were identified. Genes in the MonoC1 cluster, which involved negative regulation of protein serine/threonine kinase activity, positive regulation of de-phosphorylation, apoptotic signaling pathway, and regulation of myeloid cell differentiation, were upregulated in a pseudo-time order (see Supplementary Methods). In contrast, genes in the MonoC2 cluster, which were related to immune cell proliferation and activation, antigen processing and presentation, interferon response, and regulatory T cell activation, were upregulated in a pseudo-time order (Fig. 3h). Together, these results suggest that PM treatment may activate the innate immune response in the BM microenvironment of 5TMM model mice.

**Fig. 4:**
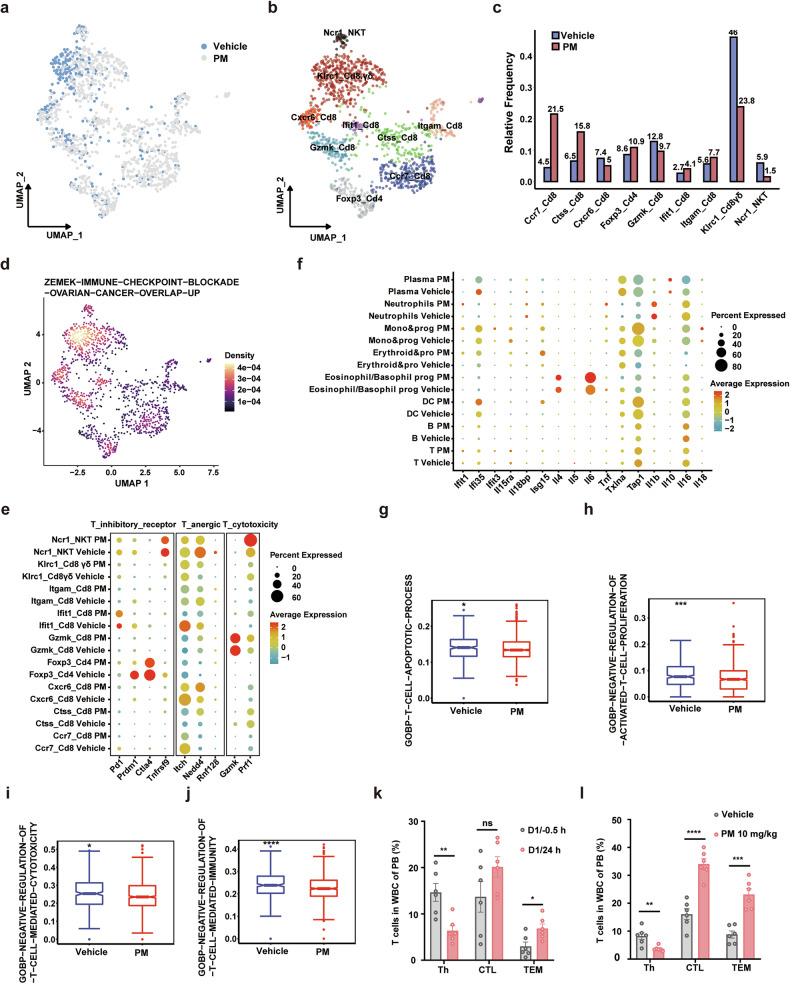
T cell characteristics in PM treated 5TMM model mice. **a** UMAP plots T cell subtypes distribution in BM of 5TMM model mice with vehicle or PM treatment. **b** T cell subpopulations are clustered and color-coded. **c** Frequencies of major T cell clusters. **d** UMAP plots show gene expression related to the “ZEMK-IMMUNE-CHECKPOINT-BLOCKADE-OVARIAN-CANCER-OVERLAP-UP” gene set in T cell clusters. Dot plots expression levels and frequencies of immune checkpoint receptors, inactive T cell genes, and T cell cytotoxicity related genes (**e**), and labeled cytokines (**f**) in indicated cell types in vehicle or PM groups. Dot sizes indicate expression frequencies and color scale indicates expression level. **g**–**j** Box plots depict gene expression intensities relative to the specific biological processes in the BM of vehicle or PM treated 5TMM mice. The percentages of T cell subtypes, including Th (CD3^+^CD4^+^), CTL (CD3^+^CD8^+^), and TEM (CD3^+^CD8^+^CD44^high^CD62L^−^), in PB of 5TMM model mice post PM treatment, **k** before administration (D1/−0.5 h) and PM 10 mg/kg single dose administration for 24 h (D1/24 h), **l** model mice treated with vehicle or PM 10 mg/kg for 1 month. *p < 0.05, ***p < 0.001, and ****p < 0.0001 (two-sided Wilcoxon rank-sum tests)

Cell type ratio analysis results revealed that PM treatment increased the percentage of T cells in the BM of the 5TMM mouse model (Supplementary Fig. 4d). To further elucidate the effects of PM on the adaptive immune response in MM treatment, we conducted a detailed analysis of the differences in T cell subsets of the 5TMM model. UMAP further divided T cells into 9 clusters based on the expression of their characteristic molecular markers (Fig. 4a, b and Supplementary Fig. 6c). The statistical results of T cell subsets suggested that PM treatment significantly increased the proportion of Ccr7^+^CD8^+^ cytotoxic T cells, while Klrc1^+^ CD8^+^ γδT cells obviously reduced (Fig. 4c). Klrc1^+^ CD8^+^ γδT cells are considered ineffective in antitumor immune responses,^37^ which shown a higher enrichment density of the immune checkpoint blockade gene set “ZEMEK-IMMUNE-CHECKPOINT-BLOCKADE-OVARIAN-CANCER-OVERLAP-UP” (Fig. 4d). To further elucidate whether these subsets ratio changes reflect functional differences, cell communication analysis was performed. The results indicated that PM treatment not only increases the ligand-receptor interactions between plasma cells and T cells, but also the ligand-receptor interactions between T cells and other immune cells (Supplementary Fig. 6a, b). We further examined the changes in the expression of T cell immune checkpoint receptors, including Pd1, Prdm1, Ctla4, and Tnfrsf9, and found that PM significantly decreased their mRNA levels compared to vehicle. Additionally, PM also increased Gzmk and Prf1 expression of the cytotoxicity T cell markers and decreased the expression of anergic T cell markers, such as Itch, Nedd4 and Rnf128 (Fig. 4e). Moreover, PM increased the expressions of Tnf in neutrophils, Ifi35 and Tap1 in mono&prog and DC cells, Il-6 in eosinophil/basophil prog cells, and Isg15 in various cells (Fig. 4f). Overall, T cell signaling pathways analysis revealed that PM treatment reduced T cell apoptosis, promoted the proliferation and activity of cytotoxicity T cells, enhanced T cell-mediated immune responses (Fig. 4g–j). Finally, we analyzed the changes of T cells in 5TMM model mice after PM treatment via flow cytometry. As shown in Fig. 4k, l, a single PM treatment significantly increased the ratio of effector memory T cells (CD3^+^CD8^+^CD44^high^CD62L^-^, TEM) in the peripheral blood (PB) of disease mice, and after one month of continuous treatment, the proportion of CD3^+^CD8^+^ T cells and TEM cells in the PB were significantly increased. Collectively, these results demonstrate that PM not only directly prevents tumor progression by targeting tumor cells but also activates the innate and adaptive immune responses.

### PM blocks the formation of osteoclasts and inhibits osteolysis

**Fig. 5:**
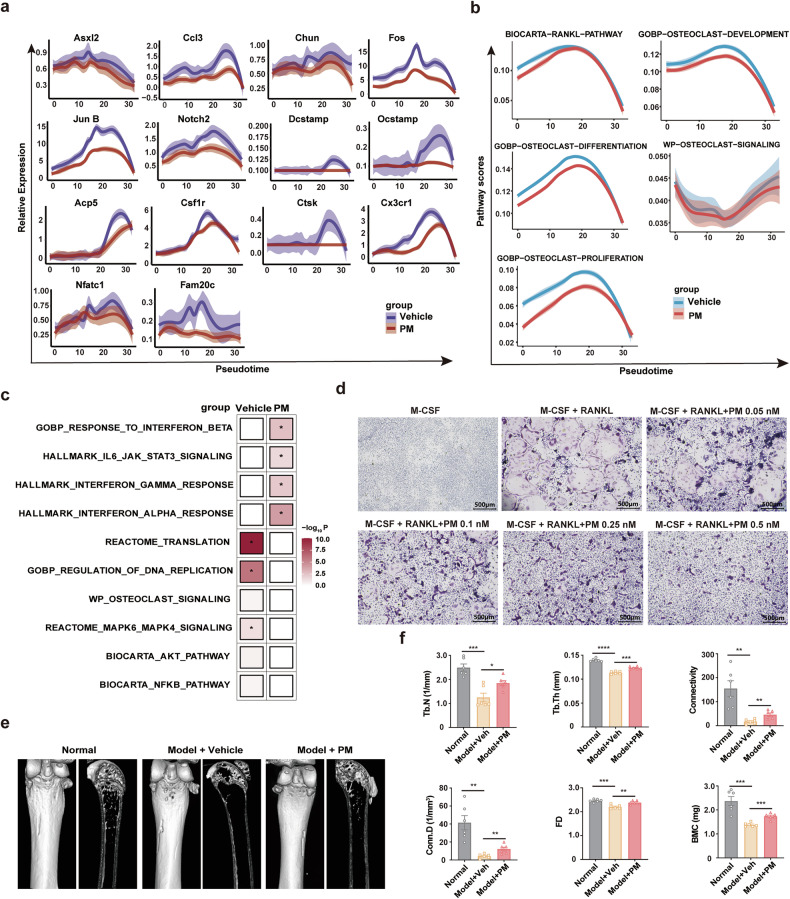
PM inhibits osteolysis in 5TMM mouse model. **a** Two-dimensional plots depict the dynamic gene expression in monocytes along pseudo-time order from vehicle or PM treated 5TMM model mice. Genes with a log-transformed fold change values greater than 0.25, the minimum percentage >0.25, and adjusted p-value < 0.05 were deemed significantly upregulated or downregulated. **b** Two-dimensional plots show the dynamic expression of scores of gene relative to “BIOCARAT-RANKL-PATHWAY”, “GOBP-OSTEOCLAST-DEVELOPMENT”, “GOBP-OSTEOCLAST-DIFFETENTISTION”, “WP-OSTEOCLAST-SIGNALING”, and “GOBP-OSTEOCLAST-PROLIFERATION” genes sets along with pseudo-time order in monocyte/macrophage in the vehicle or PM treated samples. y axis represents GSVA scores. Pathways are selected from the GSEA results (NES > 1, NOM p value < 0.05, and FDR q value < 0.25). **c** GO and GSEA results for DEGs of PM treated macrophages compared to vehicle treated. **d** TRAP staining shows osteoclast formation exposed to PM at indicated concentrations. **e**, **f** Micro-CT images were used to assess the bone structure of the femur, and bone parameters were measured in normal C57BL/KawRij mice and 5TMM model mice treated with vehicle or PM 10 mg/kg for 4 weeks (n = 6). All data are represented as mean ± SEM. *p < 0.05, **p < 0.01, ***p < 0.001, ****p < 0.0001, t test

The serum calcium concentration of diseased mice decreased (Supplementary Fig. 3c), indicating that PM treatment may improve MM osteolytic symptoms. To further investigate whether PM treatment could prevent osteolysis in the 5TMM mouse model, pseudo-time order analysis with DDRTree algorithm was performed to study the dynamic expression changes of multiple genes in monocytes that are critical for osteoclast differentiation and function activation. As shown in Fig. 5a, PM repressed the expression of these genes at most stages of osteoclast differentiation or function activation, including Csf1r, Cx3cr1, Fos, JunB, Acp5, and Nfatc1, which are involved in the transition from monocyte to pre-osteoclast; Asxl2, Chun, Dsctamp, and Ocstamp (e.g.,), which are involved in differentiation from pro-osteoclast to mature osteoclast; Ccl3, Notch2, Fam20, and Ctsk, which are essential for osteoclast function activation. GSEA results along pseudo-time order also revealed that PM decreased the expression of genes related to osteoclast development, differentiation and proliferation, specifically in RANKL pathway (Fig. 5b). Moreover, GSEA results in macrophage populations showed that PM increased the expression of genes related to the inflammation and interferon response gene sets and inhibited key signaling pathways such as Nf-kB and Mapk for macrophage differentiation into osteoclasts (Fig. 5c). Thus, these data suggest that PM may block the formation of osteoclasts and inhibit osteolysis by disrupting molecular program of osteoclastogenesis.

To verify if real-world data aligns with bioinformatics analysis, we investigated the effect of PM on osteoclastogenesis in vitro and in vivo. Monocytes were induced to mature into osteoclast cells with M-CSF and RANKL. As expected, TRAP staining showed that PM obviously suppressed mature osteoclast cells formation (Fig. 5d). Bone structure analysis with Micro-CT imaging on 5TMM model mice showed that PM effectively prevented bone destruction and trabecular changes on the femoral surface compared to vehicle (Fig. 5e). Further statistics revealed that PM treatment improved multiple trabecular bone parameters, including trabecular number (Th.N), trabecular thickness (Tb.Th), number of connections between trabecular network structures (Connectivity), number of connections between the trabecular network of bone per cubic millimeter volume (Conn.D), analytical dimension (FD) and mineral mass in bone tissue between the trabecular network in the volume of cubic millimeters (BMC) (Fig. 5f). Together, these results demonstrate that PM treatment can improve osteolytic lesions in MM.

### PM exhibits favorable tolerability and promising efficacy in phase I trials for r/r MM and lymphoma

**Fig. 6:**
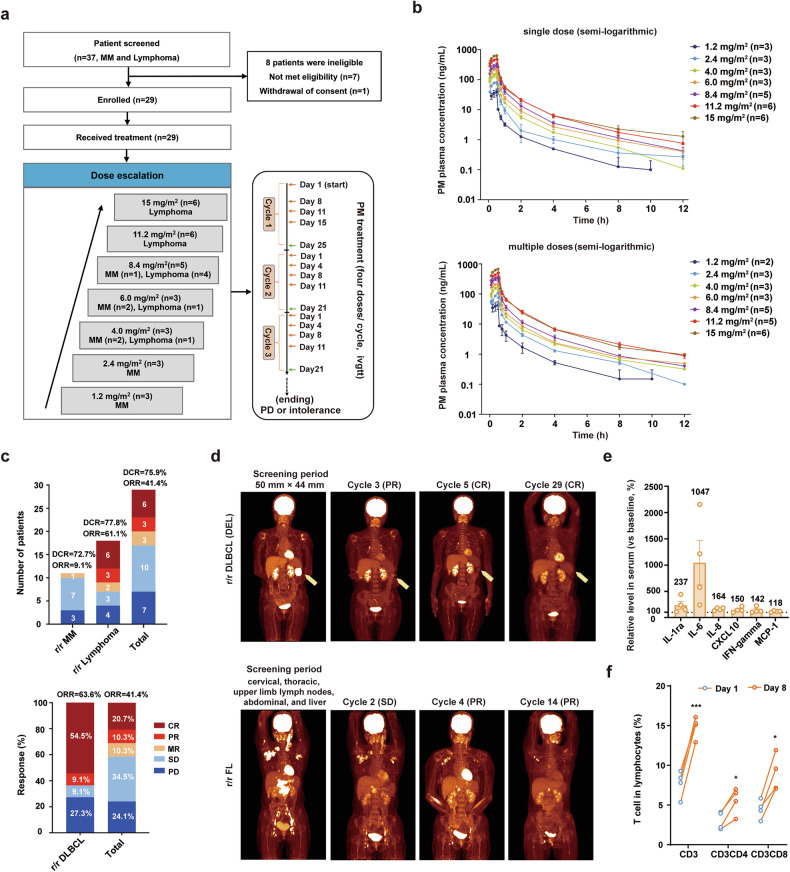
PM in phase I clinical trials for treating r/r MM and r/r lymphoma patients. **a** Schematic diagram of PM therapy administration for r/r MM and lymphoma patients. **b** Average plasma concentration-time semi-logarithmic curves of PM in clinical phase I patients. After a single intravenous infusion (top) or at specified times before and after the last dose of the multiple-dose phase (Day 15, bottom), UPLC-MS/MS was employed to detect the concentration of PM in plasma. All data are represented as mean ± SD. **c** Phase I clinical trial efficacy responses of PM in patients with r/r MM, r/r lymphoma, and r/r DLBCL. **d** PET-CT images of a patient with r/r DEL DLBCL (No.26, 15 mg/m^2^) and r/r FL (No.18, 11.2 mg/m^2^) at the screening and different stages after PM treatment. **e** Serum cytokine levels in r/r MM (n = 2) and r/r lymphoma (n = 2) patients before and after one week of PM administration. **f** Proportion of T cells in PB leukocytes in r/r MM (n = 2) and r/r lymphoma (n = 2) patients before and after 1 week of PM administration. Data are represented as mean ± SEM. *p < 0.05, ***p < 0.001, t test

From August, 2020, to August, 2022, a total of 29 patients, including 11 r/r MM and 18 r/r lymphoma, were enrolled and received dose escalation of PM at two centers (Fig. 6a). The baseline characteristics are summarized in Table 1. The median age of the patients was 53 years (range: 22–72). Most patients presented relatively high-risk disease: 91% (10/11) r/r MM patients with Durie-Salmon Staging System stage III, and 94% (17/18) r/r lymphoma patients with Lugano stage III or above. All r/r MM patients had previously been treated with Len, Bort, and DXM, and a median of three prior lines of treatment (range 2–7), including autologous stem cell transplantation, daratumumab, BCMA-CAR-T, etc. (Table 1, Supplementary Table 3). The r/r lymphoma patients had a median of two prior lines of treatment (range 1–4), and 22.2% (4/18) of patients had been previously exposed to the selective HDACi chidamide (Table 1, Supplementary Table 4).

**Table 1:** Safety population patient characteristics: dose escalation

Baseline Demographics & Disposition	N = 29
**sex, n (%)**	
Male	18 (62)
Female	11 (38)
**Age, median years (range)**	53 (22–72)
**Histology, n (%)**	
MM	11 (37.9)
Lymphoma	18 (62)
Subtype, n (%)	
DLBCL	11 (37.9)
FL	3 (10.3)
Large B-cell lymphoma	1 (3.4)
cHL	1 (3.4)
PTCL-NOS	1 (3.4)
AITL	1 (3.4)
**Stage of MM, n (%)**	
II	1 (9.1)
III	10 (90.9)
**Stage of DLBCL, n (%)**	
I–II	1 (9.1)
III–IV	10 (90.9)
**No. prior regimens, median (range)**	
MM	3 (1–7)
Lymphoma	2 (1–4)
**Prior therapy of MM, n (%)**	
Lenalidomide, Bortezomib, Dexamethasone	11 (100)
Daratumumab	3 (27.3)
Autologous Stem Cell Transplantation	2 (18.2)
BCMA-CAR-T	1 (9.1)
**Prior therapy of DLBCL, n (%)**	
Chidamide+RCHOP	2 (18.2)
Autologous stem cell transplantation	2 (18.2)

*MM* multiple myeloma, *DLBCL* diffuse large B-cell lymphoma, *FL* follicular lymphoma, *cHL* classic hodgkin lymphoma, peripheral T cell lymphoma-not otherwise specified, *AITL* angioimmunoblastic T-cell lymphoma. *RCHOP* Rituximab, Cyclophosphamide, Doxorubicin, Vincristine, Prednisone

Except for one patient (No.3) who received 1.2 mg/m^2^ intravenous infusion in multiple doses and did not undergo pharmacokinetics (PK) testing, all other 28 enrolled patients had plasma PK testing after both single and multiple doses of PM administration. The peak concentration (C_max_) of PM in the plasma is reached at 0.4–0.5 h after a single (Day 1) intravenous injection, exhibiting a half-life (t_1/2_) of ~2.30–3.51 h (Supplementary Table 5, Fig. 6b). The C_max_ and AUC_0-t_ demonstrated dose proportionality within the range of 1.2–8.4 mg/m^2^, with a slight increase beyond dose-proportional levels observed from 11.2 to 15.0 mg/m^2^. Similar results were observed after multiple dose administration (Day 15). The accumulation index in C_max_ and AUC_0–∞_ between multiple-dose and single-dose administration is especially crucial when assessing drug accumulation. As shown in Supplementary Table 5, across all ramping doses from 1.2 to 15.0 mg/m^2^, the accumulation indexes for C_max_ and AUC_0–∞_ (multiple doses/single dose) were close to 1, ranging from 0.96 to 1.32 and 0.97–1.17, respectively. These results demonstrate that PM does not accumulate in plasma after multiple dose administration, suggesting minimal risk of accumulation-related toxicity.

As of the data cutoff on July 7, 2023, the treatment-related adverse events across all dose levels are shown in Supplementary Table 6. Among the 29 treated patients, 25 (86.2%) experienced grade 3–4 treatment-emergent adverse events (TEAEs), with no grade 5 events reported (Supplementary Table 7). The most common Grade ≥3 adverse events were hematologic disorders, including thrombocytopenia (n = 22, 75.9%), neutropenia (n = 16, 55.2%), leukopenia (n = 12, 41.4%) and lymphopenia (n = 9, 31.0%) (Table 2). These adverse events mainly occurred during the extended dosing phase, which is consistent with the common adverse reactions disclosed for similar HDAC inhibitors and could be reversed through standard intervention measures. Furthermore, the cardiac toxic adverse events, which are of concern for similar HDAC inhibitors, were of low incidence and mild severity in this trial, with no grade 3 or higher events occurring. Together, PM was well tolerated in the dose range of 1.2–15 mg/m^2^, and no dose-limiting toxicity (DLT) events occurred.

**Table 2:** Patients experience any grade ≥ 3 adverse events during treatment (n = 29)

SOC PT	1.2 mg/m^2^	2.4 mg/m^2^	4.0 mg/m^2^	6.0 mg/m^2^	8.4 mg/m^2^	11.2 mg/m^2^	15 mg/m^2^	Total
N	3	3	3	3	5	6	6	29
**Hematological toxicities n (%)**								
Neutropenia	1 (33.3)	1 (33.3)	2 (66.7)	1 (33.3)	4 (80.0)	3 (50.0)	4 (66.7)	16 (55.2)
Leukopenia	1 (33.3)	0 (0)	0 (0)	1 (33.3)	5 (100.0)	3 (50.0)	2 (33.3)	12 (41.4)
Lymphopenia	0 (0)	0 (0)	1 (33.3)	1 (33.3)	2 (40.0)	2 (33.3)	3 (50.0)	9 (31.0)
Thrombocytopenia	3 (100.0)	1 (33.3)	3 (100.0)	2 (66.7)	5 (100.0)	4 (66.7)	4 (66.7)	22 (75.9)
Anemia	1 (33.3)	1 (33.3)	0 (0)	1 (33.3)	1 (20.0)	1 (16.7)	0 (0)	5 (17.2)
**Non-hematological toxicities n (%)**								
Hypokalemia	1 (33.3)	0 (0)	0 (0)	1 (33.3)	1 (20.0)	0 (0)	0 (0)	3 (10.3)
Hyperkalemia	0 (0)	0 (0)	0 (0)	0 (0)	0 (0)	1 (16.7)	0 (0)	1 (3.4)
Infectious pneumonia	0 (0)	0 (0)	0 (0)	0 (0)	0 (0)	2 (33.3)	0 (0)	2 (6.9)
Urinary tract infection	0 (0)	0 (0)	0 (0)	0 (0)	1 (20.0)	0 (0)	0 (0)	1 (3.4)
Upper respiratory tract infection	0 (0)	0 (0)	0 (0)	0 (0)	2 (40.0)	0 (0)	0 (0)	2 (6.9)
Oral mucositis	0 (0)	0 (0)	0 (0)	1 (33.3)	0 (0)	0 (0)	0 (0)	1 (3.4)

For efficacy evaluation, 11 patients with r/r MM treated with escalating doses of 1.2–8.4 mg/m^2^, the disease control rate (DCR) was 72.7% (Fig. 6a, c, Table 1 and Supplementary Table 8). Patient No.10, who relapsed after previous two-line therapy, received PM at 6.0 mg/m², the M-protein dropped from 26.290 g/L to 15.998 g/L after 2 cycles of treatment, achieving minimal response (MR), and the MR lasted for more than 6 months (Supplementary Table 3). Among the 18 r/r lymphoma patients treated with 4.0–15.0 mg/m^2^, the objective response rate (ORR) was 61.1% (11/18) (Fig. 6c, Supplementary Table 8). Surprisingly, 11 r/r DLBCL patients achieved an ORR 63.6%, with a complete response (CR) rate of 54.5% (6 CRs and 1 partial response (PR)) (Fig. 6c). Notably, a patient with r/r DEL DLBCL and MYD88 mutation, who had previously received two lines of therapy (chidamide combined with R-CHOP, R-ICE), achieved CR with PM treatment, and the CR was sustained for over 18 months (Fig. 6d). Furthermore, three follicular lymphoma (FL) patients classified as stage III or IV, two achieved MR and PR, with an ORR of 66.7% (Supplementary Tables 4 and 8). Among them, one patient with multiple extranodal lesions in the neck, chest, upper limb lymph nodes, abdomen and liver by PET-CT during the screening period, achieved PR after four PM courses, and the PR was maintained for more than 10 cycles (Fig. 6d). A patient with peripheral T-cell lymphoma relapsed after fourth-line therapy and another one with angioimmunoblastic T-cell lymphoma relapsed after second-line therapy, exhibited PR and minor response (MR) following the initial cycle of PM treatment, respectively (Supplementary Table 4). Both of them had been previously exposed to the combination of chidamide with chemotherapy drugs, and initially showed a PR but later discontinued treatment due to PD. Consistent with the results of preclinical study, the contents of IL-1ra, IL-6, IL-8, CXCL10, MCP-1, and IFN-γ in serum of the response MM and lymphoma patients were increased after one week of PM treatment (Fig. 6e). Moreover, the proportions of CD3^+^, CD3^+^CD4^+^, and CD3^+^CD8^+^ T cells in the PB of those patients increased from 8.42%, 4.12%, and 4.30% to 16.05%, 6.49%, and 9.56%, respectively (Fig. 6f). Together, these results demonstrate that PM is safe, moderately effective in r/r MM, and highly effective in r/r lymphoma, especially DLBCL.

### Combination of PM with pomalidomide and DXM shows synergistic anti-myeloma activity in preclinical and clinical studies

CDK6 upregulation mediates MM resistance to immunomodulatory drugs (IMiDs) such as Len and Pom.^34^ PM effectively inhibited CDK6 expression, suggesting that PM combined with IMiDs may benefit MM patients (Fig. 2b). Therefore, we further explored whether PM combined with Pom and DXM has synergistic anti-myeloma activity. Indeed, the antiproliferative activity assay on RPMI-8226, MM1S and MM1R cells revealed that PM effectively enhanced the antitumor effect of the combination of Pom and DXM, with combination indexes as low as 0.14, 0.12 and 0.44 at 72 h, respectively (Supplementary Fig. 7a, b). The cellular apoptosis assay of primary cells from two patients who were resistant to Len, Bort, and DXM showed similar synergistic results (Supplementary Fig. 7c). Western blotting results shown PM alone or combined with Pom and DXM down-regulated CDK6 expression while Pom plus DXM up-regulated or had no effect on CDK6. Moreover, the combination of PM and Pom and DXM also synergistically down-regulated c-MYC, IKZF1, IKZF3, and EZH2 expression (Supplementary Fig. 7d).

TP53 mutations and copy number loss are associated with disease progression and increased drug resistance in MM patients.^2,38^ We further evaluated the synergistic efficacy of the combination in RPMI-8226 xenograft mouse model (TP53 E285K, function loss). PM monotherapy (5 mg/kg), Dara+Bort+DXM (5 + 0.1 + 1 mg/kg), Pd (2.5 + 1 mg/kg), and PM+Pd (5 + 2.5 + 1 mg/kg) significantly inhibited the tumor growth with inhibition rates of 86.05%, 85.93%, 91.25%, and 96.37%, respectively (Supplementary Fig. 7e, f). Obviously, the PM plus Pd combination achieved the best anti-tumor activity, with 1 (1/6) mouse showing complete tumor regression, outperforming PM alone (P = 0.0002) and Dara+Bort+DXM (P = 0.0017) and Pd (P = 0.0001). Additionally, the PM and Pd combination displayed good animal tolerance, with less than 1% body weight loss during the one-month treatment period (Supplementary Fig. 7g). Immunohistochemistry showed that PM plus Pd reduced the protein expressions of c-MYC, IRF4, and KI67, exceeding the inhibitory effects of PM and Pd alone (Supplementary Fig. 7h). Similarly, western blotting results revealed that the combination treatment significantly inhibited EZH2, c-MYC, IKZF1, IRF4, and CDK6 protein levels (Supplementary Fig. 7i). Importantly, phase Ib/IIa of PM plus Pd treatment is undergoing dose escalation, and the first two r/r MM patients achieved SD and PR in the preliminary evaluation of C1D28, respectively (Supplementary Fig. 7j). Together, our results indicate that PM plus Pd exhibits strong synergistic anti-myeloma activity and support further phase Ib/IIa clinical trials of PM combination with Pd in r/r MM.

## Discussion

Targeting HDAC-related epigenetic modification has been considered a promising strategy for the treatment of hematologic malignancies.^6^ Based on the unique roles of each HDAC isoform, targeted inhibition of I/IIb HDACs is considered a better option for MM and lymphoma patients.^10,11,13^ PM, a novel highly selective class I and IIb HDACi with good safety and tolerability, demonstrates higher inhibitory activity on HDAC I/IIb compared to all approved HDACis (Supplementary Fig. 1a),^30^ and has been approved by the FDA and National Medical Products Administration (NMPA) of China for clinical trials in the treatment of r/r MM and r/r lymphoma. In this study, we systematically evaluated the efficacy, pharmacokinetics, safety, and potential mechanisms of PM in the treatment of MM and lymphoma from preclinical and phase I clinical studies.

In preclinical studies, we showed that the activity of PM was markedly superior to panobinostat or various first-line/second-line standard multi-drug combination therapies, and disrupted several pivotal signaling pathways and biological processes associated with the survival of MM and lymphoma. Particularly, PM has a significantly greater impact on targets and signaling pathways critical to tumor survival than panobinostat, which strongly supports the rationality of selectively targeting I/IIb HDACs in the treatment of MM and lymphoma. Tumor cell-associated inflammatory response triggers antitumor activity of immune cells, which plays an important role in the treatment of various malignancies including MM and lymphoma.^39,40^ Interestingly, our transcriptomic analysis revealed that PM treatment effectively activated multiple interferon-stimulated genes, pro-inflammatory cytokines, and chemokines, with concomitant upregulation of interferon signaling pathways, which were distinct from the effects of panobinostat (Fig. 2, Supplementary Fig. 2). These findings are consistent with the reported differential immunomodulatory effects between selective and pan-HDAC inhibitors.^20^ Toll-like receptors (TLRs) are known to initiate acute inflammatory responses and chemokine production, and further activate antitumor immune responses in vivo.^41^ Importantly, TLR3 activation induces tumor cells to produce type I interferons, which are indispensable for T cell-mediated antitumor immune responses.^40^ We observed a significant increase in TLR3 expression after PM treatment. However, whether TLR3 mediates the antitumor immunity induced by the inflammatory response in myeloma cells remains to be elucidated. In addition, PM treatment also upregulated the expression of multiple MHC-I and MHC-II molecules in tumor cells, which may facilitate the recognition of tumor antigens by immune cells (Fig. 2, Supplementary Fig. 2). Improving the tumor microenvironment is considered to be an effective synergistic treatment for MM and lymphoma.^19,20^ However, several studies have demonstrated that pan-HDACi can significantly impair the phenotype and function of various immune cells.^26,42,43^ Using the 5TMM mouse model with intact immune systems and scRNA-seq technology, we investigated the effects of PM treatment on the immune system in disease states. As key mediators of innate and adaptive immunity, monocytes often secrete proinflammatory cytokines and recruit cytotoxic T/NK cells to exert anti-tumor effects.^41^ We first focused on the effects of PM treatment on monocytes, and found that a distinct MonoC2 population had highly active IFN-α and IFN-γ responses and significantly upregulated the innate immune activation signaling pathways (Fig. 3). Further analysis of effects on T cells revealed that PM not only increases the percentage of total T cells (especially the ratio of CD8^+^ cytotoxic T cells), but also significantly improves the function of T cells. PM treatment suppressed the expression of multiple immune checkpoint receptors, including Pd1, Prdm1, Ctla4, and Tnfrsf9 on NKT, CD8, and CD4 T cells. Also, PM enhanced the secretion of Gzmk and Prf1 in cytotoxicity T cells and inhibited Itch, Nedd4, and Rnf128, which led to the anergic state of T cells (Fig. 4). However, the mechanism by which PM increases the proportion of T cells remains unclear and needs to be elucidated in the future. Cell communication analysis results further demonstrated that PM treatment generally improves the state of the tumor immune microenvironment (Supplementary Fig. 6). Within the bone microenvironment, osteoclastogenesis is driven by RANKL and M-CSF signaling in monocyte/macrophage precursors, which orchestrates the balance of bone remodeling.^44^ The scRNA-seq analysis revealed that PM could effectively disrupt the transcriptional program of monocyte differentiation into osteoclasts in MM disease states. Based on this finding, we further confirmed the effect of PM in improving pathological osteolysis in vitro and in vivo (Fig. 5). Thus, we have reason to believe that PM may be beneficial to improve osteolytic lesions in MM patients.

In this phase I trial, the ORR was low (9.09%) for r/r MM patients, as 9/11 were enrolled in the 1.2–4.0 mg/m² low-dose escalation phase, and one patient at 6.0 mg/m² had received 7 prior lines of therapy. Nevertheless, the effect of PM is still superior or comparable to other HDACis monotherapy in r/r MM, including panobinostat (phase II, 1 PR and 1 MR in 38 patients),^45^ vorinostat (phase I, 1 MR in 10 patients),^46^ and romidepsin (Phase II, 4/12 patients SD),^47^ bisthianostat (phase Ia, 50% patients SD).^48^ Notably, PM monotherapy induced a high ORR of 61.1% for r/r lymphoma. The ORR of r/r DLBCL was 63.6% with 6 CR and 1 PR, which was much higher than those of other HDACis, such as vorinostat (ORR, 6%),^49^ belinostat (ORR, 10.5%, SWOG S0520).^50^ DEL has a poor prognosis, and the 5-year overall survival and progression-free survival rates of R-CHOP treatment are less than 30%.^51^ Excitingly, PM treatment achieved CR in an r/r DEL patient (No.26) for more than 1.5 years. PM exhibits good pharmacokinetic characteristics and a favorable safety window. Only ≤grade 2 transient QTc interval prolongation has been observed, and no arrhythmias or DLT were observed. Patients who had good efficacy were associated with improved immune response (Fig. 4, Fig. 6). Thus, based on this phase I study, we selected PM 8.4 and 11.2 mg/m^2^ doses for monotherapy in a phase II clinical trial for r/r lymphoma.

In MM-resistant cell lines, primary cells from MM patients, and a TP53 mutant mouse model, the combination of PM, Pom, and DXM showed strong synergistic effects, suggesting a good combination therapy strategy for r/r MM in the clinic. Mechanistically, we demonstrated that PM may eliminate MM resistance to IMiDs by inhibiting CDK6 protein expression (Supplementary Fig. 7). Additionally, the activation of MEK/ERK pathway also contributes to MM cell resistance to IMiDs.^52^ Our aforementioned results indicated that PM significantly inhibited “RAF-MEK-ERK-AKT” signaling pathway.^53^ Importantly, this synergistic therapeutic effect was also confirmed in the r/r MM patients in phase Ib/IIa, although only 2 patients were included so far (Supplementary Fig. 7).

In summary, this study highlights HDACs as attractive targets for the treatment of hematological malignancies and the potential for selective inhibition of I/IIb HDACs in the treatment of MM and lymphomas. These findings support ongoing phase Ib/IIa trials of PM combinations in r/r MM (NCT06484829) and phase II monotherapy trials in r/r DLBCL and T-cell lymphoma (NCT05563844, NCT06485219).

## Materials and methods

### Animal experiments

All animal studies were performed in accordance with the guidelines approved by the Institutional Animal Care and Use Committee of Sichuan University, Chengdu, China. Six- to seven-week-old female NOD-SCID mice were purchased from Beijing Vital River Laboratory Animal Technology Co., Ltd. (China). Mice received subcutaneous inoculation with 0.5 × 10^7^ MM1S or RPMI-8226 cells, to establish xenograft models. Heterotopic PDX models were generated as previously described.^54^ When tumors reached 150–250 mm^3^, mice were treated with various compounds. PM and panobinostat were administered intravenously three times a week at 5 mg/kg in combination therapy. The detailed dosing schedules of Len+Bort+DXM, panobinostat+Bort+DXM, R-CHOP, Pola-BR, PM+Pd, and Dara+ Bort+DXM are shown in Supplementary Table 9. Tumor volume (tumor volume (mm^3^) = π/6 × length × width^2^) and body weights were measured every two days. At the end, tumor tissues were weighed, and antitumor activity was evaluated by the tumor inhibition rate = (1 − tumor weight of treatment group/tumor weight of vehicle group) × 100%.

The cMYC-KRAS12V-induced plasmacytoma mouse model in BALB/c (GemPharmatech Co., Ltd., Nanjing, China) was established as previously described.^33^ On day 28 or 35 post bone marrow transplantation, recipients received various treatments. The 5TMM mouse model was established as previously described.^55^ The 5TGM1-luc cells were intravenously injected into 6-week-old C57BL/KawRij mice (Harlan Co. Netherlands) with 2 × 10^6^ cells per mouse. After three weeks, mice were treated with vehicle or PM 10 mg/kg. Serum Ca^2+^ levels were monitored weekly for three weeks after the start of administration. Kaplan-Meier survival curves were generated for each treatment group. Additionally, the 5TMM mouse model studying PM’s effects on immune response and bone destruction involved randomizing mice into vehicle and PM (10 mg/kg) groups one week after inoculation. T cell subsets were analyzed by flow cytometry 24 h after a single dose and 1 month after vehicle or PM treatment. The mice were sacrificed after 1 month of treatment, and the bone destruction was detected by Micro-CT.

### Bulk RNA-seq

MM1S cells were treated with DMSO (control), 5 nM PM or panobinostat for 24 h, and cells were collected for bulk RNA-seq. When tumor size reached around 500 mm^3^, MM1S subcutaneous tumor-bearing mice were given a single dose of vehicle, PM 10 mg/kg, or panobinostat 10 mg/kg, and the DEL PDX model mice were given a single dose of vehicle or PM 5 mg/kg. Tumor tissues were collected for bulk RNA-seq after 24 h. Total RNA was extracted using the TRIzol method and RNA integrity was evaluated using an Agilent 2100 Bioanalyzer (Agilent Technologies, Santa Clara, CA, USA). The bulk RNA-seq was performed by OE Biotech Co., Ltd. (Shanghai, China), and the analysis method was similar to our previously described.^53^

### Single cell RNA-seq

After establishing the 5TMM mouse model for three weeks, tumor-bearing mice were treated with vehicle (n = 2) and PM 10 mg/kg (n = 2) for 24 h. Bone marrow cells were then collected for scRNA-seq, which was performed by OE Biotech Co., Ltd. (Shanghai, China). Single cell suspension (700–1200 cells/μL) was processed using 10×Genomics Chromium Next GEM Single Cell 3ʹ Reagent Kits v3.1. The library was sequenced on the Illumina Nova 6000 PE150 platform. The scRNA-seq was performed by OE Biotech Co., Ltd. (Shanghai, China), and the analysis method was detailed provided in Supplementary Methods.

### Preparation of mouse osteoclasts and TRAP staining

Primary mouse BM cells were collected from 6-to-8 weeks C57BL/6 mice, and 1.0 × 10^5^ cells were seeded into 48-well plates with α-MEM containing 10% FBS and 40 ng/mL M-CSF (PeproTech, catalog # 315-02) for 3 days to recruit macrophage and then induce osteoclast differentiation plus 50 ng/mL RANKL (R&D, catalog # 462-TEC-010/CF) with or without indicated concentration of PM for 4 days. Half-media changes were carried out every 2 days. The TRAP staining kit was purchased from Sigma-Aldrich (CS0704), and TRAP staining was performed according to the kit instructions.

### Clinical phase I study

This open-label, non-randomized, first-in-human phase I dose-escalation two-center trial enrolled adult patients with lymphoma or MM who were refractory or relapsed after ≥1 prior regimen (NCT05526313). All patients provided written informed consent, and the study was conducted in accordance with the principles of the Declaration of Helsinki. PM was administered by 30-min intravenous infusion in a standard 3 + 3 dose escalation design, starting from 1.2, 2.4, 4.0, 6.0, 8.4, 11.2, up to 15 mg/m^2^. This trial was conducted in three sequential stages as follows: the first stage as a single dose (day 1), the second stage as multiple doses (day 8, 11, and 15), and the third stage as extended doses (day 1, 4, 8, 11 in 21-day cycles). The decision to proceed to the third stage is based on the patient’s continued benefit from the previous two stages. Patients continued to receive PM until unacceptable toxicity or disease progression. Between August 2020 and August 2022, 11 r/r MM and 18 r/r lymphoma patients were enrolled. The cutoff date for the assessment of safety and efficacy was July 7, 2023. The incidence and severity of adverse events were assessed according to the National Cancer Institute Common Terminology Criteria for Adverse Events (CTCAE 5.0). The efficacy of PM in patients with r/r MM was evaluated according to the standards of the Chinese Multiple Myeloma Diagnosis and Treatment Guidelines (2017 Revised) and the 2016 International Myeloma Working Group (IMWG). Minimal response (MR) (only for the evaluation of r/r MM): serum M protein decreased by 25–49% and 24 h light chain decreased by 50–89%. If soft tissue plasmacytoma exists at baseline, the sum of the product of perpendicular diameters (SPD) of measurable lesions is required to be reduced by ≥50%.^56,57^ The number and size of osteolytic lesions have not increased (compression fractures are allowed). Moreover, the efficacy of PM in r/r lymphoma patients was evaluated according to the standards of the International Working Group (IWG) Lymphoma Response Evaluation System (2017), in which the minor response (MR) is defined as a decrease of ≥10% in the sum of the longest diameters of target lymphoma lesions on FDG-PET or CT scans but did not achieve the PR.^58^ Detailed methods for pharmacodynamic studies, safety and tolerability assessments (including the definition of DLT and maximum tolerated dose), and efficacy assessment are provided in Supplementary Methods.

*Cell lines, compounds, cell viability, apoptosis, cell cycle, western blotting, immunohistochemistry, and RT-PCR assays* were described in detail in Supplementary Methods. Peripheral blood samples were obtained from patients at West China Hospital of Sichuan University, with approval from the Biomedical Ethics Review Committee of West China Hospital, Sichuan University (Approval No. 2020-624).

### Statistical analysis

Statistical analyses were performed in GraphPad Prism software version 9.4.1, R package Seurat version 4.1.0, and SAS version 9.4. GraphPad Prism 9.4.1 was used for graphing and statistics of preclinical cell and animal experiments on efficacy studies. Differences between two groups were evaluated by t-tests or two-sided Wilcoxon rank-sum tests. A p-value of less than 0.05 was considered statistically significant. Survival data were analyzed by a log-rank (Mantel-Cox) test. Bioinformatics statistical analyses were performed using R 4.1.0. For general differential analysis between groups (e.g., comparisons of gene set scores), the nonparametric Wilcoxon rank-sum test (Mann-Whitney U test) was applied. For differential gene expression analysis of transcriptomic data, the DESeq2 package (version 1.32.0) was employed. All efficacy and safety data of PM in the clinic were summarized using descriptive statistics by SAS Version 9.4. Detailed methods for clinical statistical analysis are described in the Supplementary Methods.

## Supplementary information

Supplementary methods, Supplementary figures and tables

Clinical Trial Protocol of Purinostat Mesylate for Injection (PM)

All original and uncropped whole membrane images of western blot results in the manuscript

## Data Availability

The raw sequencing data generated in this study have been deposited in the Gene Expression Omnibus (GEO) database under accession numbers GSE297562, GSE296480, GSE296833, and GSE296835. All data supporting the findings of this study are available within the manuscript and its Supplementary Materials. The data will be made available upon reasonable request, subject to the terms of consent and any applicable data use restrictions.
