# Supplementary material for: Preclinical and first-in-human of purinostat mesylate, a novel selective HDAC I/IIb inhibitor, in relapsed/refractory multiple myeloma and lymphoma
Source: Signal Transduct Target Ther. 2025 Jun 23;10:201. doi: 10.1038/s41392-025-02285-w (PMC12198407; doi:10.1038/s41392-025-02285-w)
Supplement: Supplementary file 1 — Supplementary methods, Supplementary figures and tables [file 41392_2025_2285_MOESM1_ESM.docx]

Supplementary Materials for

Preclinical and first-in-human of purinostat mesylate, a novel selective HDAC I/IIb inhibitor, in relapsed/refractory multiple myeloma and lymphoma

Linyu Yang^1,2†^, Qiang Qiu^1,2†^, Jie Wang^1,2,3†^, Yi Wen^1,2†^, He Li^1,2,3†^, Rui Liang^4^, Yunyu Feng^1,2^, Fang Wang^1,2^, Xiaojing Lin^1,2^, Minghai Tang^1,2^, Jianhong Yang^1,2^, Heying Pei^1,2^, Peng Zhao^5^, Jishi Wang^5^, Jin Xiang^6^, Jia Miao^6^, Li Zheng^6^, Ke Tan^4^, Yongsheng Wang^6^, Yiguo Hu^1,2*^, Lijuan Chen^1,2,4*^, Weili Zhao^7,8*^, Ting Niu^1,2,3*^

Correspondence to: [niuting@wchscu.cn](mailto:niuting@wchscu.cn)

**This PDF file includes:**

Materials and Methods

Figures. S1 to S7

Tables S1 to S10

**Materials and Methods**

**Compounds**

Pomalidomide (batch number: SY2101116) was purchased from Sichuan shuyan Biomedical Technology Co., Ltd. Dexamethasone (batch number: Y24O11G128470) was purchased from Shanghai yuanye Bio-Technology Co., Ltd. Daratumumab injection (batch number: LHS2633) was purchased from Cilag AG Xian Janssen Pharmaceutica Co., Ltd. Selinexor (batch number: 20211121) was purchased from Chengdu Huajeming Bio-Technology Co., Ltd. Lenalidomide (batch number: F0204B) and Bortezomib (batch number: M03258) was purchased from Dalian Meilun Biotech Co., Ltd.

**Cell viability assay**

PM, panobinostat, Pom and DXM were dissolved in DMSO and stored at -20°C freezer. Cells were seeded at a density of 1.0-3.0 × 10^4^ cells/well/100 μL in 96-well plates. After overnight incubation, cells were treated with the drug-containing culture medium for 24, 48, or 72 hours, and cell viability was measured using CCK8 (Oriscience, Chengdu, China) at the indicated time points with Biotek Cytation 3 at a wavelength of 450 nm. The IC_50_ values were calculated using GraphPad Prism 9.4.1, and Calcusyn 2.0 software was employed to calculate the CI values.

**Cell apoptosis analysis**

CD138^+^, CD38^+^, or CD20^+^ primary cells were obtained from plasma cell leukemia patients and DLBCL patients who had relapsed after multiple drug therapies. These patient samples were sourced from West China Hospital and had been approved by the clinical ethics committee of West China Hospital of Sichuan University (Chengdu, China). The clinical information of these patients was summarized in Supplementary Table 2. The patient primary cells were cultured in 6-well plates at a density of 1×10^6^ cells/mL, and treated with control, PM, panobinostat, Pom and DXM at indicated concentrations for 48 hours. CD138, CD38, and CD20 monoclonal antibodies, Annexin V-APC and 7AAD apoptosis detection kits were used following the manufacturer’s instructions. The apoptosis cells were analyzed by flow cytometry (Thermo, AttuneTMNxT).

**RT-PCR analysis**

The remaining RNA samples of MM1S subcutaneous tumor tissue for bulk RNA-seq were employed for RT-PCR. The RT-PCR primer sequences were listed in Supplementary Table 10. Following the instructions, RNA was reverse-transcribed to cDNA using the Evo M-MLV kit with gDNA Eraser (agbio, Cat # AG11711). The resulting cDNA served as a template for RT-PCR gene expression analysis using Magic SYBR Mixture (cwbio, Cat # CW3008S). Quantitative studies were conducted on various inflammatory factors, chemokines, and other indicators in the vehicle, PM 10 mg/kg, and panobinostat 10 mg/kg treatment groups, with three replicate samples in each group. β-Actin was utilized as the internal control, and mRNA expression levels were calculated using the delta-delta Ct method.

**Western blotting**

MM1S, MM1R, and TMD-8 cells were treated with control, PM, panobinostat, pomalidomide, or DXM at the indicated concentrations for 24 hours. RPMI-8226 xenograft mice were treated with vehicle, PM, PM+Pd, or Pd for 18 days. Tumor tissues from each treatment group were collected and cryogenically ground into single cells. Whole cells were lysed with RIPA buffer supplemented with protease and phosphatase inhibitors. Proteins were separated through PAGE gel electrophoresis, transferred onto PVDF membranes, and subjected to immunoblotting using primary antibodies, followed by incubation with horseradish peroxidase-conjugated secondary antibodies. Antibodies against c-MYC (Cat # CY5150) and β-ACTIN (Cat # AB0035) were purchased from Abways Technology in China. Antibodies against CDK6 (Cat # ET1612-3), IKZF1 (Cat # ET7101-25), IKZF3 (Cat # HA720110), MYD88 (Cat # ET1610-81) and IRF4 (Cat # ET7110-44) were purchased from Huabio in China. Antibody against Ac-H3 (Cat # Sc-56616) was purchased from Santa Cruz Biotechnology in the USA, and antibody against EZH2 (Cat # 5246S) was purchased from Cell Signaling Technology in the USA. The final signal was detected using enhanced chemiluminescence (ECL).

**Single cell RNA-seq (ScRNA-seq) analysis**

Cell Ranger software (version 6.0.1) aligned reads to the 10× Genomics GRCm38 reference genome. Seurat package (version 4.0.5) in R v4.1.0 handled data pre-processing, including quality control, normalization, variable gene identification, dimensionality reduction, and clustering. Cells with <300 captured genes or >20% mitochondrial genes were excluded. Gene expression values were log-normalized, and the 2500 most variable genes were identified. Principal component analysis and uniform manifold approximation and projection (UMAP) were used for clustering and visualization. InferCNV (version 1.8.1) identified plasma tumor cells based on scRNA-seq data. Genomic copy number variations (CNV) of individual cells were normalized to a range of [-1,1], avoiding bias due to absolute expression levels. CNV scores were calculated for each cell as the sum of the squares of normalized CNV expression values. The specific calculation for each cell on all genes $n$ is as follows:

$$CNVscore=\sum_{i=1}^{n} \left( -1+2\times\frac{x-x_{min}}{x_{max}-x_{min}} \right)^{2}$$

This method accurately gauges cell genomic instability by measuring overall copy number variation. Non-tumor cells are anticipated to show minimal CNV scores, reflecting their lower genomic instability compared to tumor cells, as seen in past studies ^1^.

"Pseudo-time analysis" is a computational method used in scRNA-seq studies to reconstruct the temporal sequence of cellular states or developmental trajectories. Pseudo-bulks were created by combining expression counts from each cell type within samples, yielding a single profile per sample. DESeq2 (v1.32.0) identified DEGs between groups (FDR < 0.05, |log2 fold change| > 0.5). Monocle (v2.20.0) analyzed pseudo-time in monocyte/monocyte progenitors, revealing differentiation trajectories in PM/vehicle groups. Seurat data in Monocle were ordered using DDRTree. The DDRTree (Discriminative Dimensionality Reduction Tree) algorithm, implemented in the Monocle package (version v2.20.0), was employed to construct the pseudo-temporal trajectory of cells in this study. DDRTree combines dimensionality reduction with tree-based trajectory inference to model complex cellular transitions. Specifically, it reduces the high-dimensional gene expression data into a lower-dimensional manifold while simultaneously learning a tree structure that captures the branching patterns of cellular states. This is achieved by optimizing a discriminative objective function that balances data fidelity and trajectory smoothness. In our analysis, DDRTree was applied after feature selection. The resulting tree structure was used to assign pseudo-time values to each cell and identify significant branching points corresponding to key regulatory transitions of monocytes. "BEAM" function identified differentially expressed genes between branches. ClusterProfiler (v4.0.5) performed gene ontology analysis.

To gain a deeper understanding of the molecular attributes of individual cells, an evaluation of single-cell RNA gene set scores was conducted using the UCell (version 1.1.0) package. This method consisted of calculating gene set scores for each cell based on the expression of a pre-defined set of genes, obtained from the MSigDB database (https://www.gsea-msigdb.org/gsea/msigdb/).

Bioinformatics statistical analyses were performed using R (version 4.1.0). For general differential analysis between groups (e.g., comparisons of gene set scores), the non-parametric Wilcoxon rank-sum test (also known as the Mann-Whitney U test) was applied. This test was chosen due to its robustness to non-normal data distributions and its suitability for unpaired comparisons. The Wilcoxon test was implemented using the wilcox.test function in base R, with p-values calculated to assess statistical significance, and a threshold of p < 0.05 was used unless otherwise specified. For differential gene expression analysis of transcriptomic data, the DESeq2 package (version 1.32.0) was employed. DESeq2 uses a negative binomial generalized linear model to detect differentially expressed genes between conditions, accounting for biological variability and sequencing depth. We employed cell chat (version 1.1.3) to predict major signaling inputs and outputs of all cells within the BM microenvironment. Subsequently, we compared these predictions between PM and vehicle, using a significance threshold of p < 0.05.

**Immunohistochemistry analysis**

The tumor tissues were harvested from the RPMI-8226 xenograft mice treated with either vehicle or drugs and fixed in 4% PFA for subsequent analysis. The immunohistochemistry was conducted using standard procedures. Following deparaffinization in xylene, rehydration in increasing concentrations of ethanol, and antigen retrieval, incubation with different primary and secondary antibodies was carried out using a semi-automatic Dako Autostainer (DAKO, Carpinteria, CA, USA) system. The primary antibodies, including Ac-H3, c-MYC, and IRF4, were the same as those employed in the western blot analysis. Ki67 (Cat # HA721115) was obtained from Huabio in China.

**Study design of phase I clinical trail**

This was an open-label, two-center, dose-escalating, first-in-human phase I trial (NCT05526313) of single-agent purinostat mesylate following a standard 3+3 design to enroll adult multiple myeloma and lymphoma patients who were refractory or relapsed after ≥1 prior regimen. Before the commencement of this study, the study protocol, case report forms, informed consent forms, and other documents had obtained review and approval from the Institutional Review Boards (IRBs) of the two research centers involved in this clinical trial, namely, the Clinical Trial Ethics Committee of West China Hospital, Sichuan University (Chengdu, China), and the Medical Ethics Committee of Affiliated Hospital of Guizhou Medical University (Guizhou, China). Men and women aged ≥18 years, diagnosed with hematologic malignancies through organized pathological or cytological examination, including multiple myeloma, B-cell lymphoma, and T-cell lymphoma. Additionally, participants must have experienced disease progression, recurrence, or be deemed unsuitable for standard treatment protocols after standard treatment regimens. PM was administered by 30-minute intravenous infusion, starting from 1.2, 2.4, 4.0, 6.0, 8.4, 11.2, up to the highest dose of 15 mg/m^2^. This trial was conducted in three sequential stages as follows: the first stage as a single dose (day 1), the second stage as multiple doses (day 8, 11, and 15), and the third stage as extended doses (day 1, 4, 8, 11 in 21-day cycles). The decision to proceed to the third stage is based on the patient's continued benefit from the previous two stages, and patients continued to receive PM until unacceptable toxicity or disease progression. If patient experiences dose-limiting toxicity (DLT) during the single-dose and multiple-dose phases, PM administration should be temporarily suspended until toxicity recovers to grade 1 or below.

To analyze the pharmacokinetics (PK) of PM, serial blood samples were collected at pre-dose and at 5 minutes, 10 minutes, 20 minutes, 30 minutes, 32 minutes, 35 minutes, 45 minutes, 1 hour, 2 hours, 4 hours, 8 hours, and 12 hours post-dose on day 1. During the multiple dosing phase, blood samples were collected at pre-dose on day 15, and 5 minutes, 10 minutes, 20 minutes, 30 minutes, 32 minutes, 35 minutes, 45 minutes, 1 hour, 2 hours, 4 hours, 8 hours, and 12 hours post-dose on day 15. PM plasma concentrations were determined using ultra performance liquid chromatography (UPLC) coupled with tandem mass spectrometry (MS-MS) detection. Pharmacokinetic parameters, including T_max_, C_max_, AUC_0-t_, AUC_0-∞_, t_1/2_, MRT, CL, Ke, and V, were calculated using the non-compartmental analysis (NCA) method with the professional pharmacokinetic software Phoenix WinNonlin (Version 8.1). Dose proportionality was assessed in single-dose experiments, while drug accumulation was evaluated in multiple-dose experiments.

**Safety and tolerability assessments**

Adverse events were classified using the International Dictionary of Medical Terms (MedDRA) classification system. Investigators are required to grade each adverse event according to its severity according to CTCAE version 5.0 ^2^. The safety evaluation indicators for this clinical trial include DLT, maximum tolerated dose (MTD), the incidence of adverse events and serious adverse events (SAE), along with their severity, vital signs, physical examinations, 12-lead electrocardiogram (with echocardiography if necessary), laboratory tests (complete blood count, urinalysis, blood biochemistry, coagulation function), and imaging examinations (CT or PET-CT or whole-body X-ray).All AE were recorded from the first PM dose until 30 days after the last dose. SAE refers to events that require hospitalization, prolong hospitalization, become disabled, affect workability, endanger life or death, cause congenital malformations, etc. during clinical trials of PM, and perform SAE processing according to the requirements of the 2020 version of China’s Good Clinical Practice (GCP).

A dose-limiting toxicity (DLT) was defined as any of the following AEs related to PM treatment (including definitely related, probably related, and possibly related) within 25 days after the first dose according to CTCAE version 5.0: (1) hematologic toxicities include Grade 3 febrile neutropenia, defined as absolute neutrophil count (ANC) < 1.0 × 10^9^/L with fever, temperature > 38.3°C, or temperature ≥ 38.0°C for more than 1 hour, or Grade 4 non-febrile neutropenia lasting ≥5 days, or grade 3 thrombocytopenia (25×10^9^/L < platelet count ≤50×10^9^/L) associated with grade 2 or higher bleeding, or grade 4 thrombocytopenia lasting ≥7 days with platelet transfusion required on two or more occasions; (2) non-hematologic toxicity of grade 3 or above, including but not limited to grade 3 or above QT interval prolongation, will be considered a DLT if the subject's baseline levels of alanine aminotransaminase (ALT), aspartate aminotransaminase (AST), or total bilirubin (TBIL) are ≤ grade 1; (3) any grade 5 adverse event; (4) patients interrupted treatment for more than 14 days due to failure to recover from toxicity. MTD was defined as the highest dose if ≤1 DLT occurs among 6 patients. If all planned dose cohorts have been completed, and no DLT is observed in the highest dose group, then the highest dose is determined as the MTD. At least three HDACis, including vorinostat ^3^, panobinostat ^4^, and romidepsin ^5^, have demonstrated clinical evidence of QT prolongation in phase I and/or II studies. QT prolongation poses a risk of malignant arrhythmias (torsade de pointes) and sudden cardiac death. Patients were assessed for QT prolongation using a 12-lead electrocardiogram (ECG), which was monitored before the first dose on day 1 and at 2 and 6 hours after dosing. Subsequent assessments will be conducted on day 2 and 7, 2 hours and 6 hours post-dose on Day 8, and before and 2 hours and 6 hours after dosing on day 11 and 15, with an additional assessment on day 25. During the extended dosing phase, assessments will be conducted once before dosing on days 1, 11, and 21, and on the time point of 2 hours post-dose each day. Additional assessments may be added as deemed necessary by the investigator.

**Efﬁcacy assessments**

Disease response of patients was measured according to the Chinese Multiple Myeloma Diagnosis and Treatment Guidelines (2017 Revised) and the 2016 International Myeloma Working Group (IMWG) for multiple myeloma or the International Working Group consensus response evaluation criteria in lymphoma (RECIL 2017). Patients need to be evaluated for efficacy after completing the cycle 1 of PM treatment (single and multiple dosing phases, day 25) and every 2 treatment cycles during the extended dosing phase. For r/r multiple myeloma, the efficacy was evaluated by quantification of M protein immunofixation electrophoresis of serum and urine. For r/r lymphoma, if sites with available positron emission tomography (PET) imaging, tumor volume was quantified by PET computed tomography (CT), and if sites where PET was not available, CT or magnetic resonance imaging was used. Objective response rate (ORR)=CR+PR+MR, and disease control rate (DCR)=CR+PR+MR+SD, and calculate their 95% confidence interval using the Clopper-Pearson method. The efficacy assessable population was determined to include all patients who were administered at least one dose of PM and had undergone at least one tumor assessment after the baseline.

**Statistical analysis of phase I clinical trail**

The dose escalation cohort size adheres to the conventional 3+3 dose escalation design commonly employed in oncology trials, and no formal sample size estimation was conducted. The safety population encompasses all participants in the dose escalation phase of the trial who received at least one dose of PM and had at least 1 safety assessment data. The analysis set includes patients who within the first 21 days after the initial dose, either used ≥80% of the planned dose or experienced a DLT after at least one administration. This analysis set will be used for the analysis and summarization of DLT events. The analysis of MTD is as described above. All patients who enter this study and receive at least 1 dose of PM and have at least 1 efficacy assessment data other than baseline will be classified into the FAS set. All efficacy and safety data were summarized using descriptive statistics by SAS Version 9.4. Results are reported as of data cutoff on July 7, 2023. The trial has been registered with the China National Medical Products Administration with registration number CXHL1800174.

**REFERENCES**

1 Peng, J. et al*.* Single-cell RNA-seq highlights intra-tumoral heterogeneity and malignant progression in pancreatic ductal adenocarcinoma. *Cell Res*. **29**, 725-738, (2019).

2 Freites-Martinez, A., Santana, N., Arias-Santiago, S. & Viera, A. Using the Common Terminology Criteria for Adverse Events (CTCAE - Version 5.0) to Evaluate the Severity of Adverse Events of Anticancer Therapies. *Actas Dermosifiliogr (Engl Ed)*. **112**, 90-92, (2021).

3 Badros, A. et al*.* Phase I study of vorinostat in combination with bortezomib for relapsed and refractory multiple myeloma. *Clin Cancer Res*. **15**, 5250-5257, (2009).

4 Giles, F. et al*.* A phase I study of intravenous LBH589, a novel cinnamic hydroxamic acid analogue histone deacetylase inhibitor, in patients with refractory hematologic malignancies. *Clin Cancer Res*. **12**, 4628-4635, (2006).

5 Shah, M. H. et al*.* Cardiotoxicity of histone deacetylase inhibitor depsipeptide in patients with metastatic neuroendocrine tumors. *Clin Cancer Res*. **12**, 3997-4003, (2006).


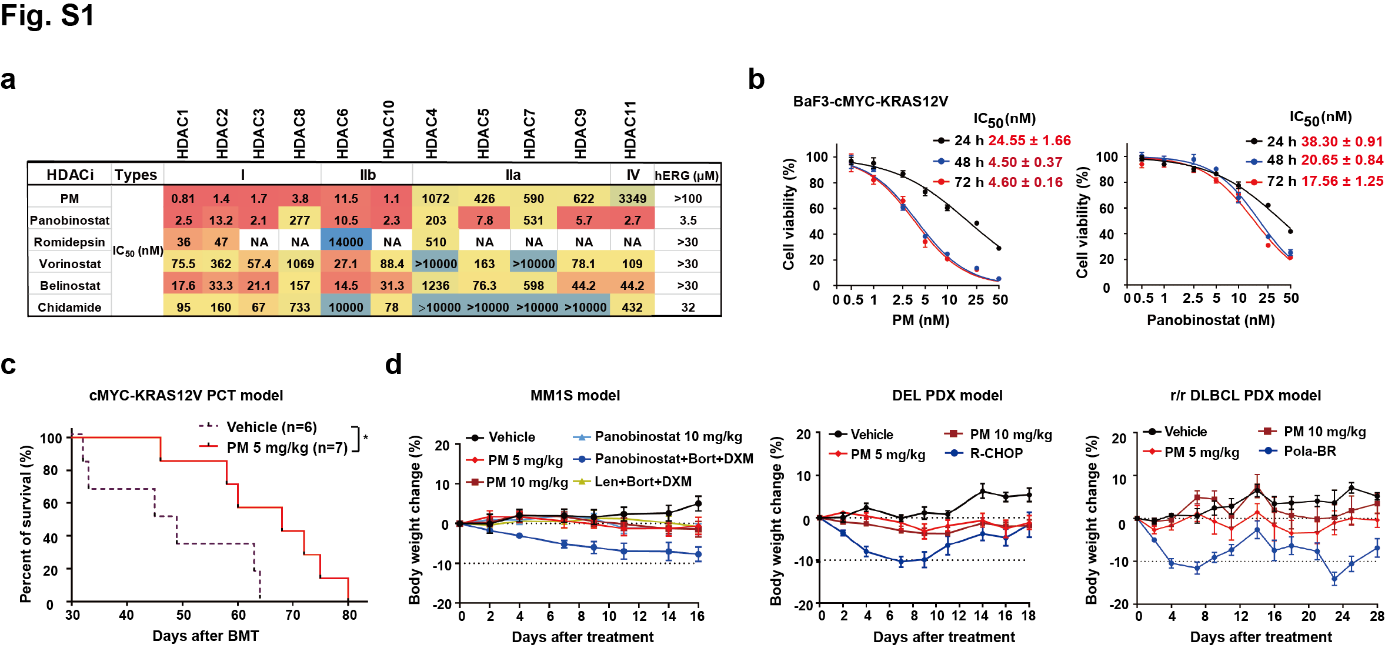


**Figure. S1.**

**High selective I/IIb HDACi PM exhibits potent activity against MM and lymphoma.** **a** IC_50_s of PM and marketed HDAC inhibitors against HDAC1-11 and hERG. **b** CCK8 determined cell viability of BaF3-cMYC-KRAS12V cells after treated with PM or panobinostat. **c** Kaplan-Meier survival curves represented the survival of cMYC-KRAS12V induced PCT mice treated with vehicle or PM 5 mg/kg. **d** Body weight changes of MM1S cell-derived xenograft mice (n=7/group), DEL (n=6/group) and r/r DLBCL (n=5/group) PDX model mice treated with the indicated drugs. Mice were weighed every two days. All data are represented as mean ± SEM.


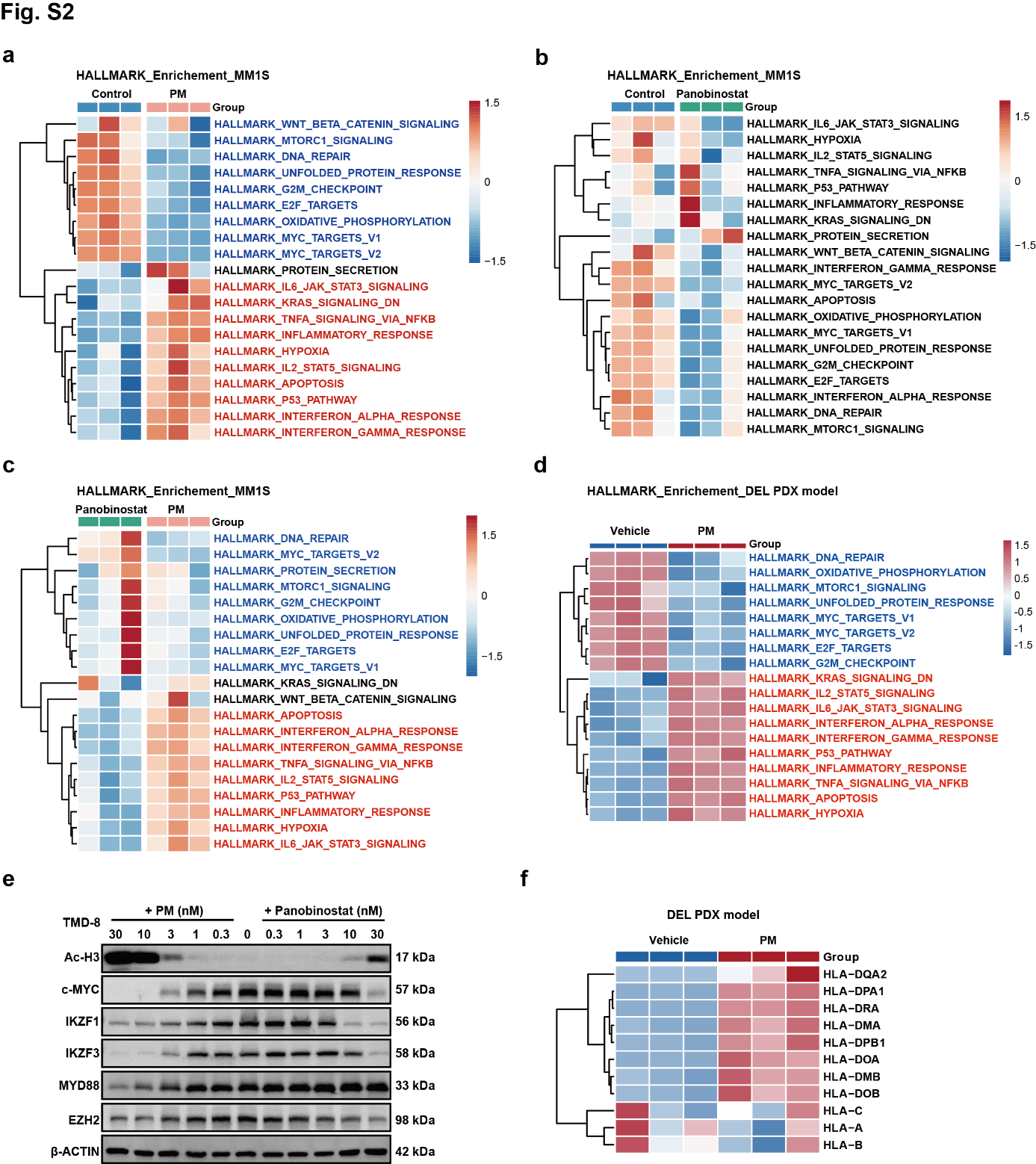


**Figure. S2.**

**PM alters multiple factors and gene sets associated with MM and lymphoma survival.** **a-c** Total RNA was extracted from MM1S cells treated with control (n=3), PM (5 nM, n=3) or panobinostat (5 nM, n=3) for 24 hours for bulk RNA-seq. Hallmark gene sets were generated by GSEA and represented as indicated. **d** Total RNA was extracted from tumor tissues of DEL PDX model mice treated with vehicle (n=3), or PM (5 mg/kg, n=3) for 24 hours for bulk RNA-seq. Hallmark gene sets were generated by GSEA and represented as indicated. **e** TMD-8 cells were treated with PM or panobinostat at indicated concentrations for 24 hours, and protein levels of Ac-H3, EZH2, c-MYC, IKZF1, IKZF3, and MYD88 were conducted with western blotting. β-ACTIN served as total protein loading control. **f, g** Heat-map of gene expression change (**f**) and GSEA (**g**) of MHC from RNA-seq of DEL PDX model tumor tissues.


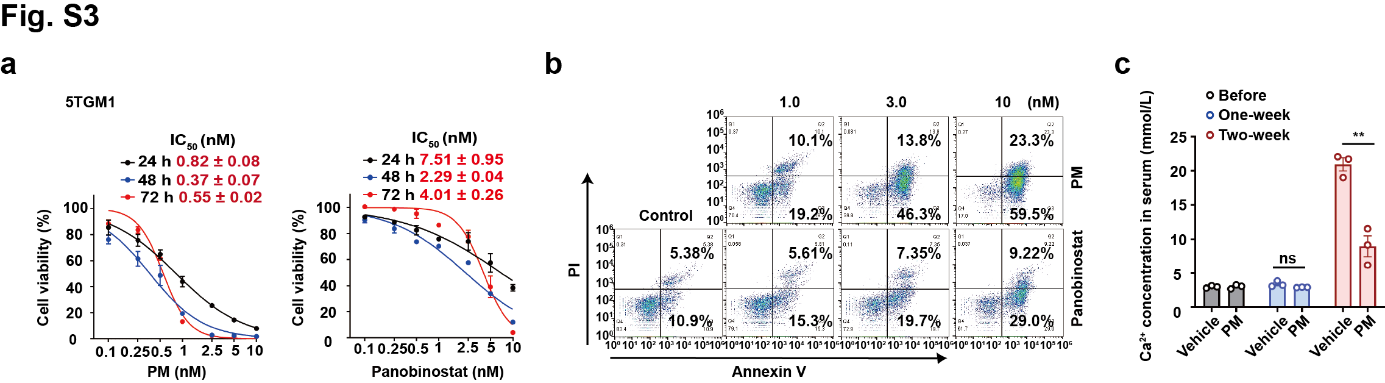


**Figure. S3.**

**PM inhibits the survival of 5TGM1 cells and reduces the serum calcium concentration of 5TMM mice. a** 5TGM1 cells were treated with PM or panobinostat at indicated concentrations for 24, 48, and 72 hours, and cell viability were analyzed by CCK8. **b** 5TGM1 cells were treated with PM or panobinostat at indicated concentrations, and cell apoptosis was analyzed after 48 hours. **c** Ca^2+^ concentrations in the serum of 5TMM mice at indicated time points post-PM treatment. All data are represented as mean ± SEM. ns P > 0.05, ** p < 0.01, *** p < 0.001, **** p < 0.0001, compared with vehicle.


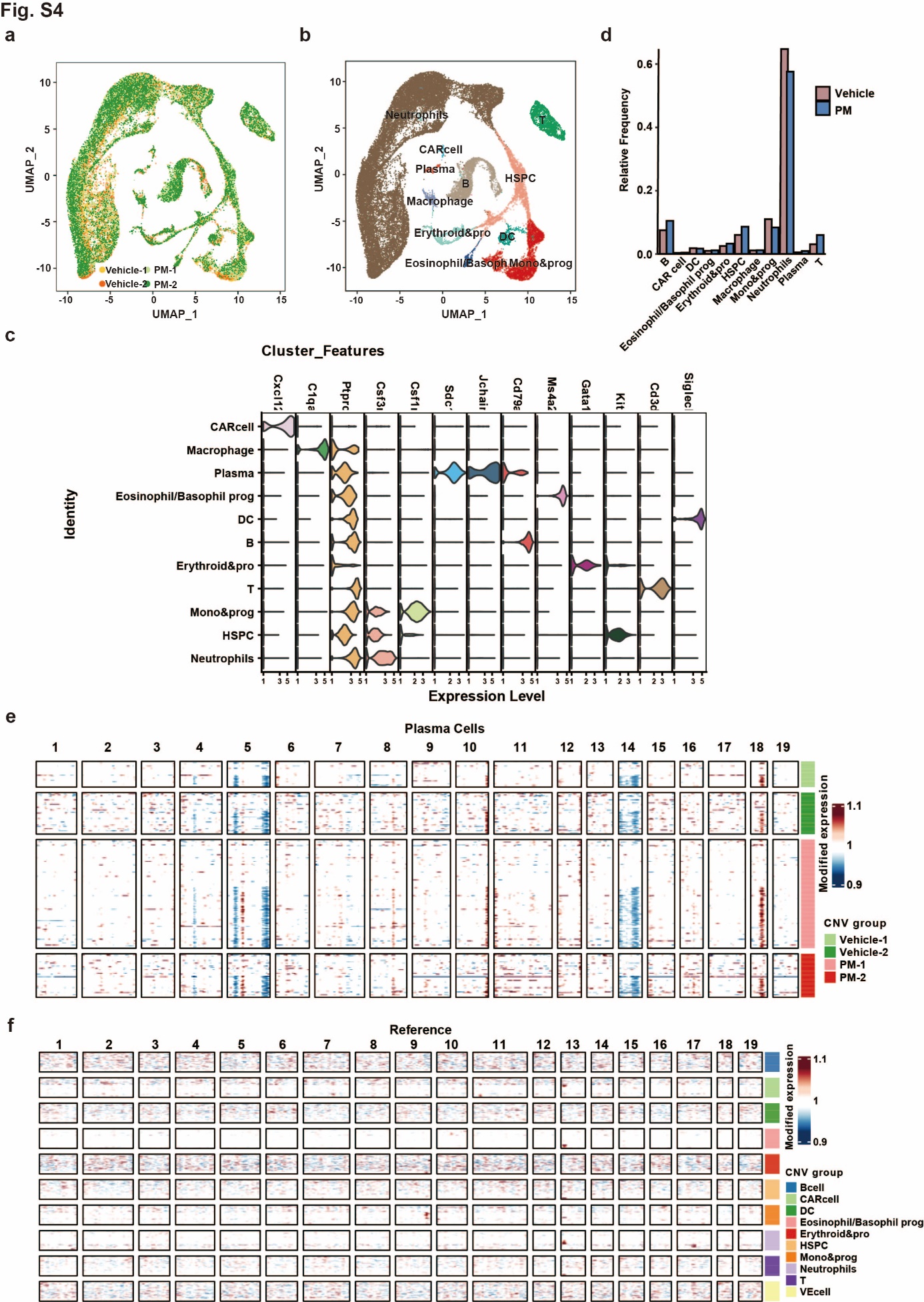


**Figure. S4.**

**ScRNA-seq analysis of BM cells from vehicle or PM treated 5TMM model mice. a** UMAP plots represented the subpopulation distributions of BM cells from 5TMM model mice treated with vehicle (n=2) and PM 10 mg/kg (n=2) for 24 hours. **b** Identification of cellular types and cluster annotation. **c** Violin plots depicted the canonical marker genes for the 11 cellular types. **d** The proportion of the cell types in the BM of 5TMM model mice treated with vehicle or PM. **e, f** Heatmap of genome-wide CNVs inferred from scRNA-seq data of plasma cells and other cell types in the BM of 5TMM model mice. Columns represent genome position across chromosomes. The red color represents high CNV level and blue represents low CNV level.

**
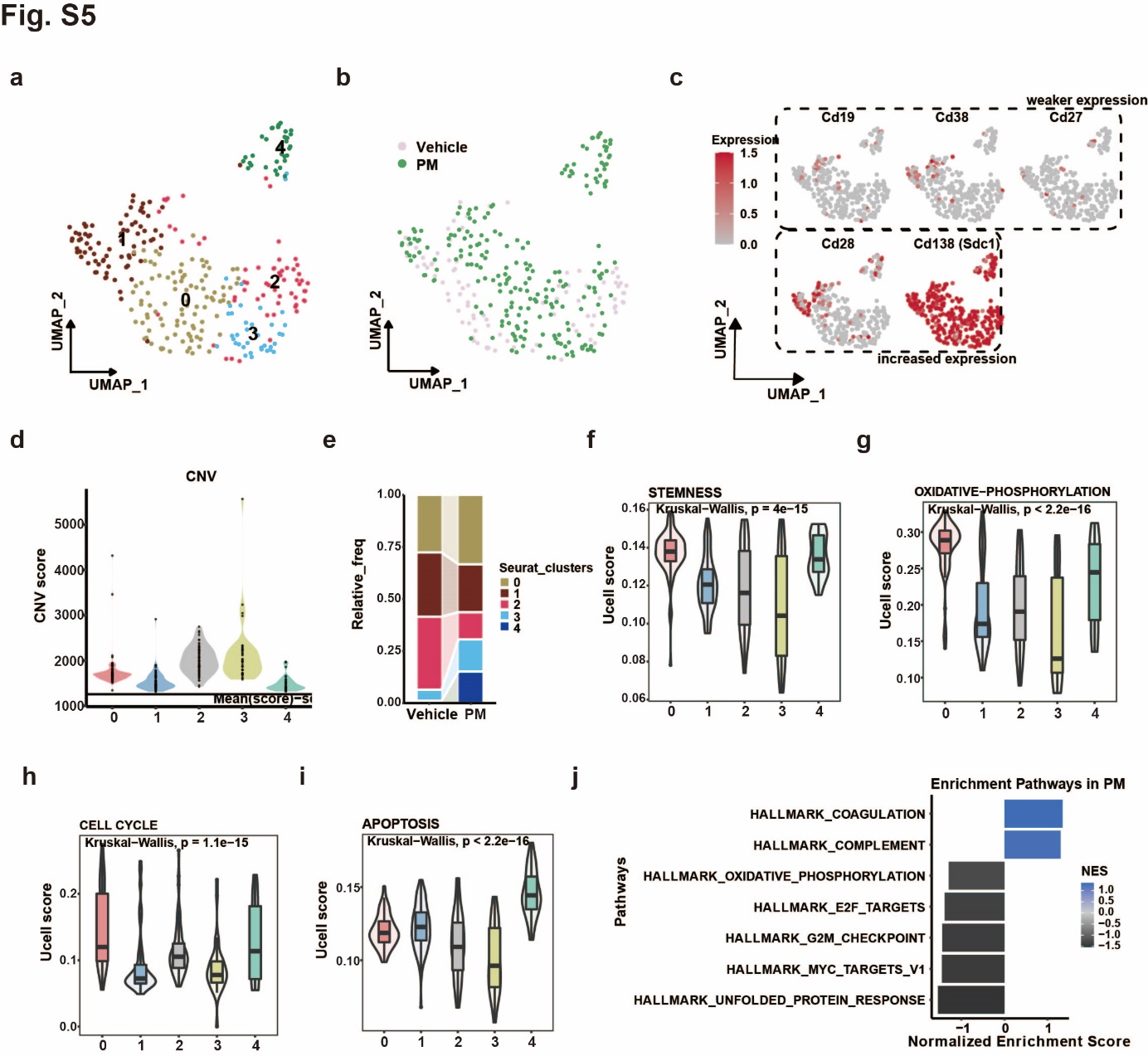
**

**Figure. S5.**

**ScRNA-seq and plasma cell activity analysis of 5TMM mouse model after PM treatment. a, b** UMAP plots represented the subpopulation distributions of tumor cells from BM of 5TMM model mouse treated with vehicle or PM. **c** Expression levels of Cd19, Cd27, Cd28, Cd38, and Cd138 (Sdc1) in tumor cells. **d** Violin plots depicted CNV score of the 5 cell clusters. **e** Proportion of the 5 tumor plasma cells. **f-i** Boxplots within violin plots depicted the gene set score of the 5 tumor plasma cells, including “APOPTOSIS”, “CELL CYCLE”, “STEMNESS” and “OXIDATIVE-PHOSPHORYLATION” gene sets. **j** The listed hallmark gene sets were significantly enriched in tumor cells from PM treated mice.


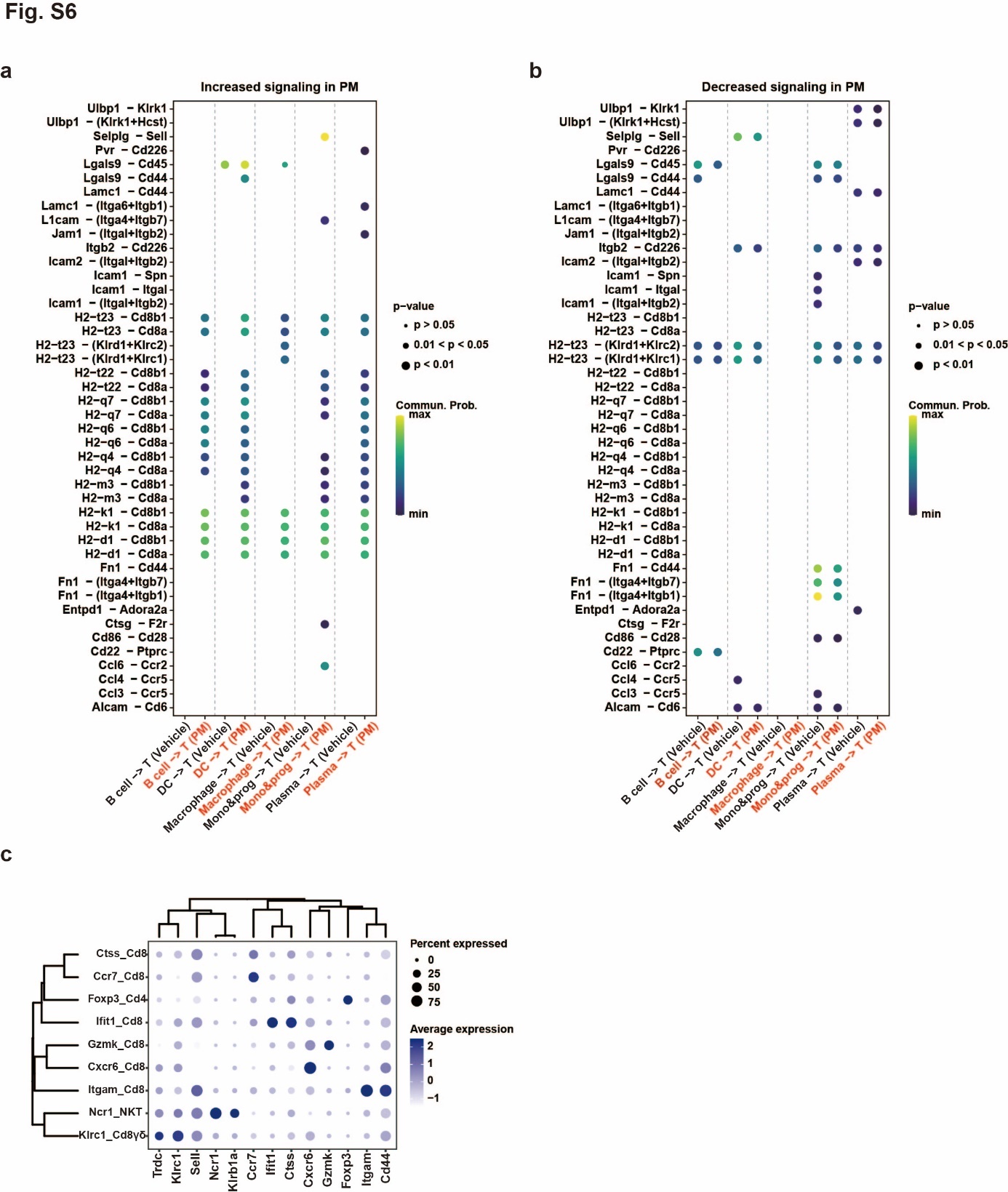


**Figure. S6.**

**PM affects cell communication between T cells and other cells in 5TMM model mice.** **a, b** Bubble heat map of ligand-receptor interaction landscape between various cells and T cells in the BM of 5TMM model mice treated with vehicle or PM. Color denotes the average expression level of ligands and receptors in interacting cells and the bubble size represents the significance of the interaction. **c** Dot plot showing the expression of representative genes for each indicated T cell type in BM of 5TMM model mice.


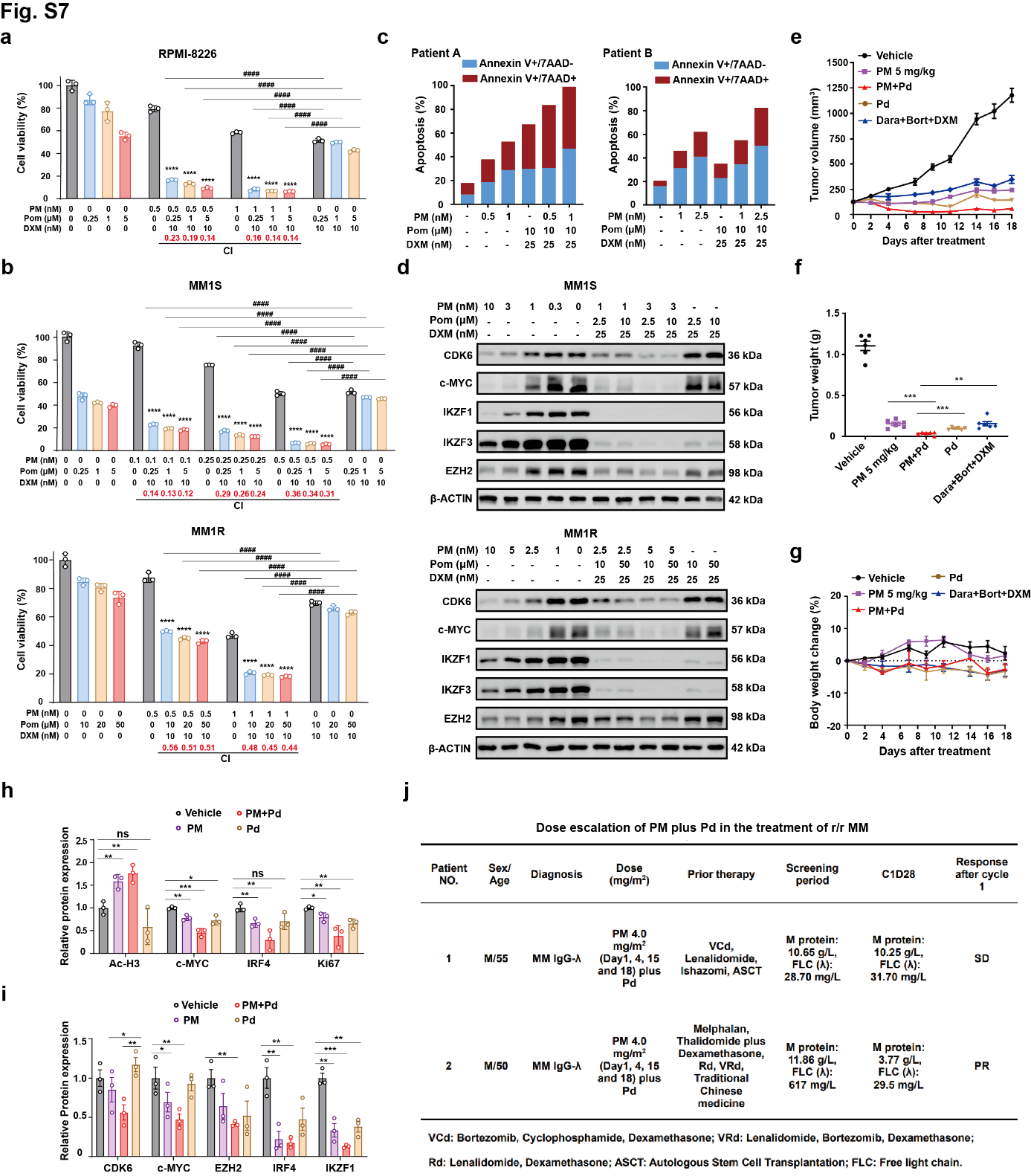


**Figure. S7.**

**The combination of PM, Pom and DXM shows synergistic anti-myeloma effects.** **a, b** RPMI-8226, MM1S and MM1R cells were treated with PM, Pom, and DXM at indicated concentrations for 72 hours, and the cell viability was measured using CCK8 assay, and the combination index (CI) value was calculated. **c** The apoptosis of CD138^+^ and CD38^+^ cells isolated from plasma cell leukemia patients treated with indicated concentrations of PM, Pom, and DXM for 48 hours. **d** MM1S and MM1R cell lines were treated for 24 hours, and protein levels were analyzed by western blotting. β-ACTIN served as the loading control. **e-g** tumor volume change (**e**), tumor weight (**f**), and mice weight change (**g**) of RPMI-8226 xenograft mice treated with vehicle, PM, PM+Pd, Pd, or Dara+Bort+DXM as indicated (n=6). **h** Immunohistochemistry showed protein levels in tumor tissues, and the results of each group were quantified using Image J (n=3). **i** Western blot analysis EZH2, c-MYC, IKZF1, IRF4, and CDK6 protein expression in RPMI-8226 tumor tissues. β-ACTIN served as loading control, and the protein expression levels were quantified using Image J with n=3 per group. **j** Information and treatment responses of the first two patients in a phase Ib/IIa clinical trial of PM combined with pomalidomide and dexamethasone acetate for the treatment of r/r MM. PM 4.0 mg/m^2^ administered by intravenous infusion on days 1, 4, 15, and 18; Pomalidomide capsules are taken orally at a dose of 4 mg per day for 21 consecutive days, followed by a one-week break. Dexamethasone acetate tablets are 20 mg orally administered on days 1, 2, 8, and 9, with a treatment cycle of 28 days. C1D28: First treatment cycle. All data are represented as mean ± SEM. ns p > 0.05, * p < 0.05, ** p < 0.01, *** p < 0.001, **** p < 0.000. ## p < 0.01, ### p < 0.001, #### p < 0.0001, compared with vehicle or the indicated group.

**Table S1 Antiproliferative activity IC_50_s of PM and panobinostat on multiple myeloma and** **lymphoma cell lines**

| **Cell lines** | | **PM** | **panobinostat** |
| --- | --- | --- | --- |
| **multiple myeloma** | **MM1S** | 0.47 ± 0.02 | 2.87 ± 0.12 |
|  | **MM1R** | 0.33 ± 0.01 | 0.87 ± 0.01 |
|  | **RPMI-8226** | 2.42 ± 0.40 | 9.30 ± 0.55 |
|  | **RPMI-8226R** | 2.02 ± 0.30 | 5.56 ± 0.49 |
|  | **AMO1** | 1.78 ± 0.14 | 3.45 ± 0.50 |
|  | **OPM2** | 1.78 ± 0.13 | 4.40 ± 0.27 |
|  | **ARD** | 0.42 ± 0.04 | 1.82 ± 0.16 |
|  | **KMS-11** | 1.99 ± 0.21 | 7.94 ± 0.59 |
| **lymphoma** | **RL** | 1.27 ± 0.14 | 6.43 ± 0.45 |
|  | **Ramos** | 1.24 ± 0.47 | 5.00 ± 0.53 |
|  | **Daudi** | 0.73 ± 0.51 | 6.99 ± 3.90 |
|  | **Raji** | 1.96 ± 0.18 | 5.48 ± 4.81 |
|  | **TMD-8** | 0.99 ± 0.14 | 9.12 ± 0.25 |
|  | **SUDHL-4** | 0.73 ± 0.05 | 6.72 ± 0.49 |
|  | **Karpas299** | 1.16 ± 0.14 | 4.03 ± 0.20 |
|  | **NKYS** | 0.77 ± 0.07 | 4.81 ± 0.14 |

**Table S2 Information of patient samples**

| **ID sample** | **Age** | **Gender** | **Source** | **Treatment information** | **Gene mutations** |
| --- | --- | --- | --- | --- | --- |
| Patient A | 71 | Male | PB | Relapse after BD, and BRD treatment, and resistant to lenalidomide and bortezomib | / |
| Patient B | 59 | Male | PB | Relapse after BRD treatment, and resistant to lenalidomide and bortezomib | / |
| Patient C | 54 | Male | PB | Relapse after R-CHOP, bendamustine plus rituximab, orelabrutinib combined with lenalidomide and selinexor | *TP53, DNMT3A* |
| Patient for DEL PDX model | 62 | Female | Tumor tissue | / | *KMT2D, MYD88, CDKN2D, TBL1XR1, SPEN* |
| Patient for r/r DLBCL PDX model | 24 | Female | Tumor tissue | Relapse after 6 cycles of R-CHOPE | *CARD11, CCND3, EBF1, EP300, SOCS1, TET2, BCL6, CD58, CD70, FAS, TNFAIP3, TNFRSF14* |

**Table S3 PM in dose escalation in r/r multiple myeloma**

| **Patient NO.** | **Sex/Age** | **Diagnosis** | **Dose**  **(mg/m^2^)** | **Prior therapy** | **Response after cycle 1** | **Best efficacy** |
| --- | --- | --- | --- | --- | --- | --- |
| **1** | F/57 | MM IgA κ | 1.2 | Vd, RVd, Rd (2 lines) | SD | SD |
| **2** | M/56 | MM IgG λ | 1.2 | VADR, RVd, Rd, VCd (3 lines) | PD | PD |
| **3** | M/58 | MM IgA λ | 1.2 | Vd, VCd, RVd (2 lines) | PD | PD |
| **4** | F/56 | MM IgA λ | 2.4 | Vd, VCd, RVd, VAD, IRd (4 lines) | SD | SD |
| **5** | M/57 | MM IgG κ | 2.4 | Rd, Dara+Vd, Dara maintenance (2 lines) | SD | SD |
| **6** | M/57 | MM IgG κ | 2.4 | VCd, ASCT, interferon, lenalidomide, clinical trial (2 lines) | SD | SD |
| **7** | M/71 | MM IgG λ | 4.0 | VCd, Rd, lenalidomide, TQB3602 clinical trial (2 lines) | SD | SD |
| **9** | M/49 | MM IgG κ | 4.0 | Vd, RVd, Rd (1 line) | SD | SD |
| **10** | M/69 | MM IgG λ | 6.0 | RVd, Vd, R, T, ICd (2 lines) | SD | MR |
| **12** | M/57 | MM IgG λ | 6.0 | TD, MPT, Cd, VADT, BDT, Rd, pomalidomide, Dara+BD, Dara maintenance, BCMA-CAR-T (7 lines) | PD | PD |
| **14** | F/55 | MM IgA κ | 8.4 | RVd, Vd, ASCT, IRd, bendamustine+IRd, Dara+Pd (3 lines) | SD | SD |

Vd: Bortezomib, Dexamethasone; RVd: Lenalidomide, Bortezomib, Dexamethasone; Rd: Lenalidomide, Dexamethasone; VADR: Vincristine, Pirarubicin, Dexamethasone, Lenalidomide; VCd: Bortezomib, Cyclophosphamide, Dexamethasone; VAd: Vincristine, Adriamycin, Dexamethasone; IRd: Ixazomib, Lenalidomide, Dexamethasone; Dara: Daratumumab; ASCT: Autologous Stem Cell Transplantation; Td: Thalidomide, Dexamethasone; MPT: Melphalan, Prednisone, Thalidomide; Cd: Cyclophosphamide, Dexamethasone; VADT: Vincristine, Epirubicin, Dexamethasone, Thalidomide; BDT: Bortezomib, Dexamethasone, Thalidomide.

**Table S4 PM in dose escalation in r/r lymphoma**

| **Patient NO.** | **Sex**  **/Age** | **Diagnosis** | **Dose**  **(mg/m^2^)** | **Prior therapy** | **Response after cycle 1** | **Best efficacy** |
| --- | --- | --- | --- | --- | --- | --- |
| **8** | F/41 | FL3a | 4.0 | R-CHOP (1 line) | PD | PD |
| **11** | M/55 | DLBCL (DEL) | 6.0 | R-CHOP, Chidamide+ R-CHOP, R-DHAP (2 lines) | PD | PD |
| **13** | M/34 | cHL | 8.4 | ABVD, ICE, PD-1 (2 lines) | SD | SD |
| **15** | F/58 | DLBCL | 8.4 | R-CHOP (1 line) | PR | CR |
| **16** | M/47 | DLBCL | 11.2 | R-CHOP (1 line) | PR | CR |
| **17** | F/72 | DLBCL | 8.4 | Surgery, traditional Chinese medicine | PR | CR |
| **18** | F/46 | FL3a | 11.2 | R-CHOP, R, IBI322 (2 lines) | SD | PR |
| **19** | M/51 | DLBCL | 11.2 | R-CHOP, Camrelizumab+R+GDPE, BEAC+R, ASCT (2 lines) | MR | CR |
| **20** | F/64 | DLBCL (DEL) | 8.4 | R-CHOP, R-DHAP+Zanubrutinib, Ibrutinib (2 lines) | SD | SD |
| **21** | M/22 | DLBCL | 11.2 | R-CHOP (1 lines) | PR | CR |
| **22** | M/50 | large B-cell lymphoma | 14 | R-CHOP, R-EPOCH, BR, R-Gemox  (4 lines) | SD | SD |
| **23** | M/34 | DLBCL (DEL) | 11.2 | R-CHOP, ASCT (1 lines) | PD | PD |
| **24** | M/65 | FL | 15 | CHOP, DHAP, R-CHOP, R-DHAP  (3 lines) | MR | MR |
| **25** | F/36 | DLBCL | 11.2 | CHOP, Radiation therapy, R-GemOXE, R-GDP, R-DHAP (3 lines) | PD | PD |
| **26** | F/55 | DLBCL (DEL) | 15 | Chidamide+R-CHOP, R-ICE (2 lines) | MR | CR |
| **27** | M/56 | PTCL-NOS | 15 | G-CHOP, Chidamide+ CHOP, GDICE, ASCT，Chidamide+IL-2, CD47/PD-L1 (4 lines) | PR | PR |
| **28** | F/57 | DLBCL | 15 | R-CHOP，Radiation therapy, Lenalidomide (1 lines) | MR | PR |
| **29** | M/51 | AITL | 15 | Azacitidine+CHOP, Chidamide+Azacitidine+CHOP, Chidamide+Azacitidine, Chidamide, Chidamide+Azacitidine+COEP (2 lines) | MR | MR |

R-CHOP: Rituximab, Cyclophosphamide, Doxorubicin, Vincristine, Prednisone; R-DHAP: Rituximab, Cisplatin, Cytarabine, Dexamethasone; ABVD: Doxorubicin, Bleomycin, Vinblastine, Dacarbazine; R-ICE: Rituximab, Ifosfamide, Carboplatin, Etoposide; GDPE: Gemcitabine, Cisplatin, Dexamethasone, Etoposide; R-EPOCH: Rituximab, Etoposide, Prednisone, Vincristine, Cyclophosphamide, Doxorubicin; BR: Rituximab, Bendamustine; R-Gemox: Rituximab, Gemcitabine, Oxaliplatin; BEAC: Bendamustine, Etoposide, Cytarabine, Cyclophosphamide.

**Table S5 Analysis of pharmacokinetic parameters after single and multiple intravenous infusions of PM**

| **Parameters** | **1.2 mg/m^2^** | **2.4 mg/m^2^** | **4.0 mg/m^2^** | **6.0 mg/m^2^** | **8.4 mg/m^2^** | **11.2 mg/m^2^** | **15 mg/m^2^** |
| --- | --- | --- | --- | --- | --- | --- | --- |
| **Day 1** | | | | | | | |
| **N** | 3 | 3 | 3 | 3 | 5 | 6 | 6 |
| **C_max_ (ng/mL)** | 38.77±17.20 | 81.48±7.67 | 153.23±40.61 | 225.90±26.96 | 298.79±94.60 | 484.27±109.86 | 635.50±100.81 |
| **AUC_0-t_ (h*ng/mL)** | 22.99±8.42 | 58.36±14.48 | 102.12±22.41 | 152.06±16.82 | 215.42±85.31 | 332.96±66.32 | 413.39±66.86 |
| **AUC_0-12h_ (h*ng/mL)** | 23.86±8.48 | 58.63±14.04 | 102.83±21.79 | 152.06±16.83 | 215.78±84.70 | 332.96±66.32 | 413.40±66.89 |
| **AUC_0-∞_ (h*ng/mL)** | 24.44±8.13 | 60.55±15.65 | 103.57±22.33 | 153.80±17.25 | 217.70±85.58 | 335.77±66.27 | 424.37±79.53 |
| **T_max_ (h)** | 0.50±0 | 0.44±0.10 | 0.50±0.00 | 0.44±0.10 | 0.48±0.09 | 0.50±0.00 | 0.40±0.17 |
| **t_1/2z_ (h)** | 2.81±2.91 | 3.67±2.44 | 2.30±0.70 | 2.99±0.38 | 2.67±0.32 | 2.58±0.39 | 3.51±2.67 |
| **V_z_ (L/m^2^)** | 220.81±239.74 | 193.74±106.83 | 128.28±24.15 | 169.59±26.89 | 163.83±51.81 | 128.19±32.99 | 171.29±97.28 |
| **CL_z_ (L/h/m^2^)** | 52.50±15.47 | 41.81±12.64 | 39.83±8.48 | 39.36±4.69 | 44.32±20.31 | 34.27±5.90 | 36.35±6.46 |
| **MRT_0-t_ (h)** | 0.58±0.28 | 0.79±0.39 | 0.67±0.07 | 0.80±0.17 | 0.76±0.10 | 0.78±0.19 | 0.72±0.15 |
| **MRT_0-∞_ (h)** | 1.34±1.31 | 1.32±0.78 | 0.84±0.11 | 0.97±0.17 | 0.92±0.14 | 0.90±0.22 | 1.19±1.06 |
| **Day 15** | | | | | | | |
| **N (NMiss)** | 2(1) | 3(0) | 3(0) | 3(0) | 5(0) | 5(1) | 6(0) |
| **C_max_ (ng/mL)** | 43.29±18.17 | 107.31±6.16 | 173.09±27.59 | 216.73±25.19 | 343.39±102.59 | 490.82±65.20 | 673.88±79.42 |
| **AUC_0-t_ (h*ng/mL)** | 27.70±14.15 | 65.76±2.82 | 114.26±15.77 | 147.79±11.20 | 226.12±77.89 | 350.88±38.93 | 442.70±73.27 |
| **AUC_0-12h_ (h*ng/mL)** | 28.23±13.85 | 66.75±2.72 | 114.26±15.78 | 147.81±11.18 | 226.45±77.39 | 350.87±38.93 | 442.70±73.27 |
| **AUC_0-∞_ (h*ng/mL)** | 28.49±14.18 | 67.13±2.72 | 115.49±15.74 | 149.95±11.29 | 227.97±77.64 | 354.12±38.62 | 445.79±73.88 |
| **T_max_ (h)** | 0.50±0 | 0.53±0.05 | 0.50±0.00 | 0.46±0.11 | 0.47±0.08 | 0.50±0.00 | 0.50±0.00 |
| **t_1/2z_ (h)** | 1.53±0.47 | 1.87±0.38 | 2.66±0.08 | 3.15±0.94 | 2.49±0.86 | 2.41±0.61 | 2.23±0.45 |
| **V_z_ (L/m^2^)** | 97.79±20.30 | 95.92±16.83 | 134.54±16.82 | 181.70±54.34 | 149.50±85.43 | 111.83±35.37 | 108.97±21.41 |
| **CL_z_ (L/h/m^2^)** | 48.09±23.93 | 35.80±1.48 | 35.06±4.70 | 40.16±2.90 | 41.00±15.95 | 31.89±3.61 | 34.38±5.33 |
| **MRT_0-∞_ (h)** | 0.86±0.30 | 0.95±0.17 | 0.97±0.08 | 1.01±0.09 | 0.77±0.08 | 0.99±0.25 | 0.76±0.09 |

**Table S6** **Adverse drug Reactions by SOC and PT**

| **SOC**  **PT** | | **1.2mg/m^2^** | **4.0mg/m^2^** | **6.0mg/m^2^** | **8.4mg/m^2^** | **11.2mg/m^2^** | **15mg/m^2^** | **Total** |
| --- | --- | --- | --- | --- | --- | --- | --- | --- |
| **N** | | 3 | 3 | 3 | 5 | 6 | 6 | 29 |
| **Investigations n (%)** | | 3(100.0) | 3(100.0) | 3(100.0) | 5(100.0) | 6(100.0) | 6(100.0) | 29(100.0) |
| decreased neutrophil count | | 3(100.0) | 3(100.0) | 2(66.7) | 5(100.0) | 4(66.7) | 6(100.0) | 25(86.2) |
| decreased white blood cell count | | 3(100.0) | 2(66.7) | 2(66.7) | 5(100.0) | 5(83.3) | 5(83.3) | 24(82.8) |
| decreased platelet count | | 3(100.0) | 3(100.0) | 2(66.7) | 5(100.0) | 4(66.7) | 5(83.3) | 24(82.8) |
| decreased lymphocyte count | | 0(0) | 1(33.3) | 3(100.0) | 2(40.0) | 5(83.3) | 4(66.7) | 16(55.2) |
| increased monocyte count | | 0(0) | 1(33.3) | 2(66.7) | 2(40.0) | 4(66.7) | 4(66.7) | 13(44.8) |
| QT interval prolongation on ECG | | 2(66.7) | 1(33.3) | 1(33.3) | 0(0) | 2(33.3) | 2(33.3) | 10(34.5) |
| ST-T segment changes on ECG | | 0(0) | 0(0) | 1(33.3) | 2(40.0) | 1(16.7) | 3(50.0) | 9(31.0) |
| T wave abnormalities on ECG | | 1(33.3) | 2(66.7) | 1(33.3) | 0(0) | 2(33.3) | 1(16.7) | 9(31.0) |
| ST segment abnormalities on ECG | | 1(33.3) | 0(0) | 0(0) | 1(20.0) | 0(0) | 2(33.3) | 5(17.2) |
| urine protein detection | | 0(0) | 0(0) | 0(0) | 0(0) | 0(0) | 4(66.7) | 4(13.8) |
| increased serum bilirubin | | 0(0) | 0(0) | 0(0) | 0(0) | 3(50.0) | 0(0) | 4(13.8) |
| increased urobilinogen in the urine | | 0(0) | 0(0) | 0(0) | 0(0) | 1(16.7) | 2(33.3) | 3(10.3) |
| high voltage on ECG | | 0(0) | 1(33.3) | 1(33.3) | 0(0) | 0(0) | 0(0) | 3(10.3) |
| increased γ-glutamyl transferase | | 0(0) | 0(0) | 0(0) | 0(0) | 1(16.7) | 1(16.7) | 2(6.9) |
| decreased monocyte count | | 0(0) | 1(33.3) | 0(0) | 0(0) | 1(16.7) | 0(0) | 2(6.9) |
| increased blood triglycerides | | 0(0) | 0(0) | 0(0) | 0(0) | 2(33.3) | 0(0) | 2(6.9) |
| increased blood creatinine | | 0(0) | 0(0) | 1(33.3) | 0(0) | 0(0) | 0(0) | 2(6.9) |
| QRS axis abnormalities | | 0(0) | 0(0) | 1(33.3) | 0(0) | 0(0) | 0(0) | 1(3.4) |
| increased troponin | | 1(33.3) | 0(0) | 0(0) | 0(0) | 0(0) | 0(0) | 1(3.4) |
| increased lymphocyte count | | 0(0) | 0(0) | 1(33.3) | 0(0) | 0(0) | 0(0) | 1(3.4) |
| ST segment abnormalities on ECG | | 1(33.3) | 0(0) | 0(0) | 0(0) | 0(0) | 0(0) | 1(3.4) |
| elevated blood unconjugated bilirubin | | 0(0) | 0(0) | 0(0) | 0(0) | 1(16.7) | 0(0) | 1(3.4) |
| elevated blood creatine phosphokinase MB | | 0(0) | 0(0) | 0(0) | 1(20.0) | 0(0) | 0(0) | 1(3.4) |
| elevated blood creatine phosphokinase | 0(0) | | 0(0) | 0(0) | 0(0) | 1(16.7) | 0(0) | 1(3.4) |
| decreased blood fibrinogen | 0(0) | | 1(33.3) | 0(0) | 0(0) | 0(0) | 0(0) | 1(3.4) |
| increased total bile acid | 0(0) | | 0(0) | 0(0) | 0(0) | 1(16.7) | 0(0) | 1(3.4) |
| **metabolic and nutritional diseases n (%)** | 3(100.0) | | 3(100.0) | 3(100.0) | 2(40.0) | 5(83.3) | 4(66.7) | 23(79.3) |
| hypophosphatemia | 0(0) | | 1(33.3) | 2(66.7) | 2(40.0) | 3(50.0) | 3(50.0) | 13(44.8) |
| hypokalemia | 2(66.7) | | 1(33.3) | 3(100.0) | 1(20.0) | 2(33.3) | 1(16.7) | 11(37.9) |
| hyponatremia | 2(66.7) | | 1(33.3) | 2(66.7) | 0(0) | 1(16.7) | 2(33.3) | 10(34.5) |
| reduced intake | 1(33.3) | | 0(0) | 1(33.3) | 1(20.0) | 5(83.3) | 2(33.3) | 10(34.5) |
| hypocalcemia | 2(66.7) | | 1(33.3) | 1(33.3) | 1(20.0) | 2(33.3) | 0(0) | 7(24.1) |
| hyperglycemia | 0(0) | | 0(0) | 3(100.0) | 1(20.0) | 0(0) | 2(33.3) | 7(24.1) |
| hypochloremia | 1(33.3) | | 0(0) | 1(33.3) | 0(0) | 2(33.3) | 0(0) | 4(13.8) |
| hypomagnesemia | 1(33.3) | | 1(33.3) | 0(0) | 1(20.0) | 0(0) | 0(0) | 4(13.8) |
| hyperuricemia | 0(0) | | 0(0) | 0(0) | 0(0) | 2(33.3) | 0(0) | 4(13.8) |
| hyperlipoidemia | 0(0) | | 1(33.3) | 1(33.3) | 1(20.0) | 1(16.7) | 0(0) | 4(13.8) |
| hypermagnesemia | 0(0) | | 2(66.7) | 0(0) | 0(0) | 0(0) | 0(0) | 3(10.3) |
| hypertriglyceridemia | 0(0) | | 0(0) | 0(0) | 1(20.0) | 0(0) | 1(16.7) | 2(6.9) |
| hypermagnesemia | 1(33.3) | | 0(0) | 0(0) | 0(0) | 0(0) | 1(16.7) | 2(6.9) |
| loss of appetite | 0(0) | | 1(33.3) | 0(0) | 0(0) | 0(0) | 0(0) | 2(6.9) |
| hypoalbuminemia | 0(0) | | 0(0) | 0(0) | 0(0) | 1(16.7) | 0(0) | 1(3.4) |
| hypercalcemia | 0(0) | | 1(33.3) | 0(0) | 0(0) | 0(0) | 0(0) | 1(3.4) |
| **gastrointestinal system diseases n (%)** | 3(100.0) | | 1(33.3) | 2(66.7) | 5(100.0) | 6(100.0) | 3(50.0) | 21(72.4) |
| nausea | 2(66.7) | | 1(33.3) | 1(33.3) | 5(100.0) | 3(50.0) | 2(33.3) | 15(51.7) |
| vomit | 1(33.3) | | 1(33.3) | 1(33.3) | 4(80.0) | 5(83.3) | 2(33.3) | 15(51.7) |
| diarrhea | 1(33.3) | | 0(0) | 0(0) | 0(0) | 1(16.7) | 2(33.3) | 4(13.8) |
| abdominal distension | 1(33.3) | | 0(0) | 0(0) | 1(20.0) | 1(16.7) | 1(16.7) | 4(13.8) |
| abdominal pain | 0(0) | | 0(0) | 0(0) | 1(20.0) | 0(0) | 1(16.7) | 2(6.9) |
| oral hypoesthesia | 0(0) | | 0(0) | 0(0) | 0(0) | 1(16.7) | 1(16.7) | 2(6.9) |
| gastroesophageal reflux disease | 0(0) | | 0(0) | 0(0) | 1(20.0) | 1(16.7) | 0(0) | 2(6.9) |
| constipation | 0(0) | | 0(0) | 0(0) | 1(20.0) | 0(0) | 0(0) | 1(3.4) |
| hematochezia | 0(0) | | 0(0) | 0(0) | 1(20.0) | 0(0) | 0(0) | 1(3.4) |
| angular cheilitis | 0(0) | | 0(0) | 0(0) | 0(0) | 1(16.7) | 0(0) | 1(3.4) |
| abdominal discomfort | 1(33.3) | | 0(0) | 0(0) | 0(0) | 0(0) | 0(0) | 1(3.4) |
| oral mucositis | 1(33.3) | | 0(0) | 0(0) | 0(0) | 0(0) | 0(0) | 1(3.4) |
| epigastric pain | 0(0) | | 0(0) | 0(0) | 1(20.0) | 0(0) | 0(0) | 1(3.4) |
| tongue ulcer | 0(0) | | 0(0) | 0(0) | 0(0) | 1(16.7) | 0(0) | 1(3.4) |
| duodenogastric reflux | 0(0) | | 0(0) | 0(0) | 1(20.0) | 0(0) | 0(0) | 1(3.4) |
| gastrointestinal hemorrhage | 1(33.3) | | 0(0) | 0(0) | 0(0) | 0(0) | 0(0) | 1(3.4) |
| gastritis | 0(0) | | 0(0) | 0(0) | 1(20.0) | 0(0) | 0(0) | 1(3.4) |
| dyspepsia | 0(0) | | 0(0) | 0(0) | 1(20.0) | 0(0) | 0(0) | 1(3.4) |
| bleeding gums | 0(0) | | 0(0) | 0(0) | 0(0) | 0(0) | 1(16.7) | 1(3.4) |
| belching | 1(33.3) | | 0(0) | 0(0) | 0(0) | 0(0) | 0(0) | 1(3.4) |
| **blood and lymphatic system diseases n (%)** | 2(66.7) | | 2(66.7) | 3(100.0) | 4(80.0) | 4(66.7) | 4(66.7) | 20(69.0) |
| anemia | 2(66.7) | | 2(66.7) | 3(100.0) | 4(80.0) | 4(66.7) | 4(66.7) | 20(69.0) |
| **various neurological diseases n (%)** | 2(66.7) | | 1(33.3) | 2(66.7) | 4(80.0) | 5(83.3) | 4(66.7) | 19(65.5) |
| headache | 2(66.7) | | 1(33.3) | 2(66.7) | 1(20.0) | 4(66.7) | 3(50.0) | 14(48.3) |
| dizziness | 0(0) | | 1(33.3) | 1(33.3) | 4(80.0) | 4(66.7) | 2(33.3) | 13(44.8) |
| head discomfort | 0(0) | | 0(0) | 0(0) | 0(0) | 0(0) | 0(0) | 1(3.4) |
| parageusia | 0(0) | | 0(0) | 0(0) | 0(0) | 0(0) | 1(16.7) | 1(3.4) |
| **systemic diseases and various reactions at the administration site n (%)** | 1(33.3) | | 0(0) | 1(33.3) | 3(60.0) | 6(100.0) | 6(100.0) | 19(65.5) |
| weakness | 0(0) | | 0(0) | 1(33.3) | 3(60.0) | 5(83.3) | 5(83.3) | 14(48.3) |
| fatigue | 1(33.3) | | 0(0) | 1(33.3) | 0(0) | 0(0) | 0(0) | 3(10.3) |
| fever | 0(0) | | 0(0) | 0(0) | 1(20.0) | 0(0) | 1(16.7) | 2(6.9) |
| heat and cold intolerance | 0(0) | | 0(0) | 0(0) | 0(0) | 1(16.7) | 0(0) | 1(3.4) |
| chill | 0(0) | | 0(0) | 0(0) | 1(20.0) | 0(0) | 0(0) | 1(3.4) |
| facial pain | 0(0) | | 0(0) | 0(0) | 1(20.0) | 0(0) | 0(0) | 1(3.4) |
| peripheral edema | 0(0) | | 0(0) | 0(0) | 0(0) | 0(0) | 0(0) | 1(3.4) |
| **infections and infestations diseases n (%)** | 1(33.3) | | 2(66.7) | 1(33.3) | 3(60.0) | 3(50.0) | 2(33.3) | 13(44.8) |
| herpes zoster | 0(0) | | 1(33.3) | 0(0) | 1(20.0) | 1(16.7) | 0(0) | 4(13.8) |
| urinary tract infection | 0(0) | | 0(0) | 0(0) | 1(20.0) | 0(0) | 1(16.7) | 3(10.3) |
| upper respiratory tract infection | 0(0) | | 2(66.7) | 0(0) | 1(20.0) | 0(0) | 0(0) | 3(10.3) |
| COVID-19 | 0(0) | | 0(0) | 0(0) | 0(0) | 0(0) | 1(16.7) | 1(3.4) |
| infectious pneumonia | 0(0) | | 0(0) | 0(0) | 0(0) | 1(16.7) | 0(0) | 1(3.4) |
| respiratory tract infection | 0(0) | | 0(0) | 1(33.3) | 0(0) | 0(0) | 0(0) | 1(3.4) |
| bacteremia | 0(0) | | 0(0) | 0(0) | 1(20.0) | 0(0) | 0(0) | 1(3.4) |
| appendicitis | 0(0) | | 0(0) | 0(0) | 0(0) | 1(16.7) | 0(0) | 1(3.4) |
| soft tissue infection | 0(0) | | 0(0) | 0(0) | 1(20.0) | 0(0) | 0(0) | 1(3.4) |
| upper respiratory tract bacterial infection | 1(33.3) | | 0(0) | 0(0) | 0(0) | 0(0) | 0(0) | 1(3.4) |
| periodontitis | 0(0) | | 0(0) | 0(0) | 0(0) | 0(0) | 0(0) | 1(3.4) |
| fungal skin infection | 0(0) | | 0(0) | 0(0) | 0(0) | 0(0) | 0(0) | 1(3.4) |
| herpesvirus infection | 0(0) | | 0(0) | 0(0) | 0(0) | 1(16.7) | 0(0) | 1(3.4) |
| **heart organ diseases n (%)** | 1(33.3) | | 1(33.3) | 1(33.3) | 1(20.0) | 4(66.7) | 4(66.7) | 13(44.8) |
| sinus tachycardia | 1(33.3) | | 0(0) | 1(33.3) | 0(0) | 2(33.3) | 3(50.0) | 7(24.1) |
| palpitation | 0(0) | | 0(0) | 0(0) | 0(0) | 2(33.3) | 2(33.3) | 4(13.8) |
| supraventricular extraventricular contraction | 0(0) | | 0(0) | 1(33.3) | 1(20.0) | 0(0) | 0(0) | 2(6.9) |
| atrial tachycardia | 0(0) | | 0(0) | 0(0) | 0(0) | 1(16.7) | 0(0) | 1(3.4) |
| extraventricular contraction | 0(0) | | 1(33.3) | 0(0) | 0(0) | 0(0) | 0(0) | 1(3.4) |
| pericardial effusion | 0(0) | | 0(0) | 0(0) | 0(0) | 1(16.7) | 0(0) | 1(3.4) |
| arrhythmia | 0(0) | | 0(0) | 0(0) | 0(0) | 1(16.7) | 0(0) | 1(3.4) |
| ventricular hypokinesia | 0(0) | | 1(33.3) | 0(0) | 0(0) | 0(0) | 0(0) | 1(3.4) |
| right bundle branch block | 0(0) | | 0(0) | 0(0) | 0(0) | 0(0) | 0(0) | 1(3.4) |
| left ventricular dysfunction | 0(0) | | 0(0) | 0(0) | 0(0) | 0(0) | 0(0) | 1(3.4) |
| sinus bradycardia | 0(0) | | 0(0) | 0(0) | 0(0) | 1(16.7) | 0(0) | 1(3.4) |
| **skin and subcutaneous tissue diseases n (%)** | 0(0) | | 0(0) | 1(33.3) | 1(20.0) | 3(50.0) | 3(50.0) | 10(34.5) |
| rash | 0(0) | | 0(0) | 0(0) | 1(20.0) | 1(16.7) | 1(16.7) | 3(10.3) |
| pruritus | 0(0) | | 0(0) | 1(33.3) | 0(0) | 0(0) | 1(16.7) | 3(10.3) |
| skin xerosis | 0(0) | | 0(0) | 0(0) | 0(0) | 2(33.3) | 0(0) | 2(6.9) |
| petechia | 0(0) | | 0(0) | 0(0) | 0(0) | 1(16.7) | 1(16.7) | 2(6.9) |
| skin pain | 0(0) | | 0(0) | 0(0) | 0(0) | 0(0) | 0(0) | 1(3.4) |
| vesicular rash | 0(0) | | 0(0) | 0(0) | 0(0) | 0(0) | 1(16.7) | 1(3.4) |
| alopecia | 0(0) | | 0(0) | 0(0) | 0(0) | 1(16.7) | 0(0) | 1(3.4) |
| **respiratory, thoracic and mediastinal diseases n (%)** | 1(33.3) | | 0(0) | 1(33.3) | 1(20.0) | 2(33.3) | 3(50.0) | 8(27.6) |
| tachypnea | 1(33.3) | | 0(0) | 0(0) | 1(20.0) | 1(16.7) | 0(0) | 3(10.3) |
| epistaxis | 0(0) | | 0(0) | 0(0) | 0(0) | 0(0) | 2(33.3) | 2(6.9) |
| nasal congestion | 0(0) | | 0(0) | 0(0) | 0(0) | 1(16.7) | 0(0) | 1(3.4) |
| pulmonary inflammation | 0(0) | | 0(0) | 1(33.3) | 0(0) | 0(0) | 0(0) | 1(3.4) |
| dyspnea | 0(0) | | 0(0) | 0(0) | 0(0) | 0(0) | 1(16.7) | 1(3.4) |
| cough | 0(0) | | 0(0) | 0(0) | 0(0) | 1(16.7) | 0(0) | 1(3.4) |
| **various musculoskeletal and connective tissue diseases n (%)** | 1(33.3) | | 0(0) | 0(0) | 2(40.0) | 0(0) | 2(33.3) | 7(24.1) |
| arthralgia | 0(0) | | 0(0) | 0(0) | 1(20.0) | 0(0) | 1(16.7) | 3(10.3) |
| limb pain | 1(33.3) | | 0(0) | 0(0) | 1(20.0) | 0(0) | 0(0) | 2(6.9) |
| backache | 0(0) | | 0(0) | 0(0) | 1(20.0) | 0(0) | 0(0) | 1(3.4) |
| musculoskeletal discomfort | 0(0) | | 0(0) | 0(0) | 0(0) | 0(0) | 0(0) | 1(3.4) |
| muscle weakness | 0(0) | | 0(0) | 0(0) | 0(0) | 0(0) | 1(16.7) | 1(3.4) |
| **kidney and urinary system diseases n (%)** | 1(33.3) | | 2(66.7) | 0(0) | 1(20.0) | 0(0) | 1(16.7) | 6(20.7) |
| hematuria | 1(33.3) | | 2(66.7) | 0(0) | 0(0) | 0(0) | 1(16.7) | 4(13.8) |
| proteinuria | 0(0) | | 0(0) | 0(0) | 0(0) | 0(0) | 0(0) | 1(3.4) |
| renal failure | 0(0) | | 0(0) | 0(0) | 1(20.0) | 0(0) | 0(0) | 1(3.4) |
| **hepato-biliary diseases n (%)** | 0(0) | | 0(0) | 0(0) | 0(0) | 1(16.7) | 1(16.7) | 3(10.3) |
| hepatic failure | 0(0) | | 0(0) | 0(0) | 0(0) | 1(16.7) | 1(16.7) | 2(6.9) |
| abnormal liver function | 0(0) | | 0(0) | 0(0) | 0(0) | 0(0) | 0(0) | 1(3.4) |
| **vascular and lymphatic diseases n (%)** | 0(0) | | 0(0) | 1(33.3) | 1(20.0) | 0(0) | 0(0) | 3(10.3) |
| hypotension | 0(0) | | 0(0) | 1(33.3) | 0(0) | 0(0) | 0(0) | 2(6.9) |
| hypertension | 0(0) | | 0(0) | 1(33.3) | 1(20.0) | 0(0) | 0(0) | 2(6.9) |
| **eye organ disease n (%)** | 0(0) | | 0(0) | 0(0) | 0(0) | 0(0) | 1(16.7) | 2(6.9) |
| conjunctival bleeding | 0(0) | | 0(0) | 0(0) | 0(0) | 0(0) | 1(16.7) | 1(3.4) |
| eyelid edema | 0(0) | | 0(0) | 0(0) | 0(0) | 0(0) | 0(0) | 1(3.4) |
| **mental disorder n (%)** | 0(0) | | 0(0) | 0(0) | 0(0) | 1(16.7) | 0(0) | 1(3.4) |
| insomnia | 0(0) | | 0(0) | 0(0) | 0(0) | 1(16.7) | 0(0) | 1(3.4) |

Note: SOC, System Organ Class; PT, Preferred Term.

MedDRA 25.0 is used for coding, and SOC and PT are arranged in descending order according to the total number of AE cases;

Number of patients: the number of cases of a subject in the same term (SOC or PT) is counted as 1 with the most severe degree.

**Table S7 Adverse event summary (n=29)**

| **Indicators n (%)** | **1.2 mg/m^2^** | **2.4 mg/m^2^** | **4.0 mg/m^2^** | **6.0 mg/m^2^** | **8.4 mg/m^2^** | **11.2 mg/m^2^** | **15 mg/m^2^** | **Total** | |
| --- | --- | --- | --- | --- | --- | --- | --- | --- | --- |
| **N** | 3 | 3 | 3 | 3 | 5 | 6 | 6 | 29 | |
| **TEAE** | 3(100.0) | 3(100.0) | 3(100.0) | 3(100.0) | 5(100.0) | 6(100.0) | 6(100.0) | 29(100.0) | |
| **ADR** | 3(100.0) | 3(100.0) | 3(100.0) | 3(100.0) | 5(100.0) | 6(100.0) | 6(100.0) | 29(100.0) | |
| **SAE** | 0(0) | 0(0) | 0(0) | 1(33.3) | 3(60.0) | 4(66.7) | 2(33.3) | 10(34.5) | |
| **SADR** | 0(0) | 0(0) | 0(0) | 1(33.3) | 2(40.0) | 3(50.0) | 2(33.3) | 8(27.6) | |
| **TEAEs leading to withdrawal from the trial** | 0(0) | 0(0) | 0(0) | 0(0) | 0(0) | 0(0) | 1(6.7) | 1(3.4) | |
| **ADRs leading to withdrawal from the trial** | 0(0) | 0(0) | 0(0) | 0(0) | 0(0) | 0(0) | 1(6.7) | 1(3.4) | |
| **TEAE grade ≥3** | 3(100.0) | 1(33.3) | 3(100.0) | 3(100.0) | 5(100.0) | 5(83.3) | 5(83.3) | 25(86.2) | |
| **ADR grade ≥3** | 3(100.0) | 1(33.3) | 3(100.0) | 3(100.0) | 5(100.0) | 5(83.3) | 5(83.3) | 25(86.2) | |
| **TEAEs leading to dose reduction or suspension** | 3(100.0) | 2(66.7) | 2(66.7) | 1(33.3) | 4(80.0) | 5(83.3) | 4(66.7) | 21(72.4) | |
| **ADRs leading to dose reduction or suspension** | 3(100.0) | 2(66.7) | 2(66.7) | 1(33.3) | 4(80.0) | 5(83.3) | 4(66.7) | 21(72.4) | |
| **TEAEs leading to discontinuation** | 0(0) | 0(0) | 0(0) | 0(0) | 0(0) | 0(0) | 1(16.7) | 1(3.4) | |
| **ADRs leading to discontinuation** | 0(0) | 0(0) | 0(0) | 0(0) | 0(0) | 0(0) | 1(16.7) | | 1(3.4) |

**Table S8 Assessment summary for response-evaluable dose-escalation patients (n=29)**

| **Indication** | **n** | **Best Response, n (%)** | | | | | **ORR (%)** | **DCR (%)** |
| --- | --- | --- | --- | --- | --- | --- | --- | --- |
|  |  | **CR** | **PR** | **MR** | **SD** | **PD** |  |  |
| **MM** | 11 | - | - | 1(9.1) | 7(63.6) | 3(27.3) | 9.1 | 72.7 |
| **DLBCL** | 11 | 6(54.5) | 1(9.1) | - | 1(9.1) | 3(27.3) | 63.6 | 72.7 |
| **FL** | 3 | - | 1(33.3) | 1(33.3) | - | 1(33.3) | 66.7 | 66.7 |
| **Other lymphomas*** | 4 | - | 1(25.0) | 1(25.0) | 2(50.0) | - | 50.0 | 100 |
| **Total** | 29 | 6(20.7) | 3(10.3) | 3(10.3) | 10(34.5) | 7(24.1) | 41.4 | 75.9 |

*Other lymphomas, including one case each of large B-cell lymphoma (SD), cHL (SD), PTCL-NOS (PR) and AITL (MR).

**Table S9 The dosing** **schedules for MM and lymphoma mouse models**

| Mouse models | Dosing regimen | Drugs | Dose (mg/kg) | Route | Dosing frequency |
| --- | --- | --- | --- | --- | --- |
| MM1S CDX model and cMYC-KRAS12V PCT model | Len (or Panobinostat) + Bort + DXM | Lenalidomide (Len) | 10 | p.o. | five consecutive days per week |
|  |  | Bortezomib (Bort) | 0.1 | i.v. | three times a week |
|  |  | Dexamethasone (DXM) | 1 | i.v. | three times a week |
|  |  | Panobinostat | 5 | i.v. | three times a week |
| RPMI-8226 CDX model | PM+Pd | PM | 5 | i.v. | three times a week |
|  |  | Pomalidomide | 2.5 | p.o. | five consecutive days per week |
|  |  | DXM | 1 | i.v. | three times a week |
|  | Dara+ Bort+DXM | Daratumumab (Dara) | 5 | i.v. | once a week |
|  |  | Bort | 0.1 | i.v. | three times a week |
|  |  | DXM | 1 | i.v. | three times a week |
| DEL PDX model | R-CHOP | Rituximab (R) | 10 | i.p. | once a week |
|  |  | Cyclophosphamide (C) | 20 | i.p. | 21 days/cycle, once on day 2 |
|  |  | Doxorubicin (H) | 1.25 | i.v. | 21 days/cycle, once on day 2 |
|  |  | Vincristine (O) | 0.2 | i.v. | 21 days/cycle, once on day 2 |
|  |  | Prednisolone (P) | 0.15 | p.o. | 21 days/cycle, dosing on days 2-6 |
| DLBCL PDX model | Pola-BR | Polatuzumab vedotin (Pola) | 2 | i.v. | 21 days/cycle, once on day 1 |
|  |  | Rituximab (R) | 10 | i.p. | once a week |
|  |  | Bendamustine (B) | 25 | i.v. | 21 days/cycle, dosing on days 2-3 |

CDX, cell derived xenograft; PDX, patient-derived tumor xenograft; i.p., Intraperitoneal; i.v., intravenous; p.o. Oral.

**Table S10 mRNA primer sequences for RT-PCR**

| Human gene | Forward (5’-3’) | Reverse (5’-3’) |
| --- | --- | --- |
| CCL7 | GTCCCCGGGAAGCTGTAATC | GCTTTGGAGTTTGGGTTTTCTT |
| CFH | GTGAAGTGTTTACCAGTGACAGC | AACCGTACTGCTTGTCCAAAA |
| DDX4 | TCATACTTGCAGGACGAGATTTG | AACGACTGGCAGTTATTCCATC |
| TLR3 | TTGCCTTGTATCTACTTTTGGGG | TCAACACTGTTATGTTTGTGGGT |
| CXCR1 | CTGACCCAGAAGCGTCACTTG | CCAGGACCTCATAGCAAACTG |
| CXCL10 | GTGGCATTCAAGGAGTACCTC | TGATGGCCTTCGATTCTGGATT |
| STAT4 | TGTTGGCCCAATGGATTGAAA | GGAAACACGACCTAACTGTTCAT |
| CCL25 | GGCCCTCATGCTGTAAAGAAG | TGCTGATGGGATTGCTAAACTT |
| GBP4 | ATGGGTGAGAGAACTCTTCACG | TGCGGTATAGCCCTACAATGG |
| RSAD2 | TTGGACATTCTCGCTATCTCCT | AGTGCTTTGATCTGTTCCGTC |
| IFI44 | TTTTCGATGCGAAGATTCACTGG | CCTGATGCGTTACATGCCCTT |
| MX2 | CACCGAGCTAGAGCTTCAGGA | CCGGGAAGGTCAATGATGGT |
| IFI27 | TGCTCTCACCTCATCAGCAGT | CACAACTCCTCCAATCACAACT |
| TLR7 | CACATACCAGACATCTCCCCA | CCCAGTGGAATAGGTACACAGTT |
| IFIT3 | AAAAGCCCAACAACCCAGAAT | CGTATTGGTTATCAGGACTCAGC |
| MX1 | GTTTCCGAAGTGGACATCGCA | CTGCACAGGTTGTTCTCAGC |
| TLR9 | CTGCCTTCCTACCCTGTGAG | GGATGCGGTTGGAGGACAA |
| IFIT1 | AGAAGCAGGCAATCACAGAAAA | CTGAAACCGACCATAGTGGAAAT |
| BCMA | ACCTTGTCAACTTCGATGTTCTT | CAGAGAATCGCATTCGTTCCTT |
| β-ACTIN | GGCTGTATTCCCCTCCATCG | CCAGTTGGTAACAATGCCATGT |
