# Supplementary material for: Preclinical and first-in-human of purinostat mesylate, a novel selective HDAC I/IIb inhibitor, in relapsed/refractory multiple myeloma and lymphoma
Source: Signal Transduct Target Ther. 2025 Jun 23;10:201. doi: 10.1038/s41392-025-02285-w (PMC12198407; doi:10.1038/s41392-025-02285-w)
Supplement: Supplementary file 2 — Clinical Trial Protocol of Purinostat Mesylate for Injection (PM) [file 41392_2025_2285_MOESM2_ESM.docx]

Clinical Trial Protocol of Purinostat Mesylate for Injection (PM) in Relapsed or Refractory Hematologic Tumors, Mainly B-cell Related Malignancies: A Single-Center, Dose-Escalation Phase I Study Evaluating Tolerance, Safety, Pharmacokinetics and Pharmacodynamics

Research Drugs: Purinostat Mesylate for Injection (PM)

Program No.: GZ2018-001-1.0

Version / Date: Version 1.4 / March 18, 2022

**Research unit: West China Hospital, Sichuan University**

**Organizer: Chengdu Zenitar Biomedical Technology Co., Ltd/ West China Hospital, Sichuan University**

**Biological Sample Analysis and Testing Unit: National Drug Clinical Trial Institution / Clinical Pharmacology Research Laboratory of West China Hospital, Sichuan University**

**Data Management and Statistics Unit: Beijing Bozhiyin Technology Co., Ltd.**

**Confidentiality Statement**

**The ownership of all information contained in this document belongs to Chengdu Zenitar Biomedical Technology Co., Ltd. and West China Hospital, Sichuan University. This information is provided solely for review by medical experts related to this trial, researchers participating in the trial, and other personnel associated with the trial, as well as the medical institutions, ethics committees, and contract research organizations involved in this trial. Without the written approval of Chengdu Zenitar Biomedical Technology Co., Ltd. and West China Hospital, Sichuan University, it is strictly prohibited to disclose any information to third parties not related to this research, except for necessary explanations to subjects signing informed consent forms who may participate in this study.**

**Sponsor**

| Company Name | **Chengdu Zenitar Biomedical Technology Co., Ltd** | | | |
| --- | --- | --- | --- | --- |
| Project Manager | Liangkun Sun | | | |
| Contact Address | 9th Floor, Building E3, Frontier Medical Center, No. 16 Hemin Street, Chengdu High-tech Zone | | Postal Code | 610212 |
| Telephone | 028-86757388 | Mobile Phone | 15885742617 | |
| Fax | 028-86757773 | E-mail | bailing_stt@126.com | |
| Company Name | West China Hospital, Sichuan University | | | |
| Project Manager | Lijuan Chen | | | |
| Contact Address | State Key Laboratory of Biotherapy, Sichuan University, No. 17, Section 3, Renmin South Road, Chengdu | | Postal Code | 610041 |
| Telephone | 028-86757388 | Mobile Phone | 18980601790 | |
| Fax | 028-86757773 | E-mail | chenlijuan125@163.com | |

**Clinical Research Unit**

| Unit Name | West China Hospital, Sichuan University | | |
| --- | --- | --- | --- |
| Responsible person | Yongsheng Wang, Ting Niu | | |
| Address | No. 37, Guoxue Alley, Wuhou District, Chengdu | | |
| Mobile Phone | 18980602258 | Postal Code | 610041 |
| E-mail | wangy756@163.com | | |

**Clinical Participation Units**

| Unit Name | Affiliated Hospital of Guizhou Medical University | | |
| --- | --- | --- | --- |
| Responsible person | Jishi Wang | | |
| Address | No. 28, Guiyi Street, Guiyang City, Guizhou Province | | |
| Mobile Phone | 13639089646 | Postal Code | 550004 |
| E-mail | Wangjishi9646@163.com | | |

**Biological Sample Analysis and Testing Unit**

| Unit Name | National Drug Clinical Trial Institution / Clinical Pharmacology Research Laboratory of West China Hospital, Sichuan University | | |
| --- | --- | --- | --- |
| Responsible person | Yongsheng Wang | | |
| Address | No. 37, Guoxue Alley, Wuhou District, Chengdu | Postal Code | 610041 |
| Mobile Phone | 18980602258 | | |
| E-mail | wangy756@163.com | | |

**Data Management Unit**

| Company Name | Beijing Bozhiyin Technology Co., Ltd. | | | |
| --- | --- | --- | --- | --- |
| Responsible person | Hongxia Liu | | | |
| Address | Room 2-2209, Times Sail Building, No. 15, Xiluoma Street, Fengtai District, Beijing | | Postal Code |  |
| Mobile Phone | 010-67587407 | Fax | 010-67538432 | |
| E-mail | hongxia.liu@drugchina.net | | | |

**Statistical Analysis Unit**

| Company Name | Beijing Bozhiyin Technology Co., Ltd. | | | |
| --- | --- | --- | --- | --- |
| Responsible person | Hongxia Liu | | | |
| Address | Room 2-2209, Times Sail Building, No. 15, Xiluoma Street, Fengtai District, Beijing | | Postal Code |  |
| Mobile Phone | 010-67587407 | Fax | 010-67538432 | |
| E-mail | hongxia.liu@drugchina.net | | | |

**CRO Company**

| Company Name | Ouyahuizhi (Beijing) Medical Research Co., Ltd. | | | |
| --- | --- | --- | --- | --- |
| Project Manager | Gubo Li | | | |
| Address | 7th Floor, Building 2, Hanwei International II, No. 186, Nansihuan West Road, Fengtai District, Beijing | | Postal Code | 100070 |
| Mobile Phone | 15308008061 | Fax | 010-83739030 | |
| E-mail | ligubo@healtech.com.cn | | | |

**Program signature page**

**Researcher's statement**

I have received the Investigator's Brochure and am aware of the preclinical research status of the investigational drug. I have been informed that I will receive updated versions of the Investigator's Brochure in a timely manner. I have read and thoroughly understood the contents of this protocol, and I commit to diligently fulfilling my responsibilities as an investigator in accordance with the Good Clinical Practice (GCP) guidelines. I will conduct this clinical trial according to the design and stipulations of this protocol, and I will provide all personnel involved in this trial with detailed information regarding the trial materials, regulations, and responsibilities.

I will strictly adhere to the Declaration of Helsinki and provide potential subjects with detailed information about this clinical trial, which has been approved by the ethics committee, and obtain their informed consent. I will be responsible for making medical decisions related to this clinical trial and ensuring that subjects receive appropriate treatment in the event of adverse events during the trial. If a serious adverse event occurs during this trial, I will take immediate appropriate measures to ensure the safety of the subjects and report to the relevant units as required by GCP guidelines.

I agree to accept the inspections by monitors or auditors dispatched by the sponsor and by the drug regulatory authorities to ensure the quality of the clinical trial.

I agree to keep confidential all information related to this protocol that I receive or obtain during the trial.

**Responsible Unit for Clinical Research: West China Hospital, Sichuan University**

Address: No. 37, Guoxue Alley, Wuhou District, Chengdu, Sichuan Province

Principal Investigator's Name: Ting Niu, Yongsheng Wang

Professional Title: Chief physician, Chief physician

Position: Director of Hematology, Director of CTC

Telephone: 18980601242, 18980602258

Principal Investigator's Signature:
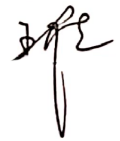

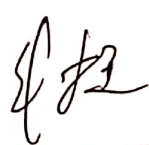


Date:2022-4-1

**Program signature page**

**Testing Unit’s statement**

I have carefully read and understood the protocol for this trial and agree to the contents of the research plan. I will conduct the clinical trial in accordance with the Declaration of Helsinki, the Good Clinical Practice (GCP) guidelines (2020 edition), and relevant laws, regulations, and guiding principles. As the person responsible for biological sample analysis, I guarantee that the methodologies I have established comply with national regulations, are authentic, scientific, and operational, and ensure that the testing data is true and reliable.

I will keep the research protocol and related content confidential. I agree to maintain the confidentiality of all information related to this protocol that I receive or obtain during the trial. **Biological Sample Analysis Unit: West China Hospital, Sichuan University**

Address: No. 37, Guoxue Alley, Wuhou District, Chengdu, Sichuan Province

Name of the Person Responsible for Biological Samples: Jin Xiang

Position: Director of the CTC Office

Telephone: 13880932568

Signature of the Person Responsible for Biological Sample Analysis:
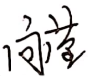


Date: 2022-3-28

**Program signature page**

**Sponsor's statement**

Our company will, in accordance with the Good Clinical Practice (GCP) guidelines (2020 edition), be responsible for initiating, applying for, organizing, and monitoring this clinical trial, as well as providing funding for the trial. We will specifically provide compensation for treatment related to any damages or deaths of subjects that occur during the trial and will provide legal and financial guarantees to the investigators.

We agree to fulfill the responsibilities of the sponsor in accordance with the Declaration of Helsinki and the Good Clinical Practice (GCP) guidelines (2020 edition). We will be responsible for establishing a quality management system for the clinical trial, managing quality based on risk, ensuring that the results of the clinical trial are true and reliable, and protecting the rights and safety of subjects in the clinical research.

I will keep the research protocol and related content confidential.

Sponsor Unit**:** Chengdu Zenitar Biomedical Technology Co., Ltd/ West China Hospital, Sichuan University

Address: 9th Floor, Building 3, No. 16 Humin Street, Chengdu High-tech Zone, China (Sichuan) Pilot Free Trade Zone

Name of the Authorized Signatory: Liangkun Sun

Position: Manager

Telephone: 15885742617

Signature of the Authorized Signatory:
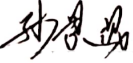


Date: 2022-3-22

**Program signature page**

**Monitor’s Statement**

I participated in the discussion of this clinical trial protocol and have carefully read and understood the protocol. I agree to conduct the clinical trial in accordance with the provisions of this protocol and all applicable laws and regulations. I will fulfill the responsibilities of the monitor in accordance with the Declaration of Helsinki and the Good Clinical Practice (GCP) guidelines (2020 edition).

I will monitor whether the rights and safety of subjects in the clinical research are protected, ensure that the clinical trial researchers and institutions have the appropriate qualifications, resources, or conditions, and ensure that the clinical trial is implemented and recorded correctly according to the protocol with proper document preservation. I will ensure that the handling and management of the drug comply with relevant laws, regulations, and the requirements of the sponsor.

I will verify that all medical reports, records, and documents provided by the investigators are traceable, clear, contemporaneous, original, accurate, and complete, with dates and trial numbers indicated. I will ensure the accuracy and completeness of the entries in the pathology report forms and compare them with source documents. I will confirm that adverse events are reported in a timely manner in accordance with relevant laws, regulations, and other stipulations. I will monitor whether the research adheres to the approved protocol, GCP, and relevant regulations.

I agree to keep confidential all information related to this protocol that I receive or obtain during the trial.

**Contract Research Organization:** Ouyahuizhi (Beijing) Medical Research Co., Ltd

Address: Room 05-06, 7th Floor, Building 2, Area 2, No. 186, Nansihuan West Road, Fengtai District, Beijing, China

Name of Project Manager: Gubo Li

Position: Project Manager

Telephone: 15308008061

Signature of the Project Manager:
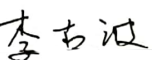


Date: 2022.3.22

**Program signature page**

**Statistician's statement**

I have carefully read and understood this clinical trial protocol and agree to the contents of the research protocol. I take responsibility for the data management and statistical analysis of this clinical trial protocol in accordance with the requirements of the Declaration of Helsinki, the Good Clinical Practice (GCP) guidelines (2020 edition), and relevant laws, regulations, and guidelines, and I will prepare the corresponding documents.

I agree to keep confidential all information related to this protocol that I receive or obtain during the trial.

**Statistical Analysis Unit:**  Beijing Bozhiyin Technology Co., Ltd.

Address: Room 2-2209, Shidai Fengfan Building, No. 15 Majia Boulevard West, Fengtai District, Beijing, China

Name of the Biostatistics Responsible Person: Hongxia Liu

Position: CEO

Telephone: 010-67587407

Signature of the Biostatistics Responsible Person:
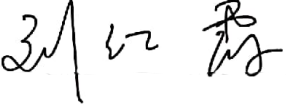


Date: 2022-3-22

Ethics Statement

1．Compliance with Documents

The trial must be conducted in accordance with the standard operating procedures of West China Hospital, Sichuan University, and must comply with the study protocol. The design of the study protocol follows the documents listed below:

1. 《Helsinki Declaration》(2013 Revision)
2. 《Drug Administration Law of the People's Republicof China》(2019 Revision)
3. 《Measures for the Administration of Drug Registration》(2020 Revision)
4. 《Good Clinical Practice》(2020 Revision)

2．Institutional Review Board and Ethics Committee

Before the trial begins, the investigator must submit the study protocol, informed consent form, approval documents from relevant authorities, drug testing reports, and any advertisements for recruiting subjects to the Institutional Review Board, Ethics Committee, or an organization with equivalent authority for approval. The written approval for the clinical trial should be submitted by the investigator to Chengdu Zenitar Biomedical Technology Co., Ltd.

3．Informed consent form

The informed consent form must be submitted to the relevant ethics committee for approval, and each informed consent form must include all pertinent information.

Before any potential participant engages in any activities related to the trial, the benefits and risks of the trial must be fully explained to them. After explaining the basic content of the trial and ensuring that each participant understands the purpose of the trial, each participant must be asked to sign their name and date on the informed consent form. The investigator must provide a copy of the informed consent form to the participant.

The investigator must explain the details of the clinical trial to the participants, including the following information:

1. Participation in the trial is voluntary, and participants have the right to withdraw from the trial at any stage without facing discrimination or retaliation; their medical treatment and rights will not be affected;
2. Participants must be informed that their participation in the trial and their personal data will be kept confidential. If necessary, the drug regulatory authorities, ethics committee, or sponsors may access the data of participants in accordance with regulations;
3. The purpose of the trial, the process and duration of the trial, the procedures involved, and the expected benefits and risks for participants;
4. Participants must be given adequate time to consider whether they wish to participate in the trial. The informed consent process should be conducted in language and terms that the participant or their legal representative can understand, and participants should have access to information related to them throughout the trial;
5. In the event of harm related to the trial, participants are entitled to receive treatment and appropriate compensation.

**Catalogs**

[Ethics Statement 9](#_Toc197889837)

[Abbreviations 6](#_Toc197889838)

[Protocol Synopsis 9](#_Toc197889839)

[General flowchart of the study 20](#_Toc197889840)

[PK and biomarker blood sample collection flowchart 24](#_Toc197889841)

[1. Introduction 26](#_Toc197889842)

[1.1. Background of the Development of Purinostat Mesylate for Injection 26](#_Toc197889843)

[1.2. Main Ingredients and Chemical Structure 34](#_Toc197889844)

[1.2.1. Drug Name 34](#_Toc197889845)

[1.2.2. The chemical structure, molecular formula, molecular weight, and basic physical and chemical properties of the active ingredient Purinostat Mesylate. 34](#_Toc197889846)

[1.2.3. Basic physicochemical properties 35](#_Toc197889847)

[1.3. Mechanism of action 35](#_Toc197889848)

[1.4. Preclinical studies 37](#_Toc197889849)

[1.4.1. Pharmacodynamic studies 37](#_Toc197889850)

[1.4.1.1. Study of Purinostat Mesylate inhibition of HDAC enzyme activity in vitro 37](#_Toc197889851)

[1.4.1.2. In vitro inhibitory activity of Purinostat Mesylate against various tumor cell lines derived from clinical patient samples 38](#_Toc197889852)

[1.4.1.3. In vivo pharmacodynamic evaluation of Purinostat Mesylate for injection 39](#_Toc197889853)

[1.4.2. Preclinical safety study 46](#_Toc197889854)

[1.4.2.1. Acute toxicity test in rodents (rats) 46](#_Toc197889855)

[1.4.2.2. Chronic toxicity test in rodents (rats) 47](#_Toc197889856)

[1.4.2.3. Acute toxicity test in non-rodent Beagle dogs 47](#_Toc197889857)

[1.4.2.4. Chronic toxicity test in non-rodent Beagle dogs administered intravenously. 48](#_Toc197889858)

[1.4.2.5. Safety Pharmacology Studies 48](#_Toc197889859)

[1.4.2.6. Special Safety Studies: Allergic, Hemolytic, and Local Irritation Tests 49](#_Toc197889860)

[1.4.2.7. Mutagenicity Test 50](#_Toc197889861)

[1.4.2.8. Embryo and Fetal Developmental Toxicity Test 52](#_Toc197889862)

[1.4.3. Pharmacokinetic Study 53](#_Toc197889863)

[1.4.3.1. Pharmacokinetics in Rodents 53](#_Toc197889864)

[1.4.3.2. Pharmacokinetics in Non-Rodent Animals 59](#_Toc197889865)

[1.4.3.3. In Vitro Metabolism Studies 61](#_Toc197889866)

[2. Purposes of the Study 63](#_Toc197889867)

[2.1. Primary Purposes 63](#_Toc197889868)

[2.2. Secondary Purposes 63](#_Toc197889869)

[3. Study Design 63](#_Toc197889870)

[3.1. Overall Description of the Study 63](#_Toc197889871)

[3.2. Dosing Regimen 64](#_Toc197889872)

[3.2.1. Considerations for the Starting Dose 64](#_Toc197889873)

[3.2.2. Considerations for Dosing Frequency 66](#_Toc197889874)

[3.2.3. Dose Group 67](#_Toc197889875)

[3.2.4. Escalation Process 68](#_Toc197889876)

[3.2.5. Individual Dose Adjustment 69](#_Toc197889877)

[3.3. Concomitant Medication 71](#_Toc197889878)

[3.3.1. Permitted Concomitant Medications 71](#_Toc197889879)

[3.3.2. Prohibited Concomitant Medications 72](#_Toc197889880)

[3.4. Definition of DLT 72](#_Toc197889881)

[3.5. Definition of MTD 73](#_Toc197889882)

[3.6. Dietary and Lifestyle Restrictions 73](#_Toc197889883)

[3.6.1. Diet 74](#_Toc197889884)

[3.6.2. Contraception 74](#_Toc197889885)

[4. Trial Population 74](#_Toc197889886)

[4.1. Sample Size 74](#_Toc197889887)

[4.2. Definition of the Trial Population 74](#_Toc197889888)

[4.3. Grouping 76](#_Toc197889889)

[4.4. Inclusion Criteria 76](#_Toc197889890)

[4.5. Exclusion Criteria 77](#_Toc197889891)

[4.6. Criteria for Withdrawal from the Study 78](#_Toc197889892)

[4.7. Subject Supplementation 79](#_Toc197889893)

[5. Investigational Drug 79](#_Toc197889894)

[5.1. Drug Introduction 79](#_Toc197889895)

[5.2. Drug Labeling 80](#_Toc197889896)

[5.3. Preparation Method 80](#_Toc197889897)

[5.4. Administration Method 81](#_Toc197889898)

[5.5. Drug Distribution and Storage 81](#_Toc197889899)

[5.6. Drug Transportation and Storage 81](#_Toc197889900)

[5.7. Drug Management 81](#_Toc197889901)

[6. Study Process 82](#_Toc197889902)

[6.1. Screening Phase（D-14 ~ D0） 82](#_Toc197889903)

[6.2. Single-Dose Administration Phase（D1-D7） 83](#_Toc197889904)

[6.3. Multiple-Dose Administration Phase（D8-D25） 85](#_Toc197889905)

[6.4. Extended Treatment Phase（D26-） 86](#_Toc197889906)

[6.5. End of Study/Early Withdrawal from the Trial 88](#_Toc197889907)

[7. Evaluation Indicators 89](#_Toc197889908)

[7.1. Tolerability and Safety Assessment 89](#_Toc197889909)

[7.2. Pharmacokinetics 90](#_Toc197889910)

[7.2.1. Pharmacokinetic Parameters 90](#_Toc197889911)

[7.2.2. Sample Collection Timing 90](#_Toc197889912)

[7.2.3. Sample Handling and Storage 91](#_Toc197889913)

[7.2.4. Sample Testing 91](#_Toc197889914)

[7.3. Biomarker Testing (Applicable Only to Leukemia Patients) 92](#_Toc197889915)

[7.3.1. Biomarker 92](#_Toc197889916)

[7.3.2. Sample Collection Timing 92](#_Toc197889917)

[7.3.3. Sample Handling, Testing, and Storage 92](#_Toc197889918)

[7.4. Sampling Time Window 93](#_Toc197889919)

[7.5. Efficacy Evaluation 93](#_Toc197889920)

[T-cell Acute Lymphoblastic Leukemia 94](#_Toc197889921)

[Other Relapsed or Refractory Hematologic Malignancies 94](#_Toc197889922)

[8. Adverse Events and Serious Adverse Events 94](#_Toc197889923)

[8.1. Adverse Events 94](#_Toc197889924)

[8.1.1. Definition 94](#_Toc197889925)

[8.1.2. Assessment of Adverse Event Severity 95](#_Toc197889926)

[8.1.3. Assessment of the Relationship Between Adverse Events and Investigational Drug 95](#_Toc197889927)

[8.1.4. Recording, Management, and Follow-Up of Adverse Events 96](#_Toc197889928)

[8.2. Serious Adverse Events 97](#_Toc197889929)

[8.3. Anticipated/Unexpected Adverse Events 98](#_Toc197889930)

[8.3.1. Anticipated Adverse Event 98](#_Toc197889931)

[8.3.2. Unexpected Adverse Events 99](#_Toc197889932)

[8.4. Risk Control Plan 99](#_Toc197889933)

[9. Data Management 101](#_Toc197889934)

[9.1. EDC Data Management 102](#_Toc197889935)

[9.2. External Data Transfer 102](#_Toc197889936)

[9.3. Medical Coding 102](#_Toc197889937)

[10. Statistical Analysis 103](#_Toc197889938)

[10.1. Analysis Dataset 103](#_Toc197889939)

[10.2. Statistical Methods 103](#_Toc197889940)

[10.3. Baseline Characteristics of Participants 103](#_Toc197889941)

[10.4. Tolerability/Safety Analysis 104](#_Toc197889942)

[10.5. Pharmacokinetic Analysis 104](#_Toc197889943)

[10.6. Clinical Efficacy Analysis 104](#_Toc197889944)

[10.7. Efficacy Endpoint Analysis 105](#_Toc197889945)

[10.8. Statistical Software 105](#_Toc197889946)

[10.9. Interim analysis 105](#_Toc197889947)

[11. Trail management 105](#_Toc197889948)

[11.1. Modifications to the Study Protocol 105](#_Toc197889949)

[11.2. Completion of the Electronic Case Report Form (eCRF) 105](#_Toc197889950)

[11.3. Data Quality Assurance 106](#_Toc197889951)

[11.4. Record Retention 106](#_Toc197889952)

[11.5. Monitoring 106](#_Toc197889953)

[11.6. Audits and Inspections 107](#_Toc197889954)

[11.7. Study Completion/Termination 108](#_Toc197889955)

[11.7.1. Study Completion 108](#_Toc197889956)

[11.7.2. Study Termination 108](#_Toc197889957)

[11.8. Use and Publication of Information 108](#_Toc197889958)

[11.9. Responsibilities of All Parties 108](#_Toc197889959)

[12. Ethical Principles 109](#_Toc197889960)

[12.1. Responsibilities of the Investigator 109](#_Toc197889961)

[12.2. Approval of the Ethics Committee 109](#_Toc197889962)

[12.3. Informed Consent 109](#_Toc197889963)

[13. References 111](#_Toc197889964)

[14. Appendix 113](#_Toc197889965)

[14.1. ECOG Performance Status Scoring Criteria 113](#_Toc197889966)

[14.2. New York Heart Association (NYHA) Functional Classification 114](#_Toc197889967)

[14.3. Criteria for Mild Abnormal Laboratory Values 115](#_Toc197889968)

[14.4. Criteria for the Evaluation of Multiple Myeloma 116](#_Toc197889969)

[14.5. Lymphoma Efficacy Evaluation Criteria 117](#_Toc197889970)

[14.6. Efficacy Evaluation Criteria for B-cell Acute Leukemia and T-cell Acute Leukemia 119](#_Toc197889971)

[14.7. Contraception 120](#_Toc197889972)

[14.8. WHO Bleeding Severity Grading (Revised Version) 121](#_Toc197889973)

Abbreviations

| English Abbreviations | Full name in English |
| --- | --- |
| AE | Adverse Event |
| ALT | Alamine Aminotransferase |
| ALP | Alkaline Phosphatase |
| ANC | Absolute Neutrophil Count |
| APTT | Activated Partial Thromboplastin Time |
| AS | Allometric Scaling |
| AST | Aspartate Aminotransferase |
| AUC | Area Under the Curve |
| AUC_0~t_ | Area Under the Curve from Time 0 to t |
| AUC_0~∝_ | Area Under the Curve from Time 0 to  Infinity |
| Ac-H_3_ | Acetylated histone H_3_ |
| Ac-H2B | Acetylated histone H2B |
| BOR | Best Overall Response |
| BrW | Brain Weight |
| Ca^2+^ | Calcium |
| CCr | Creatinine Clearance Rate |
| CK-MB | Creatine Kinase Isoenzyme |
| CFDA | China Food and Drug Administration |
| CHOL | Cholesterol |
| CI | Confidence Interval |
| CL | Clearance |
| Cl^-^ | Chlorinum |
| C_max_ | Maximum Plasma Concentration |
| CPK | Creatine Phosphokinase |
| CR | Complete Response |
| CT | Computed Tomography |
| DCR | Disease Control Rate |
| DLT | Dose Limited Toxicity |
| EC | Ethic Committee |
| eCRF | electronic Case Report Form |
| ECG | Electrocardiograph |
| ECOG | Eastern Cooperative Oncology Group |
| LVEF | Left Ventricular Ejection Fractions |
| ESI | Electrospray Ionization Source |
| FSH | Follicle-stimulating hormone |
| GCP | Good Clinical Practice |
| GGT | Gamma-Glutamyl Transpeptidase |
| GLP | Good Laboratory Practice |
| HBcAb | Hepatitis B core Antibody |
| HBsAg | Hepatitis B surface Antigen |
| HBV | Hepatitis B Virus |
| HCV | Hepatitis C Virus |
| HDAC | Histone Deacetylase |
| α-HDBD | Alpha-Hydroxybutyric Dehydrogenase |
| HED | Human Equivalent Dose |
| HIV | Human Immunodeficiency Virus |
| HNSTD | Highest Non-severely Toxic Dose |
| iCa | Ionized Calcium |
| IFN-α | Interferon-α |
| IVIVE | in vitro in vivo extrapolation |
| K^+^ | Potassium |
| ke | Elimination Rate Constant |
| LVEF | Left Ventricular Ejection Fractions |
| LD_50_ | Lethal Dose 50 |
| LDH | Lactate Dehydrogenase |
| LOCF | Last Observation Carried Forward |
| Max | Maximum |
| Mean | Mean value |
| Med | Median |
| MedDRA | Medical Dictionary for Regulatory  Activities |
| Mg^2+^ | Magnesium |
| MLP | Maximum Life-span Potential |
| Min | Minimum |
| MRI | Magnetic Resonance Imaging |
| MRM | Multiple Reaction Monitoring |
| MRSD | Maximum Recommended Strating Dose |
| MRT | Mean Residence Time |
| MTD | Maximal Tolerance Dose |
| Na^+^ | Sodium |
| NCI CTCAE | National Cancer Institute Common Termi-nology Criteria for Adverse Events 4.03 |
| NOAEL | No Observed Adverse Effect Level |
| NYHA | New York Heart Association |
| ORR | Objective Response Rate |
| PD | Progressive Disease |
| PD | Pharmacodynamic |
| PFS | Progression-Free-Survival |
| PK | Pharmacokinetic |
| Pi | Phosphorus |
| PR | Partial Response |
| PT | Prothrombin Time |
| PM | Purinostat Mesylate for Injection |
| PBMC | Peripheral blood mononuclear cell |
| PDX | Patient derived xenograft |
| Q3 | 3/4 Quantile |
| ROE | Rule of Exponent |
| RTV | Relative Tumor Volume |
| SAE | Serious Adverse Event |
| SAS | Statistical Analysis System |
| SD | Stable Disease |
| SD | Standard Deviation |
| SF | Safety Factor |
| SS | Safety Set |
| SM | Safety Margin |
| TBIL | Total Bilirubin |
| tCa | Total Calcium |
| T_max_ | Peak Time of plasma concentration |
| TT | Thrombin Time |
| TUNEL | Terminal-deoxynucleoitidyl Transferase  Mediated Nick End Labeling |
| TV | Tumor Volume |
| ULN | Upper Limit Of Normal |
| UPLC-MS/MS | Ultra-performance Liquid Chromatography-tandem Mass Spectrometry |
| V | Volume |

# Protocol Synopsis

| **Name of study** | Purinostat Mesylate for Injection (PM) in Relapsed or Refractory Hematologic Tumors, Mainly B-cell Related Malignancies: A Single-Center, Dose-Escalation Phase I Study Evaluating Tolerance, Safety, Pharmacokinetics and Pharmacodynamics |
| --- | --- |
| **Version No. and Program No.** | Version 1.4, Program No. GZ2018-001-1.0 |
| **Applicants** | Chengdu Zenitar Biomedical Technology Co., Ltd / West China Hospital, Sichuan University |
| **Research center** | West China Hospital National Drug Clinical Trial Organization |
| **Experimental drug** | Purinostat Mesylate for Injection |
| **Indications** | Relapsed or refractory hematologic malignancies predominantly of B-cell origin, including but not limited to B-cell lymphoma, multiple myeloma, B-cell acute leukemia, T-cell lymphoma, and T-cell acute leukemia. |
| **Expected progress** | Starts November 2019; ends September 2022 |
| **Research purpose** | **Primary purpose**   - Observe the tolerability and safety of Purinostat Mesylate for Injection administered as a single or multiple intravenous infusions in patients with relapsedor refractory B-cell-related hematologic malignancies, including but not limited to B-cell lymphoma, multiple myeloma, B-cell acute leukemia, T-cell lymphoma, and T-cell acute leukemia, who have experienced disease progression after standard treatment or are unsuitable for standard therapy. - Observe the dose-limiting toxicity (DLT) of Purinostat Mesylate for Injection in patients with relapsedor refractory B-cell-related hematologic malignancies to determine the maximum tolerated dose (MTD), providing a basis for the Phase II clinical dosing regimen.   **secondary purpose**   - Evaluate the pharmacokinetic parameters of Purinostat Mesylate for Injection following single and multiple intravenous infusions in patients with relapsedor refractory B-cell-related hematologic malignancies. - Evaluate the pharmacodynamic indicators of Purinostat Mesylate for Injection following single and multiple intravenous infusions in patients with relapsedor refractory B-cell-related hematologic malignancies. - Preliminarily observe the efficacy of Purinostat Mesylate for Injection in treating patients with relapsedor refractory B-cell-related hematologic malignancies. |
| **Research design** | The trial is divided into three phases: (1) Single-Dose Phase: Eligible subjects will be admitted to the ward the night before the trial. On the following morning around 8:00 AM, they will receive an intravenous infusion of Purinostat Mesylate, completed within 30 minutes. After administration, subjects will be monitored for 6 days, with pharmacokinetic blood samples collected at predefined time points. (2) Multiple-Dose Phase (D8-D25): Subjects who complete the single-dose phase may enter the multiple-dose phase. During this phase, doses will be administered on D8, D11, and D15. Subjects will be monitored for the occurrence of dose-limiting toxicity (DLT) and to determine the maximum tolerated dose (MTD). Pharmacokinetic and pharmacodynamic blood samples will also be collected at predefined time points. Completion of the multiple-dose phase will constitute the primary treatment portion of the trial. (3) Extended Treatment Phase: After completing the multiple-dose phase, if subjects tolerate the treatment well and do not experience disease progression, and if the investigator determines that continued treatment could be beneficial, subjects may continue treatment. This will follow a cycle of 21 days, with treatments on D1, D4, D8, and D11 of each cycle. During the extension treatment phase, after completing each administration cycle, subjects will be evaluated by the investigator. If they are well-tolerated without disease progression, and based on the tolerability and pharmacokinetic results from other subjects who have completed treatment in higher dose groups without DLTs, they may escalate to a higher dose group to treat until disease progression or intolerable toxicity occurs. |
| **Research population** | Patients with relapsed or refractory hematologic malignancies, predominantly of B-cell origin, including but not limited to patients with B-cell lymphoma, multiple myeloma, B-cell acute leukemia, T-cell lymphoma, T-cell acute leukemia, whose disease has progressed, relapsed after treatment with standard regimens, or is unsuitable for standard regimens. |
| **Number of subjects planned to be enrolled** | It is expected that 21 to 42 patients with relapsed or refractory B-cell-related hematologic malignancies will be enrolled, including but not limited to patients with B-cell lymphoma, multiple myeloma, B-cell acute leukemia, T-cell lymphoma, and T-cell acute leukemia who have experienced disease progression, relapse, or are unsuitable for standard treatment after receiving standard therapies. |
| **Trial groups and drug dosage** | This study will adopt a modified Fibonacci method for dose escalation, with seven predefined trial dose groups:   \| **Group** \| **1** \| **2** \| **3** \| **4** \| **5** \| **6** \| **7** \| **…..** \| \| --- \| --- \| --- \| --- \| --- \| --- \| --- \| --- \| --- \| \| Incremental ratio \| Initial dose \| 100% \| 67% \| 50% \| 40% \| 33% \| 33% \| 33% \| \| Dose (mg/m^2^) \| 1.2 \| 2.4 \| 4.0 \| 6.0 \| 8.4 \| 11.2 \| 15 \| Previous dose×33% increment  （Maximum drug exposure < 36.88 mg/m^2^） \| \| Number of subjects \| 3-6 \| 3-6 \| 3-6 \| 3-6 \| 3-6 \| 3-6 \| 3-6 \| 3-6 \|   If the maximum dose defined in this protocol (15 mg/m²) does not reach the MTD, the decision to continue dose escalation will be based on pharmacokinetic (PK), pharmacodynamic (PD) indicators, and safety data. If PK or PD indicators suggest that systemic drug exposure has not yet reached saturation, dose escalation will proceed according to the modified Fibonacci method until the MTD is observed or the drug reaches sufficient exposure in the body (the maximum drug exposure should not exceed the exposure at the canine HNSTD dose, which corresponds to an equivalent human dose of 36.88 mg/m²). |
| **Preparation and method of administration** | **Preparation Method:** The drug is prepared at room temperature. Before administration, each vial of PM (specification: 20 mg/vial) is reconstituted and diluted with sterile normal saline to an appropriate concentration until fully dissolved. After preparation, it must be administered via intravenous infusion over 30 minutes.  **Administration Method:** The drug is administered using an infusion pump through a peripheral vein, with an infusion volume of 50 mL and an infusion time of 30 minutes. The actual infusion time will be recorded. During the clinical trial, adjustments may be made to the drug concentration, infusion rate, infusion time, and infusion volume based on individual subject safety and tolerability. |
| **Inclusion criteria** | 1. Age 18 to 70 years, no restriction on gender; 2. Diagnosed through histopathology or cytology (according to the "Diagnosis and Efficacy Criteria for Hematologic Diseases" (4th Edition) (Scientific Publishing House)) as hematologic malignancies, including but not limited to B-cell lymphoma, multiple myeloma, B-cell acute leukemia, T-cell lymphoma, and T-cell acute leukemia. Patients must have experienced disease progression, relapse after receiving standard treatment, or be unsuitable for standard treatment (the specific definition of relapse or refractory disease can be found in Section 4.2). 3. No severe organic dysfunction in the heart, lungs, liver, or kidneys (LVEF (Left Ventricular Ejection Fraction)≥50%; total bilirubin ≤ 1.5×ULN; alanine aminotransferase (ALT) ≤1.5×ULN; aspartate aminotransferase (AST) ≤1.5 ×ULN; serum creatinine≤1.5 ×ULN or CCr> 40 mL/min). 4. No severe coagulation dysfunction (PT ≤1.5×ULN, APTT≤1.5× ULN, TT ≤ 1.5×ULN, and FIB≥1.0 g/L) 5. No severe hematologic abnormalities (absolute neutrophil count≥1.5×10^9^/L, platelets ≥ 75×10^9^/L, hemoglobin≥80 g/L), and no platelet, red blood cell transfusions within the 2 weeks prior to screening. 6. Patients must have received their last antitumor treatment (chemotherapy, radiotherapy, biologic therapy, or immunotherapy) at least 4 weeks or more than 5 half-lives prior to enrollment. 7. Expected life ≥ 12 weeks; 8. ECOG score≤2； 9. Those who agree to participate in the study and sign the informed consent form. |
| **Exclusion criteria** | 1. Toxicity from previous anticancer treatment has not recovered to Grade I or below, or the patient has not fully recovered from prior surgery; 2. Patients with severe heart, lung, liver, kidney, or gastrointestinal diseases and chronic diseases of major organs; 3. Female patients who are pregnant or breastfeeding, and female/male patients of childbearing potential who refuse to use contraceptive measures during the trial; 4. A history of acute myocardial infarction, congestive heart failure classified as NYHA Class II or higher, unstable angina, or stroke within the 6 months prior to enrollment; 5. Patients with impaired cardiac function (echocardiographic ejection fraction < 45% or complete left bundle branch block with ST segment depression > 1 mm in two or more leads or T wave inversion; congenital ventricular or atrial arrhythmias; clinically significant tachycardia (> 100 beats/min); bradycardia (< 50 beats/min); QTc > 450 ms (males), QTc > 480 ms (females); or clinically significant heart disease (such as unstable angina, congestive heart failure, or a history of myocardial infarction within the last 6 months)); 6. Patients with central nervous system lymphoma/leukemia or psychiatric disorders; 7. A history of organ transplantation; 8. Patients with severe active infections; 9. Known severe allergy to the investigational drug and its excipients or to HDAC inhibitors; 10. Positive for HCV antigen or antibodies, positive for HIV antigen or antibodies, positive for HBsAg, positive for HBcAb, and peripheral blood HBV DNA titer ≥ 1×10³ IU/mL; 11. Individuals with alcohol dependence or substance abuse; 12. Individuals who have participated in another clinical trial of a drug within the past month; 13. Any other condition which, in the opinion of the investigator, makes the patient unsuitable for study participation. |
| **Study Withdrawal Criteria** | Withdrawal refers to a subject no longer receiving the investigational drug treatment in this study (early termination of investigational drug treatment). The circumstances under which a subject withdraws from the study are classified into the following two categories:  **Subject-Initiated Withdrawal**  Subjects may terminate the investigational drug treatment or withdraw from the study at any time. Reasons for withdrawal from the study may include:  –The subject withdraws informed consent;  –The subject is lost to follow-up or has died.   - **Investigator-Initiated Withdrawal** - The investigator may also terminate a subject's participation in the study at any time, based on their professional judgment. - There is evidence of disease progression; - The subject experiences intolerable toxicity leading to drug discontinuation, and recovery has not occurred within 14 days after stopping the drug; - Poor subject compliance, making them unsuitable to continue in the study; - Major protocol deviations that affect drug tolerance and safety evaluation; - The subject is lost to follow-up; - Pregnancy; - Other reasons deemed by the investigator that make the subject unsuitable to continue in the trial. |
| **Drug Administration Plan** | The dosing phase of this study includes three stages: the single-dose phase, the multiple-dose phase, and the extended dosing phase. The pharmacokinetics of the single-dose and multiple-dose administrations are combined, during which safety, tolerability, and preliminary efficacy are observed.  **1. Single-Dose Phase**  The single-dose escalation phase will begin with the starting dose and proceed in an ascending order from low to high doses, with 3 to 6 subjects enrolled in each dose group. In each dose group, 3 subjects will be enrolled sequentially, and a single dose will be administered on Day 1 (D1) at that dose, followed by a 6-day observation period (D2-D7). Each subject will receive treatment with a single dose.  **2. Multiple-Dose Phase**  After completing the pharmacokinetic blood sampling for the single-dose administration, if the subject is well-tolerated, they will enter the multiple-dose phase, with doses administered on Days 8, 11, and 15. Each subject will receive the same dose as in the single-dose phase, with the dose-limiting toxicity (DLT) observation period defined as the first administration to 25 days after the first dose. Initially, only one subject will be enrolled in each dose group to observe their tolerability and safety within the 25 days following the first dose. Only if this subject does not experience a DLT will the remaining two subjects in the same dose group be allowed to enroll simultaneously.  If none of the 3 subjects in a dose group experience DLT, the trial will proceed to the next dose group. If 1 out of the 3 subjects in a dose group experiences DLT, an additional 3 subjects will be recruited to receive the same dose of the investigational drug, and they will be monitored for DLT occurrence.  If 1 DLT occurs among the 6 subjects, the trial will proceed to the next dose group. If 2 or more DLTs occur among the 3 subjects, or if 2 or more DLTs occur among the 6 subjects, the trial will be reduced to the previous dose group. If the previous dose group has fewer than 6 evaluable subjects, an additional 3 subjects will be enrolled to bring the total number of evaluable subjects to 6. If no more than 1 DLT occurs among the 6 evaluable subjects, the trial will be halted, and that dose group will be declared as the maximum tolerated dose (MTD). If the previous dose group has already reached 6 evaluable subjects, the trial will be halted, and that dose will be declared as the MTD.  During the multiple-dose escalation trial, real-time analysis of the pharmacokinetic (PK) data for the selected dose groups will be conducted to determine whether the drug reaches a saturation state in the body based on PK and pharmacodynamic (PD) indicators.  **3. Extended Treatment Phase**  After completing the single-dose and multiple-dose phases, if the efficacy assessment indicates clinical benefit for the subject, they will enter the extended dosing phase. In the extended dosing phase, treatment may continue according to the original dosing regimen or with adjustments to the dose based on the subject's tolerability and existing clinical results. Each dosing cycle lasts 21 days, with doses administered on Days 1, 4, 8, and 11 of each cycle. The number of extended dosing cycles will vary based on the patient's efficacy; subjects who achieve an objective response rate of partial response (PR) or higher after 6 cycles may continue receiving the treatment until disease progression, while those who do not reach PR will discontinue the treatment. Efficacy and safety data will continue to be collected during the extended dosing phase. |
| **PK blood collection points** | **Single-Dose Phase****:**  **The PK sampling points have been adjusted as follows based on the pharmacokinetic results and tolerability observed in the first patient**：  Blood samples of 3 mL will be collected from the antecubital vein of the forearm at the following time points: within 0.5 h before administration, and at 5 min, 10 min, 20 min, 30 min (immediately after administration), 32 min, 35 min, 45 min, 1 h, 2 h, 4 h, 8 h, and 12 h after administration, using an indwelling needle.  **Multiple-Dose Phase:**  D11：Blood samples were collected in 3 mL within 0.5 h prior to administration；  D15：Blood samples of 3 mL were collected 0.5 h before, 5 min, 10 min, 20 min, 30 min (immediately after the end of administration), 32 min, 35 min, 45 min, 1 h, 2 h, 4 h, 8 h, and 12 h, respectively. |
| **Biomarker blood collection points** | **Single-Dose Phase**：  Blood samples of 4 mL were collected within 0.5 h before, 1 h, 4 h, 24 h, and 48 h after the end of drug administration.；  **Multiple-Dose Phase:**  D8 Blood samples of 4 mL were collected within 0.5 h before, 1 h, 4 h, and 12 h after administration;  D11 Blood samples of 4 mL were collected within 0.5 h before and 1 h after administration;  D15 4 mL of blood samples were collected within 0.5 h before, 1 h, 4 h, 24 h, 48h and 72 h after administration; |
| **Safety assessments** | - Dose limiting toxicity (DLT); - Maximum tolerated dose (MTD); - According to CTCAE version 5.0, the types, severity, incidence, timing, duration, and relationship to the investigational drug of adverse events; - Vital signs: including temperature, heart rate, respiratory rate, and blood pressure (systolic and diastolic); - Physical examination: including examination of the skin, mucous membranes, lymph nodes, head, neck, chest, abdomen, spine/extremities, and nervous system - Laboratory tests: - Complete blood count: red blood cell count, hemoglobin, platelet count, white blood cell count, neutrophil count, and lymphocyte count; - Urinalysis: urine protein, urine red blood cells, and urine white blood cells; - Blood biochemistry: blood creatinine, urea, total bilirubin, alanine aminotransferase (ALT), aspartate aminotransferase (AST), alkaline phosphatase, total protein, albumin, electrolytes (K^+^, Na^+^, Ca2^+^, Mg^2+^, Cl^-^), total cholesterol, low-density lipoprotein (LDL), high-density lipoprotein (HDL), creatine kinase (CK), creatine kinase isoenzyme (CK-MB), α-hydroxybutyrate dehydrogenase (α-HBDH), lactate dehydrogenase, and fasting blood glucose; - Coagulation: PT, APTT, TT, FIB; - 12-lead ECG: PR interval, QRS interval, QT interval, QTc interval; - Echocardiogram (this examination may be performed if symptoms such as precordial pain or palpitations occur, or if the investigator deems it necessary); - Imaging assessments (CT or PET-CT or whole-body X-ray); |
| **Pharmacokinetic evaluation** | Single-Dose Phase：T_max_, C_max_, AUC_(0-t)_, AUC_(0-∞)_, V_d_, Ke, t_1/2_, MRT, CL  Multiple-Dose Phase：T_max_, C_ss_min_, C_ss_max_, C_ss_av_, t_1/2_, CL, AUC_ss_ |
| **Biomarker** | The acetylation levels of histones H3 and H4 are to be measured exclusively in leukemia patients. |
| **Evaluation of efficacy** | Assess the clinical efficacy of the drug in subjects before treatment and after multiple doses according to the evaluation criteria for different hematological malignancies.   - Objective mitigation rate (ORR) - Progression free survival (PFS) - Disease Control Rate (DCR): This refers to the proportion of patients in whom the tumor has reduced in size or remained stable for a certain period of time, including cases of complete remission (CR), partial remission (PR), and stable disease (SD). |
| **Concomitant Medications** | The following medications are allowed during the screening and treatment periods:   - Supportive care for adverse events related to the tumor and the study, such as antimicrobial agents, antiemetics, antidiarrheals, antipyretics, antihistamines, analgesics, medications to elevate white blood cell counts, and medications to control elevated white blood cell counts (e.g., hydroxyurea), etc.；Corticosteroids: Prednisone<20 mg/day, or equivalent doses of other corticosteroids, for the treatment of conditions not related to this disease; if using prednisone > 20 mg/day, or equivalent doses of other corticosteroids, the duration of use should not exceed 4 days for the prevention or treatment of adverse events occurring during the trial (e.g., infusion reactions); - Others: Concurrent conditions such as hypertension, diabetes, etc., for which the investigator deems that the use of basic medications will not affect the observation of study endpoints, may be used concurrently.   Subjects are prohibited from using the following medications during the screening period and the study period:   - Other antitumor medications (chemotherapy, immunotherapy) other than the investigational drug; - Radiation Therapy; - Medications that prolong the QT interval; |
| **DLT definition** | According to NCI-CTCAE version 5.0, any of the following toxicities related to the study drug (including definitely related, probably related, or possibly related) that occur within 25 days after the first administration of the drug (during Phase 1 and Phase 2 of the trial) may be considered a dose-limiting toxicity (DLT):   1. Hematological toxicity;  - Grade 3 febrile neutropenia is defined as an absolute neutrophil count (ANC) of < 1.0×10^9^/L accompanied by fever, with a temperature > 38.3℃, or a temperature sustained at≥38.0℃for more than 1 hour; - Grade 4 neutropenia without fever lasting for ≥ 5 days; - Grade 3 thrombocytopenia (25×10^9^/L < platelet count≤50×10^9^/L) accompanied by bleeding classified as Grade 2 or higher according to the WHO bleeding classification (2017 revised edition), or Grade 4 thrombocytopenia lasting≥7 days that requires platelet transfusion treatment≥2 times (excluding patients with acute leukemia);  1. Any non-hematological toxicity of Grade 3 or higher, including but not limited to:  - Grade 3 or higher QT interval prolongation; - Hepatotoxicity: If the baseline levels of ALT, AST, or TBIL in the subjects are≤ Grade 1, then drug-induced Grade 3 toxicity will be considered a dose-limiting toxicity (DLT).   **Except for the following:**   - Grade 3 fatigue, weakness, fever; - Grade 4 infusion reaction; - Grade 3 controlled hypertension (systolic blood pressure≥160 mmHg, diastolic blood pressure≥100 mmHg); - Grade 3 nausea and vomiting lasting less than 72 hours, with adequate antiemetic medication and other supportive treatment; - Grade 3 diarrhea lasting less than 72 hours, with adequate antidiarrheal medication and other supportive treatment; - Grade 3-4 electrolyte abnormalities that improve to Grade 2 or lower within 72 hours; - Alopecia;  1. Acute leukemia patients  - Persistent pancytopenia lasting > 42 days, accompanied by bone marrow hypoplasia (bone marrow cells < 5%) and the absence of leukemic cells in peripheral blood and bone marrow, will be considered a dose-limiting toxicity (DLT) due to bone marrow suppression. Pancytopenia is defined as white blood cells < 4.0 × 10^9^/L, hemoglobin < 100 g/L, and platelets < 100 × 10^9^/L. - If the investigator cannot attribute any of the following events to a clearly identifiable cause (such as tumor progression, underlying or concurrent diseases, or concomitant medications), these events will be considered dose-limiting toxicities: hematological toxicities potentially related to the study drug, Grade 3 or higher non-hematological toxicities, with the following exceptions: - Alopecia - Grade 3 fatigue, weakness, anorexia, fever, or constipation - Grade 3 nausea, vomiting, or diarrhea that does not require nasogastric feeding, parenteral nutrition (TPN), or hospitalization - Infections, bleeding, or other expected direct complications caused by underlying acute leukemia  1. Other: Important medical events that the investigator believes cannot be ruled out as related to the study drug may be considered dose-limiting toxicities (DLT) after discussion with the sponsor. 2. Any Grade 5 adverse events. 3. Treatment interruption >14 days due to unresolved toxicity (excluding cases of acute leukemia where treatment is interrupted for more than 14 days due to hematological toxicity). |
| **MTD definition** | If no more than 1 case of dose-limiting toxicity (DLT) occurs among the 6 subjects within 25 days after the first dose, the highest dose that meets this condition will be defined as the maximum tolerated dose (MTD). If all planned dose groups have been completed and no DLTs are observed in the highest planned dose group, that dose will be considered the MTD. |
| **Statistical analysis** | The statistical analysis of this study will be conducted using SAS 9.4 (or a later version). The study results will be reported in the form of summary tables (or graphs) and listings, primarily using descriptive statistical methods. Numerical variables will be described using mean, standard deviation (SD), median (Med), first quartile (Q1), third quartile (Q3), minimum (Min), and maximum (Max). Categorical variables will be described using counts and percentages within each category.  The safety statistical analysis will include statistical assessments of the incidence of adverse events/adverse reactions, listings of adverse events, vital signs, laboratory tests, physical examination results, and electrocardiogram (ECG) findings.  Pharmacokinetic analysis will be conducted using the WinNonlin program with a standard non-compartmental model to calculate pharmacokinetic parameters of the drug, including T_max_, C_max_, AUC_0-t_, AUC_0-∞_, t_1/2_, MRT, CL, Ke, and V. Descriptive statistics will be used to report measurements such as mean, standard deviation, median, maximum, and minimum. In single-dose studies, an evaluation of the dose proportionality will be performed. In multiple-dose studies, the degree of drug accumulation will be assessed.  Efficacy evaluation analysis will include the assessment of PFS (progression-free survival), ORR (objective response rate), and DCR (disease control rate). |
| **Ethics-related information** | The PM human pharmacokinetic trial strictly adheres to the Declaration of Helsinki and the ethical guidelines for human medical research. The study protocol was implemented after being approved by the Medical Ethics Committee of West China Hospital, Sichuan University. |
| **Termination criteria** | Trial termination refers to the situation where the clinical trial has not been completed according to the protocol and is stopped prematurely. The primary purpose of terminating a trial is to protect the rights and interests of the participants, ensure the quality of the trial, and avoid unnecessary financial losses. Any premature termination of the clinical trial should be promptly communicated to all parties involved in the study. Reasons for terminating the trial include, but are not limited to:  1. The discovery of significant errors in the clinical trial protocol that make it difficult to evaluate the safety of the drug;  2. Considerations related to safety;  3. A request from the sponsor to terminate the trial (e.g., due to funding issues, management reasons, etc.);  4. An order from the National Medical Products Administration (NMPA) or the ethics committee to terminate the trial for any reason. |

**General flowchart of the study**

|  | **Screening Periods** | **Single administration phase（D1-D7）** | | | **Multiple administration phase（D8-D25）** | | | | **Extended Treatment Phase（D26-）** | | | **Termination / Early Withdrawal from the Trial** |
| --- | --- | --- | --- | --- | --- | --- | --- | --- | --- | --- | --- | --- |
|  | **D-14 - D0** | **D1** | **D2** | **D7** | **D8** | **D11** | **D15** | **D25** | **On the day of drug administration at the beginning of each cycle（D1）** | **On the day of drug administration at the end of each cycle（D11）** | **The last day of each cycle（D21）** |  |
| **Time window** |  |  |  | **±1D** | **±1D** | **±1D** | **±1D** | **±1D** | **±3D** | **±3D** | **±3D** | **±7D** |
| Signed informed consent | **√** |  |  |  |  |  |  |  |  |  |  |  |
| Histopathology or cytopathology | **√** |  |  |  |  |  |  |  |  |  |  |  |
| Demographic information | **√** |  |  |  |  |  |  |  |  |  |  |  |
| Past medical and therapeutic history | **√** |  |  |  |  |  |  |  |  |  |  |  |
| allergic history | **√** |  |  |  |  |  |  |  |  |  |  |  |
| Verification of criteria for admission | **√** |  |  |  |  |  |  |  |  |  |  |  |
| Test drug administration^a^ |  | **√** |  |  | **√**（Administered once on D8, D11, and D15） | | |  | **√**（Administered once on D1, D4, D8, and D11 of each cycle） | |  |  |
| PK collects blood samples |  | **√**（See the PK and biomarker blood sampling workflow diagram） | | | | | |  |  |  |  |  |
| Biomarker collection of blood samples |  | **√**（See the PK and biomarker blood sampling workflow diagram） | | | | | |  |  |  |  |  |
| Vital signs^b^ | **√** | **√**（Checked once at 0.5 hours before administration on D1, and at 0.5 hours, 2 hours, and 24 hours after administration, as well as once on D7） | | | **√**（On D8, D11, and D15, checked once within 0.5 hours before administration and once 2 hours after administration） | | | **√** | **√**（On D1, D4, D8, and D11 of each cycle, each will be checked once within 0.5 hours before administration and once 2 hours after administration） | | **√** | **√** |
| Physical examination | **√** |  | **√** | **√** |  | **√** | **√** | **√** | **√** | **√** | **√** | **√** |
| ECOG score | **√** |  |  |  |  |  |  |  | **√**（pre-administration） |  |  | **√** |
| Urine or blood pregnancy test | **√** |  |  |  |  |  |  |  |  |  |  | **√** |
| Routine blood test^c^ | **√** |  | **√** | **√** |  | **√** | **√** | **√** | **√**^#^ | **√** | **√** | **√** |
| Urine routine^d^ | **√** |  | **√** | **√** |  | **√** | **√** | **√** | **√**^#^ | **√** | **√** | **√** |
| Blood biochemistry^e^ | **√** |  | **√** | **√** |  | **√** | **√** | **√** | **√**^#^ | **√** | **√** | **√** |
| Coagulation^f^ | **√** |  | **√** | **√** |  | **√** | **√** | **√** | **√**^#^ | **√** | **√** | **√** |
| M protein^L^ | **√** |  |  |  |  |  |  | **√** | **√**（pre-administration）^m^ |  |  |  |
| Virological examination^g^ | **√** |  |  |  |  |  |  |  |  |  |  |  |
| 12-lead ECG ^h^ | **√** | **√**（Checked once before administration, and 2 hours, 6 hours after administration, as well as on D2 and D7） | | | √（Checked once 2 hours and 6 hours after administration on D8, and once before administration, as well as 2 hours and 6 hours after administration on D11 and D15, and once on D25） | | | | √（Checked once before administration on D1, D11, and D21, and once 2 hours after administration, with additional checks as deemed necessary by the investigator） | | | **√** |
| Radiographic examination^i^ | **√** |  |  |  |  |  |  | **√** |  |  | **√** | **√** |
| Bone marrow examination^n^ | **√** |  |  |  |  |  |  | **√** |  |  | **√** | **√** |
| Echocardiography^j^ | **√** | △ | △ | △ |  | △ | △ | △ | △ | △ | △ | △ |
| Efficacy evaluation^k^ |  |  |  |  |  |  |  | **√** |  |  | **√** | **√** |
| Compliance evaluation |  | **√** |  |  |  |  |  | **√** |  |  | **√** | **√** |
| Adverse events | **√** | **√** | **√** | **√** | **√** | **√** | **√** | **√** | **√** | **√** | **√** | **√** |
| Concomitant medication | **√** | **√** | **√** | **√** | **√** | **√** | **√** | **√** | **√** | **√** | **√** | **√** |

Note: **√** indicates mandatory items, and **△** indicates optional items based on specific circumstances.

The single-dose phase is from D1 to D7, and the multiple-dose phase is from D8 to D25. The DLT observation period is from D1 to D25. If the participant tolerates the treatment well, they may enter the continuation dosing phase on D26.

**BSA Calculation Formula: Dubois-Dubois formulas，BSA = (W ^0.425^ x H ^0.725^) x 0.007184，Weight (kg), Height (cm); The single and multiple doses are calculated based on the weight at the time of the first administration. In the continuation dosing phase, weight is measured again before each cycle. If the weight changes by 10% or more compared to the initial dosing weight, the dosage for that visit will be based on the actual weight. However, the dosage remains unchanged for each cycle.**

1. Intravenous drip test drug:

- Single administration phase: D1 was administered and observed until D7;
- Multiple administration phase: Administered once on D8, D11, and D15;
- Extended Treatment Phase: Administered once on D1, D4, D8, and D11 of each cycle;

1. Vital signs: including temperature, heart rate, respiratory rate, and blood pressure (systolic and diastolic).

- Single administration phase: Checked once within 0.5 hours before administration, and once at 0.5 hours, 2 hours, and 24 hours after administration;
- Multiple administration phase: On D8, D11, and D15, checked once within 0.5 hours before administration and once 2 hours after administration; checked once on D25;
- Extended Treatment Phase: D1, D4, D8, and D11 of each cycle were examined once within 0.5 h before and 2 h after administration;

1. Complete blood count: red blood cell count, hemoglobin, platelet count, white blood cell count, neutrophil count, lymphocyte count;
2. Urinalysis: urine protein, urine red blood cells, urine white blood cells;
3. Blood biochemistry: blood creatinine, urea, total bilirubin, alanine aminotransferase (ALT), aspartate aminotransferase (AST), alkaline phosphatase, total protein, albumin, electrolytes (K+, Na+, Ca2+, Mg2+, Cl-), total cholesterol, low-density lipoprotein (LDL), high-density lipoprotein (HDL), creatine kinase (CK), creatine kinase isoenzyme (CK-MB), α-hydroxybutyrate dehydrogenase (α-HBDH), lactate dehydrogenase, fasting blood glucose;
4. Coagulation: PT, APTT, TT, FIB;
5. Virology tests: HIV testing (antibodies, antigens), HCV testing (antibodies), HBV testing (HBsAg, HBsAb, HBcAb, HBeAg, HBeAb, HBV-DNA titer).
6. 12-lead ECG: Includes PR intervals, QRS intervals, QT intervals, QTc intervals，

Single administration phase: Checked once before administration on D1 (results from within 5 days prior to D1 may be accepted), and once 2 hours, 6 hours after administration, as well as on D2 and D7;Multiple administration phase: Checked once 2 hours and 6 hours after administration on D8, and once before administration, as well as 2 hours and 6 hours after administration on D11 and D15, and once on D25;Extended Treatment Phase: Checked once before administration on D1, D11, and D21, and once 2 hours after administration; additional checks may be added as deemed necessary by the investigator.

Imaging studies: CT, PET-CT, or whole-body X-ray examinations. Results from screenings conducted within 4 weeks prior do not need to be repeated. During the continuation dosing phase, imaging should be performed once every 2 dosing cycles. If the dosing phase is extended, imaging should occur before the next cycle after the 2nd, 4th, 6th, etc. dosing cycles, with the imaging methods used for evaluating efficacy remaining consistent with the baseline. The examination window is ±3 days. For subjects with leukemia, the primary efficacy evaluation is based on bone marrow smears, and the investigator will determine whether imaging studies are necessary based on clinical circumstances.

Whole-body X-rays include views of the skull, pelvis, femur, humerus, thoracic spine, lumbar spine, and cervical spine.

1. Echocardiography is not a routine examination. It should be performed if the subject experiences symptoms such as precordial pain or palpitations, or if deemed necessary by the investigator.
2. Efficacy assessment: During the continuation dosing phase, for subjects who first achieve disease remission (CR/PR) after the 2nd, 4th, 6th, etc. dosing cycles, efficacy confirmation should be conducted 4 weeks after the initial efficacy assessment.
3. M protein testing is conducted only for patients with multiple myeloma and includes: (1) serum protein electrophoresis (Albumin, Alpha 1, Alpha 2, Beta 1, Beta 2, Gamma, M-spike); (2) immunofixation electrophoresis (IgG, IgA, IgM, light chain types); (3) β2-MG; (4) quantitative serum immunoglobulins (serum IgG, serum IgA, serum IgM, serum kappa light chains, serum lambda light chains, and the kappa/lambda ratio);
4. M protein testing is conducted only at the start of each cycle beginning with the 2nd cycle during the extended dosing phase;
5. Bone marrow examination includes: results from examinations conducted within 4 weeks prior to screening do not need to be repeated. The procedures include: (1) classification of bone marrow cytology smears; (2) bone marrow biopsy with immunohistochemistry; (3) flow cytometry. For leukemia patients, the bone marrow examination is limited to bone marrow smears and flow cytometry;

- The frequency of bone marrow cytology smear classification, bone marrow biopsy with immunohistochemistry, and flow cytometry at the end of multiple dosing and during the extended treatment phase will be determined based on clinical needs.

1. The time window for vital sign checks after administration is±30min.

**PK and biomarker blood sample collection flowchart**

**Single administration phase（D1-D7）**

| Time point | D1 | | | | | | | | | | | | | D2 | D3 |
| --- | --- | --- | --- | --- | --- | --- | --- | --- | --- | --- | --- | --- | --- | --- | --- |
|  | pre-medication | post-drug administration | | | | | | | | | | | | | |
|  | Within 0.5h | 5min | 10min | 20min | 30min | 32min | 35min | 45min | 1h | 2h | 4h | 8h | 12h | 24h | 48h |
| PK collects blood samples | **√** | **√** | **√** | **√** | **√** | **√** | **√** | **√** | **√** | **√** | **√** | **√** | **√** |  |  |
| Biomarker collection of blood samples * | **√** |  |  |  |  |  |  |  | **√** |  | **√** |  |  | **√** | **√** |

Note: At the same time point, if there are several examination or procedure items, the priority is as follows: PK blood sample collection > biomarker blood sample collection > vital sign checks > other items.

* Biomarker testing is conducted only for patients with leukemia

The pharmacokinetic blood collection time window is specified in section 7.2.2

**Multiple administration phase（First week，D8-D14）**

| Time point | D8 | | | | D9 | D11 | | | |
| --- | --- | --- | --- | --- | --- | --- | --- | --- | --- |
|  | pre-medication | post-drug administration | | | | pre-medication | post-drug administration | | |
|  | Within 0.5h | 1h | 2h | 4h | 24h | 0.5h内 | 1h | 2h | 4h |
| PK collects blood samples |  |  |  |  |  | **√** |  |  |  |
| Biomarker collection of blood samples * | **√** | **√** |  | **√** | **√** | **√** | **√** |  |  |

Note: At the same time point, if there are several examination or procedure items, the priority is as follows: PK blood sample collection > biomarker blood sample collection > vital sign checks > other items.

* Biomarker testing is conducted only for patients with leukemia

The pharmacokinetic blood collection time window is specified in section 7.2.2

**Multiple administration phase（2nd week，D15-D25）**

| Time point | D15 | | | | | | | | | | | | | D17 | D18 | | D19 | D25 |
| --- | --- | --- | --- | --- | --- | --- | --- | --- | --- | --- | --- | --- | --- | --- | --- | --- | --- | --- |
|  | pre-medication | post-drug administration | | | | | | | | | | | | | | | |  |
|  | Within 0.5h | 5min | 10min | 20min | 30min | 32min | 35min | 45min | 1h | 2h | 4h | 8h | 12h | 24h | | 48h | 72h |  |
| PK collects blood samples | **√** | **√** | **√** | **√** | **√** | **√** | **√** | **√** | **√** | **√** | **√** | **√** | **√** |  | |  |  |  |
| Biomarker collection of blood samples * | **√** |  |  |  |  |  |  |  | **√** |  | **√** |  |  | **√** | | **√** | **√** |  |

Note: At the same time point, if there are several examination or procedure items, the priority is as follows: PK blood sample collection > biomarker blood sample collection > vital sign checks > other items.

* Biomarker testing is conducted only for patients with leukemia

The pharmacokinetic blood collection time window is specified in section 7.2.2

# Introduction

## Background of the Development of Purinostat Mesylate for Injection

Hematologic malignancies include lymphoma, leukemia, and multiple myeloma. Among the various classifications of hematologic malignancies, with the exception of myeloid leukemia and T-cell or NK-cell derived lymphomas and leukemias, the other subtypes are classified as malignancies predominantly of B-cell origin. Malignancies predominantly of B-cell origin account for over 80% of the total proportion of hematologic malignancies. The global incidence and mortality data for hematologic malignancies in 2002, 2012, and 2015 are shown in Table 1^[1-3]^。It is evident that compared to 2012, the incidence and mortality rates of various hematologic malignancies surged in 2015. According to 2012 statistics, in developed countries, the number of new cases of hematologic malignancies has surpassed that of previously high-incidence liver cancer, and the gap in incidence with stomach cancer is also narrowing^[4]^。These facts indicate that with economic development, environmental changes, and shifts in human lifestyles (such as the adoption of healthy dietary habits and smoking control), along with advancements in clinical treatment methods, the incidence of certain solid tumors has been effectively controlled, and the treatment landscape has significantly improved. In contrast, the pathogenic factors for hematologic malignancies are more complex, and these diseases exhibit systemic and diffuse characteristics, making them inoperable. Therefore, hematologic malignancies are more reliant on chemotherapy.

Table 1 Epidemiological data on hematological malignancies for the years 2002, 2012, and 2015

|  | Global incidence rate ^*^ | | | | | |
| --- | --- | --- | --- | --- | --- | --- |
|  | Male | | | Female | | |
| Year | 2002 | 2012 | 2015 | 2002 | 2012 | 2015 |
| Lymphoma | 7.3 | 7.1 | 13.1 | 4.7 | 4.8 | 8.9 |
| Leukemia | 5.9 | 5.6 | 10.8 | 4.1 | 3.9 | 7.1 |
| Multiple Myeloma | 1.7 | 1.7 | 2.7 | 1.2 | 1.2 | 1.8 |
|  | Global incidence rate ^*^ | | | | | |
|  | Male | | | Female | | |
| Year | 2002 | 2012 | 2015 | 2002 | 2012 | 2015 |
| Lymphoma | 4.0 | 3.6 | 4.9 | 2.6 | 2.3 | 3 |
| Leukemia | 4.3 | 4.2 | 6.6 | 3.1 | 2.8 | 4.2 |
| Multiple Myeloma | 1.2 | 1.2 | 1.8 | 0.9 | 0.8 | 1.4 |

* Age-standardized rate (per 100,000 population)

Malignancies predominantly of B-cell origin include B-cell lymphoma, B-lymphocyte-associated leukemia, and multiple myeloma. Various subtypes of B-cell-related tumors originate from different stages of B-cell development and are often associated with gene translocations^[5]^，gene mutations (increases or deletions in gene copy number), and epigenetic abnormalities.^[6]^。The immunoglobulin heavy chain (IGH) enhancer located on chromosome 14 in B cells is crucial for normal physiological functions and remains in an activated state in mature B lymphocytes. However, this characteristic is often linked to the malignant transformation of B cells. In B-cell lymphoma, various oncogenes are commonly found to be juxtaposed with the IGH enhancer. For instance, follicular lymphoma is often caused by the translocation of the IGH enhancer to the BCL2 gene locus on chromosome 18 (t(14;18) (q32; q21)), leading to the overexpression of BCL2, a gene that encodes a protein that inhibits apoptosis. This translocation results in the spontaneous activation of the BCL2 gene, disrupting normal apoptotic pathways and leading to tumorigenesis.^[7]^;The translocation of the MYC gene to the IGH enhancer (t(8;14)(q24;q32)) is a molecular marker of Burkitt lymphoma. ^[8]^;One of the hallmarks of mantle cell lymphoma is the overexpression of Cyclin D1 caused by t(11;14) (q13; q32). ^[9]^。 In the most common subtype of B-cell lymphoma, diffuse large B-cell lymphoma, some cases show overexpression of the oncogene BCL6 due to gene rearrangements. ^[10]^。Some cases of B-cell lymphoma may exhibit either t (14;18) (q32; q21) or BCL6 gene rearrangement (or both), along with t (8;14) (q24; q32), which are newly classified as "double-hit" lymphomas (DHL). ^[11]^。Currently, there are no effective clinical treatment options for DHL.

In B-lymphocyte-associated leukemia, the common subtypes are B-cell acute lymphoblastic leukemia (ALL) and B-cell chronic lymphocytic leukemia (CLL). B-cell acute lymphoblastic leukemia originates from the malignant transformation of pre-B cells in the bone marrow, with common genetic abnormalities including the formation of the BCR-ABL1 fusion oncogene on chromosome 22 (the chromosome carrying the BCR-ABL fusion oncogene is also known as the Philadelphia chromosome), translocations or mutations of the RUNX1, PBX1, and MLL genes, and chromosomal amplifications ^[12]^. B-cell chronic lymphocytic leukemia, on the other hand, originates from mature B lymphocytes in the spleen or lymph nodes and is categorized into IgV gene mutated and unmutated types^[13]^，Due to its molecular characteristics being similar to those of lymphomas, B-cell chronic lymphocytic leukemia has been incorporated into the category of B-cell lymphomas according to current classifications.

Multiple Myeloma (MM) is a type of B-cell tumor that originates from malignant plasma cells in the bone marrow ^[14]^。Malignant plasma cells uncontrollably secrete mutated and immature immunoglobulins (M-proteins). The pathogenesis of multiple myeloma (MM) is driven by genetic abnormalities in the bone marrow microenvironment, such as mutations in oncogenes like MYC and IRF4, leading to the malignant proliferation of plasma cells. However, many specific mechanisms involved in this process remain unclear. ^[16]^。

Currently, chemotherapy is the primary treatment regimen for malignancies predominantly of B-cell origin, and some eligible patients may undergo stem cell transplantation after initial chemotherapy. The basic therapeutic agents for various B-cell lymphomas and BCR-ABL-negative B-cell acute lymphoblastic leukemia (ALL) include the monoclonal antibody rituximab, which targets the B-cell surface antigen CD20, along with several traditional chemotherapy agents. For BCR-ABL-positive B-cell acute lymphoblastic leukemia, treatment involves chemotherapy in combination with BCR-ABL kinase inhibitors such as dasatinib or imatinib. In recent years, targeted therapies have been developed based on the molecular characteristics of certain B-cell lymphoma subtypes, including the PI3K inhibitor idelalisib and the BTK inhibitor ibrutinib, which have been approved for use primarily in second-line treatment. The CAR-T therapy targeting CD19, axicabtagene ciloleucel, which is primarily aimed at diffuse large B-cell lymphoma (DLBCL), has also gained attention and was approved in 2017. The advent of CAR-T therapy has opened new avenues in cancer treatment, but its potential toxic side effects are still not fully understood, and it is characterized by high individualization and cost, making widespread adoption challenging in the short term. For cases of BCR-ABL-positive B-cell acute lymphoblastic leukemia that develop resistance mutations after treatment with first-generation kinase inhibitors dasatinib and imatinib, the novel inhibitor ponatinib can be used to target these mutations. In 2014, the first bispecific monoclonal antibody drug, blinatumomab, was approved for the treatment of acute lymphoblastic leukemia. Blinatumomab targets CD19 and CD3, with CD3 being a T-cell-specific antigen, allowing blinatumomab to also be used for treating T-cell leukemia. For multiple myeloma, the current commonly used clinical regimens include combinations of the immunomodulator lenalidomide, dexamethasone, and the proteasome inhibitor bortezomib, or lenalidomide, bortezomib, and the traditional chemotherapy agent cyclophosphamide. Eligible patients may also undergo stem cell transplantation after chemotherapy. In 2015, the HDAC inhibitor panobinostat (LBH589) was approved for use in combination with bortezomib and dexamethasone for the treatment of relapsed multiple myeloma, making panobinostat the first HDAC inhibitor approved for the treatment of this condition. Recent new drugs in the treatment of multiple myeloma include the novel immunomodulator pomalidomide, the new proteasome inhibitor ixazomib, the anti-CD38 monoclonal antibody daratumumab, and the anti-SLAMF7 monoclonal antibody elotuzumab. CAR-T therapy targeting BCMA, known as KITE-585, has recently emerged as a hot topic in the treatment of multiple myeloma and is currently undergoing phase I clinical trials.。

Despite the availability of therapies that show good treatment effects for certain subtypes of hematologic malignancies, some B-cell lymphomas, such as double-hit lymphoma (DHL) and Burkitt lymphoma (BL), remain resistant or have limited efficacy to existing drug treatments. Currently, the median survival for DHL is less than 12 months ^[11]^;The current clinical regimens for Burkitt lymphoma (BL) have significant toxic side effects, and the survival rate for elderly patients is extremely low ^[17]^。According to the NCCN guidelines, participation in clinical trials for new drugs is recommended for patients with double-hit lymphoma (DHL) and Burkitt lymphoma (BL), highlighting the urgent need for innovative therapies targeting these types of tumors. In terms of multiple myeloma treatment, due to issues such as intellectual property and drug pricing, patients in China are still limited to traditional chemotherapy agents, which have suboptimal efficacy and significant side effects. For BCR-ABL-positive B-cell acute leukemia, kinase inhibitors show good short-term efficacy and relatively low toxicity. However, when resistance mutations occur at the target site, the drugs become ineffective. The current clinical treatment landscape for T-cell-related tumors is also concerning. Therefore, there is a pressing need for the development of more effective and innovative drugs for the treatment of hematologic malignancies, particularly for certain refractory tumor subtypes.

In the field of cancer treatment, histone deacetylases (HDACs) are very important therapeutic targets. Purinostat Mesylate targets HDACs and is classified as a selective inhibitor of class I and IIb HDAC subtypes. The HDAC enzyme family consists of 11 subtypes, which are categorized into classes I, II, and IV^[18]^. Class I HDACs are primarily located in the nucleus and include HDAC1, HDAC2, HDAC3, and HDAC8. Class II HDACs are further divided into class IIa, which includes HDAC4, HDAC5, HDAC7, and HDAC9, and class IIb, which includes HDAC6 and HDAC10. Class IIa HDACs are found in both the nucleus and the cytoplasm, while class IIb HDACs are predominantly located in the cytoplasm. Class IV only includes HDAC11, which is also found in both the nucleus and the cytoplasm^[19]^. The function of HDACs is to catalyze the deacetylation of histone and certain non-histone lysine residues, thereby playing a crucial role in epigenetic and post-translational modifications within the cell^[20]^. Epigenetic abnormalities are one of the typical characteristics of tumors ^[21, 22]^，HDACs, primarily composed of class I enzymes, alter the cellular gene transcription profile through the deacetylation of histones, thereby achieving epigenetic regulation ^[23]^;Subtypes represented by class IIb HDACs regulate post-translational modifications through the deacetylation of certain cytoplasmic proteins, thereby affecting protein function and promoting effects such as tumor cell proliferation and migration ^[24, 25]^. The frequent overexpression or abnormal activity of HDAC enzymes in tumor cells leads to a loss of epigenetic control and dysregulation of important cytoplasmic functional proteins. These events can impact various cellular behaviors closely related to carcinogenesis, including the cell cycle, apoptosis, autophagy, migration, intracellular transport, and metabolism. Additionally, they can also induce alterations in the immune system at the organismal level^[26, 27]^. Targeted inhibition of HDACs in cancer therapy can reactivate tumor suppressor genes by restoring histone acetylation levels. Additionally, it can induce the acetylation of various cytoplasmic proteins, thereby inhibiting tumor cell migration, suppressing tumor cell proliferation signals, and promoting apoptosis in tumor cells ^[28]^. Moreover, HDAC inhibitors have the advantage of being more cytotoxic to cancer cells than to normal cells in the body^[29]^. Therefore, HDAC enzymes play a crucial role in the occurrence and development of tumors, making them important targets for drug development.

Currently, five HDAC inhibitors (HDACi) have been approved for clinical use, all indicated for hematologic malignancies, including lymphomas and multiple myeloma. Vorinostat (SAHA) has been approved for the treatment of cutaneous T-cell lymphoma (CTCL); Romidepsin is approved for the treatment of both CTCL and peripheral T-cell lymphoma (PTCL); in 2014, Belinostat was approved for the treatment of relapsed or refractory PTCL. Chidamide, as the first anti-tumor drug with independent intellectual property rights in China, was launched in December 2014 for the treatment of PTCL^[30]^. In February 2015, the FDA approved another HDAC inhibitor, LBH589 (panobinostat), developed by Novartis, for use in combination with bortezomib and dexamethasone for the treatment of relapsed and refractory multiple myeloma. In addition to the approved inhibitors, there are numerous HDAC inhibitors currently undergoing clinical evaluation, with indications covering a wide range of tumors, including lymphomas, leukemias, and lung cancer（www.clinicaltrial.gov）. In August 2014, the FDA granted orphan drug designation to Mocetinostat for the monotherapy treatment of diffuse large B-cell lymphoma (DLBCL), and its Phase II clinical trial targeting DLBCL has been completed^[31]^;The HDAC inhibitor Entinostat, developed by Syndax Pharmaceuticals, was granted breakthrough therapy designation by the FDA in September 2013 for use in combination with the aromatase inhibitor Exemestane for the treatment of metastatic estrogen receptor-positive (ER+) breast cancer in postmenopausal women as a second- or third-line therapy. Additionally, Entinostat has shown potential in the treatment of triple-negative breast cancer and non-small cell lung cancer ^[32, 33]^. In addition, a large number of HDAC inhibitors are currently undergoing various stages of clinical evaluation, with indications covering hematological malignancies, lymphomas, lung cancer, gliomas, prostate cancer, colorectal cancer, breast cancer, and melanoma. These inhibitors are administered either in combination with chemotherapy agents or as monotherapy. Among all reported HDAC inhibitors, LBH589 (panobinostat) is noted for its high activity and the broadest range of evaluated indications. LBH589 was approved by the FDA in 2015 for use in combination with bortezomib and dexamethasone for the treatment of relapsed and refractory multiple myeloma. Additionally, it is being investigated in multiple clinical trials for various subtypes of lymphomas, lung cancer, and other tumors. These studies demonstrate the significant value of targeting histone deacetylase inhibitors in both hematological and solid tumors. However, most existing HDAC inhibitors are pan-inhibitors, and their activity needs further improvement. Therefore, developing selective inhibitors and optimizing their activity may help reduce drug toxicity and enhance efficacy.

Purinostat Mesylate is a novel and structurally distinct highly selective inhibitor of Class I a and IIb HDACs. In vitro enzyme activity screening results demonstrate that Purinostat Mesylate exhibits high inhibitory activity against HDAC isoforms closely associated with tumors, including HDAC1, 2, 3, and 8, as well as Class II isoforms such as HDAC6 and 10. Its activity surpasses that of SAHA, Belinostat, and Mocetinostat, as well as Novartis' LBH589. Anti-proliferative activity screening across multiple tumor cell lines indicates that, similar to the control compound LBH589, Purinostat Mesylate shows IC_50_ values in the nM range for most hematological tumor cell lines, with some cell lines exhibiting values below 1 nM, demonstrating superior anti-tumor activity compared to LBH589. Further analysis of the cytotoxic activity of Purinostat Mesylate was conducted on several hematological tumor cell lines, measuring the IC_50_ values for proliferation inhibition and the LD_50_ values for cytotoxic activity after 72 hours of drug exposure. The results indicated that Purinostat Mesylate exhibited higher cytotoxic and anti-proliferative activity against hematological tumor cells compared to LBH589. A pharmacokinetic study and tumor tissue distribution experiments were performed using HCT116 tumor-bearing mice. The concentration of injected Purinostat Mesylate in plasma and tumor tissues, as well as the levels of histone acetylation at various time points, were measured. The experimental results showed that, despite a plasma elimination half-life (T_1/2_) of 1.21 hours, the average elimination half-life in tumor tissues was 45.34 hours. After 96 hours, the drug concentration remained at 68.95 ng/mL (approximately 0.14 µM), which is significantly higher than the IC_50_ values observed in vitro. The area under the curve (AUC) in tumor tissues was also much greater than that in plasma, with a tumor AUC_0-t_ / plasma AUC_0-t_ ratio of 12921.94 / 173.65 =74.41 times. Additionally, histone H4 acetylation levels in the tumors of solid tumor-bearing mice remained elevated following administration of Purinostat Mesylate, indicating that spaced dosing can maintain high activity levels. In multiple dosing groups, both tissue and plasma concentrations of Purinostat Mesylate were lower compared to the single-dose group, suggesting that Purinostat Mesylate does not accumulate in vivo, potentially avoiding toxicity associated with drug accumulation. Additionally, we investigated the pharmacokinetic characteristics and tissue distribution of Purinostat Mesylate in the Ighmyc model of primary B-cell lymphoma. The experimental results demonstrated that, after a single intravenous injection of 5 mg/kg of Purinostat Mesylate in Ighmyc mice, the elimination half-lives (T_1/2_) in plasma, spleen, and lymph nodes were 12.2 ± 2.10 hours, 20.8 ± 7.94 hours, and 21.9 ± 10.2 hours, respectively. The mean residence times (MRT) were recorded as 6.00 ± 1.00 hours for plasma, 24.8 ± 1.03 hours for the spleen, and 23.4 ± 3.59 hours for the lymph nodes. In comparison, wild-type mice receiving a single intravenous injection of 5 mg/kg of Purinostat Mesylate showed no detectable drug concentration in plasma after 24 hours. In contrast, the Ighmyc mice exhibited a plasma drug concentration of 7.11 ± 3.15 ng/mL at the same time point, indicating a significant difference in drug retention between the two mouse models. These results indicate that in primary B-cell lymphoma, the drug exhibits higher retention concentrations and longer retention times in plasma, as well as in the target tissues such as the spleen and lymph nodes, compared to normal mice. After a single intravenous injection of 5 mg/kg of Purinostat Mesylate, the concentrations in the spleen and lymph nodes of Ighmyc mice remained at 134 ± 18.5 ng/mL and 178 ± 46.0 ng/mL, respectively, after 48 hours, which is significantly higher than the in vitro IC_50_ values of the drug. Furthermore, the distribution concentrations of the drug in the spleen and lymph nodes were significantly higher than in other tissues, with minimal distribution in the brain and skeletal muscle. This indicates that in the B-cell lymphoma model, the concentration of Purinostat Mesylate in tumor cells and target tissues such as the spleen and lymph nodes is substantially greater than in other tissues, and the retention time in these tumor cells and target tissues is prolonged. These favorable pharmacokinetic characteristics support a dosing regimen of three times per week. Various molecular biology techniques combined with gene chip analysis confirmed that Purinostat Mesylate inhibits HDAC, reversing the cancerous phenotype through epigenetic intervention. It significantly suppresses the expression of key oncogenes associated with the MYC pathway, including cancer proteins IKZF1, IKZF3, and MYC, while downregulating the expression of MYC-related target genes. Additionally, Purinostat Mesylate induces cell cycle arrest at the G1 phase, causes DNA damage in tumor cells, and promotes tumor cell apoptosis.

The IC_50_ of this compound against hERG is greater than 100 μM, suggesting that it has low cardiac toxicity. In preclinical pharmacodynamics experiments, we established various B-cell lymphoma models, including a human-derived dual-hit lymphoma SUDHL-6 subcutaneous tumor model, human Burkitt lymphoma Daudi and Raji subcutaneous tumor models, multiple myeloma models PRMI-8226 and MMIS subcutaneous tumor models, an Ighmyc transgenic mouse model for spontaneous B-cell lymphoma, a mouse model for spontaneous B-cell acute leukemia, and a mouse xenograft model using samples from clinical B-cell acute leukemia patients. Clinical marketed drugs were used as positive controls for comparison with injectable Purinostat Mesylate. The experimental results indicated that Purinostat Mesylate at doses of 1.25, 2.5, 5, 10, and 20 mg/kg can dose-dependently inhibit tumor growth and significantly prolong survival.

Especially in the B-cell lymphoma models, including Daudi and Raji, as well as the multiple myeloma model PRMI-8226, a dose of 1.25 mg/kg is the effective dose for the drug, achieving a tumor inhibition rate of over 75%. At doses of 5 and 10 mg/kg, the inhibition rates exceed 90%, with the 10 mg/kg dose resulting in partial or complete tumor regression. Its antitumor activity is significantly superior to that of first-line drugs. In the human-derived double-hit lymphoma SUDHL-6 subcutaneous tumor model^[11, 34]^, doses of 2.5, 5, 10, and 20 mg/kg significantly inhibited tumor growth, with average inhibition rates of 58.99±2.32%, 69.35±3.47%, 75.81±8.28%, and 81.40±5.38%, respectively. In contrast, the first-line chemotherapy regimen HyperCVAD and the combination of HyperCVAD with monoclonal antibodies were ineffective. During the treatment process, there were no significant changes in body weight or general condition in the mice across all dosage groups, indicating that this compound maintains excellent antitumor activity while exhibiting low toxicity. In the human Burkitt lymphoma Daudi xenograft model, injectable Purinostat Mesylate at doses of 2.5, 5, and 10 mg/kg achieved inhibition rates of 82.84 ± 2.18%, 87.86 ± 2.38%, and 90.86 ± 3.84%, respectively. In comparison, the simulated clinical combination regimen of five chemotherapy drugs (CODOX group) resulted in only 51.33% ± 14.84% inhibition, and it exhibited significant toxicity, with incidents of mortality observed during the administration to the mice. In the human Burkitt lymphoma Daudi xenograft model, injectable Purinostat Mesylate at doses of 2.5, 5, and 10 mg/kg achieved inhibition rates of 82.84 ± 2.18%, 87.86 ± 2.38%, and 90.86 ± 3.84%, respectively. In contrast, the simulated clinical combination regimen of five chemotherapy drugs (CODOX group) only achieved an inhibition rate of 51.33% ± 14.84%, and it exhibited significant toxicity, with instances of mortality observed during administration to the mice. In the human Burkitt lymphoma Raji xenograft model, the inhibition rates for doses of 1.25, 2.5, 5, and 10 mg/kg were 52.03 ± 3.36%, 66.15 ± 12.49%, 81.65 ± 8.67%, and 89.98 ± 8.01%, respectively. The simulated clinical combination regimen of five chemotherapy drugs (CODOX group) achieved an inhibition rate of 67.15 ± 14.74%. Furthermore, in the human multiple myeloma RPMI-8226 xenograft model, the inhibition rates for doses of 1.25, 2.5, 5, and 10 mg/kg were 81.61%, 92.33%, 97.77%, and 97.77%, respectively. Injectable Purinostat Mesylate demonstrated superior efficacy compared to the clinical first-line regimen of lenalidomide + bortezomib + dexamethasone (with an inhibition rate of 89.39%), and also outperformed similar drugs such as LBH589 and the approved regimen of LBH589 + bortezomib + dexamethasone (with an inhibition rate of 87.49%). Additionally, injectable Purinostat Mesylate showed good therapeutic effects in various preclinical models, including the spontaneous B-cell lymphoma model induced in Ighmyc transgenic mice, the spontaneous B-cell acute lymphoblastic leukemia (B-ALL) model (resistant to imatinib and dasatinib), and patient-derived xenograft (PDX) models established from samples of clinical B-cell acute leukemia patients, significantly prolonging survival. These pharmacodynamic models support the proposed clinical indication of this compound for the treatment of hematological malignancies primarily associated with B-cell tumors.

## Main Ingredients and Chemical Structure

### Drug Name

Generic Name: Purinostat Mesylate for Injection

English name: Purinostat Mesylate for Injection

Hanyu Pinyin: Zhusheyong Jiahuangsuan Puyisita

The specification of this product is that each vial contains 20 mg of injectable Purinostat Mesylate, prepared as a sterile lyophilized product with appropriate excipients. It appears as a light yellow to off-white loose mass, packaged in a medium borosilicate glass lyophilization vial, protected from light and sealed. It should be stored in a cool, dark place. The shelf life is provisionally set at 24 months.

### The chemical structure, molecular formula, molecular weight, and basic physical and chemical properties of the active ingredient Purinostat Mesylate.

Figure 1 Chemical Structure of Purinostat Mesylate

Molecular formula: C_23_H_26_N_10_O_3_﹒CH_4_O_3_S

Molecular weight: 586.62

The chemical name of the active ingredient of the drug: 2-(((2-(4-Aminophenyl)-9-methyl-6-(4-morpholinyl)-9H-purine-8-yl)methyl)methylamino)-N-hydroxy-5-pyrimidine-carboxamide Mesylate (IUPAC)

English name: 2-(((2-(4-Aminophenyl)-9-methyl-6-(4-morpholinyl)-9H-purine-8-yl)methyl)methylamino)-N-hydroxy-5-pyrimidine-carboxamide Mesylate (IUPAC)

### Basic physicochemical properties

The API of this preparation is a dark yellow powder; Odorless.

**Solubility:** This product is soluble in dimethyl sulfoxide, slightly soluble in dimethylformamide, very slightly soluble in methanol, and practically insoluble in ethanol, ethyl acetate, or chloroform. It is very slightly soluble in 0.1 mol/L sodium hydroxide solution and practically insoluble in 0.1 mol/L hydrochloric acid solution.

**Absorption Coefficient:** Weigh approximately 20 mg of the product accurately and place it in a 50 mL volumetric flask. Dissolve it in methanol and dilute to the mark, shaking well. Accurately take 1 mL of this solution and transfer it to another 50 mL volumetric flask, diluting with 0.1 mol/L hydrochloric acid to the mark and shaking well. Measure the absorbance at a wavelength of 262 nm using the UV-Visible spectrophotometry method (as per the 2015 edition of the Pharmacopoeia of the People's Republic of China, General Rule 0401). The absorption coefficient （$E_{1cm}^{1\%}$） should be between 670 and 750, calculated on a dry basis.

## Mechanism of action

This product, Purinostat Mesylate, is a novel structure with a completely new skeleton and is an efficient selective inhibitor of Class I a and IIb HDACs. In vitro enzyme activity screening results show that Purinostat Mesylate exhibits high inhibitory activity against HDAC family members closely related to tumors, including HDAC subtypes 1, 2, 3, and 8, as well as Class IIb subtypes 6 and 10. Its activity is superior to that of SAHA, belinostat, and Mocetinostat, and it also outperforms Novartis LBH589. Results from anti-proliferation activity screening across multiple tumor cell lines indicate that, similar to the control compound LBH589, Purinostat Mesylate has IC_50_ values in the nM range for most blood cancer cell lines, with some cells showing values below 1 nM, demonstrating superior anti-tumor activity compared to LBH589. Further cytotoxicity analysis of the compound was conducted on certain blood cancer cell lines, measuring both the IC_50_ values for inhibition of proliferation after 72 hours of drug exposure and the LD_50_ values for cytotoxic activity. The results indicate that Purinostat Mesylate exhibits higher cytotoxic and anti-proliferative activity against blood cancer cells compared to LBH589. Through established pharmacokinetic and tumor tissue distribution studies in human xenograft HCT116 subcutaneous tumor mouse models, the concentrations of Purinostat Mesylate in plasma and tumor tissues, along with the levels of histone acetylation at various time points, were measured. The experimental results show that after a single dose of injectable Purinostat Mesylate, although the elimination half-life (T_1/2_) of the drug in plasma is 1.21 hours, the average elimination half-life in tumor tissue is 45.34 hours. Furthermore, 96 hours post-administration, the drug concentration remains at 68.95 ng/mL, approximately 0.14 µM, which is significantly higher than the IC_50_ value of Purinostat Mesylate in vitro. The area under the curve (AUC) in tumor tissue is also substantially greater than that in plasma, with a ratio of AUC_0-t_ (tumor) to AUC_0-t_ (plasma) of 12921.94 /173.65=74.41 times. In mice with solid tumors, the level of histone H4 acetylation in the tumor tissues remains elevated, indicating that interval dosing of injectable Purinostat Mesylate can maintain high activity. In multiple dosing groups, both tissue and plasma concentrations of Purinostat Mesylate are lower than in the single dosing group, suggesting that Purinostat Mesylate does not accumulate in vivo, thus avoiding toxicity associated with drug accumulation. Additionally, we investigated the pharmacokinetic characteristics and tissue distribution of Purinostat Mesylate in the plasma and target organs of the primary Ighmyc model B-cell lymphoma. The results indicated that after a single intravenous injection of 5 mg/kg of injectable Purinostat Mesylate in Ighmyc mice, the elimination half-lives (T_1/2_) of the drug in plasma, spleen, and lymph nodes were 12.2 ± 2.10 hours, 20.8 ± 7.94 hours, and 21.9 ± 10.2 hours, respectively. The mean residence times (MRT) were 6.00 ± 1.00 hours, 24.8 ± 1.03 hours, and 23.4 ± 3.59 hours, respectively. In comparison, after a single intravenous injection of 5 mg/kg of injectable Purinostat Mesylate in wild-type mice, no drug concentration was detected in the plasma at 24 hours. However, in Ighmyc mice, the plasma drug concentration at the same time point was 7.11 ± 3.15 ng/mL. These results indicate that in primary B-cell lymphoma, the drug retains higher concentrations and longer retention times in plasma, spleen, and lymph nodes, which contain a large number of tumor cells, compared to normal mice. After a single intravenous injection of 5 mg/kg of injectable Purinostat Mesylate, the concentrations of Purinostat Mesylate in the spleen and lymph nodes of Ighmyc mice at 48 hours reached 134 ± 18.5 ng/mL and 178 ± 46.0 ng/mL, respectively, which are significantly higher than the in vitro IC_50_ values of the drug. Furthermore, the distribution concentrations of the drug in the spleen and lymph nodes were markedly higher than in other tissues, with minimal distribution in the brain and skeletal muscle. This indicates that in the B-cell lymphoma model, the concentration of the drug in tumor cells and target tissues (spleen and lymph nodes) is significantly higher than that in other tissues, and the retention times in tumor cells and target tissues are prolonged. These favorable pharmacokinetic characteristics support a dosing regimen of three times per week. Using various molecular biology techniques combined with gene chip analysis, it was confirmed that Purinostat Mesylate inhibits HDAC, reverses the cancerous phenotype through epigenetic intervention, and significantly suppresses the oncogenic proteins IKZF1, IKZF3, and MYC in the MYC-related pathways associated with hematological malignancies. It also downregulates the expression of MYC-related target genes. Purinostat Mesylate induces cell cycle arrest in the G1 phase, induces DNA damage in tumor cells, and triggers apoptosis in tumor cells.

## Preclinical studies

### Pharmacodynamic studies

In accordance with the "Regulations on Drug Registration" and the "Guidelines for Non-Clinical Evaluation of Cytotoxic Antitumor Drugs," preclinical pharmacodynamic studies were conducted on the histone deacetylase (HDAC) inhibitor injectable Purinostat Mesylate (abbreviated as PM, with Purinostat Mesylate as the main component) for its primary indications of lymphoma, multiple myeloma, and acute B-cell leukemia. The main studies included: (1) an investigation of the HDAC enzyme inhibitory activity of Purinostat Mesylate in vitro; (2) an assessment of the effects of Purinostat Mesylate on the proliferation of various tumor cell lines in vitro; (3) the establishment of subcutaneous tumor models for three types of lymphoma, two types of multiple myeloma, a mouse B-cell lymphoma induced by Ighmyc transgenic mice, a primary mouse B-cell acute leukemia (B-ALL) model induced by the T315I mutant Bcr-Abl, and a patient-derived xenograft (PDX) model from clinical B-ALL samples to evaluate in vivo antitumor activity; (4) studies on the antitumor mechanisms of Purinostat Mesylate in multiple tumor cell line models. These studies provide experimental evidence for the clinical trial application of this drug for the indications of lymphoma, multiple myeloma, and acute B-cell leukemia.

#### Study of Purinostat Mesylate inhibition of HDAC enzyme activity in vitro

In vitro enzyme activity screening results showed that Purinostat Mesylate inhibits class I and class IIb HDACs, with the IC_50_ values for HDAC1, HDAC2, HDAC3, HDAC8, HDAC6, and HDAC10 being 0.81, 1.4, 1.7, 3.8, 11.5, and 1.1 nM, respectively. In contrast, it exhibited lower inhibitory activity against class IIa and class IV enzymes, with IC_50_ values for HDAC4, HDAC5, HDAC7, HDAC9, and HDAC11 being 1072, 426, 590, 622, and 3349 nM, respectively. The selectivity of Purinostat Mesylate for the class I HDACs (including HDAC1, 2, 3, and 8) and class IIb HDACs (including HDAC6 and 10) that are closely related to tumors is significantly superior to that of marketed drugs such as SAHA, Belinostat (PXD-101), and Chidamide, as well as to Mocetinostat, which has been granted "orphan drug" status by the FDA for the treatment of diffuse large B-cell lymphoma. This demonstrates a high selectivity for the tumor-associated class I and class IIb HDACs.

In experiments with the lymphoma and multiple myeloma cell lines SUDHL-6, Daudi, Raji, Ramos, and RPMI-8226, it was confirmed that concentrations of Purinostat Mesylate ranging from 0.3 to 1 nM significantly induced an increase in the acetylation levels of the class I HDAC substrates histone H3 and H4. Additionally, concentrations of 3 to 10 nM of Purinostat Mesylate significantly induced an increase in the acetylation levels of the class IIb HDAC substrate α-tubulin. The results of these cell experiments were consistent with the in vitro enzyme activity assay results.

The prototype drug Purinostat was tested for its inhibitory activity against 89 kinases that are strongly associated with tumors. The results showed that at a concentration of 1 μM, Purinostat exhibited no significant inhibitory activity against any of the screened 89 kinases, indicating that Purinostat is a highly selective inhibitor of class I and class IIb HDACs, with no other targets identified.

#### In vitro inhibitory activity of Purinostat Mesylate against various tumor cell lines derived from clinical patient samples

A total of 29 human cancer cell lines and human umbilical vein endothelial cells (HUVEC) were selected and cultured using standard cell culture methods. After treating the tumor cells with different concentrations of Purinostat Mesylate, the growth inhibition rates of the tumor cells were assessed using the MTT reduction method or the Alamar Blue assay. Dose-response curves were generated by plotting the growth inhibition rates against the different concentrations of the same sample to determine the half-maximal inhibitory concentration (IC_50_) of Purinostat Mesylate for various tumor cells. Additionally, using the Alamar Blue method, dose-response curves were created to assess both the growth inhibition rates and the cytotoxicity against tumor cells, allowing for the determination of IC_50_ values for cell proliferation inhibition and LD_50_ values for cytotoxicity. For the luciferase-labeled lymphoma cell lines Ramos and Raji (Ramos-luc and Raji-luc), the effects of different concentrations of Purinostat Mesylate on tumor cell viability were measured over 24 to 72 hours using a luciferase substrate assay.

The cell proliferation experiments with Purinostat Mesylate on various in vitro tumor cells indicate that it exhibits inhibitory effects on hematological tumor cells, with a half-maximal inhibitory concentration (IC_50_) of less than 10 nM, which is superior to that of the similar drug LBH589. Further analysis of the cytotoxic activity of the compound on certain hematological tumor cells revealed the IC_50_ values for proliferation inhibition and LD_50_ values for cytotoxic activity after 72 hours of drug treatment. The results showed that Purinostat Mesylate has higher cytotoxic and anti-proliferative activities against hematological tumor cells compared to LBH589.

In the luciferase-labeled cell experiments, the effects of Purinostat Mesylate on the Ramos-luc cell line were detectable 24 hours after treatment, while effects on the Raji-luc cell line were observed 36 hours post-treatment.

The results of the proliferation experiments with Purinostat Mesylate on various solid tumor cells showed that it has inhibitory effects on solid tumor cells, with an IC_50_ of less than 100 nM, and for most tumor cell lines, the IC_50_ is less than 50 nM, which is comparable to or better than LBH589. The IC_50_ value for normal HUVEC cells was 68.93 nM, indicating that the inhibitory effect of Purinostat Mesylate on normal HUVEC cells is less than that on most tumor cells.

In a clinical sample of BCR-ABL positive B-ALL, treatment with Purinostat Mesylate for 24 hours in vitro significantly induced apoptosis and inhibited the marker proteins Bcr-Abl, MYC, and phosphorylated Src. In a clinical sample of B-ALL carrying the T315I mutation in BCR-ABL, Purinostat Mesylate at a concentration of 20 nM for 24 hours in vitro notably induced apoptosis and inhibited the marker protein MYC. Additionally, in a clinical sample of lymphoblastic lymphoma, treatment with Purinostat Mesylate for 24 hours in vitro significantly induced apoptosis, with activity that was markedly superior to that of the similar drug LBH589. The study of samples from clinical tumor patients reveals that Purinostat Mesylate has the potential for treating the corresponding conditions.

#### In vivo pharmacodynamic evaluation of Purinostat Mesylate for injection

Based on the in vitro anti-tumor activity results of Purinostat Mesylate and referencing the preferred indications of similar drugs, we selected the most sensitive lymphoma cell lines, including SUDHL-6, Daudi, and Raji, as well as multiple myeloma cell lines RPMI-8226 and MM1S. Following the "Drug Registration Management Measures" and the "Guidelines for Non-Clinical Evaluation of Cytotoxic Antitumor Drugs," we established subcutaneous tumor models in NOD/SCID mice. Additionally, we created mouse models of B lymphoma or primary mouse B-ALL using transgenic mouse strains or molecular biology techniques. We also established patient-derived xenograft (PDX) models in NOD/SCID mice using samples from clinical B-ALL patients. The preclinical pharmacodynamic studies of injectable Purinostat Mesylate will focus on B-cell-related hematological tumors as the main indications, providing experimental evidence for clinical application submissions.

The test drug is injectable Purinostat Mesylate, provided by the State Key Laboratory of Biotherapy at Sichuan University. Subcutaneous or orthotopic tumor models were established using NOD/SCID mice for human-derived lymphoma, multiple myeloma cell lines, and samples from clinical B-ALL patients. The Ighmyc transgenic mice and primary B-ALL models were induced using C57 mouse strains. The experimental doses of Purinostat Mesylate include 1.25, 2.5, 5, 10, and 20 mg/kg, administered via tail vein (i.v.) three times a week. In each model experiment, the positive control drugs included the HyperCVAD regimen (cyclophosphamide, doxorubicin, vincristine, dexamethasone, methotrexate, and cytarabine) simulating clinical treatment for double-hit lymphoma, the CODOX regimen (cyclophosphamide, doxorubicin, vincristine, methotrexate, and cytarabine) simulating clinical treatment for Burkitt lymphoma, as well as lenalidomide (L), bortezomib (B), dexamethasone (D), and LBH589 lactate, which is marketed for relapsed or refractory multiple myeloma. In the subcutaneous tumor model experiments using human-derived tumor cell lines, the inhibitory effect of Purinostat Mesylate on tumors was assessed by measuring tumor volume (TV), tumor weight, relative tumor volume (RTV), and relative tumor growth rate (T/C). Additionally, in some model experiments, satellite groups were designed, where animals in these groups were not sacrificed at the same treatment endpoint and received extended treatment. The criteria for determining animal mortality included tumor growth reaching a predefined volume or animal death during the treatment process, allowing for the assessment of animal survival and the trend of tumor volume (TV) changes during the extended treatment period. This demonstrated the inhibitory effect of Purinostat Mesylate on tumors during the extended treatment phase. For the mouse B-cell lymphoma model induced by Ighmyc transgenic mice, the primary mouse B-ALL model, and the patient-derived xenograft (PDX) model from clinical B-ALL patients, the survival of the experimental animals was the primary indicator for evaluating the tumor treatment effect of Purinostat Mesylate.

1. **A subcutaneous tumor model of human double-blow lymphoma SUDHL-6**

In the two batches of repeated experiments using the human-derived double-hit lymphoma SUDHL-6 cell line subcutaneous tumor model in NOD/SCID mice, the average tumor inhibition rates for Purinostat Mesylate at doses of 2.5, 5, 10, and 20 mg/kg were 58.99±2.32%, 69.35±3.47%, 75.81±8.28%, and 81.40±5.38%, respectively. The relative tumor growth rates (T/C values) were 47.88±9.41%, 34.37±3.79%, 29.62±1.77%, and 21.28±6.72%, respectively. The average tumor inhibition rates for the four positive control groups (HyperCVAD six-drug combination, monoclonal antibody RTX, HyperCVAD + RTX, and LBH589) were 31.64%±2.20%, 20.16%±13.17%, 38.42%±7.28%, and 12.92%±13.85%, with average T/C values of 72.14±13.98%, 79.86±18.17%, 68.95±20.28%, and 92.55±13.13%. Purinostat Mesylate showed significant tumor inhibition effects starting from the low dose of 2.5 mg/kg, greatly outperforming the four positive control groups. In both batches of repeated experiments, the tumor-bearing animals in each Purinostat Mesylate group did not experience significant weight loss compared to the blank control group, and the animals remained in good condition throughout the entire experiment.

In the extended observation of satellite group animals from the first batch of experiments, Purinostat Mesylate at doses of 10 and 20 mg/kg demonstrated sustained inhibitory activity against subcutaneous tumor growth. The criteria for mortality in survival statistics were defined as reaching a subcutaneous tumor volume of 3000 mm³ or animal death during treatment. The median survival times for the blank control group and the solvent control group were both 14 days, while the median survival time for the positive control group (HyperCVAD) was 16 days. In contrast, the median survival times for the 10 and 20 mg/kg Purinostat Mesylate groups were both 30 days. The results from the extended observation in the first batch of experiments indicate that injectable Purinostat Mesylate has long-term inhibitory activity against human-derived double-hit lymphoma SUDHL-6 subcutaneous tumors, whereas the positive control HyperCVAD did not show any effect in delaying subcutaneous tumor growth during the extended observation.

In the first batch of experiments on SUDHL-6 subcutaneous tumors, histological analysis was performed on tumor tissues from some groups using HE staining, immunohistochemical analysis targeting the acetylation marker H4 (Ac-H4), the cell proliferation marker Ki67, and DNA damage markers, along with TUNEL staining. The HE staining results indicated that in the non-tumor internal necrotic areas, the blank control and solvent control groups displayed densely arranged tumor cells. There were no significant differences between the two positive control groups (LBH589 and HyperCVAD + RTX) and the blank or solvent controls. In contrast, the tumor tissues from the four dosage groups of Purinostat Mesylate exhibited a relatively loose arrangement of cells. Analysis of the acetylation marker Ac-H4 showed that all four doses of Purinostat Mesylate resulted in high levels of H4 acetylation, which was superior to the effects observed in the LBH589 group. The analysis of the cell proliferation marker Ki67 indicated that all four doses of Purinostat Mesylate inhibited Ki67 expression compared to the blank and solvent control groups, while the positive control groups (LBH589 and HyperCVAD + RTX) did not show a significant inhibitory effect on Ki67. The analysis of DNA damage markers demonstrated that all four doses of Purinostat Mesylate induced an upregulation of γH2AX levels compared to the blank and solvent control groups, with effects that were superior to those of LBH589. The TUNEL staining results revealed that the 10 mg/kg dose of Purinostat Mesylate significantly induced apoptosis in tumor tissues compared to the blank and solvent controls.

（**2）A Daudi subcutaneous tumor model of human Burkitt lymphoma**

In the two batches of repeated experiments using the human Burkitt lymphoma Daudi cell line NOD/SCID mouse subcutaneous tumor model, the tumor inhibition rates (mean values) for the groups treated with 2.5, 5, and 10 mg/kg of Purinostat Mesylate were 82.84±2.18%, 87.86±2.38%, and 90.86±3.84%, respectively. The relative tumor proliferation rates (T/C values, mean values) were 17.75±2.47%, 10.40±2.97%, and 7.35±3.75%. The positive control group CODOX had an average tumor inhibition rate of 51.33%±14.84% and an average T/C value of 40.95±13.36%.

Purinostat Mesylate demonstrated excellent therapeutic effects starting from the low dose of 2.5 mg/kg, significantly outperforming the five-drug combination positive control group CODOX. Throughout the treatment in both batches of repeated experiments, the body weight of tumor-bearing animals in each Purinostat Mesylate group did not show a significant decline compared to the blank control group, and the animals remained in good condition throughout the entire experiment. In contrast, the positive control group CODOX experienced individual deaths during the treatment in both batches of experiments, indicating that the CODOX group had greater toxicity and side effects. Thus, the efficacy and safety of Purinostat Mesylate were superior to those of CODOX.

In the extended study of the satellite group animals from two batches of repeated experiments, both the 5 mg/kg and 10 mg/kg doses of Purinostat Mesylate demonstrated sustained inhibitory activity against subcutaneous tumor growth. Using a tumor volume of 3000 mm³ or animal mortality during treatment as the criteria for survival statistics, the average median survival time for the blank control group across both batches was 19.5±2.1 days, while the solvent control group had an average median survival time of 20.0±1.4 days. The positive control group CODOX had an average median survival time of 26.0±2.8 days, whereas the average median survival times for the 5 mg/kg and 10 mg/kg groups of Purinostat Mesylate were 54.5±5.0 days and 58.5±2.1 days, respectively. In the first batch of experiments, a satellite group treated with Purinostat Mesylate at a dose of 20 mg/kg was also evaluated. This group paused treatment on day 28 and resumed on day 35. After a 7-day treatment interruption, the 20 mg/kg dose of Purinostat Mesylate continued to show tumor growth inhibition, with a median survival time of 54.4 days. The results from the extended study of both batches of repeated experiments indicate that injectable Purinostat Mesylate exhibits long-term inhibitory activity against human Burkitt lymphoma Daudi subcutaneous tumors, with its tumor suppression effects proving superior to those of CODOX.

**（3）Raji subcutaneous tumor model of human Burkitt lymphoma**

In the two batches of repeated experiments using the established human Burkitt lymphoma Raji cell line subcutaneous tumor model in NOD/SCID mice, the tumor inhibition rates (mean) for the groups treated with Purinostat Mesylate at doses of 1.25, 2.5, 5, and 10 mg/kg were 52.03±3.36%, 66.15±12.49%, 81.65±8.67%, and 89.98±8.01%, respectively. The relative tumor growth inhibition rates (T/C values, mean) were 48.13±5.13%, 31.86±8.56%, 18.11±7.50%, and 7.75±5.02%, respectively. The positive control group CODOX had an average tumor inhibition rate of 67.15±14.74% and an average T/C value of 34.77±18.77%. Purinostat Mesylate demonstrated excellent therapeutic effects starting from the low dose of 1.25 mg/kg, with the mid-dose of 2.5 mg/kg being close to the positive control group CODOX. During the treatment process in both batches of repeated experiments, the tumor-bearing animals in each dose group of Purinostat Mesylate did not exhibit significant weight loss compared to the blank control group, and the animals remained in good condition throughout the experiment. In contrast, both batches of the positive control CODOX group experienced instances of individual mortality during administration. This indicates that the five-drug regimen CODOX, simulating clinical medication, has greater toxic side effects, while Purinostat Mesylate shows superior efficacy and safety compared to CODOX.

In the extended survival investigation of the satellite group animals, 10 mg/kg of Purinostat Mesylate consistently demonstrated sustained inhibitory activity against subcutaneous tumor growth. Using a subcutaneous tumor volume reaching 3000 mm³ or animal mortality during treatment as the criteria for survival statistics, the median survival time for the blank control group was 14 days, the median survival time for the solvent control group was 16 days, and the median survival time for the positive control CODOX group was 32 days, while the median survival time for the 10 mg/kg Purinostat Mesylate group was 39 days. The results from the extended survival experiments indicate that injectable Purinostat Mesylate exhibits long-term inhibitory activity against human Burkitt lymphoma Daudi subcutaneous tumors, with its inhibitory effects being superior to those of CODOX.

**（4）Human multiple myeloma RPMI-8226 model**

In the experiment using the established human multiple myeloma RPMI8226 cell line subcutaneous tumor model in NOD/SCID mice, the tumor inhibition rates for the groups treated with Purinostat Mesylate at doses of 1.25, 2.5, 5, and 10 mg/kg were 81.61%, 92.33%, 97.77%, and 97.77%, respectively. The relative tumor growth inhibition rates (T/C values) were 13.9%, 6.6%, 2.7%, and 2.2%, respectively. The positive control group treated with LBH589 alone had a tumor inhibition rate of 59.79% and an average T/C value of 35.1%. The tumor inhibition rates for the positive control combination groups LBH589 + bortezomib + dexamethasone and lenalidomide + bortezomib + dexamethasone were 87.49% and 89.39%, with T/C values of 19.5% and 9.6%, respectively. Purinostat Mesylate demonstrated excellent therapeutic effects starting from the low dose of 1.25 mg/kg, surpassing the positive control LBH589 group. The 2.5 mg/kg group showed better efficacy than the positive control combination groups LBH589 + bortezomib + dexamethasone and lenalidomide + bortezomib + dexamethasone. Throughout the experiment, the tumor-bearing animals in each dose group of Purinostat Mesylate did not experience significant weight loss compared to the blank control group, and the animals remained in good condition during the experiment, indicating the efficacy and safety of Purinostat Mesylate are favorable. In contrast, one animal in the positive control combination group LBH589 + bortezomib + dexamethasone died, and two animals in the lenalidomide + bortezomib + dexamethasone group died.

In the RPMI-8226 model batch experiment, injectable Purinostat Mesylate (PM) was designed to be used in combination at a dose of 5 mg/kg, including PM + bortezomib + dexamethasone and PM + lenalidomide + dexamethasone. The tumor inhibition rate for the PM + bortezomib + dexamethasone group was 99.08%, with a T/C value of 1.3%. The tumor inhibition rate for the PM + lenalidomide + dexamethasone group was 98.59%, with a T/C value of 1.8%. Notably, in the combination groups, two mice had tumors that nearly completely regressed, and one mouse showed complete regression. In the PM + bortezomib + dexamethasone group, one animal experienced complete tumor regression, while another animal died. In the corresponding extended survival study for the satellite group animals, the median survival time for both the blank control group and the solvent control group was 32.0 days. The median survival time for the 5 mg/kg PM monotherapy group was 67.5 days, while the median survival time for the PM + bortezomib + dexamethasone group was 51 days. The lower survival rate in the combination group compared to the PM monotherapy group was attributed to increased toxicity, which led to one animal's death. The median survival time for the PM + lenalidomide + dexamethasone group was 74.5 days, whereas the median survival time for the positive control group treated with LBH589 alone was 52.5 days. The median survival times for the positive control combination groups, LBH589 + bortezomib + dexamethasone and lenalidomide + bortezomib + dexamethasone, were 52.5 days and 61.5 days, respectively. These results indicate that both PM + bortezomib + dexamethasone and PM + lenalidomide + dexamethasone possess superior antitumor activity. However, the PM + bortezomib + dexamethasone combination exhibited some toxicity, while the PM + lenalidomide + dexamethasone combination showed better efficacy in the extended survival assessment.

**（5）MM1S model of human multiple myeloma**

In the experiment using the established MM1S cell line subcutaneous tumor model in NOD/SCID mice, the tumor inhibition rates for the Purinostat Mesylate groups at doses of 2.5, 5, 10, and 20 mg/kg were 55.67%, 64.39%, 71.76%, and 76.44%, respectively. The relative tumor growth inhibition rates (T/C values) were 49.9%, 32.0%, 21.8%, and 18.3%, respectively. The positive control group treated with LBH589 had a tumor inhibition rate of 49.37% and an average T/C value of 45.1%. The combination treatment groups LBH589 + bortezomib + dexamethasone (B+D) and lenalidomide + bortezomib + dexamethasone (L+B+D) had tumor inhibition rates of 74.91% and 62.05%, with T/C values of 20.7% and 32.0%, respectively. Purinostat Mesylate demonstrated therapeutic effects starting from the low dose of 2.5 mg/kg, surpassing the positive control LBH589 group. Throughout the experiment, the tumor-bearing animals in each dose group of PM did not experience significant weight loss compared to the blank control group, and the animals remained in good condition throughout the study, indicating that the efficacy and safety of Purinostat Mesylate are favorable.

In the extended survival study for the satellite group animals, both the 5 mg/kg and 10 mg/kg doses of Purinostat Mesylate demonstrated sustained inhibitory activity against subcutaneous tumor growth. Using a criterion for mortality in survival statistics defined as either achieving a subcutaneous tumor volume of 1500 mm³ or the death of the animal during treatment, the median survival times for the blank control group and the solvent control group were both 16.0 days. In contrast, the median survival times for the PM groups at 5 mg/kg and 10 mg/kg were 29.0 days and 34.5 days, respectively. The results of the extended survival study indicate that Purinostat Mesylate exhibits long-term inhibitory activity against human multiple myeloma MM1S subcutaneous tumors, with its antitumor effect surpassing that of the positive control drug LBH589, which had a median survival time of 26.5 days.

**（6）Mouse B-cell lymphoma model induced by Ighmyc transgenic mice**

In the experiment using the mouse B-cell lymphoma model induced by Ighmyc transgenic mice, the median survival time for the solvent control group was 10.5 days, while the median survival times for the positive control groups were 20 days for LBH589 and 19.5 days for CODOX. The median survival times for the groups treated with 2.5, 5, and 10 mg/kg of Purinostat Mesylate were 19.5 days, 23 days, and 22.5 days, respectively. Compared to the solvent control group, the median survival times for the Purinostat Mesylate groups were significantly extended, with the 5 mg/kg and 10 mg/kg groups showing better therapeutic effects than the positive control groups LBH589 and CODOX. Regular blood sampling and analysis indicated that treatment with Purinostat Mesylate reduced the proportion of tumor cells in the peripheral blood of the mice in all groups.

**（7）Primary mouse B-ALL model induced by T315I mutant Bcr-Abl**

In the survival study of the primary mouse B-ALL model induced by the T315I mutant Bcr-Abl, the median survival time for the solvent control group was 44 days. The median survival times for the groups treated with 5 mg/kg and 10 mg/kg of Purinostat (Purinostat Mesylate) were both greater than 80 days. Compared to the solvent control group, the median survival times for the Purinostat-treated groups were significantly extended. Throughout the experiment, treatment with Purinostat resulted in a substantial reduction of tumor cell populations (GFP+, B220+) in the animals' blood.

**（8）PDX model of clinical B-ALL patients**

A clinical sample from a B-ALL patient was obtained, characterized by BCR-ABL positivity and relapse after first-line treatment. In the survival study of the PDX model established from this patient sample, the median survival time for the solvent control group was 21 days. The median survival times for the groups treated with 2.5 mg/kg and 10 mg/kg of Purinostat Mesylate were 66 days and 73 days, respectively, while the median survival time for the positive control drug LBH589 was 17 days. Compared to the solvent control group, the median survival times for the Purinostat Mesylate groups were significantly extended, whereas the positive control LBH589 was ineffective in this PDX model. Injectable Purinostat Mesylate shows potential for treating this type of condition.

### Preclinical safety study

The National Chengdu Center for Safety Evaluation of New Drugs conducted systematic studies on the injectable Purinostat Mesylate lyophilized powder in accordance with the relevant requirements for new drug registration as outlined in the "Regulations on the Registration of Drugs." These studies included preclinical assessments of acute toxicity, chronic toxicity, special safety (such as allergenicity, hemolysis, local irritation, etc.), genetic toxicity, and reproductive toxicity.

#### Acute toxicity test in rodents (rats)

The experimental design included low, medium, and high doses of 50, 100, and 200 mg/kg administered intravenously. In the medium and high-dose groups, symptoms observed included ear reddening, reduced activity, prone posture, rapid breathing, and deep gasping; in the high-dose group, additional signs included deep gasping, arched back, and weight loss. Six out of ten rats (2 females and 4 males) died between days 1 and 6 of the experiment, and there was a decrease in body weight and food intake. Hematological analysis showed that WBC and LYM counts were reduced in the low, medium, and high-dose groups, while EOS%, RET, and RET% were elevated, demonstrating a dose-dependent relationship. Histopathological findings indicated that in the high-dose group, deceased animals exhibited dark red changes in the lungs, mild to moderate pulmonary edema, slight to moderate hemorrhage, and severe inflammation. Surviving animals showed reduced testicular volume, dark red changes at the injection site, and moderate atrophy of the seminiferous tubules. The injection site exhibited moderate inflammation accompanied by moderate ulceration, leading to the identification of the maximum tolerated dose (MTD) as 100 mg/kg.

#### Chronic toxicity test in rodents (rats)

In this study, the low, medium, and high dose groups were set at 3, 10, and 30 mg/kg, respectively. The blank formulation was set at low and high doses of 45 and 450 mg/kg, corresponding to the doses of hydroxypropyl beta-cyclodextrin in the low and high dose groups of injectable Purinostat Mesylate. Dosing occurred once every two days, three times a week, for a total of four weeks, with some animals undergoing a four-week recovery period after the treatment ended. The main results indicated no mortality. In the high-dose group, symptoms included sparse fur, reduced activity, and scabbing at the injection site. Body weight and food intake were slightly decreased across all groups. In the treatment groups, WBC, PLT, and RET% were partially or fully reduced in a dose-dependent manner. Biochemical results showed elevated TBIL and partial or complete reductions in ALB, ALP, TP, and TG, also demonstrating a dose-dependent relationship. The primary toxic target organs or systems included the immune system (spleen, thymus, various lymph nodes), submandibular gland, bone marrow, testis, and epididymis. The lesions mentioned above showed a recovery period with near-complete restoration. Pharmacokinetics demonstrated linearity with minimal accumulation, and the highest no serious toxicity dose (HNSTD) was determined to be 30 mg/kg.

#### Acute toxicity test in non-rodent Beagle dogs

In this study, the designed doses were 10 and 50 mg/kg administered intravenously. The 10 mg/kg dose group exhibited vomiting and yellow mucous stools, which returned to normal the following day. In the 50 mg/kg dose group, symptoms included vomiting, tremors, reduced activity, decreased food intake, anorexia, and dark red stools, with all animals dying between days 2 and 4. Hematological analysis showed that WBC, RET, and RET% were reduced at both 10 and 50 mg/kg, while NEU and NEU% were elevated. Biochemical analysis revealed no abnormalities in the 10 mg/kg group, whereas in the 50 mg/kg group, AST, ALT, TBIL, CK, CREA, UREA, LDH, and LIPC levels were elevated. Histopathological examination showed no abnormalities upon gross necropsy. The maximum tolerated dose (MTD) was determined to be 10 mg/kg.

#### Chronic toxicity test in non-rodent Beagle dogs administered intravenously.

In this study, the low, medium, and high dose groups were set at 0.3, 1, and 3 mg/kg, respectively. The excipient was set at a high dose of 45 mg/kg. In the low dose group, occasional vomiting was observed; in the medium dose group, symptoms included vomiting, loose stools, and dark red stools; in the high dose group, symptoms included vomiting and dark red stools. Four dogs (4/10, 2 females and 2 males) died between days 13 and 26 of the experiment. Electrocardiogram (ECG) analysis showed that in the high dose group, male dogs exhibited increased heart rates and a slight prolongation of QTc (9.6%). Hematological analysis revealed that PLT, RET, and RET% were partially or fully reduced in the low, medium, and high dose groups, with an increase in APTT, indicating a dose-dependent relationship. Biochemical analysis showed that TP, TG, ALP, HDL, K+, and other parameters were partially or fully reduced in all dosing groups, also demonstrating a dose-dependent relationship. The primary toxic target organs or systems included the immune system (spleen, thymus, lymph nodes), digestive system (esophagus, liver, pancreas, intestine), kidneys, heart, lungs, trachea, testes, bone marrow, and injection site. There was a dose-dependent relationship, and recovery was observed during the recovery period. The highest no serious toxicity dose (HNSTD) was determined to be 1 mg/kg.

In conclusion, in the chronic toxicity study of injectable Purinostat Mesylate in dogs, the no observed adverse effect level (NOAEL) was found to be below 0.3 mg/kg, while the highest no serious toxicity dose (HNSTD) was 1 mg/kg.

#### Safety Pharmacology Studies

The safety pharmacology study indicated that Beagle dogs were administered intravenous injections of Purinostat Mesylate at doses of 0.4, 1.2, and 3.6 mg/kg. In the 1.2 and 3.6 mg/kg groups, transient increases in heart rate were observed, and in the 3.6 mg/kg group, a transient and reversible prolongation of QT interval (9.6%) was also noted. Additionally, there were no significant effects observed on other qualitative and quantitative ECG parameters or blood pressure in the dogs. In rats, intravenous administration of doses of 10, 30, and 90 mg/kg resulted in a decrease in body temperature at the 30 and 90 mg/kg doses. In the 90 mg/kg group, reduced locomotor activity and decreased standing frequency were also observed, but these effects returned to baseline after 24 hours. No significant effects were noted on other neurological behaviors in rats, and no impacts on the respiratory system were observed.

#### Special Safety Studies: Allergic, Hemolytic, and Local Irritation Tests

1. **Systemic Active Allergic Test in Guinea Pigs**

This experiment included a negative control group, a positive control group, a blank formulation group of injectable Purinostat Mesylate, and low and high dose groups of injectable Purinostat Mesylate, with 6 British guinea pigs in each group, evenly divided by sex. During the sensitization phase, the low and high dose groups received intravenous injections of 0.204 mg/mL and 0.612 mg/mL of injectable Purinostat Mesylate (1 and 3 times the maximum intended clinical concentration, respectively) at a volume of 0.5 mL per guinea pig. The blank formulation group received intravenous injections of the excipient in the same volume as the high dose group. The negative control group received an equal volume of 0.9% sodium chloride injection, while the positive control group received an intraperitoneal injection of 4 mg of ovalbumin solution per guinea pig. Sensitization was performed once every other day for a total of 3 consecutive sensitizations. On days 14 and 21 after the last sensitization, each group received an intravenous injection of 2 times the sensitizing dose to provoke an allergic response. Reactions and mortality were observed 30 minutes after the provocation injection.

Under the conditions of this experiment, the British guinea pigs that received intravenous injections of 0.204 mg/mL and 0.612 mg/mL of injectable Purinostat Mesylate (1 and 3 times the maximum intended clinical concentration, respectively) did not exhibit any allergic symptoms, resulting in a negative outcome for the systemic active allergic test.

1. **In Vitro Hemolytic Test**

This experiment included a negative control group (0.9% sodium chloride injection), a positive control group (sterile water for injection), a blank formulation group of injectable Purinostat Mesylate with five dosage levels (0.1, 0.2, 0.3, 0.4, and 0.5 mL/test tube at a concentration of 3.06 mg/mL, which is the same as the concentration of the excipient in the injectable Purinostat Mesylate group), and five dosage levels of injectable Purinostat Mesylate (0.1, 0.2, 0.3, 0.4, and 0.5 mL/test tube at a concentration of 0.204 mg/mL, the maximum intended clinical concentration). A 2% red blood cell suspension was mixed with 0.9% sodium chloride injection, sterile water for injection, the blank formulation of injectable Purinostat Mesylate, and injectable Purinostat Mesylate in the specified proportions, and then placed in a constant temperature incubator at 37±0.5°C. Hemolysis and aggregation were observed at 0, 15, 30, 45, 60, 120, and 180 minutes. Each group was measured in parallel three times.

The results showed that under the conditions of this experiment, the injectable Purinostat Mesylate at a concentration of 0.204 mg/mL (the maximum intended clinical concentration) did not cause hemolysis or aggregation in rabbit red blood cells, resulting in a negative outcome for the in vitro hemolytic test.

**（3）Intravenous Vascular Irritation Test in New Zealand White Rabbits**

This experiment included a blank formulation group of injectable Purinostat Mesylate and an injectable Purinostat Mesylate group, with 8 Japanese White Rabbits in each group, evenly divided by sex. The injectable Purinostat Mesylate group received an intravenous infusion of 0.204 mg/mL (the maximum intended clinical concentration) at a volume of 10 mL/kg via the right ear marginal vein. The blank formulation group received an intravenous infusion of hydroxypropyl beta-cyclodextrin at the same dose as the injectable Purinostat Mesylate group. Each rabbit in both groups also received an equal volume of 0.9% sodium chloride injection via the left ear marginal vein as a self-control. The administration was performed once daily for a total of 5 consecutive days, with the first day of administration defined as Day 1 of the experiment. During the study, the general condition of the rabbits and the injection site were monitored daily. Approximately 72 hours and 16 days after administration, 4 rabbits from each group were euthanized for gross anatomical observation of the injection site, and blood vessels and surrounding tissues from the injection site were collected for histopathological examination.

The results indicated that under the conditions of this experiment, the continuous intravenous injection of 0.204 mg/mL (the maximum intended clinical concentration) of injectable Purinostat Mesylate via the bilateral ear marginal veins in Japanese White Rabbits did not cause irritation at the injection site. Histological examination of the rabbit ear venous blood vessels showed normal vascular structure, with no endothelial damage, thrombus formation, or other pathological changes.

#### Mutagenicity Test

**（1）Microbial Reverse Mutation Test (Ames Test)**

In this experiment, two methods were selected: one with the S9 metabolic activation system and one without. The test strains used were histidine-dependent mutant strains of Salmonella typhimurium, specifically TA97, TA98, TA100, TA102, and TA1535. Under both conditions, with and without the S9 metabolic activation system, Purinostat Mesylate was tested at a maximum dose of 5000 μg/plate, along with four additional dose groups of 1500, 500, 150, and 50 μg/plate. A solvent control group (DMSO) and a positive control group (administering the appropriate positive agent) were also included, with each concentration tested in triplicate. The experiment was repeated once.

The results showed that under both conditions, with and without the S9 metabolic activation system, there was no observed antibacterial effect at any dose of Purinostat Mesylate for all test strains. Furthermore, under both conditions, there was no significant increase in the number of revertant colonies for any of the test strains at the various doses of Purinostat Mesylate. The results from both trials were consistent.

In summary, under the conditions of this experiment, neither the doses of 50, 150, 500, 1500, nor 5000 μg/plate of Purinostat Mesylate caused gene mutations in any of the test strains, indicating that the Ames test results were negative.

**（2）In Vitro Chromosomal Aberration Test in CHL Cells**

This experiment employed two methods: one without the S9 metabolic activation system and one with the S9 metabolic activation system. The method without the S9 metabolic activation system utilized two exposure times of 4 hours and 24 hours, while the method with the S9 metabolic activation system used a 4-hour exposure time. The test was conducted using a 2% dosing volume ratio. Under the 4-hour exposure conditions, the final concentrations of Purinostat Mesylate were set at 30, 100, 300, and 1000 µg/mL; under the 24-hour exposure conditions, the final concentrations were set at 3.75, 7.5, 15, and 30 µg/mL. Additionally, a solvent control group (DMSO) and a positive control group (administering the appropriate positive agent) were included. After the culture period for each concentration group, cells were collected and prepared for microscopy. After Giemsa staining, 1000 cells were observed under a microscope to record the number of cells in the metaphase stage and calculate the mitotic index inhibition rate for each group. For chromosome aberration assessment, 200 metaphase cells were observed, and the chromosome aberration rate was calculated for each group.

Under the conditions of this experiment, neither the concentrations of 30, 100, 300, nor 1000 µg/mL of Purinostat Mesylate caused a significant increase in the chromosome structural aberration rate in CHL cells after approximately 4 hours of exposure with or without the S9 metabolic activation system. Similarly, under the 24-hour exposure condition without the S9 metabolic activation system, the concentrations of 3.75, 7.5, 15, and 30 µg/mL of Purinostat Mesylate also did not lead to a significant increase in the chromosome structural aberration rate in CHL cells. Therefore, the results of the in vitro chromosomal aberration test in CHL cells for Purinostat Mesylate were negative.

**（3）Mouse Bone Marrow Micronucleus Test**

In this experiment, 90 SPF-grade NIH mice (half male and half female) were randomly divided into 7 groups: a negative control group (0.9% sodium chloride injection, intravenous injection, 10 mL/kg), an excipient control group (blank formulation, intravenous injection, 10 mL/kg), a positive control group (cyclophosphamide 40 mg/kg, intraperitoneal injection, 10 mL/kg), and four groups for intravenous injection of Purinostat Mesylate at low, medium, high, and highest doses (20, 40, 60, and 80 mg/kg, intravenous injection, 10 mL/kg). The negative control and positive control groups each consisted of 20 mice, while the other groups each consisted of 10 mice, with an equal number of males and females. Mice in each group were administered the specified doses once daily for 3 consecutive days, while the positive control group received only a single dose. Twenty to twenty-four hours after the last administration, all mice in each group were euthanized, and femoral bone marrow smears were prepared. The smears were stained with Giemsa and observed under an oil immersion microscope. For each mouse, 4000 polychromatic erythrocytes (PCEs) were counted to calculate the micronucleus incidence (‰), while 500 bone marrow erythrocytes were counted to determine the ratio of polychromatic erythrocytes to total erythrocytes.

During the experiment, a noticeable decrease in body weight was observed in both male and female mice in the 80 mg/kg group. Aside from this, mice in the other groups exhibited normal activity, good mental state, and clean fur, with no other toxic symptoms noted throughout the treatment period until euthanasia for sampling.

In the 80 mg/kg group, the ratio of polychromatic erythrocytes (PCEs) to total erythrocytes decreased, indicating bone marrow suppression. Among these, 4 male mice (4 out of 5) and 5 female mice (5 out of 5) had insufficient PCEs, with fewer than 4000 available for observation. The micronucleus rate among the observed PCEs did not show a significant increase.

In the 60 mg/kg group, the ratio of PCEs to total erythrocytes in male mice also decreased, indicating bone marrow suppression. However, in the 60 mg/kg female mice, as well as in the excipient control group and the 20 and 40 mg/kg groups of both male and female mice, no abnormal changes in the PCEs to total erythrocytes ratio were observed.

In the excipient control group and the 20, 40, and 60 mg/kg groups of both male and female mice, there was no significant increase in the micronucleus rate in the bone marrow.

In summary, under the conditions of this experiment, intravenous injection of Purinostat Mesylate at doses of 20, 40, 60, and 80 mg/kg, administered once daily for 3 consecutive days to NIH mice, did not cause any damage to the integrity of bone marrow cell chromosomes or lead to abnormalities in chromosome separation. The results of the bone marrow micronucleus test for Purinostat Mesylate were negative.

#### Embryo and Fetal Developmental Toxicity Test

Male SD rats were administered intravenous injections of Purinostat Mesylate at doses of 3, 10, and 20 mg/kg every two days from 4 weeks prior to mating until the end of mating. Female SD rats received the same treatment from 2 weeks prior to mating until day 7 of pregnancy. The no observed adverse effect level (NOAEL) for males was less than 3 mg/kg, while for females it was 20 mg/kg. The NOAEL for reproductive toxicity in both male and female rats was 3 mg/kg, and the NOAEL for early embryonic development was less than 3 mg/kg.

### Pharmacokinetic Study

#### Pharmacokinetics in Rodents

**（1）Pharmacokinetic Study in SD Rats**

After a single intravenous injection of Purinostat Mesylate at doses of 1, 3, and 10 mg/kg, the average initial concentrations (C0) in male and female SD rats were 736 ± 66.5, 2880 ± 566, and 11500 ± 1790 ng/mL, respectively. The plasma clearance rates (CL) were 301 ± 20.0, 205 ± 36.4, and 131 ± 12.8 mL/min/kg, respectively. The steady-state apparent volume of distribution (Vdss) was 1.43 ± 0.135, 1.13 ± 0.241, and 0.948 ± 0.125 L/kg, respectively. The half-lives (T1/2) were 3.79 ± 0.511, 4.21 ± 0.248, and 6.24 ± 1.57 min, respectively. The area under the curve (AUC_0-last_) values were 55.3 ± 3.68, 248 ± 36.1, and 1280 ± 122 ng.h/mL, respectively. After daily intravenous injections of 3 mg/kg Purinostat Mesylate for 7 consecutive days, the average AUC_0-last_ on day 7 for both male and female rats was 332 ± 43.6 ng.h/mL.

In the dose ranges of 1 to 3 mg/kg and 3 to 10 mg/kg, both C0 and AUC_0-last_ values in male and female rats increased proportionally with the dose. Across the dose range of 1 to 10 mg/kg, the C0 values increased proportionally with the dose, while the AUC_0-last_ values increased slightly more than proportionately. A tenfold increase in dose corresponded to a 21.7-fold and 24.4-fold increase in AUC_0-last_, respectively, for male and female rats.

At the doses of 1, 3, and 10 mg/kg, there were no significant gender differences in C_0_ and AUC_0-last_ values between male and female rats.

Following daily intravenous injections of 3 mg/kg for 7 consecutive days, there were no significant differences in AUC_0-last_ between days 1 and 7 for both male and female rats, indicating no abnormal accumulation.

**（2）Tissue Distribution and Mass Balance in SD Rats**

Using radiolabeled isotopes, a tissue distribution study was conducted in male and female SD rats following a single intravenous administration of [14C] Purinostat Mesylate at a dose of 3 mg/100 µCi/kg. The results indicated that the total radioactivity of Purinostat Mesylate quickly distributed from the blood to various tissues, demonstrating widespread distribution throughout the body, primarily in the lungs, liver, kidneys, and intestinal walls. At 0.167 hours post-administration, except for the ovaries and uterus (1549 ng Eq./g), gastric wall (1498 ng Eq./g), skin (1389 ng Eq./g), thymus (1359 ng Eq./g), skeletal muscle (1337 ng Eq./g), whole blood (1303 ng Eq./g), body fat (1067 ng Eq./g), testes and epididymis (312 ng Eq./g), and whole brain (99.1 ng Eq./g), where the total radioactivity was approximately equal to or lower than the plasma concentration at the same time point (1530 ng Eq./g), the total radioactivity in other tissues was significantly higher than the plasma concentration. The total radioactivity ranked from high to low was as follows: lungs (38170 ng Eq./g), liver (12220 ng Eq./g), kidneys (5739 ng Eq./g), intestinal wall (3862 ng Eq./g), heart (1917 ng Eq./g), spleen (1906 ng Eq./g), and mammary glands (1727 ng Eq./g), ranging from 1.13 times (mammary glands) to 24.9 times (lungs) the plasma concentration at the same time point. At 4 hours post-administration, the total radioactivity in the whole brain had fallen below the detection limit, while the total radioactivity in other tissues and plasma (44.9 ng Eq./g) decreased by 24.37% (intestinal wall) to 97.10% (skeletal muscle) compared to levels at 0.167 hours. At 24 hours post-administration, the total radioactivity in the whole brain, mammary glands, skin, heart, gastric wall, ovaries and uterus, whole blood, skeletal muscle, and body fat had all fallen below the detection limit. At the final collection time point (96 hours), only the spleen (1234 ng Eq./g), liver (670 ng Eq./g), lungs (603 ng Eq./g), kidneys (140 ng Eq./g), thymus (67.6 ng Eq./g), and heart (46.2 ng Eq./g) showed detectable low concentrations of total radioactivity, collectively accounting for less than 0.78% of the administered dose. The total radioactivity in other tissues had fallen below the detection limit. At the same time point, the drug content in the tissues was significantly higher than the plasma drug concentration.

Following a single intravenous administration of [14C] Purinostat Mesylate at a dose of 3 mg/100 µCi/kg in male and female rats, the excretion rate and amount of total radioactivity were found to be similar between genders. The total recovery rate over 0-168 hours was 98.49% of the administered dose, achieving a material balance of 98.49%, with the majority being excreted in feces, accounting for 83.15% of the administered dose. Excretion primarily occurred within the first 24 hours post-administration, approximately 81.38% of the total dose. In male and female BDC rats following a single intravenous administration, the total amount excreted over 0-72 hours was 95.54% of the administered dose, with the total amount excreted in bile accounting for 62.37% of the administered dose.

**（3）Metabolites and Pathways in SD Rats**

Following a single intravenous administration of [14C] Purinostat Mesylate at a dose of 3 mg/100 µCi/kg in male and female rats, a total of 14 metabolites were identified, in addition to the parent compound. These were designated as M382, M396, M474, M475, M490, M516, M517, M532, M546, M548, M558, M666, M708a, and M708b. Among these, M382 is an acetylated product of the parent drug after N-dealkylation, M396 is a carboxylic acid derivative of M382, M474 is a reduction product of the parent drug, M475 is a hydroxylated product derived from the amino conversion of M474, M490 is a mono-oxidation product of M474, M516 is an acetylated product of M474, M517 is a hydroxylated product derived from the amino conversion of M516, M532 is an acetylated product of the parent drug, M546 is a methylated product of M532, M548 is a dioxidation product of M516, M558 is a glucuronic acid conjugate of M382, M666 is a glucuronic acid conjugate of the parent drug, M708a is a mono-oxidation glucuronic acid conjugate of M516, and M708b is a glucuronic acid conjugate of M532.

**Metabolites in Plasma**

Following a single intravenous administration of [^14^C] Purinostat Mesylate at a dose of 3 mg/100 µCi/kg in male and female rats, the parent drug accounted for 11.15% of the AUC_0-96h_ in females and 8.79% in males. The main metabolites in plasma were M474, M516, and M708b, with M474 representing 13.75% of the AUC_0-96h_ in females and 13.79% in males, M516 accounting for 15.61% in females and 17.42% in males, and M708b comprising 10.04% in females and 8.18% in males. The secondary metabolites M382, M475, M532, and M546 accounted for 4.71% to 9.05% of the AUC_0-96h_ in females and 4.85% to 6.67% in males. Other metabolites contributed less than 3.50% to the AUC_0-96h_ in the rats.

**Metabolites in Bile**

Following a single intravenous administration of [14C] Purinostat Mesylate at a dose of 3 mg/100 µCi/kg in male and female rats, the total radioactivity excreted in bile from 0 to 72 hours was 64.01% of the administered dose in females and 60.72% in males. The parent drug accounted for 2.45% of the administered dose in females and 2.47% in males. Additionally, a total of 14 metabolites were identified in bile, with M516, M548, and M708b being the major metabolites, accounting for 5.77% to 7.49% of the administered dose in females and 4.75% to 8.74% in males. The secondary metabolites M382, M396, M474, M490, M517, M546, M558, M666, and M708a accounted for 1.16% to 3.30% of the administered dose in females and 0.97% to 4.38% in males. Other metabolites contributed less than 3.50% of the administered dose.

**Metabolites in Urine**

Following a single intravenous administration of [14C] Purinostat Mesylate at a dose of 3 mg/100 µCi/kg in male and female rats, the total radioactivity excreted in urine from 0 to 168 hours was 12.98% of the administered dose in females and 15.34% in males. The content of the parent drug in urine was low, accounting for 0.35% of the administered dose in females and 0.66% in males. Additionally, a total of 14 metabolites were identified in urine. Among these, M516 and M558 were the major metabolites, accounting for 2.35% to 2.49% of the administered dose in females and 0.88% to 3.01% in males. The secondary metabolites M474, M532, and M708b accounted for 0.43% to 0.87% of the administered dose in females and 1.02% to 1.92% in males. Other metabolites contributed less than 1.00% of the administered dose.

**Metabolites in Feces**

Following a single intravenous administration of [14C] Purinostat Mesylate at a dose of 3 mg/100 µCi/kg in male and female rats, the total radioactivity excreted in feces from 0 to 168 hours was 85.41% of the administered dose in females and 80.90% in males. The parent drug accounted for 0.84% of the administered dose in females and 0.94% in males. Additionally, a total of 14 metabolites were identified in feces, with M516 being the major metabolite, accounting for 30.10% of the administered dose in females and 27.70% in males. The secondary metabolites M382, M396, M474, M490, M517, M546, M548, M666, M708a, and M708b accounted for 0.79% to 5.53% of the administered dose in females and 1.27% to 6.00% in males. Other metabolites contributed less than 3.50% of the administered dose.

**Metabolic pathway**

Based on the identification of metabolites from [^14^C] Purinostat Mesylate, it is inferred that the main metabolic pathways of Purinostat Mesylate in SD rats are: 1) reduction, 2) oxidative deamination, and 3) N-dealkylation; as well as phase II reactions including methylation, acetylation, and glucuronidation. The proposed major metabolic pathways of Purinostat Mesylate in SD rats are illustrated in the figure below:


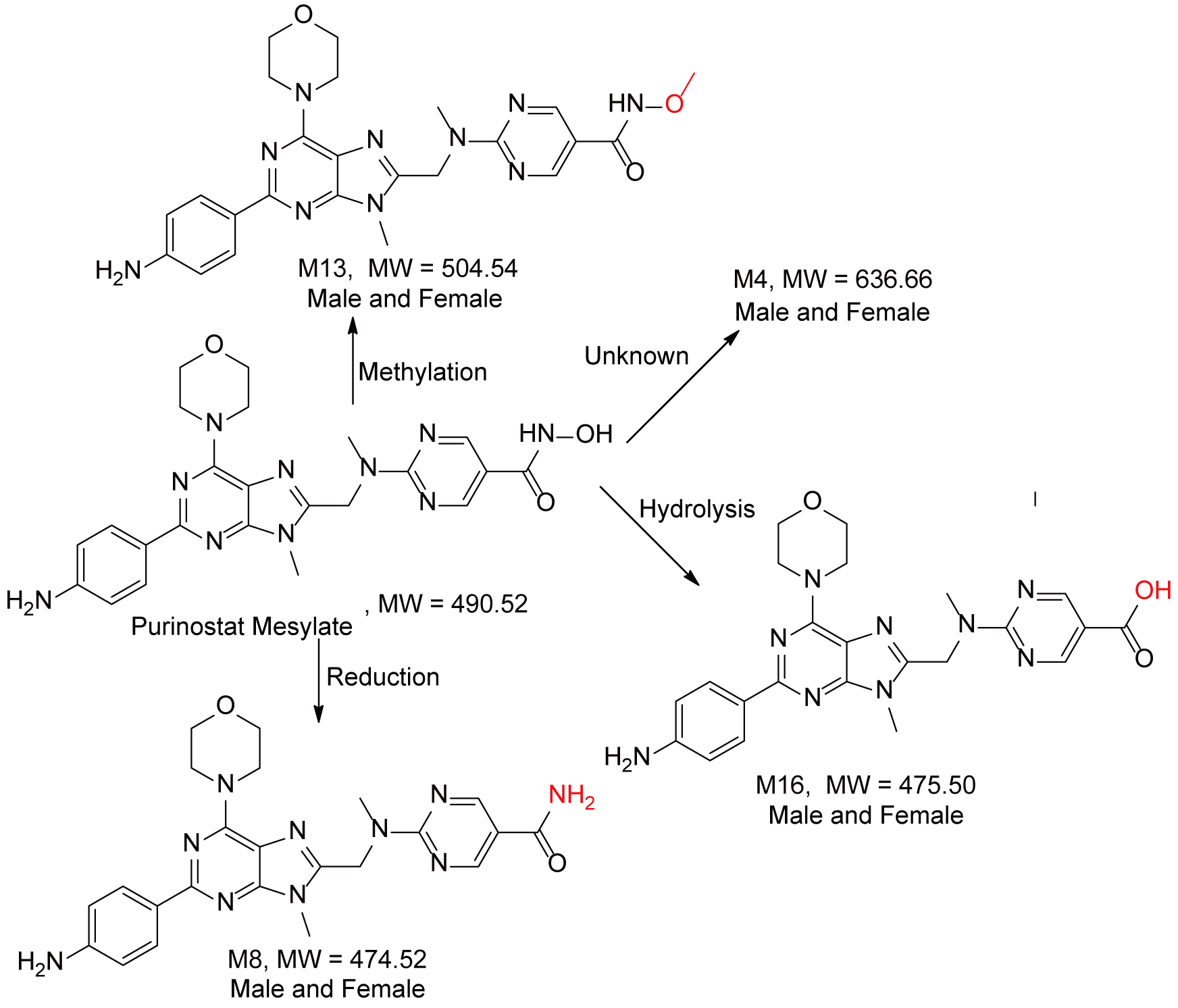


**（4）Pharmacokinetic Study of HCT116 Tumor-Bearing Nude Mice**

After a single and multiple intravenous administrations of 10 mg/kg Purinostat Mesylate (PM) to tumor-bearing nude mice, the plasma concentrations of PM in the multiple dosing group were found to be lower than those in the single dosing group. Following a single intravenous injection of Purinostat Mesylate, the elimination half-life (T_1/2_) of PM in plasma and tumor was 1.21 ± 0.82 hours and 45.34 ± 4.74 hours, respectively. The plasma AUC_(0-8h)_ was 174 ± 25.2 µg/Lh, while the tumor AUC_(0-96h)_ was 12922 ± 498 µg/Lh. The peak concentrations (C_max_) were 442 ± 67.2 ng/mL and 1041 ± 167 ng/mL, respectively. The mean residence time (MRT) was 0.51 ± 0.06 hours in plasma and 39.48 ± 4.74 hours in tumor.

After multiple intravenous administrations of Purinostat Mesylate, the drug was measurable in plasma only at 0.5, 2, and 4 hours, resulting in insufficient data points to calculate the corresponding pharmacokinetic parameters. The elimination half-life (T_1/2_) in the tumor was 68.7 ± 54.5 hours, with an AUC_(0-96h)_ of 9068 ± 213 µg/L*h and a peak concentration (C_max_) of 283 ± 64.8 ng/mL. The mean residence time (MRT) was 34.7 ± 1.86 hours.

The elimination half-life of Purinostat Mesylate in plasma was relatively short for both single and multiple dosing groups, while the elimination half-life in the tumor was long. This indicates that Purinostat Mesylate is rapidly eliminated from plasma and quickly reaches the tumor and other tissues, where it is maintained for an extended period, achieving considerable concentrations even 96 hours post-administration. Western Blot results showed that the changes in the tumor marker Ac-H4 correlated with the changes in Purinostat Mesylate concentration in the tumor.

In tumor-bearing nude mice receiving multiple intravenous injections of 10 mg/kg Purinostat Mesylate, tumor size was measured with calipers before treatment, and the dynamic changes in tumor size were monitored daily throughout the treatment until the conclusion of the experiment. Compared to the blank control group, multiple dosing significantly inhibited tumor growth.

**（5）Pharmacokinetics and Tissue Distribution Study of Purinostat Mesylate in Ighmyc Mice**

In B cell-related tumors, the mechanism of disease often involves the translocation of the MYC oncogene to the immunoglobulin promoter region, leading to its hyperactivation. The translocation of the MYC gene to the IGH enhancer (t(8;14) (q24;q32)) is a molecular hallmark of Burkitt lymphoma. The Ighmyc (Eμ-myc) mouse model successfully mimics this pathogenic mechanism by translocating the MYC oncogene to the immunoglobulin μ enhancer. These transgenic mice exhibit a high incidence of spontaneous B cell lymphoma, characterized by affected organs such as bone marrow, spleen, and enlarged lymph nodes. We isolated bone marrow and enlarged spleen from diseased Ighmyc mice and transplanted them into normal C57 mice via tail vein injection to induce a primary B cell lymphoma model. This study aims to investigate the pharmacokinetic characteristics and tissue distribution of Purinostat Mesylate in plasma and target organs in the context of primary B cell lymphoma.

The experimental results indicate that after a single intravenous injection of 5 mg/kg Purinostat Mesylate (PM) in Ighmyc mice, the elimination half-lives (T_1/2_) of the drug in plasma, spleen, and lymph nodes were 12.2 ± 2.10 h, 20.8 ± 7.94 h, and 21.9 ± 10.2 h, respectively. The plasma AUC_(0-48h)_ was 875 ± 437 µg/L*h, while the spleen AUC_(0-72h)_ was 12462 ± 244 µg/L*h, and the lymph node AUC_(0-72h)_ was 16135 ± 2036 µg/L*h. The peak concentrations (C_max_) were 304 ± 277 ng/mL, 410 ± 69.9 ng/mL, and 578 ± 153 ng/mL, respectively. The mean residence times (MRT) were 6.00 ± 1.00 h, 24.8 ± 1.03 h, and 23.4 ± 3.59 h, respectively. In contrast, after a single intravenous injection of 5 mg/kg Purinostat Mesylate in wild-type mice, no drug concentration was detectable in plasma after 24 hours. However, in Ighmyc mice, the plasma drug concentration after the same duration was 7.11 ± 3.15 ng/mL. These results indicate that in primary B cell lymphoma, the drug remains at high concentrations in plasma and target tissues, such as the spleen and lymph nodes, which contain a large number of tumor cells. The half-lives (T_1/2_) of 12.2 h, 20.8 h, and 21.9 h are significantly longer than the half-life of 1.21 h observed in normal mice. The pharmacokinetic characteristics support the notion that spaced dosing of the drug can still maintain favorable pharmacodynamic outcomes.

After a single intravenous injection of 5 mg/kg Purinostat Mesylate in Ighmyc mice, the drug was maintained for a long duration in the spleen and lymph nodes. Even 48 hours post-administration, the concentrations of Purinostat Mesylate were still 134 ± 18.5 ng/mL and 178 ± 46.0 ng/mL, respectively, which are significantly higher than the in vitro IC50 values of the drug. Furthermore, the distribution concentrations of the drug in the spleen and lymph nodes were notably higher than in other tissues, with minimal distribution in the brain and skeletal muscle, suggesting a lower risk of neurotoxicity for this strain.

Additionally, flow cytometric analysis of the peripheral blood in the mice showed that approximately 48.9% of the abnormal B220+/IgM- tumor cell population could be detected in the peripheral blood of model mice that had not been treated with Purinostat Mesylate. A high proportion of transformed cells was also present in the peripheral blood of diseased mice, with the disease phenotype consistent with that of the original donor. After a single intravenous treatment of Purinostat Mesylate at a dose of 5 mg/kg, there was a significant reduction in the proportion of tumor cells in the peripheral blood 48 hours post-treatment, with only about 18.8% of the abnormal B220+/IgM- cell population remaining detectable. Moreover, 48 hours after the single intravenous administration of Purinostat Mesylate at 5 mg/kg, a comparison with the blank control group revealed a significant reduction in the size of the spleen and lymph nodes, indicating that Purinostat Mesylate has a good therapeutic effect on Ighmyc mice.

#### Pharmacokinetics in Non-Rodent Animals

**（1）Pharmacokinetics of Purinostat Mesylate After Intravenous Injection in Male and Female Beagle Dogs**

After a single intravenous injection of 0.3, 1, and 3 mg/kg Purinostat Mesylate in male and female Beagle dogs, the maximum blood concentrations (C_max_) were reached immediately after the injection, measuring 130 ± 35.5, 411 ± 162, and 1230 ± 166 ng/mL, respectively. The plasma clearance rates (CL) were 68.4 ± 13.2, 73.4 ± 17.9, and 71.5 ± 11.6 mL/min/kg, respectively. The steady-state apparent volume of distribution (Vdss) values were 3.69 ± 0.957, 3.85 ± 1.80, and 2.89 ± 0.605 L/kg, respectively. The elimination half-lives (T_1/2_) were 4.49 ± 0.669, 7.05 ± 3.07, and 8.47 ± 1.26 hours, respectively. The area under the plasma concentration-time curve from time zero to the last quantifiable time point (AUC_0-last_) was 75.1 ± 18.3, 238 ± 63.1, and 713 ± 122 h•ng/mL, respectively.

After a single intravenous injection of Purinostat Mesylate at doses of 0.3, 1, and 3 mg/kg in male and female Beagle dogs, the systemic exposure (AUC_0-last_ and C_max_) demonstrated a dose-dependent linear increase as the dose increased from 0.3 to 1 mg/kg and from 1 to 3 mg/kg. Overall, within the dose range of 0.3 to 3 mg/kg, the systemic exposure (AUC_0-last_ and C_max_) in both male and female animals showed a dose-dependent linear increase.

After the single intravenous administration in male and female Beagle dogs at doses of 0.3, 1, and 3 mg/kg, there were no significant gender differences in systemic exposure (AUC_0-last_ and C_max_). Following a continuous intravenous injection of 1 mg/kg Purinostat Mesylate once daily for 7 days, there was no significant accumulation observed on day 7 compared to day 1 for both male and female experimental animals.

**（2）Plasma Protein Binding Rate**

The plasma protein binding rate of Purinostat Mesylate was measured using equilibrium dialysis in Sprague-Dawley rats, Beagle dogs, crab-eating monkeys, and human plasma. The experimental results indicated that at three tested concentrations (0.2, 2, and 10 µM), the binding rates (%) of Purinostat Mesylate were as follows: 94.3, 95.5, and 95.5 in Sprague-Dawley rat plasma; 83.1, 83.6, and 83.3 in Beagle dog plasma; 78.4, 79.5, and 79.0 in crab-eating monkey plasma; and 90.3, 90.0, and 90.4 in human plasma. At concentrations of 0.2, 2, and 10 µM, Purinostat Mesylate exhibited moderate binding rates in SD rat plasma (at the concentration of 0.2 µM), while demonstrating higher binding rates in SD rat plasma at the concentrations of 2 and 10 µM. The binding rates in the plasma of all four species did not show significant concentration dependence.

**（3）Metabolites and Metabolic Pathways in Beagle Dog Plasma**

Four metabolites of Purinostat Mesylate were detected in plasma samples from both male and female dogs, namely M4 (MW = 636.66, unknown product), M8 (MW = 474.52, reduction product), M13 (MW = 505.54, methylation product), and M16 (MW = 475.50, hydrolysis product). The primary metabolic pathways involved hydrolysis, methylation, and reduction, with no significant gender differences observed. The parent compound, Purinostat Mesylate, accounted for more than 35% of the UV absorption peak area in the plasma samples from both male and female dogs, indicating it is one of the major components. Purinostat Mesylate is primarily metabolized in the plasma of male and female dogs through hydrolysis, methylation, and reduction pathways. A possible metabolic pathway structure diagram is shown below:


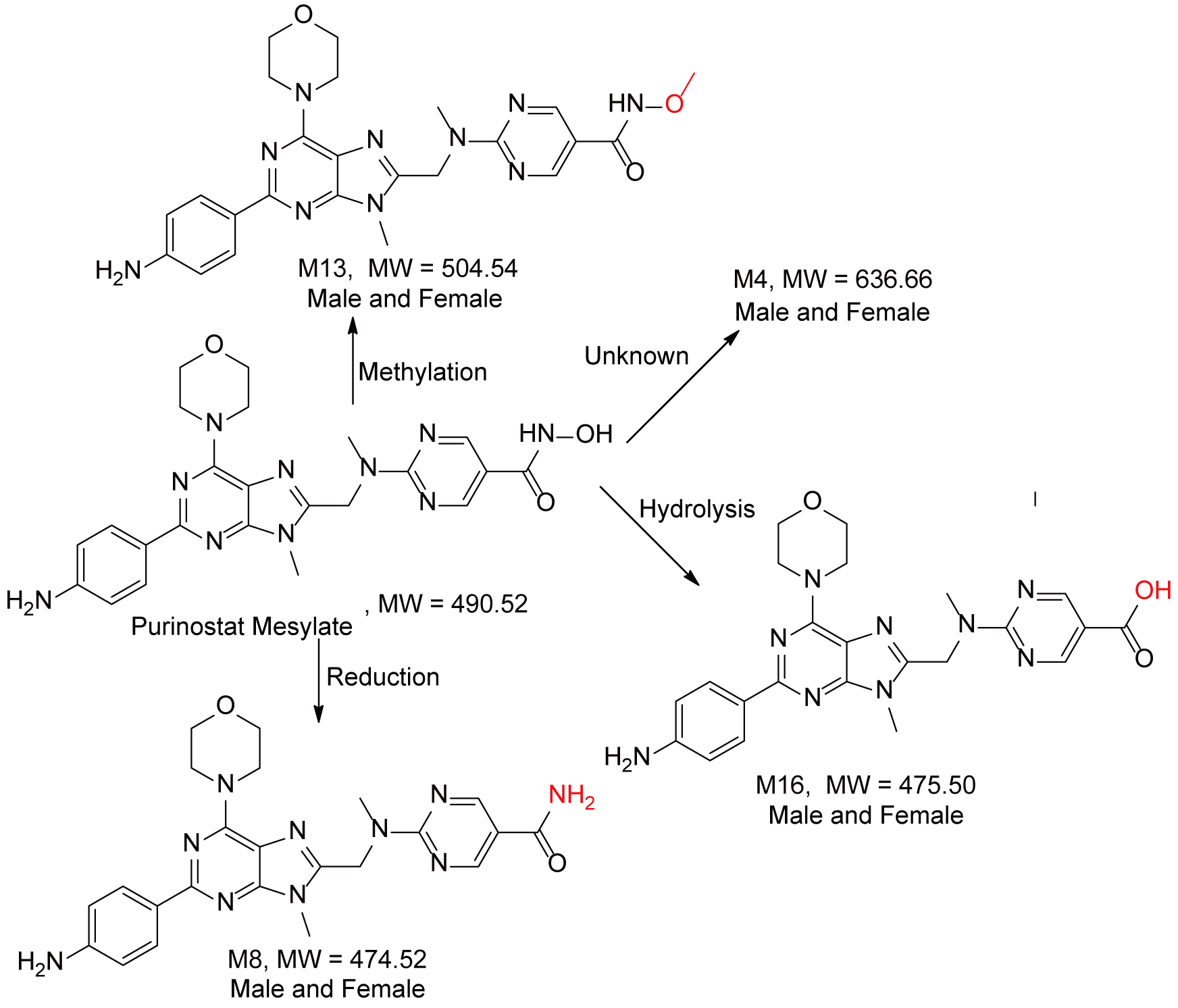


#### In Vitro Metabolism Studies

**（1）Inhibition Studies of Purinostat Mesylate on Human Liver Microsomal Cytochrome P450 Isoenzymes**

Inhibition studies of Purinostat Mesylate on human liver microsomal cytochrome P450 isoenzymes indicated that Purinostat Mesylate exhibited weak or no inhibition on CYP1A2, CYP2B6, CYP2C8, CYP2C9, CYP2C19, CYP2D6, and CYP3A4 (using midazolam as the substrate) with IC_50_ values greater than 10.0 μM. However, it showed moderate inhibition of CYP3A4 (using testosterone as the substrate) with an IC_50_ of 1.13 μM.

**（2）Induction Studies of Purinostat Mesylate on Human Liver Cells**

Co-culturing Purinostat Mesylate with cryopreserved human liver cells demonstrated that it did not act as an inducer of the cytochrome P450 isoenzymes CYP1A2, CYP2B6, and CYP3A4 at concentrations of 0.0500, 0.500, and 5.00 µM, as indicated by both in vitro enzyme activity and gene expression data across the three donor liver cell lines. Cytotoxicity data showed that Purinostat Mesylate exhibited cytotoxic effects on donor liver cells at concentrations up to 5.00 μM.

**（3）Metabolic Stability Studies of Purinostat Mesylate in In Vitro CD-1 Mice, SD Rats, Beagle Dogs, Crab-Eating Monkeys, and Human Liver Microsomes**

The metabolic stability of Purinostat Mesylate was evaluated in CD-1 mice, SD rats, Beagle dogs, crab-eating monkeys, and human liver microsomes. Under conditions of 37°C, 1 µM of Purinostat Mesylate was incubated with liver microsomes from the five species mentioned above, supplemented with a NADPH regeneration system, for varying durations up to 60 minutes. The concentration of Purinostat Mesylate in the resulting samples was analyzed using LC/MS/MS. By calculating the remaining percentage of Purinostat Mesylate at each time point, the half-lives (T_1/2_) of Purinostat Mesylate in the liver microsomes of CD-1 mice, SD rats, Beagle dogs, crab-eating monkeys, and humans were determined to be >186.0, >186.0, 97.6, 3.1, and >186.0 minutes, respectively. The corresponding liver intrinsic clearance (CLint) values were <29.7, <13.5, 20.4, 598.1, and <6.8 mL/min/kg. These results indicate that Purinostat Mesylate is metabolized slowly or not at all in CD-1 mice, SD rats, and human liver microsomes, is metabolized at a moderate rate in Beagle dog liver microsomes, and is metabolized rapidly in crab-eating monkey liver microsomes. Additionally, the metabolism of Purinostat Mesylate in crab-eating monkey liver microsomes does not depend on NADPH.

**（4）Recombinant Human Cytochrome P450 Isoenzymes**

The metabolic reaction phenotype of Purinostat Mesylate was determined using in vitro human liver microsomes and recombinant human cytochrome P450 isoenzyme test systems (CYP1A2, CYP2B6, CYP2C8, CYP2C9, CYP2C19, CYP2D6, and CYP3A). The results from the specific chemical inhibition experiments showed that the specific inhibitors for cytochrome P450 isoenzymes (CYP1A2, CYP2B6, CYP2C8, CYP2C9, CYP2C19, CYP2D6, and CYP3A) had no significant inhibitory effect on the formation of metabolite M8, with all inhibition rates being less than or equal to 1.3%. This indicates that CYP1A2, CYP2B6, CYP2C8, CYP2C9, CYP2C19, CYP2D6, and CYP3A play a minimal role or no role in the generation of M8. The recombinant human cytochrome P450 enzyme metabolic experiments indicated that CYP2C19 and CYP3A4 may be involved in the production of the metabolite M8, with relative contribution rates of 31.5% and 35.8%, respectively. Other isoenzymes (CYP1A2, CYP2B6, CYP2C8, CYP2C9, and CYP2D6) played a minimal role or no role in the formation of M8, with relative contribution rates all being less than or equal to 16.6%. Using recombinant enzyme metabolism methods, studies were conducted on CYP3A5, CYP4F2, CYP4F3B, and FMO3. The experimental results indicated that CYP4F2 is involved in the generation of the metabolite M8, while CYP3A5, CYP4F3B, and FMO3 do not play a role in the formation of M8. In summary, the results from both the specific chemical inhibition method and the recombinant human cytochrome P450 enzyme metabolism method suggest that CYP2C19 and CYP3A may be involved in the generation of the metabolite M8, whereas the other isoenzymes (CYP1A2, CYP2B6, CYP2C8, CYP2C9, and CYP2D6) have minimal or no effect on the formation of M8. This indicates that there may be other unknown enzymes participating in the metabolism of Purinostat Mesylate to produce M8 in human liver microsomes and playing a certain role.

# Purposes of the Study

## Primary Purposes

- To observe the tolerability and safety of intravenous administration of Purinostat Mesylate, either as a single or multiple infusions, in patients with hematological malignancies, primarily those related to B-cell tumors (including but not limited to B-cell lymphomas, multiple myeloma, B-cell acute leukemia, T-cell lymphomas, and T-cell acute leukemia) who have relapsed or are refractory to treatment, particularly those who have experienced disease progression after standard treatment or are unsuitable for standard treatment.
- To observe the dose-limiting toxicities (DLTs) of intravenous Purinostat Mesylate in patients with relapsed or refractory B-cell-related hematological malignancies, in order to determine the maximum tolerated dose (MTD) and provide a basis for the dosing regimen in Phase II clinical trials.

## Secondary Purposes

- To evaluate the pharmacokinetic parameters of intravenous administration of Purinostat Mesylate, either as a single or multiple infusions, in patients with relapsed or refractory B-cell-related hematological malignancies.
- To assess the pharmacodynamic indicators of intravenous Purinostat Mesylate after single and multiple infusions in patients with relapsed or refractory B-cell-related hematological malignancies.
- To preliminarily observe the efficacy of Purinostat Mesylate in treating patients with relapsed or refractory B-cell-related hematological malignancies.

# Study Design

## Overall Description of the Study

This study is a single-center, open-label, dose-escalation Phase I clinical trial that employs a conventional "3+3" dose escalation scheme. A total of 21 to 42 patients with relapsed or refractory hematological malignancies primarily associated with B-cell tumors will be enrolled (including but not limited to B-cell lymphomas, multiple myeloma, B-cell acute leukemia, T-cell lymphomas, and T-cell acute leukemia, in cases of disease progression, relapse, or those who are unsuitable for standard treatment after receiving standard treatment protocols). After obtaining informed consent, participants will undergo screening within two weeks prior to the first administration to confirm eligibility and to conduct a series of baseline assessments.

The study is divided into three phases: (1) Single Administration Phase (D1-D7): Eligible participants will be admitted to the hospital on the night before the trial (D0) and will receive an intravenous infusion of Purinostat Mesylate at approximately 8:00 AM the following day (D1). The infusion will be completed within 30 minutes, and participants will be monitored for six days post-administration. (2) Multiple Administration Phase (D8-D25): Participants who complete the single administration phase will proceed to the multiple administration phase. In this phase, doses will be administered on D8, D11, and D15. Participants will be monitored for the occurrence of dose-limiting toxicities (DLTs) and to determine the maximum tolerated dose (MTD). Completion of the multiple administration phase will constitute the main portion of the study. (3) Extended Treatment Phase: After completing the multiple administration phase, participants who tolerate the treatment well and do not experience disease progression, as determined by the investigator, may continue treatment according to the original protocol if it is deemed beneficial. During the extended treatment phase, each treatment cycle will be evaluated by the investigator for tolerability and absence of tumor progression. Based on the tolerability and pharmacokinetic results from other participants in the higher dose groups that have been completed, if no DLTs are observed, participants may be adjusted to a higher dose group for continued treatment until disease progression or intolerable toxicity occurs.

The starting dose will be 1.2 mg/m², and dose escalation will be conducted according to the Modified Fibonacci Scheme, with escalation increments of 100%, 67%, 50%, 40%, 33%, and 33%. Only after completing the single and multiple administration phases of the previous dose group, and based on the tolerability and pharmacokinetic results of that dose group, can a decision be made on whether to proceed to the next dose group, until the maximum tolerated dose (MTD) is established. It is anticipated that the MTD will be determined within seven dose groups, with a maximum sample size projected to be 42 participants.

## Dosing Regimen

### Considerations for the Starting Dose

The starting dose of Purinostat Mesylate will be determined based on a comprehensive evaluation of preclinical in vitro pharmacodynamics, in vivo pharmacodynamics across different species, pharmacokinetic data, and toxicological study results.

**1）Estimation of the Maximum Recommended Starting Dose Based on Toxicology Results**

According to the "Guidelines for the Clinical Trial of Antineoplastic Drugs," for cytotoxic agents, the maximum recommended starting dose (MRSD) can be determined as one-tenth of the maximum tolerated dose (MTD) in rodent studies or one-sixth of the MTD in non-rodent species, expressed in mg/m². For some non-cytotoxic antineoplastic agents, due to their relatively lower toxicity, the starting dose for single administration in Phase I clinical trials can be calculated using one-fifth of the No Observed Adverse Effect Level (NOAEL) from non-rodent studies, or higher.

Based on the MTD calculations, the MRSD should be the value derived from the most sensitive species, which is 3.33 mg/m². From the NOAEL calculations, the MRSD should be less than 1.2 mg/m².

Table 2 Calculation of MRSD Based on MTD from Rodent and Non-Rodent Species

|  | MTD（mg/kg） | Km | HED（mg/m^2^） | MRSD（mg/m^2^） |
| --- | --- | --- | --- | --- |
| Rats | 30 | 6 | 180 | 18 |
| Beagle dogs | 1 | 20 | 20 | 3.33 |

*MTD: Maximum Tolerated Dose，Km: mg/kg Dose conversion to human equivalent dose inmg/m2 Dosing conversion factor，HED: Human Equivalent Dose，MRSD: Maximum Recommended Starting Dose*

Table 3 Calculation of MRSD Based on NOAEL from Non-Rodent Species

|  | NOAEL（mg/kg） | Km | HED（mg/m^2^） | MRSD（mg/m^2^） |
| --- | --- | --- | --- | --- |
| Beagle dogs | <0.3 | 20 | 6 | <1.2 |

**2）Calculation of Pharmacologically Active Dose Based on Preclinical In Vivo Pharmacodynamic Study Results**

The Pharmacological Active Dose (PAD) based on mice is used to predict the minimum dose that may produce the expected pharmacological effect in humans. According to the calculated results, the minimum dose that produces the expected pharmacological effect is 0.75 mg/m².

Table 4 Calculation of MRSD Based on NOAEL from Non-Rodent Species

|  | PAD（mg/kg） | Km | HED（mg/m2） | MRSD（mg/m2） |
| --- | --- | --- | --- | --- |
| Mouse | 2.5 | 3 | 7.5 | 0.75 |

**3）Prediction of Human Clearance Based on Preclinical Pharmacokinetic Results**

Using allometric scaling, combined with preclinical pharmacokinetic results, the clearance rate of the drug in humans is predicted. The calculated clearance rate of the drug in humans ranges from 74.64 to 164.16 L/h.

Table 5 Prediction of the Drug Clearance Rate in Humans Using Allometric Scaling

| **Methods** | **Predicted Human Clearance Rate（L/h）** |
| --- | --- |
| Simple Allometric Scaling | 164.16 |
| Single-Species Allometric Scaling | 96.5（rat） |
|  | 133.90（dog） |
| Two-Species Allometric Scaling | 100 |
| MLP-Corrected Species Scaling (ROE) | 74.64 |
| Multi-Exponential Allometric Scaling | 106.70 |

According to the formula Dose  = CL × AUC× F × 1/SF，where F is the bioavailability (equal to 1 for intravenous infusion) and SF is the safety factor, typically chosen as 10. Based on the NOAEL from preclinical trials in rats and Beagle dogs, along with the predicted human clearance rate using single-species allometric scaling, the calculated starting doses are 1.46 mg/m² and 0.62 mg/m², respectively.

Table 6 Calculation of the Starting Dose Based on the NOAEL from Rats and the Predicted Human Clearance Rate

|  | NOAEL (mg/kg) | AUC (ng.h/ml) | CL (L/h) | Initial dose (mg/m^2^) |
| --- | --- | --- | --- | --- |
| Rats | < 3 | 249 | 96.5 | 1.46 |
| Beagle dogs | < 0.3 | 76 | 133.9 | 0.62 |

*Note: The body surface area for humans is calculated based on a height of 170 cm and a weight of 60 kg, resulting in 1.65 m^2^*

**4）Determination of Starting Dose**

Taking into account the above information and the dose used in Phase I clinical trials of a similar HDAC inhibitor (Panobinostat), which is 1.2 mg/m², the starting dose is determined to be 1.2 mg/m².

### Considerations for Dosing Frequency

In preclinical pharmacokinetic studies, after a single intravenous injection of Purinostat Mesylate in rats, the drug is eliminated quickly, with a half-life of 3.79-6.24 minutes. In contrast, following a single intravenous injection in Beagle dogs, the elimination is slower, with a half-life of 4.49-8.47 hours.

In preclinical in vivo pharmacodynamic studies using a human HCT116 tumor xenograft mouse model, although the plasma half-life of Purinostat Mesylate after a single intravenous injection is only 1.21 hours, the elimination half-life in tumor tissue averages 45.34 hours. Furthermore, after 96 hours, the concentration remains at 0.14 µM, which is significantly higher than the IC50 of Purinostat Mesylate observed in in vitro studies. Additionally, the AUC_0-t_ of the drug in tumor tissue is 74.41 times greater than that in plasma. These results suggest that Purinostat Mesylate can maintain a high level of activity in tumor tissue even 96 hours after administration.

After injecting 5 mg/kg of Purinostat Mesylate into Ighmyc transgenic mice (B-cell lymphoma) and wild-type mice, it was observed that 24 hours later, the drug concentration in the plasma of wild-type mice was undetectable, while the drug concentration in the plasma of Ighmyc transgenic mice was 7.11 ng/mL. Additionally, higher drug concentrations were found in the blood, spleen, and lymph nodes containing a large number of tumor cells, with a longer retention time compared to wild-type mice. Even 48 hours post-administration, the drug concentration remained far above the IC_50_ observed in in vitro studies, and the distribution of the drug in the brain and skeletal muscle was minimal, suggesting a potentially lower risk of neurotoxicity.

Considering the information from preclinical studies and the administration methods of similar HDAC inhibitors in clinical settings (for example, Panobinostat is administered in cycles of 21 days, with four doses given in the first two weeks, where the interval between each pair of doses is no less than 3 days), the current dosing frequency for Purinostat Mesylate is provisionally set to a single administration and continuous dosing on days D1, D8, D11, and D15, with a 10-day interval. Subjects meeting the criteria as assessed by the physician will enter the continuation dosing phase, which consists of administering the drug on days 1, 4, 8, and 11 of each cycle, again with a 10-day interval, for a total cycle length of 21 days. The subsequent dosing frequency may be further adjusted based on the pharmacokinetic and pharmacodynamic results from the first subject.

### Dose Group

The starting dose is set at 1.2 mg/m², with dose escalation conducted according to the Modified Fibonacci Scheme, with escalation increments of 100%, 67%, 50%, 40%, 33%, and 33%. The dose escalation increments may be further adjusted based on the pharmacokinetic (PK), pharmacodynamic (PD), and safety data from the previous dose group.

If the maximum dose specified in this protocol (15 mg/m²) has not yet reached the maximum tolerated dose (MTD), the decision to continue dose escalation will be based on PK, PD metrics, and safety data. If PK or PD indicators suggest that the drug has not reached a saturating level of exposure in the body, the dose group will continue to escalate according to the Modified Fibonacci Scheme until MTD is observed or until sufficient drug exposure is achieved (the maximum drug exposure should not exceed the exposure levels observed at the dog HNSTD dose, which corresponds to an equivalent human dose of 36.88 mg/m²). The dose preceding the MTD may be considered the recommended dose for Phase II clinical trials.

Table 7 Dose Escalation Scheme

| **Group Number** | **1** | **2** | **3** | **4** | **5** | **6** | **7** | **…..** |
| --- | --- | --- | --- | --- | --- | --- | --- | --- |
| Escalation Ratio | Starting Dose | 100% | 67% | 50% | 40% | 33% | 33% | 33% |
| Administered Dose (mg/m^2^) | 1.2 | 2.4 | 4.0 | 6.0 | 8.4 | 11.2 | 15 | Previous Dose×33% increment  （Maximum Drug Exposure < 36.88 mg/m^2^） |
| Number of Cases | 3-6 | 3-6 | 3-6 | 3-6 | 3-6 | 3-6 | 3-6 | 3-6 |

### Escalation Process

The trial begins with a low dose, enrolling 3-6 subjects in each dose group. The decision to proceed to the next dose group will be based on whether at least 3 evaluable subjects from the previous dose group experience Dose Limiting Toxicity (DLT) and the corresponding safety data within 25 days after the first administration. An evaluable subject is defined as one who has received ≥80% of the planned dose or has experienced DLT after at least one dose

The specific regulations for dose escalation are as follows:

Table 8 Dose Escalation Rules

| **Occurrence of DLT** | **Actions** |
| --- | --- |
| No DLT observed | Proceed to the next dose group |
| 1 DLT observed among 3 subjects | Recruit an additional 3 subjects to receive the same dose of the investigational drug and observe for DLT |
| 1 DLT observed among 6 subjects | Proceed to the next dose group |
| ≥2 DLTs observed among 3 or 6 subjects | Reduce to the previous dose group.   - If there are fewer than 6 evaluable subjects in the previous dose group, recruit an additional 3 subjects to reach a total of 6 evaluable subjects. If no more than 1 DLT is observed among the 6 evaluable subjects, terminate the trial, and that dose group will be considered the MTD. - If there are already 6 evaluable subjects in the previous dose group, terminate the trial, and that dose will be considered the MTD. |

**Enrollment Sequence for Subjects in the Same Dose Group:** At the beginning of each dose group, only 1 subject will be enrolled to observe the tolerability and safety of that subject within 25 days after the first administration.

- Only if this subject does not experience DLT can the remaining 2 subjects in the same dose group be enrolled simultaneously.
- If this subject experiences DLT, enrollment of the third subject can only occur after the second subject has completed the 25-day observation period following the first administration, and only if the second subject has not experienced DLT.

### Individual Dose Adjustment

**1）Dose Escalation**

Dose escalation is not permitted during the single-dose and multiple-dose phases.

In the continuation dosing phase, individual dose escalation for a subject is allowed under the following conditions:

- The subject has received at least 2 cycles of the specified dose of the drug and has tolerated it well, with no dose-limiting toxicities (DLTs) observed;
- For the proposed escalated dose, there are safety data available for at least 2 evaluable subjects 25 days after their first dose, with no DLTs observed;

**2）Dose Reduction and Treatment Interruption**

If a subject experiences a dose-limiting toxicity (DLT) during the single-dose or multiple-dose phases, treatment must be interrupted until the toxicity recovers to Grade 1 or below. Based on the investigator's judgment that the subject may benefit from continued treatment, the subject may resume medication.

In the continuation dosing phase, the dose may be adjusted or treatment may be interrupted according to the following rules:

| **Toxicity** | **CTCAE Grade** | **Measures** |
| --- | --- | --- |
| Nausea and vomiting | Grade 3-4 | If it occurs for the first time, consider using antiemetics for treatment and interrupt the medication until the toxicity recovers to Grade 1 or below. Continue treatment with the original dose, and it is recommended to use antiemetics as a preventive measure.  If it occurs again, interrupt the medication until recovery to Grade 1 or below, and reduce the dose to the previous level. |
| Diarrhea | Grade 3 | If it occurs for the first time, consider using antidiarrheal medication for treatment and interrupt the medication until the toxicity recovers to Grade 1 or below. Continue treatment with the original dose, and it is recommended to use antidiarrheal medication as a preventive measure.  If it occurs again, interrupt the medication until recovery to Grade 1 or below, and when resuming treatment, reduce the dose to the previous level. |
|  | Grade 4 | If it occurs for the first time, consider using antidiarrheal medication for treatment and interrupt the medication until the toxicity recovers to Grade 1 or below. Adjust the dose to the previous level for treatment, and it is recommended to use antidiarrheal medication as a preventive measure.  If it occurs again, discontinue treatment and withdraw from the study. |
| Neutropenia | Grade 3 | ANC: 0.75 - 1.0 × 10^9^ /L, maintain the current dose and continue treatment. ANC: 0.5 - 0.75 × 10^9^ /L, interrupt treatment until ANC recovers to 1.0 × 10^9^ /L, then continue treatment at the original dose. |
| Febrile neutropenia | Grade 3 | Interrupt treatment until ANC recovers to 1.0 × 10^9 /L; when resuming treatment, reduce the dose to the previous level. |
| Thrombocytopenia | Grade 3 | Maintain the current dose of treatment and monitor platelet counts weekly |
|  | Grade 3-4 | Grade 3 thrombocytopenia with bleeding: interrupt treatment until PLT recovers to 50 × 10^9^ /L; when resuming treatment, reduce the dose to the previous level and monitor platelet counts weekly.  Grade 4 thrombocytopenia: interrupt treatment until PLT recovers to 50 × 10^9^ /L; when resuming treatment, reduce the dose to the previous level and monitor platelet counts weekly. |
| Anemia | Grade 3 | Interrupt treatment until Hb recovers to 10 g/dL; when resuming treatment, reduce the dose to the previous level. |
| QTc interval prolongation | Grade 2 | If it occurs for the first time: interrupt treatment until QTc < 450 ms; when resuming treatment, reduce the dose to the previous level.  If it occurs again: discontinue treatment and withdraw from the study. |
| Neurotoxicity | Grade 2 | If it occurs for the first time: interrupt treatment until the toxicity recovers to Grade 1 or below; when resuming treatment, reduce the dose to the previous level.  If it occurs again: discontinue treatment and withdraw from the study. |
| Liver function (elevated ALT and AST levels) | Grade 2 | Baseline normal, with Grade 2 elevation of ALT or AST occurring after treatment.  If it occurs for the first time: interrupt medication until toxicity recovers to Grade 1 or below; when resuming medication, reduce the dose by one level and increase the frequency of liver function monitoring.  If it occurs again: discontinue treatment and withdraw from the study. |
|  | Grade 3 | For patients with normal baseline, if Grade 3 toxicity occurs with ALT or AST > 8 × ULN, discontinue treatment and withdraw from the study.  For patients with Grade 1 baseline, if Grade 3 toxicity occurs with ALT or AST > 8 × ULN, consider discontinuing treatment and withdrawing from the study.  For patients with normal baseline or Grade 1 baseline, if Grade 3 toxicity occurs for the first time with 5 × ULN < ALT or AST < 8 × ULN, interrupt medication until toxicity recovers to Grade 1 or below; when resuming medication, reduce the dose by one level and increase the frequency of liver function monitoring. If it occurs again, discontinue treatment and withdraw from the study. |
|  | Grade 4 | Discontinue treatment and withdraw from the study. |
| Others | Grade 2-3 | For relapsedGrade 2-3 toxicities, except for alopecia and fatigue, treatment must be interrupted until the toxicity recovers to Grade 1 or below; when resuming treatment, reduce the dose to the previous level. |

## Concomitant Medication

### Permitted Concomitant Medications

From the date of signing the informed consent form, any concomitant treatments or changes in concomitant medications that subjects must receive during the trial must be recorded by the investigator in the corresponding section of the eCRF, including the drug name, dosage, administration times, and indications.

During the screening period and throughout the trial, subjects are allowed to use the following medications:

- Supportive treatment for tumors and adverse events occurring during the trial, such as antibiotics, antiemetics, antidiarrheals, antipyretics, antihistamines, analgesics, medications to increase white blood cell counts, and medications to control high white blood cell counts (e.g., hydroxyurea), etc.;
- Corticosteroids: prednisone < 20 mg/day or equivalent doses of other corticosteroids for the treatment of conditions unrelated to the disease under study; if using prednisone > 20 mg/day or equivalent doses of other corticosteroids, the duration should not exceed 4 days for the prevention or treatment of adverse events occurring during the trial (e.g., infusion reactions);
- Others: Baseline medications for concomitant conditions such as hypertension or diabetes that the investigator deems will not affect the observation of study endpoints and can be used concomitantly.

### Prohibited Concomitant Medications

During the screening period and throughout the trial, subjects are prohibited from using the following medications:

- Any other antitumor drugs (chemotherapy, immunotherapy) except for the investigational drug;
- Radiation therapy;
- Medications that prolong the QT interval;
- Other investigational therapeutic agents.

## Definition of DLT

Any drug-related toxicity occurring within 25 days after the first dose of the investigational drug, as defined by CTCAE version 5.0, that is considered related to the investigational drug (including definitely related, probably related, or possibly related) will be regarded as a DLT:

1. Hematologic Toxicity

- Grade 3 febrile neutropenia, defined as an absolute neutrophil count (ANC) < 1.0 × 10^9^/L accompanied by fever, with a temperature > 38.3℃, or a temperature sustained ≥ 38.0℃ for more than 1 hour;
- Grade 4 neutropenia without fever lasting ≥ 5 days;
- Grade 3 thrombocytopenia (25×10^9^/L < platelet count≤50×10^9^/L) accompanied by bleeding of Grade 2 or higher according to the WHO bleeding classification (2017 revised version), or Grade 4 thrombocytopenia lasting≥7 days with platelet transfusion treatment occurring≥2 times (excluding acute leukemia patients).

1. Any Grade 3 or higher non-hematologic toxicity, including but not limited to:

- Grade 3 or higher QT interval prolongation;
- Hepatic toxicity: If the baseline levels of ALT, AST, or TBIL for the subject are ≤ Grade 1, then drug-related Grade 3 toxicity will be considered as DLT.

**The following situations are excluded:**

- Grade 3 fatigue, weakness, or fever;
- Grade 4 infusion reactions;
- Grade 3 controllable hypertension (systolic blood pressure ≥ 160 mmHg or diastolic blood pressure≥100 mmHg);
- Grade 3 nausea or vomiting lasting less than 72 hours, with adequate antiemetic and supportive treatment provided;
- Grade 3 diarrhea lasting less than 72 hours, with adequate antidiarrheal and supportive treatment provided;
- Grade 3 or 4 electrolyte abnormalities that can improve to Grade 2 or lower within 72 hours;
- Hair loss.

1. Patients with Acute Leukemia

- Persistent pancytopenia lasting > 42 days, accompanied by bone marrow hypoplasia (bone marrow cellularity < 5%) and the absence of leukemic cells in peripheral blood and bone marrow, will be considered a dose-limiting toxicity (DLT) due to bone marrow suppression. Pancytopenia is defined as leukocyte count < 4.0 × 10^9^/L, hemoglobin < 100 g/L, and platelet count < 100 × 10^9^/L.
- If the investigator cannot attribute any of the following events to a clearly identifiable cause (such as tumor progression, underlying or concurrent diseases, or concomitant medications), these events will be considered dose-limiting toxicities: hematologic toxicities possibly related to the study drug, grade 3 or higher non-hematologic toxicities, with the following exceptions:
- Alopecia
- Grade 3 fatigue, weakness, anorexia, fever, or constipation
- Grade 3 nausea, vomiting, or diarrhea that does not require tube feeding, intravenous nutritional support (TPN), or hospitalization
- Infections, bleeding, or other expected direct complications caused by underlying active leukemia

1. Others: Significant medical events deemed by the investigator to be possibly related to the study drug and discussed with the sponsor may be considered dose-limiting toxicities (DLT).
2. Any grade 5 adverse events.
3. Participants who have not recovered from toxicity and have interrupted treatment for more than 14 days (excluding interruptions greater than 14 days due to hematologic toxicity in acute leukemia).

## Definition of MTD

Within 25 days after the first dose, if ≤ 1 DLT occurs among 6 subjects, the highest dose that meets this criterion will be defined as the MTD. If all dose groups specified in the protocol have been completed and no DLT occurs in the highest dose group, then the highest dose will be determined as the MTD.

## Dietary and Lifestyle Restrictions

### Diet

During the single-dose and multiple-dose phases, subjects should avoid consuming foods or beverages containing caffeine (such as tea, coffee, cola, etc.), xanthines (such as chocolate), and grapefruit. Smoking and alcohol consumption are prohibited throughout the entire trial.

Subjects will be admitted to the ward the day before the PK study and will remain there until 24 hours after the PK blood samples are collected post-dosing. The investigational drug will be administered to subjects in the morning on the day of the trial, following an overnight fast (at least 10 hours) for fasting administration. A light meal may be provided 2 hours after dosing, with lunch approximately 4 hours after dosing and dinner approximately 10 hours after dosing; thereafter, normal meal times may resume. Fluid intake will be controlled for 1 hour before dosing and for 2 hours after dosing, with no restrictions on fluid intake during other times, while avoiding drinks containing caffeine such as coffee, tea, and cola. Subjects should refrain from vigorous exercise during the trial. When blood collection time points overlap with meal or examination time points, blood collection will be performed first, followed by the examination, and then the meal.

### Contraception

Male participants (and their female partners) as well as female participants of childbearing potential should use reliable contraception from the first dose of the study drug until six months after the last dose (see Appendix 14.9)

# Trial Population

## Sample Size

This trial is provisionally set to include 7 dosage groups, with a planned enrollment of 21-42 participants primarily consisting of patients with B-cell-related tumors in the category of relapsed or refractory hematologic malignancies.

## Definition of the Trial Population

The specific definition of relapsed or refractory hematologic malignancies primarily involving B-cell-related tumors is referenced as follows. For diseases not specifically defined in this section, the investigator will determine them according to the latest guidelines for each condition:

- 1. **B-cell lymphoma and T-cell lymphoma**

According to the "Guidelines for the Diagnosis and Treatment of Diffuse Large B-Cell Lymphoma in China (2013 Edition)" and recent clinical studies of newly marketed drugs, relapsedor refractory B-cell lymphoma and T-cell lymphoma are defined as follows (may not apply to certain rare or currently non-research hotspot pathological types):

- RelapsedB-cell lymphoma and T-cell lymphoma: Refers to disease progression after adequate treatment achieving remission (including PR and CR).

Disease progression is indicated by the appearance of any new lesions or an increase in the diameter of existing lesions by ≥ 50%, including but not limited to the following situations: (1) Lymphadenopathy: The appearance of any new lesion with a diameter > 1.5 cm; an increase of ≥ 50% in the sum of the products of the maximum vertical diameters of multiple lesions; or an increase of ≥ 50% in the maximum diameter of a single lesion with a short axis > 1 cm before treatment. Additionally, lesions that were FDG avid or PET positive before treatment and are PET positive after treatment. (2) Spleen and liver: An increase of ≥50% in the sum of the products of the maximum vertical diameters of any lesions. (3) New or relapsedbone marrow involvement.

- Refractory B-cell lymphoma and T-cell lymphoma: Defined as meeting any of the following criteria: (1) Disease progression after two cycles of standard regimen chemotherapy, or failure to achieve remission (including PR and CR) after four cycles of treatment; (2) Achieving CR after standard regimen chemotherapy, followed by relapse within 6 months; (3) Two or more relapses after achieving CR; (4) Relapse after hematopoietic stem cell transplantation.
  1. **B-cell acute leukemia and T-cell acute leukemia (collectively referred to as acute lymphoblastic leukemia, ALL)**

According to the "Guidelines for the Diagnosis and Treatment of Adult Acute Lymphoblastic Leukemia in China (2016 Edition)" and the National Comprehensive Cancer Network (NCCN) guidelines, relapsedor refractory acute lymphoblastic leukemia in adults is defined as follows:

- RelapsedALL: The reappearance of blasts in peripheral blood or bone marrow (proportion > 5%) after achieving complete remission (CR) following induction therapy, or the presence of extramedullary leukemia.
- Refractory ALL: Defined as meeting any of the following criteria: ① Failure to achieve CR with a standard 4-week induction therapy regimen, and no response after an additional two weeks of chemotherapy. ② Relapse within 6 months of the first remission, classified as early relapse. ③ Relapse more than 6 months after the first remission, but failure of retreatment with the original induction regimen. ④ Multiple relapses (relapse ≥ 2 times).
  1. **Multiple Myeloma（MM）**

According to the "Chinese Guidelines for the Diagnosis and Treatment of Multiple Myeloma (2017 Revision)" and the definition provided by the International Myeloma Working Group (IMWG) in 2016, relapsed or refractory multiple myeloma is defined as follows:

- Relapsed multiple myeloma (MM) refers to patients who have previously achieved at least a minimal response (MR) or better following treatment but then experience disease progression, necessitating the initiation of salvage therapy. It is categorized into clinical relapse and relapse after complete response (CR).

Clinical relapse is defined as meeting at least one of the following criteria: (1)The appearance of new bone lesions or soft tissue plasmacytomas (excluding osteoporotic fractures); (2)A clear increase in existing plasmacytomas or bone lesions (an increase of 50% in the sum of the products of the maximum vertical diameters of measurable lesions, with an absolute value of ≥1 cm); (3)Hypercalcemia; (4) A decrease in hemoglobin of≥20 g/L (unrelated to treatment or non-MM factors); (5)An increase in serum creatinine of≥176.8 µmol/L that is related to MM since the start of MM treatment; (6) Hyperviscosity syndrome related to serum M-protein.

Relapse after complete response (CR) is defined as meeting any of the following criteria: (1)The reappearance of serum or urine M-protein confirmed by immunofixation electrophoresis; (2) A bone marrow plasma cell percentage of ≥5%; (3) Any other signs of progressive disease (PD).

- Refractory Multiple Myeloma (MM) is categorized into primary refractory and relapsed refractory.

Primary Refractory: This is defined as the lack of response to any treatment, where the patient has not achieved at least a minimal response (MR). This includes cases where the disease does not respond to treatment but has not progressed, as well as cases where the disease does not respond to treatment and progresses.

Relapsed Refractory: This refers to patients who had previously achieved at least a minimal response (MR) or better following treatment but then experience disease progression. In this case, after salvage therapy, there is no response, or the disease progresses within 60 days of the last treatment.

## Grouping

Once participants have signed the informed consent form and undergone screening, they will receive a three-digit screening number in the order of their screening, such that the first participant to sign the consent and undergo screening will have a screening number of 001. Eligible participants will be assigned a three-digit enrollment number based on the order of their enrollment, with the first enrolled participant receiving an enrollment number of 001. In cases where a participant needs to be replaced, the replacement participant will be assigned a new enrollment number in accordance with the order of enrollment.

## Inclusion Criteria

1. Age between 18 and 70 years, regardless of gender.
2. Diagnosis confirmed by histopathological or cytological examination (according to the "Diagnostic Criteria and Efficacy Standards for Hematological Diseases" (4th Edition) published by Science Press) for hematological malignancies, including but not limited to B-cell lymphoma, multiple myeloma, B-cell acute leukemia, T-cell lymphoma, and T-cell acute leukemia. Patients must have experienced disease progression, relapse, or be unsuitable for standard treatment after receiving standard treatment regimens (specific definitions for relapsed or refractory disease can be found in Section 4.2).
3. No severe organic lesions in the heart, lungs, liver, or kidneys (LVEF≥50%; total bilirubin ≤1.5×ULN; ALT ≤1.5×ULN; AST ≤1.5×ULN; serum creatinine ≤1.5×ULN or CCr > 40 mL/min).
4. No severe coagulation dysfunction (PT≤1.5×ULN, APTT≤1.5×ULN, TT≤1.5× ULN, and FIB≥1.0 g/L).
5. No severe hematological abnormalities (absolute neutrophil count≥1.5×10^9^/L, platelets≥75×10^9^/L, hemoglobin≥80 g/L), and no transfusion of platelets, red blood cells, or hemoglobin within the 2 weeks prior to screening.
6. At least 4 weeks or more than 5 half-lives since the last anti-tumor treatment (chemotherapy, radiotherapy, biological therapy, or immunotherapy) prior to enrollment.
7. Expected survival time of≥12 weeks.
8. ECOG performance status of≤2.
9. Willingness to participate in the study and sign the informed consent form.

## Exclusion Criteria

1. Toxicity Recovery: Patients whose prior cancer treatment-related toxicities have not recovered to Grade I or below, or who have not fully recovered from previous surgeries.
2. Severe Chronic Diseases: Patients with severe lung, liver, kidney, gastrointestinal diseases, or chronic diseases of major organs.
3. Pregnancy and Lactation: Pregnant or breastfeeding women, and patients of childbearing potential who refuse to use contraception during the trial.
4. Recent Cardiac Events: History of acute myocardial infarction, congestive heart failure (NYHA Class ≥ 2), unstable angina, or stroke within the past 6 months.
5. Impaired Cardiac Function: Patients with impaired cardiac function (echocardiogram showing ejection fraction < 45%, complete left bundle branch block with ST segment depression > 1 mm or T-wave inversion in two or more leads; congenital ventricular or atrial arrhythmias; clinically significant tachycardia (> 100 beats/min), bradycardia (< 50 beats/min), QTc > 450 ms (male), QTc > 480 ms (female), or clinically significant heart disease such as unstable angina, congestive heart failure, or myocardial infarction within the last 6 months).
6. CNS Involvement or Mental Disorders: Patients with central nervous system lymphoma/leukemia or significant psychiatric disorders.
7. Organ Transplant History: Patients with a history of organ transplantation.
8. Severe Active Infections: Patients with severe active infections.
9. Allergies: Known severe allergy to the study drug, its excipients, or HDAC inhibitors.
10. Viral Infections: Patients who are positive for HCV antigen or antibodies, HIV antigen or antibodies, HBsAg positive, HBcAb positive, and have peripheral blood HBV DNA levels ≥ 1 × 10^3 IU/mL.
11. Substance Abuse: Individuals with alcohol dependence or drug abuse.
12. Recent Participation in Other Trials: Patients who have participated in other drug clinical trials within the past month.
13. Other Factors: Any other factors that the investigator deems as unsuitable for participation in the trial.

## Criteria for Withdrawal from the Study

Participants have the right to withdraw from the study at any stage, and their interests will not be adversely affected by their decision to exit. If a participant requests to withdraw from the trial, study treatment must be discontinued. Additionally, if a participant does not explicitly state their intention to withdraw but ceases to take medication and undergo tests, this will also be considered a withdrawal or dropout.

If a participant decides to withdraw, all observations should be completed and reported as thoroughly as possible before exiting. A full final assessment should be conducted at the time of withdrawal, and the reason for withdrawal must be documented. If a participant withdraws due to adverse events or abnormal laboratory results, relevant details must be recorded in the case report form.

The investigator should consider early termination of the study for a participant in the event of any of the following occurrences:

1. Evidence of disease progression;
2. Appearance of intolerable toxicity leading to discontinuation of the study drug, and failure to recover within 14 days after discontinuation;
3. Poor compliance by the participant, making them unsuitable to continue in the study;
4. Serious violations of the study protocol that affect drug tolerance and safety evaluation;
5. Participant lost to follow-up;
6. Pregnancy;
7. Other reasons deemed by the investigator as making the participant unsuitable to continue in the trial.

## Subject Supplementation

Subjects who discontinue the study within 25 days after the first dose for reasons other than dose-limiting toxicities (DLTs) — such as participant withdrawal from the clinical trial, serious protocol violations, failure to meet inclusion/exclusion criteria, poor compliance, or early evidence of disease progression — may be supplemented to ensure that the number of evaluations for DLTs remains sufficient.

# Investigational Drug

## Drug Introduction

| Drug Name | Purinostat Mesylate for injection |
| --- | --- |
| Characteristics | Light yellow to off-white loose aggregate or powder |
| Active Ingredient | Purinostat Mesylate（Purinostat Mesylate）  Chemical name: 2-(((2-(4-Aminophenyl)-9-methyl-6-(4-morpholinyl)-9H-purine-8-yl)methyl)methylamino)-N-hydroxy-5-pyrimidine-carboxamide Mesylate (IUPAC)  Chemical structure :   |
| Composition | Purinostat Mesylate， Hydroxypropyl beta-cyclodextrin, arginine, glucosamine, mannitol |
| Specification | 20 mg/bottle (as Purinostat Mesylate) |
| Storage Conditions | Protect from light, store in a tightly closed container in a cool place |
| Expiration Date | Tentative shelf life of 24 months |
| Batch Number |  |
| Manufacturer | Chengdu Zenitar Biomedical Technology Co., Ltd |

## Drug Labeling

The sponsor will package the investigational drug and affix a label that includes the drug name, specification, dosage and administration, drug batch number, expiration date, storage conditions, supplying entity, precautions, and the statement "For Phase I clinical trial use only."

**Sample Drug Label**

| **Purinostat Mesylate for injection**  **（For Phase I clinical trial use only.）**  **Specification:** 20 mg/bottle  **Dosage Form:** Intravenous infusion  **Dosage:** Administer according to the specified dose group for the subject  **Drug Batch Number:**  **Expiration Date:**  **Supplying Entity:** Chengdu Zenitar Biomedical Technology Co., Ltd  **Storage Conditions:** Protect from light, store in a tightly closed container in a cool place  **Precautions:** The product should be immediately prepared into a solution after dissolution and should not be frozen. The prepared solution should be administered immediately, and the time from preparation to the end of infusion should not exceed 6 hours. |
| --- |

## Preparation Method

Prior to administration, allow each vial of lyophilized Purinostat Mesylate to reach room temperature. Once equilibrated, add physiological saline to each vial to achieve the appropriate drug concentration. Withdraw the intended dose of the diluted solution and add it to physiological saline to a total volume of 50 mL, and infuse intravenously at a constant rate over 30 minutes.

The product should be prepared into a solution immediately after dissolution and must not be frozen. The prepared solution should be administered immediately, and the time from preparation to the end of infusion should not exceed 6 hours.

## Administration Method

The designated personnel will administer the drug via an infusion pump for a constant rate intravenous infusion. The administration volume is 50 mL, and the infusion time is 30 minutes. The actual infusion time will be recorded. During the clinical trial, adjustments to the drug concentration, infusion rate, infusion time, and administration volume will be made as necessary based on the subject's safety and tolerability.

## Drug Distribution and Storage

The investigational drug is provided by the sponsor and is prepared in a workshop that meets the conditions of the Good Manufacturing Practice (GMP) standards. The drug is inspected and must meet quality standards. The sponsor will package and distribute the investigational drug to the research sites in a single shipment according to the study phase, with records of distribution and receipt. The research site will have designated personnel responsible for storing the investigational drug and recording its usage. The investigator will maintain accurate records of the receipt, distribution, use, return, and disposal of the investigational drug. After the trial is completed, any remaining and used drug must correspond with the records. This investigational drug is solely for clinical trial purposes and is not intended for any other use.

## Drug Transportation and Storage

The study drug requires cold chain transportation and must be stored under refrigeration at 2-8°C, protected from light and in a tightly sealed container.

## Drug Management

The investigational drug for this trial may only be used for the purposes of this study and must not be used for any other purpose. It must not be sold or transferred to any individuals not participating in the clinical trial.

To ensure the safety of the drug, a designated pharmacist is responsible for its management, storing it according to the specified conditions and maintaining detailed records of its distribution, receipt, collection, and return.

The study drug requires cold chain transportation and must be stored under refrigeration.

Once the investigational drug is received, the pharmacist must conduct an inventory of the study drug. Receipts and shipping documents should be kept in the investigator's management records. The following information must be recorded: the quantity received and stored, the batch number on the label, the dates of storage, transfer, and removal, the name of the pharmacist, the quantity dispensed to each subject, and the name of the person responsible for each batch of drug procurement.

Only the pharmacist is authorized to dispense the investigational drug. Before the trial, the drug will be distributed to the investigator responsible for medication management, and drug usage will be recorded. After the trial, the remaining and used drugs must correspond with the records. Monitoring during the study will include the supply, usage, storage, and handling of leftover drugs, as well as maintaining accurate records.

Once the study is completed, all unused drugs, if not authorized by Chengdu Zenitar Biomedical Technology Co., Ltd for local disposal, will be collected by the clinical monitor for the project along with the corresponding usage records. The outer packaging of the returned drugs must clearly indicate the study protocol number and the research site number, and the quantity returned, drug usage, and signatures with dates must be recorded.

Researchers must maintain all records related to drug management. The investigational drug distribution log should be kept up to date and include the following information: ① subject identification numbers; ② the quantity of drug dispensed to each subject and the corresponding dates. During the study, the sponsor's monitors may inspect the drug management records at any time.

# Study Process

The study is divided into five phases: the screening phase, the single-dose administration phase, the multiple-dose administration phase, the extended dosing phase, and the study conclusion or early termination of participation.

## Screening Phase（D-14 ~ D0）

After the subject signs the informed consent form, the following items will be conducted within 2 weeks prior to the first administration (D-14 to D0):

- Signing the informed consent form
- Tissue or cellular pathology
- Demographic information (age, gender, height, weight, etc.)
- Medical history and treatment history
- Allergy history
- Review of inclusion and exclusion criteria
- ECOG performance status
- Vital signs: including temperature, heart rate, respiratory rate, and blood pressure (systolic and diastolic)
- Physical examination: including examinations of the skin, mucous membranes, lymph nodes, head, neck, chest, abdomen, spine/extremities, and nervous system
- Urine or blood pregnancy test
- Laboratory tests:
- Complete blood count: red blood cell count, hemoglobin, platelet count, white blood cell count, neutrophil count, lymphocyte count
- Urinalysis: urine protein, urine red blood cells, urine white blood cells
- Blood biochemistry: serum creatinine, urea, total bilirubin, alanine aminotransferase (ALT), aspartate aminotransferase (AST), alkaline phosphatase, total protein, albumin, electrolytes (K+, Na+, Ca2+, Mg2+, Cl-), total cholesterol, low-density lipoprotein (LDL), high-density lipoprotein (HDL), creatine kinase (CK), creatine kinase isoenzyme (CK-MB), α-hydroxybutyrate dehydrogenase (α-HBDH), lactate dehydrogenase, fasting blood glucose
- Coagulation function: prothrombin time (PT), activated partial thromboplastin time (APTT), thrombin time (TT), fibrinogen (FIB)
- M Protein Testing: Conducted only for patients with multiple myeloma, including: (1) serum protein electrophoresis (Albumin, Alpha 1, Alpha 2, Beta 1, Beta 2, Gamma, M-spike); (2) immunofixation electrophoresis (IgG, IgA, IgM, light chain types); (3) β2-microglobulin (β2-MG); (4) serum immunoglobulin quantification (serum IgG, serum IgA, serum IgM, serum kappa light chains, serum lambda light chains, kappa/lambda ratio).
- 12-Lead Electrocardiogram: PR interval, QRS duration, QT interval, QTc interval.
- Virology Testing: HIV testing (antibodies, antigens), HCV testing (antibodies), HBV testing (HBsAg, HBsAb, HBcAb, HBeAg, HBeAb, HBV DNA titer).
- Imaging Studies: CT or PET-CT or whole-body X-ray examination; lymphoma subjects will undergo CT or PET-CT, while multiple myeloma patients will undergo X-ray examination (the investigator will determine if additional localized CT is needed); for leukemia subjects, the need for examination will be determined by the investigator based on clinical circumstances.
- Bone Marrow Examination Includes: (1) bone marrow cytology smear classification; (2) bone marrow biopsy + immunohistochemistry; (3) flow cytometry; for leukemia patients, bone marrow examination will include only the bone marrow smear and flow cytometry.
- Echocardiogram.
- Observation and Recording of Adverse Events.
- Record of Concomitant Medications.

## Single-Dose Administration Phase（D1-D7）

After successful screening and enrollment, the single-dose administration phase begins on Day 1 (D1). Following the single intravenous infusion on D1, the subject will be monitored for 3 days.

- D1 intravenous infusion administration
- Pharmacokinetic (PK) blood sample collection: collect 3 mL blood samples at the following time points: within 0.5 hours before D1 administration, and at 5 minutes, 10 minutes, 20 minutes, 30 minutes (immediately after administration), 32 minutes, 35 minutes, 45 minutes, 1 hour, 2 hours, 4 hours, 8 hours, and 12 hours after administration
- Biomarker blood sample collection (only for leukemia patients): collect 4 mL blood samples at the following time points: within 0.5 hours before D1 administration, and at 1 hour, 4 hours, 24 hours (D2), and 48 hours (D3) after administration
- Vital signs: including temperature, heart rate, respiratory rate, and blood pressure (systolic and diastolic), to be checked within 0.5 hours before administration, and at 0.5 hours, 2 hours, 24 hours, and on D7 after administration
- Physical examination: including examination of skin, mucous membranes, lymph nodes, head, neck, chest, abdomen, spine/extremities, and nervous system, to be conducted on D2 and D7
- Laboratory tests: to be conducted on D2 and D7
- Complete Blood Count (CBC): Red blood cell count, hemoglobin, platelet count, white blood cell count, neutrophil count, lymphocyte count
- Urinalysis: Urine protein, urine red blood cells, urine white blood cells
- Blood Biochemistry: Blood creatinine, urea, total bilirubin, alanine aminotransferase (ALT), aspartate aminotransferase (AST), alkaline phosphatase, total protein, albumin, electrolytes (K+, Na+, Ca2+, Mg2+, Cl-), total cholesterol, low-density lipoprotein (LDL), high-density lipoprotein (HDL), creatine kinase (CK), creatine kinase isoenzyme (CK-MB), α-hydroxybutyrate dehydrogenase (α-HBDH), lactate dehydrogenase, fasting blood glucose
- Coagulation Function: Prothrombin time (PT), activated partial thromboplastin time (APTT), thrombin time (TT), fibrinogen (FIB)
- 12-lead Electrocardiogram (ECG): PR interval, QRS duration, QT interval, QTc interval, to be checked once before administration on D1 (results from within 5 days prior to D1 are acceptable), and again 2 hours, 6 hours, on D2, and on D7 after administration.
- Echocardiogram: This may be performed if symptoms such as precordial pain or palpitations occur, or if the investigator deems it necessary.
- Adherence Assessment: Adherence will be assessed on D1.
- Observation and Recording of Adverse Events.
- Record of Concomitant Medications.

## Multiple-Dose Administration Phase（D8-D25）

After the single-dose administration phase is completed, if the subject tolerates the treatment well, they will enter the multiple-dose administration phase.

- IV Infusion Administration on D8, D11, and D15
- PK Blood Sample Collection:

On D11: collect blood samples 0.5 hours before administration.

On D15: collect blood samples within 0.5 hours before administration, and at 5 minutes, 10 minutes, 20 minutes, 30 minutes (immediately after administration), 32 minutes, 35 minutes, 45 minutes, 1 hour, 2 hours, 4 hours, 8 hours, and 12 hours after administration (3 mL each time).

- Biomarker Blood Sample Collection (only for leukemia patients):

On D8: collect blood samples within 0.5 hours before administration, and at 1 hour, 4 hours, and 24 hours (D9) after administration (4 mL each time).

On D11: collect blood samples within 0.5 hours before administration and at 1 hour after administration (4 mL each time).

On D15: collect blood samples within 0.5 hours before administration, and at 1 hour, 4 hours, 24 hours (D16), 48 hours (D17), and 72 hours (D18) after administration (4 mL each time).

- Vital Signs: Including temperature, heart rate, respiratory rate, and blood pressure (systolic and diastolic), to be checked once within 0.5 hours before each administration and again 2 hours after administration on D8, D11, and D15, as well as once on D25.
- Physical Examination: Includes examination of the skin, mucous membranes, lymph nodes, head, neck, chest, abdomen, spine/extremities, and nervous system. This will be conducted once on D11, D15, and D25.
- Laboratory Tests: These will be conducted once on D11, D15, and D25.
- Complete Blood Count (CBC): Red blood cell count, hemoglobin, platelet count, white blood cell count, neutrophil count, lymphocyte count.
- Urinalysis: Urine protein, urine red blood cells, urine white blood cells.
- Blood Biochemistry: Blood creatinine, urea, total bilirubin, alanine transaminase (ALT), aspartate transaminase (AST), alkaline phosphatase, total protein, albumin, electrolytes (K+, Na+, Ca2+, Mg2+, Cl-), total cholesterol, low-density lipoprotein (LDL), high-density lipoprotein (HDL), creatine kinase (CK), creatine kinase isoenzyme (CK-MB), α-hydroxybutyrate dehydrogenase (α-HBDH), lactate dehydrogenase (LDH), fasting blood glucose.
- Coagulation Function: Prothrombin time (PT), activated partial thromboplastin time (APTT), thrombin time (TT), fibrinogen (FIB).
- M Protein Testing: Conducted only for patients with multiple myeloma, including: (1) serum protein electrophoresis (Albumin, Alpha 1, Alpha 2, Beta 1, Beta 2, Gamma, M-spike); (2) immunofixation electrophoresis (IgG, IgA, IgM, light chain types); (3) β2-microglobulin (β2-MG); (4) serum immunoglobulin quantification (serum IgG, serum IgA, serum IgM, serum kappa light chains, serum lambda light chains, kappa/lambda ratio); D25 examination once.
- 12-Lead Electrocardiogram: PR interval, QRS duration, QT interval, QTc interval; assessments at 2 hours and 6 hours after administration on D8, and before and after administration on D11 and D15 (2 hours and 6 hours post-dose), and once on D25.
- Echocardiogram: Performed if symptoms of cardiac abnormalities such as precordial pain or palpitations occur, or if the investigator deems it necessary.
- Imaging Studies: CT or PET-CT or whole-body X-ray examination conducted once on D25; lymphoma subjects will undergo CT or PET-CT, while multiple myeloma patients will undergo X-ray examination (the investigator will determine if additional localized CT is needed); for leukemia subjects, the need for examination will be determined by the investigator based on clinical circumstances.
- Bone Marrow Examination: Determined by the investigator based on clinical needs, including: (1) bone marrow cytology smear classification; (2) bone marrow biopsy + immunohistochemistry; (3) flow cytometry; for leukemia patients, bone marrow examination will include only the bone marrow smear and flow cytometry; D25 examination once.
- Efficacy Assessment: Efficacy will be assessed on D25.
- Compliance Assessment: Patient compliance will be assessed on D25.
- Observation and Recording of Adverse Events.
- Record of Concomitant Medications.

## Extended Treatment Phase（D26-）

- Intravenous Infusion Administration: Administered on D1, D4, D8, and D11 of each treatment cycle (21 days).
- ECOG Performance Status: Assessed before administration on D1 of each treatment cycle.
- Vital Signs: Including temperature, heart rate, respiratory rate, and blood pressure (systolic and diastolic); measured within 0.5 hours before each administration, 2 hours after administration, and once on D21.
- Physical Examination: Includes assessments of skin, mucous membranes, lymph nodes, head, neck, chest, abdomen, spine/extremities, and neurological system; conducted once on D1, D11, and D21 of each treatment cycle.
- Laboratory Tests: Conducted once on D1, D11, and D21 of each treatment cycle.
- Complete Blood Count (CBC): Red blood cell count, hemoglobin, platelet count, white blood cell count, neutrophil count, lymphocyte count.
- Urinalysis: Urine protein, urine red blood cells, urine white blood cells.
- Blood Biochemistry: Serum creatinine, urea, total bilirubin, alanine aminotransferase (ALT), aspartate aminotransferase (AST), alkaline phosphatase, total protein, albumin, electrolytes (K+, Na+, Ca2+, Mg2+, Cl-), total cholesterol, low-density lipoprotein (LDL), high-density lipoprotein (HDL), creatine kinase (CK), creatine kinase-MB (CK-MB), alpha-hydroxybutyrate dehydrogenase (α-HBDH), lactate dehydrogenase (LDH), fasting blood glucose.
- Coagulation Function Tests: Prothrombin time (PT), activated partial thromboplastin time (APTT), thrombin time (TT), fibrinogen (FIB).
- M Protein Testing: Conducted prior to administration at the start of the second treatment cycle only for participants with multiple myeloma.
- 12-Lead Electrocardiogram (ECG): PR interval, QRS interval, QT interval, and QTc interval; measured once before administration on D1, D11, and D21 of each treatment cycle, and once 2 hours after administration. Additional tests may be added at the discretion of the investigator if deemed necessary.
- Echocardiogram: Conducted if there are symptoms of cardiac abnormalities such as precordial pain or palpitations, or if deemed necessary by the investigator.
- Imaging Studies (CT or PET-CT or Whole-Body X-ray): Conducted before the next cycle of treatment after the 2nd, 4th, 6th, etc. cycles. CT or PET-CT scans are performed for lymphoma participants, while X-rays are conducted for multiple myeloma patients (local CT may be added at the investigator's discretion). For leukemia participants, the decision to conduct imaging tests will be made by the investigator based on clinical circumstances.
- Bone Marrow Examination (based on clinical needs during the extended treatment phase): Includes bone marrow cytology smear classification, bone marrow biopsy with immunohistochemistry, and flow cytometry; for leukemia patients, only bone marrow smears and flow cytometry will be performed.
- Efficacy Assessment: Completed before the next treatment cycle after the 2nd, 4th, 6th, etc. cycles.
- Adherence Assessment: Completed before each cycle to evaluate the participant's adherence from the previous cycle.
- Monitoring and Recording of Adverse Events.
- Recording of Concomitant Medications.

Note: If the laboratory tests are conducted within 3 days of the last laboratory tests from the previous cycle, they may be omitted.

## End of Study/Early Withdrawal from the Trial

- Vital Signs: Including temperature, heart rate, respiratory rate, and blood pressure (systolic and diastolic).
- Physical Examination: Including assessments of skin, mucous membranes, lymph nodes, head, neck, chest, abdomen, spine/extremities, and neurological system.
- ECOG Performance Status.
- Laboratory Tests:
- Complete Blood Count (CBC): Red blood cell count, hemoglobin, platelet count, white blood cell count, neutrophil count, lymphocyte count.
- Urinalysis: Urine protein, urine red blood cells, urine white blood cells.
- Blood Biochemistry: Serum creatinine, urea, total bilirubin, alanine aminotransferase (ALT), aspartate aminotransferase (AST), alkaline phosphatase, total protein, albumin, electrolytes (K+, Na+, Ca2+, Mg2+, Cl-), total cholesterol, low-density lipoprotein (LDL), high-density lipoprotein (HDL), creatine kinase (CK), creatine kinase-MB (CK-MB), alpha-hydroxybutyrate dehydrogenase (α-HBDH), lactate dehydrogenase (LDH), fasting blood glucose.
- Coagulation Function Tests: Prothrombin time (PT), activated partial thromboplastin time (APTT), thrombin time (TT), fibrinogen (FIB).
- M Protein Testing: Includes: (1) Serum protein electrophoresis (Albumin, Alpha 1, Alpha 2, Beta 1, Beta 2, Gamma, M-spike); (2) Immunofixation electrophoresis (IgG, IgA, IgM, light chain types); (3) Beta-2 microglobulin (β2-MG); (4) Serum immunoglobulin quantification (serum IgG, serum IgA, serum IgM, serum kappa light chain, serum lambda light chain, kappa/lambda ratio); this test is performed only for participants with multiple myeloma.
- 12-Lead Electrocardiogram (ECG): PR interval, QRS interval, QT interval, QTc interval.
- Echocardiogram: Conducted if there are symptoms of cardiac abnormalities such as precordial pain or palpitations, or if deemed necessary by the investigator.
- Urine or Serum Pregnancy Test.
- Imaging Studies (CT or PET-CT or Whole-Body X-ray): CT or PET-CT is performed for lymphoma participants, while X-ray is conducted for multiple myeloma patients (local CT may be added at the investigator's discretion). The decision for imaging in leukemia participants will be made by the investigator based on clinical circumstances.
- Bone Marrow Examination: (1) Bone marrow cytology smear classification; (2) Bone marrow biopsy with immunohistochemistry; (3) Flow cytometry; for leukemia patients, only bone marrow smears and flow cytometry will be performed.
- Efficacy Assessment.
- Adherence Assessment.
- Monitoring and Recording of Adverse Events.
- Recording of Concomitant Medications.

Note: (1) The time window for vital sign checks after administration is ±30 minutes. (2) Imaging studies and bone marrow examinations are acceptable if the results from the previous 4 weeks prior to screening do not require re-examination.

# Evaluation Indicators

## Tolerability and Safety Assessment

Participants who have received the experimental drug at least once will be included in the tolerability and safety assessment dataset. The evaluation indicators include:

- Dose-Limiting Toxicity (DLT): Defined in Section 3.4.
- Maximum Tolerated Dose (MTD): Defined in Section 3.5.
- Adverse Events: Types, severity, incidence, timing, duration, and relationship to the experimental drug, according to the CTCAE version 5.0.
- Vital Signs: Including temperature, heart rate, respiratory rate, and blood pressure (systolic and diastolic).
- Physical Examination: Including assessments of skin, mucous membranes, lymph nodes, head, neck, chest, abdomen, spine/extremities, and neurological system.
- Laboratory Tests:
- Complete Blood Count (CBC): Red blood cell count, hemoglobin, platelet count, white blood cell count, neutrophil count, lymphocyte count.
- Urinalysis: Urine protein, urine red blood cells, urine white blood cells.
- Blood Biochemistry: Blood creatinine, urea, total bilirubin, alanine aminotransferase (ALT), aspartate aminotransferase (AST), alkaline phosphatase, total protein, albumin, electrolytes (K⁺, Na⁺, Ca²⁺, Mg²⁺, Cl⁻), total cholesterol, low-density lipoprotein (LDL), high-density lipoprotein (HDL), creatine kinase (CK), creatine kinase-MB (CK-MB), alpha-hydroxybutyrate dehydrogenase (α-HBDH), lactate dehydrogenase (LDH), fasting blood glucose.
- Coagulation Function: Prothrombin time (PT), activated partial thromboplastin time (APTT), thrombin time (TT), fibrinogen (FIB).
- 12-Lead Electrocardiogram (ECG): PR interval, QRS interval, QT interval, QTc interval.
- Echocardiogram: Conducted if there are symptoms of cardiac abnormalities such as precordial pain or palpitations, or if deemed necessary by the investigator.
- Imaging Studies: CT, PET-CT, or whole-body X-ray.

## Pharmacokinetics

### Pharmacokinetic Parameters

Pharmacokinetic parameters will be calculated using the professional pharmacokinetic software WinNonlin version 8.1 or higher, utilizing the non-compartmental analysis (NCA) method. The pharmacokinetic parameters include:

Single-Dose Administration Phase: T_max_, C_max_, AUC_(0-t)_, AUC_(0-∞)_, Vd, Ke, T_1/2_, MRT, CL

Multiple-Dose Administration Phase: T_max_, C_ss_min_, C_ss_max_, Css_av, t_1/2_, CL, AUC_ss_

When calculating AUC, actual sampling times will be used for time points that fall outside the sampling window.

### Sample Collection Timing

Single-Dose Pharmacokinetic Study:

The PK sampling points will be adjusted based on the pharmacokinetic trial results and tolerability observed in the first patient as follows:

For each patient, blood samples of 3 mL will be collected from the antecubital vein using an indwelling needle at the following time points: within 0.5 hours before administration, and at 5 minutes, 10 minutes, 20 minutes, 30 minutes (immediately after administration), 32 minutes, 35 minutes, 45 minutes, 1 hour, 2 hours, 4 hours, 8 hours, and 12 hours after administration.

Multiple-Dose Administration Phase

D11: Blood samples of 3 mL will be collected within 0.5 hours before administration;

D15: Blood samples of 3 mL will be collected within 0.5 hours before administration, and at 5 minutes, 10 minutes, 20 minutes, 30 minutes (immediately after administration), 32 minutes, 35 minutes, 45 minutes, 1 hour, 2 hours, 4 hours, 8 hours, and 12 hours after administration.

### Sample Handling and Storage

Anticoagulant: K_2_EDTA

For each blood collection, 3 mL of blood should be placed in a pre-labeled anticoagulant vacuum tube. The tube should be gently inverted several times to mix the contents, then the blood sample tube should be positioned vertically in a tube rack. Centrifugation should be completed within 1 hour (2-8°C, 3500 rpm, 10 minutes). After centrifugation, plasma samples should be removed from the centrifuge, with each plasma sample aliquoted into two pre-labeled cryovials—one for testing and one as a backup. The samples should be stored in a -20±5°C freezer for pre-freezing and temporary storage, and should be transferred to a -70±10°C freezer within 2 hours or placed directly into a -70±10°C freezer for storage.

### Sample Testing

Establish and validate a UPLC-MS/MS method for determining the concentration of Purinostat Mesylate in plasma. According to the requirements of the 2015 edition of the "Pharmacopoeia of the People's Republic of China," Part IV, Section 9012 "Guidelines for the Validation of Quantitative Analysis Methods for Biological Samples," the method for determining drug concentration should be thoroughly validated, including evaluations of selectivity, carryover, lower limit of quantification, standard curve, accuracy, precision, matrix effect, and stability. The results should meet the requirements for drug evaluation.

This trial is designed to recruit 6 healthy volunteers (3 males and 3 females) to provide blank blood samples for pharmacokinetic (PK) method validation and testing. Healthy volunteers participating in this part of blood sample collection will sign a dedicated informed consent form, and the screening results and basic information of the volunteers will be retained in the corresponding subject's original data file. However, since they are not participating in the trial, their information does not need to be entered into the electronic case report form (eCRF).

After the validation of the biological sample analysis method is completed, the determination of the biological samples from the subjects will begin. Each sample is generally measured once, but re-testing may be conducted as per the standard operating procedures (SOP) of the testing unit when necessary. Biological samples from the same individual should preferably be analyzed within the same batch. A new standard curve should be established for each analytical batch of biological samples, and quality control (QC) samples at high, medium, and low concentrations should be analyzed concurrently. Each concentration of QC samples should be measured in duplicate and should be evenly distributed throughout the order of unknown samples. If the number of samples in an analytical batch is large, the number of QC samples for each concentration should be increased so that the total number of QC samples exceeds 5% of the total number of unknown samples. A maximum of 1/3 of the QC sample results may exceed the limits, but this cannot occur for QC samples of the same concentration. If the results of the QC samples do not meet the above requirements, the test results for that analytical batch will be considered invalid.

Samples with drug concentrations exceeding the upper limit of the linear range will be appropriately diluted with biological matrix and re-analyzed, with validation of the dilution methodology. For samples with concentrations below the lower limit of quantification, during pharmacokinetic analysis, samples taken before reaching C_max_ should be calculated as zero, while samples taken after reaching C_max_ should be considered not detectable (ND) to minimize the impact of zero values on the AUC calculation.

## Biomarker Testing (Applicable Only to Leukemia Patients)

### Biomarker

- Acetylation Levels of Histones H3 and H4

### Sample Collection Timing

Single-Dose Administration Phase: Blood samples of 4 mL will be collected within 0.5 hours before administration, and at 1 hour, 4 hours, 24 hours, and 48 hours after administration.

Multiple-Dose Administration Phase:

D8: Blood samples of 4 mL will be collected at 0.5 hours before administration, and at 1 hour, 4 hours, and 24 hours after administration.

D11: Blood samples of 4 mL will be collected at 0.5 hours before administration and at 1 hour after administration.

D15: Blood samples of 4 mL will be collected at 0.5 hours before administration, and at 1 hour, 4 hours, 24 hours, 48 hours, and 72 hours after administration.

### Sample Handling, Testing, and Storage

Anticoagulant: K_2_EDTA

For each blood collection, 4 mL of peripheral blood will be placed in an anticoagulant collection tube. Peripheral blood mononuclear cells (PBMCs) will be isolated using red blood cell lysis buffer or the Ficoll-Paque method. The isolated PBMCs will be lysed and prepared as samples according to the protocol for Western Blot analysis. The acetylation levels of histones H3 and H4 will be detected using the Western Blot method. Samples will be stored in a freezer at -70 ± 10°C.

Quantitative Analysis Method:

In the single-dose administration phase, the acetylation levels of histones will be referenced to the baseline (Baseline) established from the sample collected 0.5 hours before administration on D1. The results of all time point samples will be quantified, and the trends in histone acetylation levels at each time point will be analyzed. In the multiple-dose administration phase, the acetylation levels of histones will be referenced to the baseline (Baseline) established from the sample collected 0.5 hours before administration on D5. The results of all time point samples will be quantified, and the trends in histone acetylation levels at each time point will be analyzed.

## Sampling Time Window

For sampling intervals of less than or equal to 1 hour, the allowable error must be within ±5% of the previous time point interval. For sampling intervals greater than 1 hour, the allowable error must be within ±2.5% of the previous time point interval. Any deviations from the permitted sampling time range must be immediately documented in the relevant original data forms.

Table 9 Sampling Time Window

| Time Points (Post-Administration) | Time Window |
| --- | --- |
| 10min | ±1min |
| 20min | ±1min |
| 30min | ±1min |
| 35min | ±1min |
| 45min | ±1min |
| 1h | ±1min |
| 2h | ±2min |
| 4h | ±3min |
| 6h | ±3min |
| 8h | ±3min |
| 10h | ±3min |
| 24h | ±21min |
| 48h | ±36min |
| 72h | ±48min |

## Efficacy Evaluation

- Objective Response Rate (ORR): Defined as the proportion of patients whose tumor volume has decreased to a predetermined value and maintained for a minimum required duration. ORR is generally defined as the sum of complete response (CR) and partial response (PR).
- Progression-Free Survival (PFS): The time from the start of patient enrollment until the occurrence of objective tumor progression or death.
- Disease Control Rate (DCR): Refers to the proportion of patients whose tumors have either decreased or stabilized for a certain period, including cases of complete response (CR), partial response (PR), and stable disease (SD).

The efficacy evaluation correspondence for this trial is referenced in the table below. For other diseases not specified in the table, the investigator will determine efficacy based on the latest evaluation criteria for that disease:

| Disease Names | Efficacy Evaluation Criteria |
| --- | --- |
| B-cell Lymphoma  T-cell Lymphoma | ORR =CR+PR+MR  DCR= CR+PR+MR+SD |
| Multiple Myeloma | ORR =sCR+CR+VGPR+PR+MR |
| B-cell Acute Lymphoblastic Leukemia  T-cell Acute Lymphoblastic Leukemia | ORR= CR+CRi  This disease is not applicable for DCR analysis. |
| Other Relapsed or Refractory Hematologic Malignancies | For diseases not defined in this trial, the determination will be based on the clinical researcher's judgment according to the efficacy evaluation guidelines for each disease. |

# Adverse Events and Serious Adverse Events

## Adverse Events

### Definition

Adverse Events (AEs) refer to any unfavorable medical occurrences experienced by a patient or clinical trial subject after receiving a medication, but they do not necessarily have a causal relationship with the treatment. An adverse event can include any unfavorable and unintended sign (including abnormal laboratory findings), symptom, or disease that occurs in temporal relation to the use of the investigational drug, regardless of whether it is related to the drug. This includes, but is not limited to:

1. Worsening of pre-existing conditions prior to the use of the investigational drug;
2. Increased frequency or severity of pre-existing episodic events prior to the use of the investigational drug;
3. Abnormal changes detected or diagnosed after the use of the investigational drug, even if these changes may have existed prior to treatment;
4. Deterioration of diseases or symptoms that were already persistently present before the start of the study.

### Assessment of Adverse Event Severity

Adverse events will be categorized using the Medical Dictionary for Regulatory Activities (MedDRA) classification system. Investigators must grade each adverse event according to its severity based on the CTCAE version 5.0. If the severity of an individual adverse event is not graded in the text, the investigator may use their medical judgment to assign a grade based on the levels defined by CTCAE (ranging from Grade 1 to Grade 5).

Table 10 NCI-CTCAE Adverse Event Grading

| Grading | Description |
| --- | --- |
| Grade 1 | Mild; asymptomatic or mild symptoms; clinical or diagnostic observations only; intervention not indicated. |
| Grade 2 | Moderate; minimal, local, or non-invasive intervention indicated; age-related instrumental activities of daily living are limited. |
| Grade 3 | Severe or medically significant but not immediately life-threatening; hospitalization or prolongation of existing hospitalization indicated; disabling; limitations in activities of daily living. |
| Grade 4 | Life-threatening; urgent intervention indicated. |
| Grade 5 | Death |

Note: *Instrumental Activities of Daily Living (IADLs) refer to tasks such as cooking, shopping for groceries or clothing, using the telephone, managing finances, etc.
**Activities of Daily Living (ADLs) refer to tasks such as bathing, dressing and undressing, eating, toileting, and taking medications, as opposed to being bedridden.

### Assessment of the Relationship Between Adverse Events and Investigational Drug

The investigator should conduct a comprehensive analysis of the specific circumstances surrounding the adverse events experienced by the subjects, taking into account the subjects' medical history, concomitant conditions, and concomitant medications, in order to assess the relationship between the adverse events and the drug. The investigator should perform a causal analysis of the symptoms presented by the subjects during the medication process to evaluate any potential associations between the adverse events and the investigational drug. The considerations for causal analysis include the following five aspects:

1. Is there a reasonable temporal relationship between the start of medication and the occurrence of the adverse event (AE)?
2. Does the AE correspond to known types of AEs associated with the drug (as per literature)?
3. Can the AE be explained by concomitant medications, previous medications, the patient's clinical condition, or the effects of other therapies (alternative explanations)?
4. Does the AE resolve or lessen after discontinuation or dose reduction of the medication (resolution after discontinuation)?
5. Does the AE reoccur upon re-exposure to the same drug (recurrence upon re-administration)?

Assessment of Adverse Events and Causal Relationship with the Drug: Based on the five criteria mentioned above, the causal relationship will be analyzed and classified into five levels: definitely related, probably related, possibly related, probably not related, and definitely not related (Table 10).

Table 11 Relevant Criteria for Assessing Adverse Events

| Considerations | Drug Administration Occurred | Consistent with Literature | Other Explanations | Disappearance Upon Discontinuation | Reappearance Upon Readministration |
| --- | --- | --- | --- | --- | --- |
| Definitely Related | + | + | - | + | + |
| Likely Related | + | + | - | + | ？ |
| Possibly Related | + | + | ± | ± | ？ |
| Possibly Unrelated | + | - | ± | ± | ？ |
| Definitely Unrelated | - | - | + | - | - |
| **Note:** “+” indicates affirmative; “-” indicates negative; “±” indicates uncertain; “?” indicates unknown. | | | | | |

### Recording, Management, and Follow-Up of Adverse Events

The investigator should carefully observe any adverse events that occur in subjects during the clinical study and ask subjects to honestly report any changes in their condition after taking the medication, avoiding leading questions. While observing efficacy, the investigator should also pay attention to adverse events or unexpected side effects (including symptoms, signs, and laboratory findings). All adverse events, regardless of whether they are related to the investigational drug, should be documented in detail in the eCRF, including the time of onset, symptoms, signs, severity, duration, laboratory test results, treatment methods, course of events, outcomes, and follow-up times. Information about concomitant medications should also be recorded in detail to facilitate analysis of the relationship between the adverse events and the investigational drug. Documentation should be signed and dated.

When an adverse event is identified during the trial, the investigator may take necessary actions based on the clinical situation, such as adjusting the dose or temporarily interrupting the medication, and decide whether to terminate the trial. In the case of a serious adverse event, the sponsoring institution must immediately take necessary actions to ensure the safety of the subjects.

All adverse events should be tracked until they return to normal or baseline levels to ensure the safety of the subjects. If an adverse event or its sequelae persist, follow-up should continue until the results are normal or the clinical manifestations stabilize. The follow-up method may vary based on the severity of the adverse event and can include hospitalization, outpatient visits, home visits, phone calls, or correspondence.

**Adverse events should be recorded starting from the time the subject signs the informed consent form.**

## Serious Adverse Events

Serious Adverse Event Handling Process


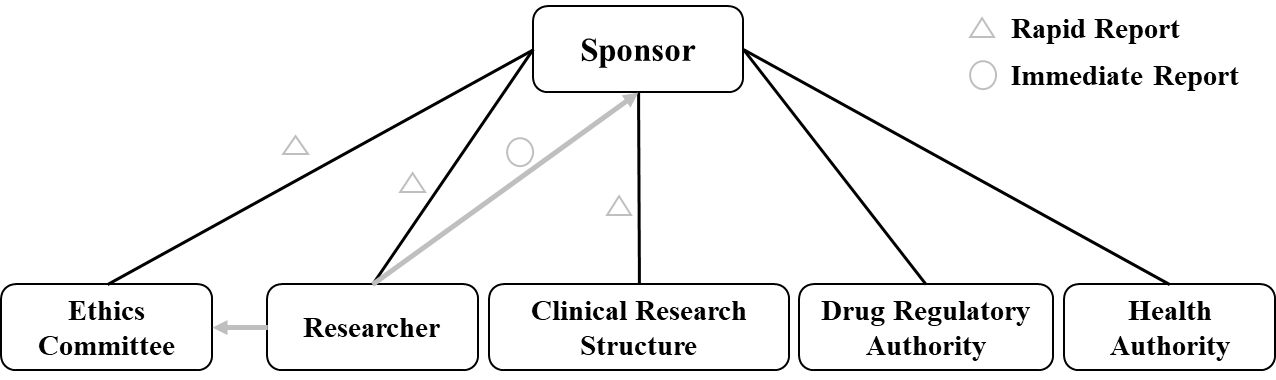


According to the requirements of the 2020 version of GCP, the handling process for SAEs is illustrated in the diagram.

Except for serious adverse events that do not require immediate reporting as specified in the study protocol or other documents (such as the investigator's brochure), the investigator must submit a written report of all SAEs to the sponsor within 24 hours of the event's occurrence or upon becoming aware of the event. For reports involving death, the investigator should provide the sponsor and the ethics committee with any additional necessary information, such as autopsy reports and final medical reports.

Upon receiving any safety-related information from any source, the sponsor must promptly analyze and assess it, including its severity, relevance to the investigational drug, and whether it is an anticipated event. The sponsor should report any suspected unexpected serious adverse reactions (SUSARs) to all investigators participating in the clinical trial, as well as to the regulatory authorities and health departments.

Investigators who receive safety information related to the clinical trial from the sponsor should promptly acknowledge receipt and read it. They should consider whether to make any necessary adjustments to the treatment of the subjects and communicate with the subjects as soon as possible if necessary. Additionally, they should report the SUSARs provided by the sponsor to the ethics committee.

Handling Measures: In the event of an emergency involving a patient, the investigator at the trial site should take appropriate actions based on the medication and the symptoms presented, and notify the clinical monitor of the handling results. The investigator should document the cause, date, actions taken, outcomes, and sign the SAE report form in detail.

Follow-up on Unresolved Adverse Events: All adverse events should be tracked until they are adequately resolved or the condition stabilizes.

## Anticipated/Unexpected Adverse Events

### Anticipated Adverse Event

Based on the preclinical safety data of this drug and clinical experience with similar HDAC inhibitors, the anticipated adverse events may include:

1. Gastrointestinal abnormalities: nausea, vomiting, diarrhea, bloating, decreased appetite
2. Blood and lymphatic system abnormalities: thrombocytopenia, neutropenia, lymphopenia
3. Cardiac abnormalities: prolonged QTc interval, tachycardia, arrhythmia
4. Hepatobiliary abnormalities: elevated ALT, AST, ALP, TBIL
5. Respiratory abnormalities: dyspnea, pulmonary edema
6. Metabolic abnormalities: hyperglycemia, hyperuricemia, hypophosphatemia, hyponatremia, hypokalemia
7. Psychiatric abnormalities: insomnia
8. Neurological abnormalities: headache, dizziness
9. Vascular abnormalities: hypotension
10. Renal function abnormalities: elevated SCr
11. Infections, pneumonia
12. Fatigue, weakness, fever
13. Weight loss
14. Hemorrhage

### Unexpected Adverse Events

An unexpected adverse event refers to an adverse medical event that occurs during the trial and is not explicitly mentioned in the study protocol, informed consent document, investigator's brochure, or drug usage or packaging information.

## Risk Control Plan

According to Section 8.3.1, reference should be made to similar drugs and the main anticipated adverse events that may occur with this product, along with the implementation of certain risk control measures.

**Hematologic toxicity**

Hematological adverse reactions (thrombocytopenia, neutropenia, anemia) commonly occur during the early stages of treatment. Close monitoring of complete blood count (CBC) is essential, and adjustments to medication and management of related side effects should be made based on the results, ensuring safety while striving to maintain the same dosage.

For grade 1–3 neutropenia, the treatment regimen should not be changed temporarily, and granulocyte colony-stimulating factor (G-CSF) should be added. CBC should be conducted approximately every two days to monitor whether neutrophil counts recover. If there is no recovery, consideration may be given to adjusting the treatment plan. If grade 4 neutropenia persists for≥5 days, despite the addition of G-CSF, Purinostat Mesylate treatment should be permanently discontinued unless the investigator and sponsor agree to continue treatment at a reduced dose in the best interest of the subject.

Patients with multiple myeloma often present with thrombocytopenia and anemia. Close monitoring of platelet counts is essential. For grade 1–3 thrombocytopenia, it is recommended to maintain the dose under supportive treatment with recombinant human thrombopoietin injection (e.g., TPO), recombinant human interleukin-11, or platelet transfusions to prevent life-threatening or severe gastrointestinal or pulmonary bleeding. If bleeding occurs, treatment should be immediately suspended until platelet counts return to baseline levels. If the patient experiences severe anemia (e.g., hemoglobin<60 g/L), red blood cell transfusions or erythropoietin injections may be used to increase red blood cell counts.

**Liver Toxicity**

In the event of liver toxicity, additional clinical information should be collected to determine the most likely cause of the observed liver abnormalities. This includes identifying abnormalities in liver function indicators that may be due to other factors, such as concomitant medications, alcoholic and autoimmune hepatitis, tumor metastasis, disease progression, viral hepatitis, cardiovascular disease, and other causes. The following measures should be taken:

Grade 2 Toxicity

- Continue to closely monitor liver function indicators during the Purinostat Mesylate treatment cycle until they return to baseline levels.
- For patients with baseline normal liver function who experience a Grade 2 increase in ALT or AST after treatment, suspend treatment until liver function returns to Grade 1 or below. Upon resuming treatment, the dose should be reduced by one level, and the frequency of liver function monitoring should be increased.

Grade 3 Toxicity

- If ALT or AST> 5×ULN, treatment should be discontinued, and the subject should exit the study. The subject will receive a consultation from a liver disease specialist.
- Closely monitor liver function indicators. If liver function is severely impaired relative to baseline values or reaches the level of severe liver injury, the medication should be discontinued, and supportive care should be strengthened, such as bed rest and close monitoring of liver function indicators, until liver function returns to baseline levels.
- During the process of reducing the dose by one level, if Grade 2 or higher toxicity recurs, treatment should be discontinued, and the subject should exit the study.

Grade 4 Toxicity

- Treatment should be discontinued, and the subject should exit the study. The subject will receive a consultation from a liver disease specialist.

**Cardiac Toxicity**

Grade 2 Toxicity

- Suspend treatment and increase the frequency of ECG monitoring until QTc<450 ms.
- Correct any electrolyte imbalances and discontinue any medications that may prolong the QTc interval.
- If toxicity can be restored to Grade 1 or below within 14 days, reduce the dose for treatment and continue to increase the frequency of ECG monitoring; otherwise, treatment should be discontinued, and the subject should exit the study.
- If Grade 2 toxicity recurs, treatment should be discontinued, and the subject should exit the study.

≥ Grade 3 Toxicity

- Treatment should be discontinued, and the subject should exit the study. The subject will receive a consultation from a cardiology specialist.

**Diarrhea**

Inform the subject to closely monitor for symptoms of enteritis (such as diarrhea, abdominal pain, bloody stools, or fever). If diarrhea occurs, it is recommended that the subject drink 8-10 cups of clear water daily to stay hydrated. Antidiarrheal medication (such as loperamide) may be administered until symptoms resolve. If diarrhea persists for more than 48 hours, consideration should be given to administering prophylactic antibiotics.

Grade 3 Toxicity

- Upon first occurrence of Grade 3 toxicity, treatment should be suspended, and antidiarrheal medications should be administered until toxicity resolves to Grade 1 or below. Once resolved, the medication can be resumed at the originally planned dose.
- If, after resuming treatment, Grade 3 toxicity occurs again, treatment should be suspended, and antidiarrheal medications should be administered until toxicity resolves to Grade 1 or below. Upon resolution, the dose should be reduced to the previous level.

Grade 4 Toxicity

- Upon first occurrence of Grade 4 toxicity, treatment should be suspended, and antidiarrheal medications should be administered until toxicity resolves to Grade 1 or below. Once resolved, the dose should be reduced to the previous level.
- If, after resuming treatment, Grade 4 toxicity occurs again, treatment should be discontinued, and the subject should exit the study.

**Nausea/Vomiting**

If the subject experiences vomiting symptoms for the first time, antiemetic medications may be administered for treatment.

Grade 3-4 Toxicity

- Upon first occurrence of Grade 3 or 4 toxicity, treatment should be suspended, and antiemetic medications should be administered until toxicity resolves to Grade 1 or below. Once resolved, the medication can be resumed at the originally planned dose, and it is recommended to use antiemetics as a preventive measure before dosing.
- If, after resuming treatment, Grade 3 or higher toxicity occurs again, treatment should be suspended, and antiemetic medications should be administered until toxicity resolves to Grade 1 or below. Upon resolution, the dose should be reduced to the previous level.

# Data Management

This trial employs electronic data management using DAS for EDC (V6.0). The main data management processes are outlined below, with further details available in the Data Management Plan (DMP).

The DMP, which serves as the guiding document for data management, is written by the Data Manager (DM) and approved by the sponsor. Data management activities will be conducted according to the timelines, content, and methods defined in the DMP.

## EDC Data Management

- Electronic Case Report Form (eCRF): The data manager designs and constructs the eCRF based on the study protocol and sets logical checks according to the Data Verification Plan (DVP). After testing and obtaining approval from the sponsor, the eCRF is released for use.
- Data Entry: eCRF data is sourced from original records. Data entry personnel enter the subject visit data into the Electronic Data Capture (EDC) system in a timely manner, following the eCRF completion guidelines.
- Source Data Verification (SDV): Monitors verify the consistency between eCRF data and source data. Any discrepancies may lead to queries.
- Data Queries and Responses: Queries arise from system-generated questions during EDC logical checks, as well as from manual queries by monitors and data managers. Researchers are required to respond to queries in a timely manner. Data managers and monitors will address the queries, and if necessary, additional queries may be issued until the data is deemed "clean."
- Investigator Signature: After data entry is completed and Source Data Verification (SDV) is performed, the investigator conducts an electronic signature review for confirmation. If there are any data revisions after signing, a new signature is required.
- Database Locking: The database lock record is signed by the principal investigator, the sponsor, the statistical analysts, and the data management personnel. Following this, the data manager locks the database.
- Database Submission: The data manager submits the database to the statistical personnel.
- eCRF Archiving: A PDF electronic document of the eCRF for each subject is generated and saved.
- Data Management Report: A report is written by the data manager.
- EDC Closure: After the statistical analysis is completed, the data manager closes the database.

## External Data Transfer

Sign the external data transfer agreement and manage external data according to the Data Management Plan (DMP).

## Medical Coding

Adverse events are coded using the MedDRA dictionary (version 22.0 or above), while concomitant medications are classified using the latest version of the WHO ATC classification.

# Statistical Analysis

## Analysis Dataset

**Safety Analysis Set (SS)**

The safety analysis population is defined as all patients who received at least one dose of the investigational drug and have at least one safety assessment data point. The SS set is the dataset used for the safety analysis.

**Full Analysis Set (FAS)**

All participants who entered this study and received at least one dose of the investigational drug, along with at least one efficacy assessment data point excluding baseline, will be included in the FAS (Full Analysis Set).

**Pharmacokinetic concentration Set (PKCS) :**

All enrolled participants in this study have received at least one dose of the investigational drug and have at least one valid concentration data point for the active ingredient after treatment during the trial period.

**Pharmacokinetic Parameter Set (PKPS):**

All participants enrolled in this study have received at least one dose of the investigational drug and have at least one valid pharmacokinetic (PK) parameter during the trial period. Participants who have serious protocol violations that impact the PK parameter results will not be included in the PK Population Set (PKPS).

**DLT Analysis Set**

The DLT analysis set includes participants who received at least 80% of the planned dosage within 21 days after the first dose, or who experienced a DLT after receiving at least one dose. This analysis set will be used for the analysis and summary of DLT events.

## Statistical Methods

The statistical analysis of this study will be conducted using the SAS 9.4 statistical software (or a later version). The results will be reported in summary tables (or graphs) and listings, primarily utilizing descriptive statistical methods for summarization. Numerical variables will be described using the mean, standard deviation (SD), median, first quartile (Q1), third quartile (Q3), minimum (Min), and maximum (Max). Categorical variables will be described using counts and percentages for each category.

## Baseline Characteristics of Participants

Demographic information for all participants during the screening period will be provided, including data on age, sex, medical history, treatment history, histopathology, lesion characteristics, and screening eligibility criteria. This baseline information will be summarized and categorized by dose group.

## Tolerability/Safety Analysis

In the safety analysis, adverse events/adverse reactions will be summarized by dose group using qualitative descriptions of the number of occurrences and incidence rates after medical coding is completed. A listing will be provided that includes information on the names of adverse events/adverse reactions, standard names, system classifications, occurrence times, severity, relationship to the investigational drug, whether they are serious adverse events, and outcomes.

Clinical significance of laboratory tests, results of physical examinations, and electrocardiogram (ECG) results will be analyzed using cross-tabulations for changes at each visit stage relative to pre-treatment values. A listing will also be provided for results that were normal/clinically insignificant prior to treatment but became abnormal/clinically significant afterward.

Key laboratory parameters, vital signs, ECOG scores, 12-lead ECGs, and cardiac enzyme profiles will be summarized quantitatively for the values at each visit, and line graphs will be created for the key parameters.

A summary of DLT events for each dose will also be provided.

## Pharmacokinetic Analysis

The blood concentration-time curves for each subject will be plotted for each time point. Based on the blood concentration-time data, pharmacokinetic parameters will be calculated using the standard non-compartmental model method in the WinNonlin software. The main pharmacokinetic parameters obtained will include T_max_, C_max_, AUC_0-t_, AUC_0-∞_, t_1/2_, MRT, CL, Ke, and V. Descriptive statistics will be used to list the mean, standard deviation, median, maximum, and minimum for the continuous data.

Dose proportionality analysis will be performed using single-dose pharmacokinetic parameters (PKPS) and analyzed with a power function model to examine the relationship between AUC, C_max_, and dose. The linearity criterion will be that the 95% confidence interval for the β value includes 1.

Accumulation will be assessed by comparing Cmax and AUC_SS_ from multiple dosing with C_max_ and AUC_0-48h_ from single dosing to calculate the accumulation ratio.。

## Clinical Efficacy Analysis

- Use FAS Analysis:
- Objective Response Rate (ORR): Calculate the ORR and its 95% confidence interval.
- Disease Control Rate (DCR): Calculate the DCR and its 95% confidence interval.
- Progression-Free Survival (PFS): The time from the start of the study to the date of the first recorded progression (PD) or death, whichever occurs first; if no disease progression is observed, the cutoff date should be the date of the last tumor measurement. PFS will be summarized using Kaplan-Meier plots, and the rates of censoring, quartiles, and their 95% confidence intervals will be listed, along with estimates of progression-free survival rates at different time points and their 95% confidence intervals.

## Efficacy Endpoint Analysis

Using the baseline at 0.5 hours before administration, the time-dependent changes in histone acetylation levels will be described for each dose group at various time points.

Exploratory analyses will be tentatively conducted to assess the relationships between pharmacodynamic (PD) endpoints and drug exposure, as well as between PD endpoints and efficacy. This includes examining the relationship between blood drug concentration and changes in pharmacodynamic values, the relationship between maximum PD changes, area under the curve (AUC), and dose, as well as the relationship between maximum PD changes, AUC, and efficacy.

## Statistical Software

Pharmacokinetic (PK) parameters will be calculated using WinNonlin (version 8.1 or higher), while other analyses will be conducted using SAS software (version 9.4 or higher).

Detailed statistical methods will be provided in the statistical analysis plan.

## Interim analysis

This study will not conduct an interim analysis.

# Trail management

## Modifications to the Study Protocol

Neither the investigator nor the sponsor may modify the study protocol without the consent of the other party. Any modifications to the protocol that could affect the conduct of the study, patient benefits, study objectives, study design, sample size, or study procedures must be presented in a protocol amendment. These amendments must be approved by the sponsor, the investigator, and the ethics committee before they can be implemented.

## Completion of the Electronic Case Report Form (eCRF)

The investigator will first summarize the general information about the subjects according to the content of the "Screening Form" to determine whether the subjects are eligible for enrollment. After enrollment, treatment and assessments will be conducted as required by the protocol, and relevant information will be recorded in the hospital medical records or original data. Subsequently, the data required by the protocol, including hospital records and test results, will be entered into the eCRF.

During the study, the Clinical Research Associate (CRA) will perform source data verification (SDV) comparing the original data with the data in the Electronic Data Capture (EDC) system. Any data that significantly deviates from clinically acceptable ranges must be verified and explained. Suspect data will prompt online queries, which the investigator or Clinical Research Coordinator (CRC) will verify against the original data and make corrections in the EDC. Throughout the study, the investigator should allow the sponsor’s monitors or representatives to inspect study-related documents at the research site (such as patient informed consent forms, study drug accountability logs, and ethics committee approval documents). When monitors visit, they will conduct a thorough review of patient records to verify the information recorded in the hospital medical records or eCRF, particularly key information related to safety and efficacy.

## Data Quality Assurance

To ensure the integrity, accuracy, and reliability of the data, the following measures have been implemented in this study:

1. Selection of qualified and experienced research institutions and investigators;
2. Providing detailed information about the study protocol to investigators through lectures and written materials before the study begins, and collaboratively developing solutions for potential issues;
3. Regular verification of the authenticity, accuracy, and completeness of the data by monitors, data managers, and other personnel;
4. If any data discrepancies are identified, timely communication with the investigators should occur for confirmation or correction.

## Record Retention

The research institution will retain the hospital medical records and other original data for each subject. After the study concludes, the data management team will export the finalized eCRF data from the Electronic Data Capture (EDC) system and transfer it to the sponsor. All records will be maintained for a minimum of five years after the completion of the clinical trial.

## Monitoring

Throughout the clinical trial, the Contract Research Organization (CRO) for this project will designate qualified monitors to conduct regular on-site monitoring of the clinical trial centers to ensure strict compliance with all aspects of the study protocol. The trial centers should assist and cooperate with the monitors in their work.

The main tasks of the monitoring process include:

1. Confirm that the trial site has appropriate conditions before the clinical trial begins, including personnel staffing and training, laboratory equipment that is complete and operational, all necessary testing conditions related to the trial, familiarity with the requirements of the study protocol, and sufficient numbers of researchers participating in the study.
2. Monitor the investigator's adherence to the study protocol during the trial, ensuring that informed consent has been obtained from all subjects prior to their enrollment, understanding the subjects' enrollment rates and the progress of the trial, and confirming the eligibility of enrolled subjects.
3. Ensure that all data is recorded and reported accurately and completely, with all electronic case report forms entered correctly and consistent with the original data. Any errors or omissions should be corrected or noted. Changes in dosage, treatment modifications, concomitant medications, adverse events, lost to follow-up, and missed assessments for each subject must be confirmed and recorded. Verify that the reasons for withdrawal or loss to follow-up for enrolled subjects are documented in the electronic case report forms.
4. Confirm that all adverse events are recorded, serious adverse events are reported and documented within the specified time frame; verify that trial medications are supplied, stored, distributed, and retrieved in accordance with relevant regulations, with appropriate records maintained.
5. Assist the investigator with necessary notifications and applications, and report trial data and results to the sponsor.
6. Clearly and accurately document any follow-ups that the investigator failed to perform, any tests that were not conducted, any assessments that were not completed, and whether any errors or omissions were corrected.
7. Complete a written monitoring report after each visit, including the date and time of the monitoring visit, the name of the monitor, and the results of the monitoring.

## Audits and Inspections

Throughout the clinical trial process or after its completion, the sponsor, Chengdu Zenitar Biomedical Technology Co., Ltd, the CRO, government regulatory authorities, and independent ethics committees have the right to conduct audits or inspections at the trial centers, which must cooperate with these activities.

The main tasks of the audits include: ensuring the existence of all documentation required by Good Clinical Practice (GCP); verifying original data against eCRF data; inspecting instruments and equipment; reviewing the storage and management of trial medications; and assessing the fulfillment of the monitor's responsibilities, among other tasks.

## Study Completion/Termination

### Study Completion

The study is considered complete after the last participant's follow-up has concluded, at which point the investigator should notify the sponsor.

### Study Termination

Study termination refers to the premature cessation of the clinical trial before it is completed according to the protocol. The primary purpose of terminating a study is to protect the rights of participants, ensure the quality of the trial, and avoid unnecessary economic loss. All parties involved in the study should be promptly notified of an early termination. Reasons for terminating the study include, but are not limited to:

1. Significant errors are found in the clinical trial protocol that make it difficult to assess the safety of the drug;
2. Concerns related to safety;
3. The sponsor requests termination (e.g., for reasons related to funding, management, etc.);
4. The National Medical Products Administration or ethics committee mandates the termination of the study for any reason.。

## Use and Publication of Information

All information and data related to this trial are confidential and are the property of the sponsor. Except as required by the National Medical Products Administration, the investigator may not disclose any information to third parties in any form without the written consent of the sponsor, nor may this information be used for any other purposes.

The investigator is required to provide all data obtained during the trial to the sponsor. For the protection of legitimate business secrets, the investigator may not publish research results without prior review and approval from the sponsor.

## Responsibilities of All Parties

The investigator must read and understand the content of the study protocol in detail and execute the trial in strict accordance with the "Good Clinical Practice" (GCP) guidelines and the protocol. They are responsible for recording the participants' original data, obtaining informed consent, promptly entering data into the electronic case report forms, and documenting and counting the investigational medication. The investigator should accept regular monitoring by the monitors to ensure the quality of the clinical trial and ensure that participants receive appropriate treatment in the event of adverse events during the trial. After the clinical trial concludes, the investigator should review and finalize the summary report, sign it, and date it.

The sponsor is responsible for initiating the clinical trial and providing funding. The Contract Research Organization (CRO) collaborates with the sponsor to organize, monitor, and audit the clinical trial. The sponsor is also responsible for providing the investigational drug to the investigator and ensuring its quality. Additionally, the sponsor is responsible for submitting the trial summary report to the National Medical Products Administration.

# Ethical Principles

## Responsibilities of the Investigator

The investigator is responsible for ensuring compliance with the Declaration of Helsinki and the Chinese Good Clinical Practice (GCP) guidelines, as well as relevant laws and regulations, while executing this study protocol.

## Approval of the Ethics Committee

Before the trial begins, the investigator must provide relevant materials as required by the ethics committee to obtain their approval.

This trial may only commence after receiving written approval from the ethics committee. If the study protocol needs to be revised during the course of the research, the revised protocol must be submitted to the ethics committee of the sponsoring institution for filing/approval before implementation. If important new information regarding the investigational drug is discovered, the relevant materials must be modified in writing and submitted to the ethics committee for approval, and informed consent from the participants must be obtained again. At the conclusion of the trial, the investigator should notify the ethics committee that the trial is completed.

## Informed Consent

Before enrolling in the trial, the investigator should provide the participant or their legal representative with a complete and thorough explanation of the purpose, methods, procedures, potential benefits, and possible risks of the trial, and this information should be documented in writing. Only after obtaining the participant's consent and voluntarily signing the informed consent form can they be enrolled in the clinical trial. The participant retains a copy of the informed consent form.

- Information to be Provided to Participants

1. Participation in the trial is voluntary, and participants have the right to withdraw from the trial at any stage without discrimination or retaliation, and their medical treatment and rights will not be affected.
2. The personal information of participants in the trial is confidential. Only the regulatory authorities, ethics committee, or sponsor may access the data of participants when necessary.
3. Participants will be informed about the purpose of the trial, the methods, procedures, and duration, as well as the expected potential benefits and risks.
4. In the event of any trial-related harm, participants may receive appropriate treatment and compensation.

- Important Considerations for Signing the Informed Consent Form

1. The investigator must provide a thorough and detailed explanation of the trial to the participant and allow sufficient time for the participant to consider whether they wish to participate in the study. The informed consent form should only be signed after obtaining the participant's full consent.
2. The informed consent form must be signed and dated by the participant or their legal representative. The investigator or their representative who conducts the informed consent process must also sign and date the form.
3. The informed consent process should be conducted in a language and manner that the participant or their legal representative can understand. Throughout the trial, the participant has the right to access any information or materials related to the study at any time.
4. If the participant or their legal representative is unable to read, a witness must be present throughout the process. After the informed consent form is explained in detail, the participant or their legal representative may provide verbal consent, which must be signed and dated by the witness.
5. For participants who are legally incompetent, if the ethics committee gives its principal approval and the investigator believes that participation in the clinical trial is in the participant's best interest, these patients may also be included in the study, provided their legal representative signs and dates the consent form.
6. If the informed consent form is not signed by the participant, legal representative, or witness, the investigator must document the situation and the reasons for the inability to obtain consent, and sign this record.

# References

1. Parkin, D.M., et al., *Global cancer statistics, 2002.* CA Cancer J Clin, 2005. **55**(2): p. 74-108.2. Ferlay, J., et al., *Cancer incidence and mortality worldwide: sources, methods and major patterns in GLOBOCAN 2012.* Int J Cancer, 2015. **136**(5): p. E359-86.

3. Global Burden of Disease Cancer, C., et al., *Global, Regional, and National Cancer Incidence, Mortality, Years of Life Lost, Years Lived With Disability, and Disability-Adjusted Life-years for 32 Cancer Groups, 1990 to 2015: A Systematic Analysis for the Global Burden of Disease Study.* JAMA Oncol, 2017. **3**(4): p. 524-548.

4. Torre, L.A., et al., *Global cancer statistics, 2012.* CA Cancer J Clin, 2015. **65**(2): p. 87-108.

5. Rickert, R.C., *New insights into pre-BCR and BCR signalling with relevance to B cell malignancies.* Nat Rev Immunol, 2013. **13**(8): p. 578-91.

6. Armitage, J.O., et al., *Non-Hodgkin lymphoma.* The Lancet, 2017. **390**(10091): p. 298-310.

7. Kridel, R., L.H. Sehn, and R.D. Gascoyne, *Pathogenesis of follicular lymphoma.* J Clin Invest, 2012. **122**(10): p. 3424-31.

8. Schmitz, R., et al., *Oncogenic mechanisms in Burkitt lymphoma.* Cold Spring Harb Perspect Med, 2014. **4**(2).

9. Fiskus, W., et al., *Superior efficacy of a combined epigenetic therapy against human mantle cell lymphoma cells.* Clin Cancer Res, 2012. **18**(22): p. 6227-38.

10. Tibiletti, M.G., et al., *BCL2, BCL6, MYC, MALT 1, and BCL10 rearrangements in nodal diffuse large B-cell lymphomas: a multicenter evaluation of a new set of fluorescent in situ hybridization probes and correlation with clinical outcome.* Hum Pathol, 2009. **40**(5): p. 645-52.

11. Esteve-Arenys, A., et al., *The BET bromodomain inhibitor CPI203 overcomes resistance to ABT-199 (venetoclax) by downregulation of BFL-1/A1 in in vitro and in vivo models of MYC+/BCL2+ double hit lymphoma.* Oncogene, 2018.

12. Downing, J.R. and K.M. Shannon, *Acute leukemia: a pediatric perspective.* Cancer Cell, 2002. **2**(6): p. 437-45.

13. Seifert, M., et al., *Cellular origin and pathophysiology of chronic lymphocytic leukemia.* J Exp Med, 2012. **209**(12): p. 2183-98.

14. Paton-Hough, J., A.D. Chantry, and M.A. Lawson, *A review of current murine models of multiple myeloma used to assess the efficacy of therapeutic agents on tumour growth and bone disease.* Bone, 2015. **77**: p. 57-68.

15. Morelli, E., et al., *Selective targeting of IRF4 by synthetic microRNA-125b-5p mimics induces anti-multiple myeloma activity in vitro and in vivo.* Leukemia, 2015. **29**(11): p. 2173-83.

16. Rollig, C., S. Knop, and M. Bornhauser, *Multiple myeloma.* Lancet, 2015. **385**(9983): p. 2197-208.

17. Molyneux, E.M., et al., *Burkitt's lymphoma.* The Lancet, 2012. **379**(9822): p. 1234-1244.

18. West, A.C. and R.W. Johnstone, *New and emerging HDAC inhibitors for cancer treatment.* J Clin Invest, 2014. **124**(1): p. 30-9.

19. Giannini, G., et al., *Histone deacetylase inhibitors in the treatment of cancer: overview and perspectives.* Future Medicinal Chemistry, 2012. **4**(11): p. 1439-1460.

20. Spiegel, S., S. Milstien, and S. Grant, *Endogenous modulators and pharmacological inhibitors of histone deacetylases in cancer therapy.* Oncogene, 2012. **31**(5): p. 537-51.

21. Dawson, M.A. and T. Kouzarides, *Cancer epigenetics: from mechanism to therapy.* Cell, 2012. **150**(1): p. 12-27.

22. Shen, H. and P.W. Laird, *Interplay between the cancer genome and epigenome.* Cell, 2013. **153**(1): p. 38-55.

23. Stewart, A.K., *Novel therapies for relapsed myeloma.* Hematology Am Soc Hematol Educ Program, 2009: p. 578-86.

24. Schrump, D.S., *Cytotoxicity mediated by histone deacetylase inhibitors in cancer cells: mechanisms and potential clinical implications.* Clin Cancer Res, 2009. **15**(12): p. 3947-57.

25. Valenzuela-Fernandez, A., et al., *HDAC6: a key regulator of cytoskeleton, cell migration and cell-cell interactions.* Trends Cell Biol, 2008. **18**(6): p. 291-7.

26. Bolden, J.E., M.J. Peart, and R.W. Johnstone, *Anticancer activities of histone deacetylase inhibitors.* Nat Rev Drug Discov, 2006. **5**(9): p. 769-84.

27. Li, Y. and E. Seto, *HDACs and HDAC Inhibitors in Cancer Development and Therapy.* Cold Spring Harb Perspect Med, 2016. **6**(10).

28. Giannini, G., et al., *Histone deacetylase inhibitors in the treatment of cancer: overview and perspectives.* Future Med Chem, 2012. **4**(11): p. 1439-60.

29. Falkenberg, K.J. and R.W. Johnstone, *Histone deacetylases and their inhibitors in cancer, neurological diseases and immune disorders.* Nat Rev Drug Discov, 2014. **13**(9): p. 673-91.

30. Lu, X., et al., *Development of chidamide for peripheral T-cell lymphoma, the first orphan drug approved in China.* Intractable Rare Dis Res, 2016. **5**(3): p. 185-91.

31. Batlevi, C.L., et al., *A phase 2 study of mocetinostat, a histone deacetylase inhibitor, in relapsed or refractory lymphoma.* Br J Haematol, 2017. **178**(3): p. 434-441.

32. Schech, A., et al., *Histone Deacetylase Inhibitor Entinostat Inhibits Tumor-Initiating Cells in Triple-Negative Breast Cancer Cells.* Mol Cancer Ther, 2015. **14**(8): p. 1848-57.

33. Garmpis, N., et al., *Histone Deacetylases as New Therapeutic Targets in Triple-negative Breast Cancer: Progress and Promises.* Cancer Genomics Proteomics, 2017. **14**(5): p. 299-313.

34. Culjkovic-Kraljacic, B., et al., *Combinatorial targeting of nuclear export and translation of RNA inhibits aggressive B-cell lymphomas.* Blood, 2016. **127**(7): p. 858-68.

35. Giles, F., et al., *A phase I study of intravenous LBH589, a novel cinnamic hydroxamic acid analogue histone deacetylase inhibitor, in patients with refractory hematologic malignancies.* Clin Cancer Res, 2006. **12**(15): p. 4628-35.

36. Venugopal, B., et al., *A phase I study of quisinostat (JNJ-26481585), an oral hydroxamate histone deacetylase inhibitor with evidence of target modulation and antitumor activity, in patients with advanced solid tumors.* Clin Cancer Res, 2013. **19**(15): p. 4262-72.

# Appendix

## ECOG Performance Status Scoring Criteria

| **Grading** | **Performance Status** |
| --- | --- |
| 0 | Fully active, able to carry on all normal activities without restriction. |
| 1 | Restricted in physically strenuous activity but ambulatory and able to carry out work of a light or sedentary nature. For example, light housework, office work. |
| 2 | Ambulatory and capable of all self care but unable to carry out any work activities. Up and about more than 50% of waking hours. |
| 3 | Ambulatory and capable of all self care but unable to carry out any work activities. Up and about more than 50% of waking hours. |
| 4 | Completely disabled. Cannot carry on any self-care. Totally confined to bed or chair |
| 5 | Death. |

## New York Heart Association (NYHA) Functional Classification

Class I: Patients have heart disease but do not experience any limitations in physical activity. Ordinary physical activities do not cause fatigue, palpitations, shortness of breath, or angina.

Class II: Patients with heart disease have mild limitations in physical activity. They do not experience any symptoms at rest, but ordinary physical activities may lead to fatigue, palpitations, shortness of breath, or angina.

Class III: Patients with heart disease have marked limitations in physical activity. Symptoms occur with less than ordinary physical activity.

Class IV: Patients with heart disease are unable to engage in any physical activity. Symptoms of heart failure occur even at rest and are exacerbated by physical activity.

## Criteria for Mild Abnormal Laboratory Values

**Criteria for Mild Abnormal Laboratory Values - NCI CTCAE v5.0 Grade 1**

| Laboratory Test Item | Mildly Abnormal Criteria | Laboratory Test Item | Mildly Abnormal Criteria |
| --- | --- | --- | --- |
| Hb | < LLN - 100 g/L | Cr | > ULN – 1.5×ULN |
| WBC | < LLN - 3000/mm^3^ | Na | > ULN – 150 mmol/L  < LLN – 130 mmol/L |
| N | < LLN - 1500/mm^3^ | K | > ULN – 5.5 mmol/L  < LLN – 3.0 mmol/L |
| L | < LLN - 800/mm^3^ | Ca | > ULN – 2.9 mmol/L  < LLN – 2.0 mmol/L |
| Plt | < LLN - 75000/mm^3^ | Mg | > ULN – 1.23 mmol/L  < LLN – 0.5 mmol/L |
| BIL | > ULN - 1.5×ULN | P | < LLN – 0.8 mmol/L |
| ALT | > ULN - 2.5×ULN | Glu | > ULN – 8.9 mmol/L  < LLN – 3.0 mmol/L |
| AST | > ULN - 2.5×ULN | TG | > ULN – 2.5×ULN |
| ALP | > ULN - 2.5×ULN | CHOL | > ULN – 7.75 mmol/L |
| CPK | > ULN - 2.5×ULN | CK-MB | > ULN |
| LDH | > ULN - 2.5×ULN | Troponin I | > ULN |
| α-HDBD | > ULN - 2.5×ULN | CRP | > ULN |

## Criteria for the Evaluation of Multiple Myeloma

"Chinese Guidelines for the Diagnosis and Treatment of Multiple Myeloma (Revised in 2017)" and the 2016 International Myeloma Working Group (IMWG)

| **IMWG Response Criteria** | |
| --- | --- |
| **sCR** | The following definition of complete response includes a normal free light chain (FLC) ratio, and a bone marrow biopsy (after calculating ≥100 plasma cells) showing no clonal cells based on immunohistochemical examination (with κ and λ ratios of ≤4:1 or ≥1:2 for the patient). |
| **CR** | Serum and urine immunofixation electrophoresis negative, with no evidence of any soft tissue plasmacytomas, and plasma cells in the bone marrow aspirate <5%. |
| **VGPR** | Immunofixation electrophoresis may detect serum and urine M proteins, but electrophoresis is negative or serum M protein is reduced by ≥90% with urine M protein levels. |
| **PR** | - Serum M protein decreased by ≥50% and 24-hour urine M protein decreased by ≥90% or reached <200 mg/24 h. - If serum and urine M protein cannot be measured, a difference of ≥50% in free light chain (FLC) levels between the affected and unaffected areas can be used as a substitute for the M protein criteria. - If serum and urine M protein testing and serum free light chain testing cannot be performed, plasma cells must be reduced by ≥50% to substitute for the M protein criteria, provided that the baseline percentage of plasma cells in the bone marrow is ≥30%. In addition to these criteria, if present at baseline, the size of soft tissue plasmacytomas (the sum of the products of the maximum vertical diameters of multiple measurable lesions [SPD]) must decrease by ≥50%. |
| **MR** | Serum M protein decreased by ≥25% but ≤49%, and 24-hour urine M protein decreased by 50%-89%. In addition to the criteria listed above, if present at baseline, the size of soft tissue plasmacytomas (SPD) must decrease by ≥50%. |
| **SD** | It is not recommended to use this as a response indicator; the best description of disease stability should be "time to progression estimate." This does not meet the criteria for CR, VGPR, PR, MR, or PD. |
| **PD** | Meets any one or more of the following criteria:  Any one or more of the following criteria indicate a minimum confirmation of response value increase of 25%.   - Serum M protein (the absolute increase must be > 0.5 g/dL); - If the baseline minimum M component value is ≥5 g/dL, an increase in serum M protein of ≥1 g/dL; - Urine M protein (the absolute increase must be ≥200 mg/24 h); - For patients where serum and urine M protein levels cannot be measured, the difference in free light chain (FLC) levels between the affected and unaffected areas (the absolute increase must be >10 mg/dL); - For patients where serum and urine M protein levels cannot be measured and where the FLC levels in the affected area cannot be measured, the percentage of plasma cells in the bone marrow is not affected by baseline status (the absolute increase must be ≥10%); - New lesions appear, with the SPD of >1 lesion increasing by ≥50% from the lowest value, or the maximum diameter of the original lesion with a minimum diameter >1 cm increasing by ≥50%; if this is the only measurable value of the disease, the number of circulating plasma cells (at least 200 cells per μL) increases by ≥50%. |
| **Clinical relapse** | Clinical relapse requires one or more of the following criteria:   - Direct indicators of disease progression and/or end-organ dysfunction (such as elevated calcium levels, renal failure, anemia, and osteolytic lesions [CRAB symptoms]) associated with potential clonal plasma cell proliferation. This is not used to calculate time to progression or progression-free survival but can be selectively reported or used in clinical practice; - The occurrence of new soft tissue plasmacytomas or bone lesions (osteoporotic fractures do not constitute lesions); - A clear increase in the size of existing plasmacytomas or bone lesions. A clear increase is defined as a ≥50% increase in the SPD of measurable lesions (and ≥1 cm) after consecutive measurements; - Hypercalcemia (>11 mg/dL); - Hemoglobin decrease of ≥2 g/dL unrelated to treatment or other non-myeloma related symptoms; - An increase in serum creatinine of 2 mg/dL or more at the start of autonomous treatment, attributed to myeloma; - Hyperviscosity related to serum paraproteins. |
| **Relapse after complete response (used only when the endpoint of the study is disease-free survival)** | Meets any one or more of the following criteria:   - Immunofixation or electrophoresis shows reappearance of serum or urine M protein; - Development of bone marrow plasma cells ≥5%; - Appearance of any other progression markers (such as new plasmacytomas, osteolytic lesions, or hypercalcemia) (as mentioned above). |

*Note: CR = Complete Response, sCR = Strict Complete Response, PR = Partial Response, VGPR = Very Good Partial Response, MR = Minimal Response, SD = Stable Disease, PD = Progressive Disease.*

***Note: ORR=* sCR *+* CR *+* VGPR *+* PR *+* MR**

## Lymphoma Efficacy Evaluation Criteria

* For B-cell lymphoma and T-cell lymphoma, refer to the following criteria.

**International Working Group (IWG) Lymphoma Response Evaluation Criteria (2017)**

|  | **Complete Response（CR）** | **Partial Response（PR）** | **Minor Response（MR）** | **Stable Disease（SD）** | **Progressive Disease（PD）** |
| --- | --- | --- | --- | --- | --- |
| Change from baseline (%) | All target lesions completely disappear, and the longest diameter of all lymph nodes is < 10 mm; | The sum of the longest diameters of target lesions decreases by ≥30% (Partial Response), but does not reach Complete Response; | The sum of the longest diameters of target lesions decreases by ≥10%, but does not reach Partial Response; | The sum of the longest diameters of target lesions decreases by <10% or increases by ≤20%; | The sum of the longest diameters of target lesions increases by >20% |
|  | The sum of the longest diameters of target lesions decreases by ≥30% (Partial Response), while the FDG-PET scan results are normal; |  |  |  |  |
| FDG-PET CT | FDG-PET CT scan results are normal (Deauville score 1 to 3) | FDG-PET CT scan results are positive (Deauville score 4 to 5); | Any FDG-PET CT scan results | Any FDG-PET CT scan results | Any FDG-PET CT scan results |
| Bone marrow involvement | No bone marrow involvement; | Any lesions involving the bone marrow; | Any lesions involving the bone marrow; | Any lesions involving the bone marrow; | Any lesions involving the bone marrow; |
| New lesions | No new lesions. | No new lesions. | No new lesions. | No new lesions. | With or without new lesions. |

*Note: FDG-PET: 18F-fluorodeoxyglucose positron emission tomography.*

Note: ORR =CR+PR+MR; DCR= CR+PR+MR+SD

Reference: Younes A, Hilden P, Coiffier B, et al. International Working Group consensus response evaluation criteria in lymphoma (RECIL 2017) [J]. Annals of Oncology, 2017, 28(7): 1436-1447.

## Efficacy Evaluation Criteria for B-cell Acute Leukemia and T-cell Acute Leukemia

**Guidelines for the Diagnosis and Treatment of Adult Acute Lymphoblastic Leukemia in China (2016 Edition)**

1. **Bone Marrow and Peripheral Blood Efficacy Criteria**
2. CR: 1) No blasts in peripheral blood and no extramedullary leukemia; 2) Restoration of trilineage hematopoiesis in bone marrow, with blasts < 5%; 3) Peripheral blood ANC > 1.0 × 10^9/L; 4) Peripheral blood PLT > 100 × 10^9/L; 5) No relapse within 4 weeks.
3. CR with incomplete blood count recovery (CRi): PLT < 100 × 10^9/L and/or ANC < 1.0 × 10^9/L. All other criteria must meet those for CR.
4. a) Objective response rate (ORR) = CR + CRi. This disease does not conduct DCR analysis.
5. Refractory disease: Failure to achieve CR after the completion of induction therapy.
6. Progressive disease (PD): An increase of 25% in the absolute number of blasts in peripheral blood or bone marrow, or the emergence of extramedullary disease.
7. Disease relapse: In patients who have achieved CR, the reappearance of blasts in peripheral blood or bone marrow (proportion > 5%) or the emergence of extramedullary disease.
8. **Treatment Response for CNS Disease**
9. CNS Remission: Patients with CNS-2 or CNS-3 status achieve CNS-1 status.
10. CNS Relapse: The emergence of CNS-3 status or clinical symptoms of CNSL (such as facial nerve paralysis, brain/eye involvement, or manifestations of hypothalamic syndrome).

**Ⅲ Treatment Response for Mediastinal Disease**

The assessment of efficacy for mediastinal disease relies on chest CT and PET-CT. Complete Response (CR): Complete disappearance of mediastinal enlargement on CT or a negative PET. Partial Response (PR): A reduction of more than 50% in the product of the maximum vertical diameter of the enlarged mediastinum (Sum of the Products of Diameters, SPD). Progressive Disease (PD): An increase of more than 25% in SPD. Stable Disease (SD): Fails to meet the criteria for partial response or progressive disease. Relapse: Patients who achieved CR and subsequently exhibit mediastinal enlargement again.

## Contraception

**（1） Definition of women of childbearing potential and contraceptive requirements for female subjects (and their male partners)**

Women who have not undergone surgical sterilization (i.e., have not had bilateral tubal ligation, bilateral oophorectomy, or total hysterectomy) and non-menopausal women are considered women of childbearing potential. Menopause is defined as the cessation of menstruation for ≥12 months without any other medical reasons.

Female subjects of childbearing potential must have a negative blood pregnancy test at screening. From the first administration of the drug until six months after the last dose, female subjects of childbearing potential must agree to take one of the following actions:

1. Complete abstinence. The use of periodic abstinence methods (such as the calendar method, ovulation method, symptoms-temperature method, or post-ovulation method) is not permitted.
2. In addition to the correct use of condoms by male partners, the following contraceptive methods may be used:

- Intrauterine device (IUD) with a failure rate of <1% per year
- Cervical cap or diaphragm with spermicide
- Tubal sterilization
- Male partner undergoing vasectomy
- Hormonal contraceptives
- Levonorgestrel implants
- Injectable progesterone
- Oral contraceptives (combined or progestin-only)
- Vaginal contraceptive ring
- Transdermal contraceptive patch

**（2） Contraceptive requirements for male subjects (and their female partners)**

All male subjects must agree to consistently use condoms correctly from the first administration of the drug until 3 months after the last dose. In addition to correct condom use, the female partners of male subjects of childbearing potential may also choose one of the aforementioned contraceptive methods.

**（3）Procedures to be followed in the event of pregnancy**

If a subject (or their partner) becomes unexpectedly pregnant during the trial or within six months after the last dose, they must inform the investigator of the pregnancy.

## WHO Bleeding Severity Grading (Revised Version)

| Grading | Bleeding characteristics |
| --- | --- |
| Grade 1 | 1. Localized: 1-2 independent ecchymoses/petechiae, or scattered/non-confluent. 2. Oral or nasal bleeding < 30 minutes. |
| Grade 2 | 1. Melena, hematemesis, hemoptysis, fresh blood in stool, musculoskeletal or soft tissue bleeding, with bleeding < 24 hours and no need for red blood cell transfusion, hemodynamically stable. 2. Skeletal or soft tissue bleeding. 3. Significant oral or nasal bleeding lasting > 30 minutes. 4. Oral vesicles with bleeding or symptoms of significant discomfort. 5. Multiple bruises, each > 2 cm, or one bruise > 10 cm. 6. Diffuse ecchymoses/petechiae. 7. Gross hematuria. 8. Abnormal bleeding from invasive or surgical sites. 9. Unexpected vaginal bleeding, with bleeding < 24 hours and blood soaking > 2 pads. 10. Visible hemorrhage in body cavities. 11. Retinal bleeding, but vision is not affected. |
| Grade 3 | 1. Bleeding<24 hours require red blood cell transfusion as a targeted treatment measure, with hemodynamic stability. 2. Severe hemorrhage in body cavities. 3. Computed tomography (CT) shows cerebral hemorrhage, but without neurological symptoms or signs. |
| Grade 4 | 1. Bleeding resulting in weakness, including retinal bleeding leading to vision impairment. * 2. Non-fatal cerebral hemorrhage with neurological symptoms and signs. 3. Hemodynamic instability due to bleeding (hypotension, decrease in systolic or diastolic blood pressure>30 mm Hg). 4. Fatal bleeding from any site. |

* Definition of visual impairment: Visual field defects; patients suspected of visual impairment require an ophthalmological consultation
