# Supplementary material for: Preclinical and first-in-human of purinostat mesylate, a novel selective HDAC I/IIb inhibitor, in relapsed/refractory multiple myeloma and lymphoma
Source: Signal Transduct Target Ther. 2025 Jun 23;10:201. doi: 10.1038/s41392-025-02285-w (PMC12198407; doi:10.1038/s41392-025-02285-w)

ALL original and uncropped films of Western blots of Fig. 2b, Supplementary Fig. 2e, and Supplementary Fig. 7d.


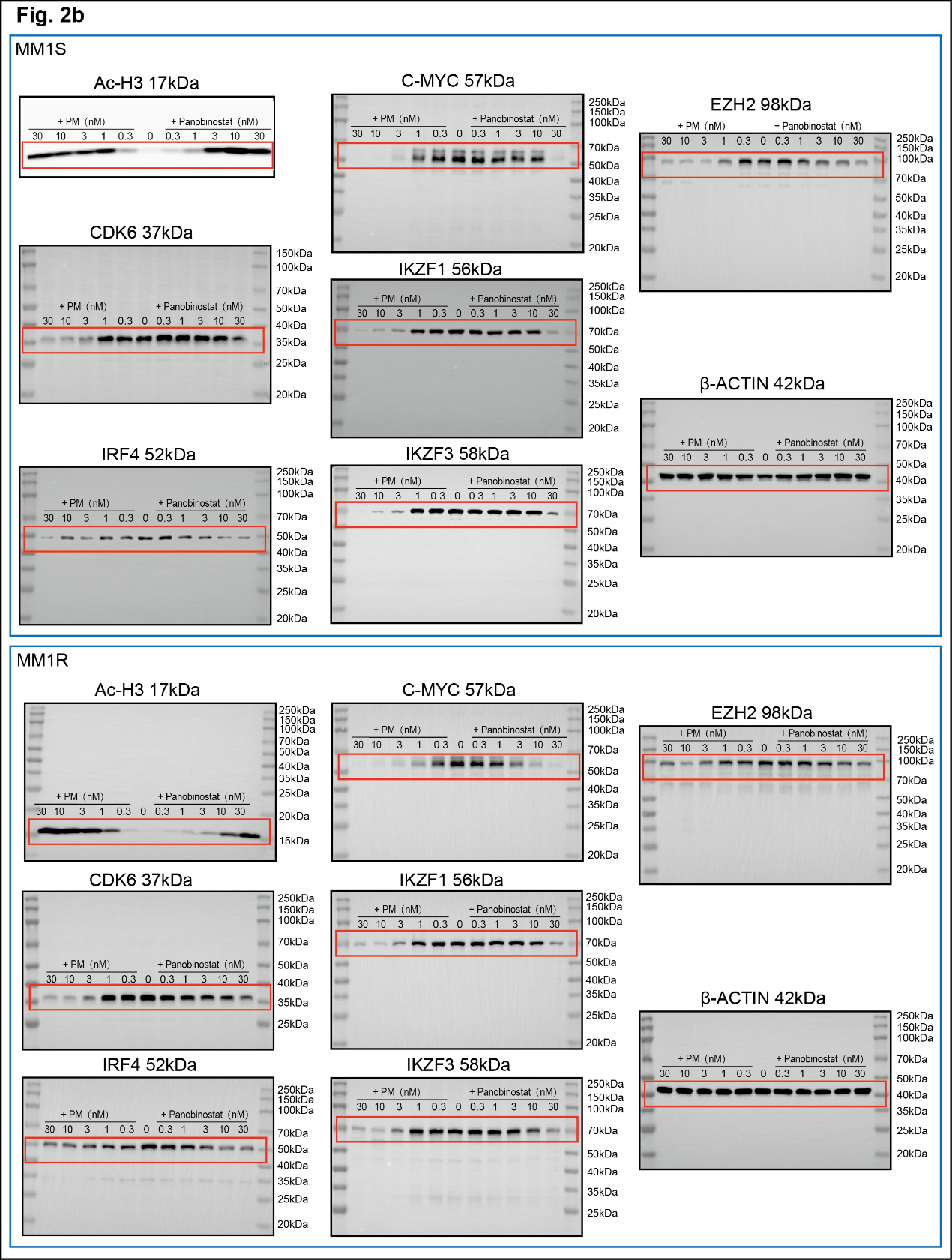


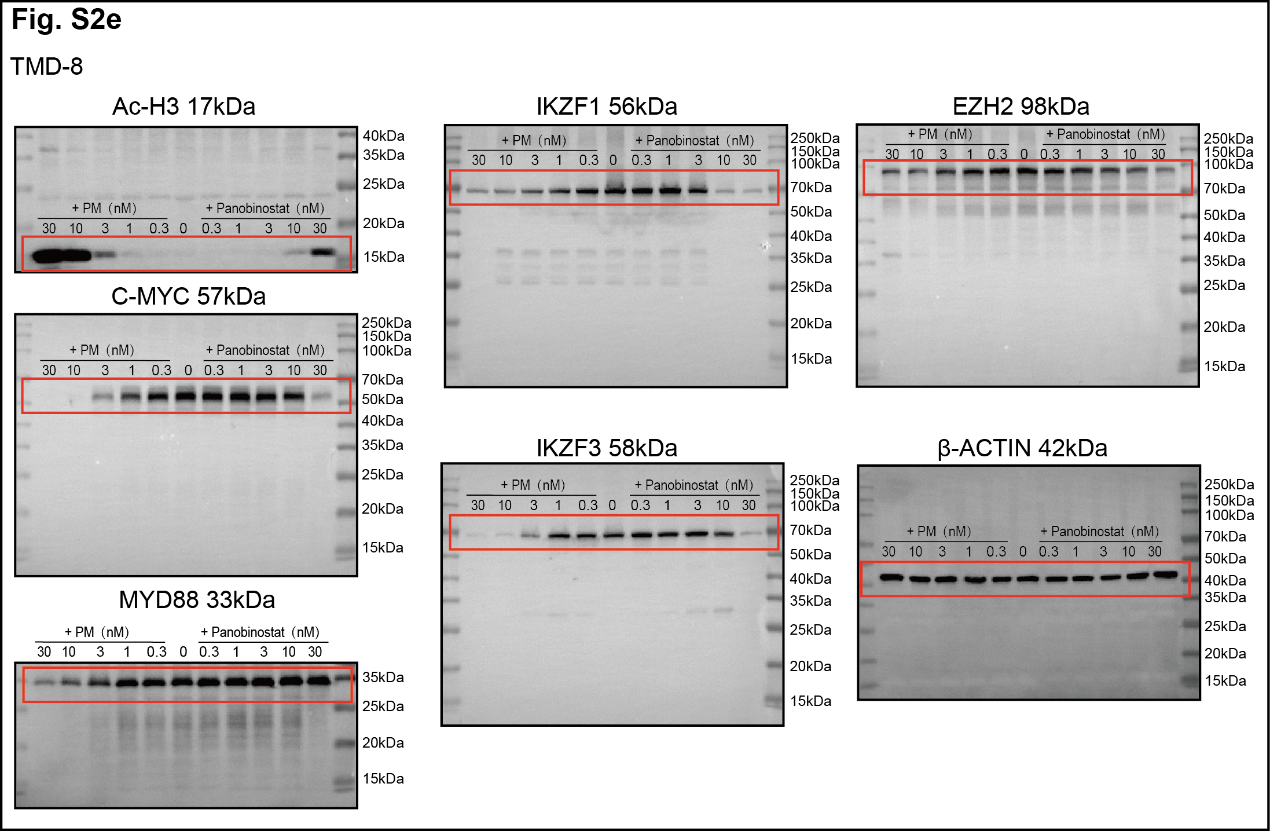


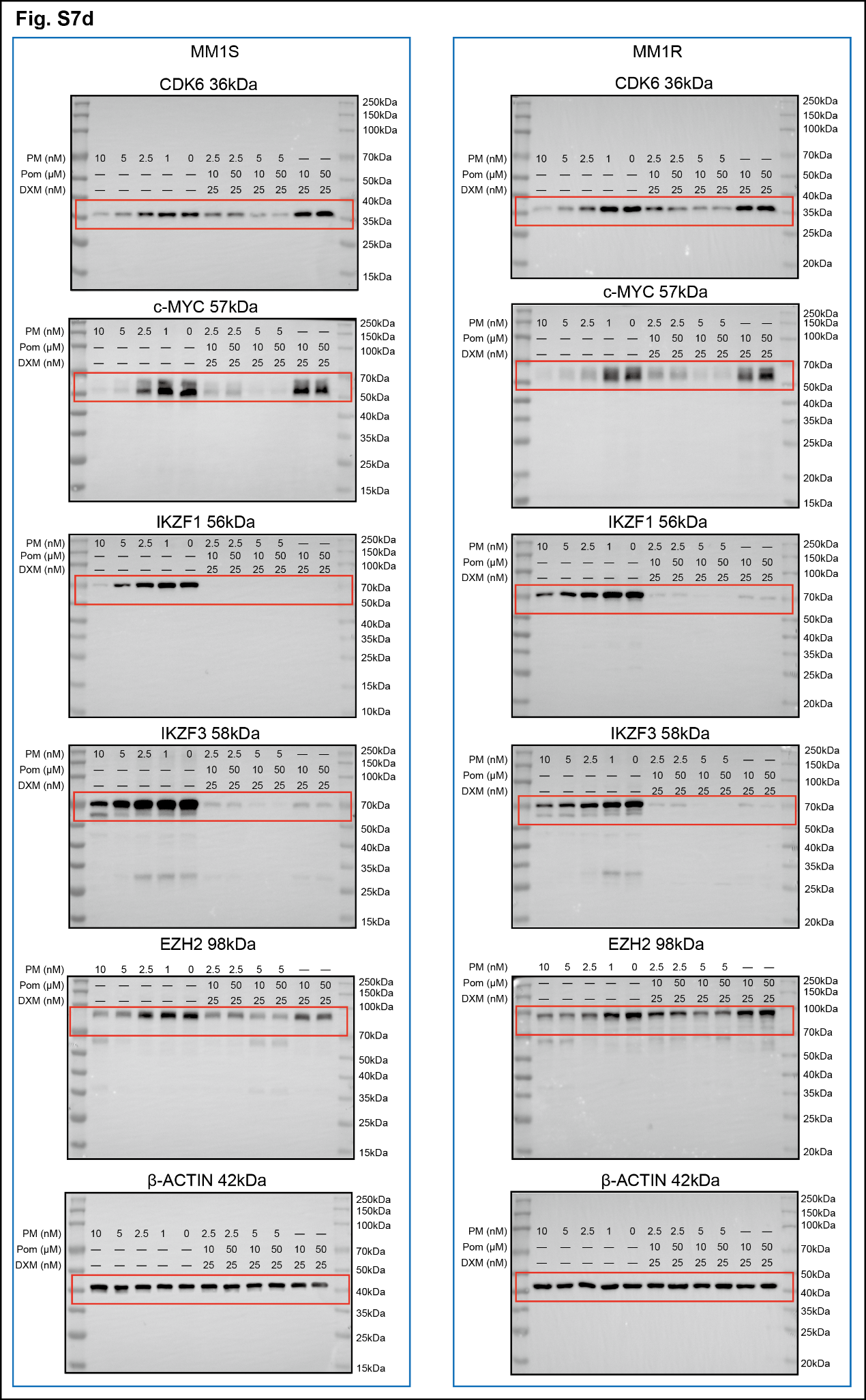

Supplement: Supplementary file 3 — All original and uncropped whole membrane images of western blot results in the manuscript [file 41392_2025_2285_MOESM3_ESM.docx]
